# Identification of a Vitamin-D Receptor Antagonist, MeTC7, which Inhibits the Growth of Xenograft and Transgenic Tumors *In Vivo*

**DOI:** 10.1021/acs.jmedchem.1c01878

**Published:** 2022-04-11

**Authors:** Negar Khazan, Kyu Kwang Kim, Jeanne N. Hansen, Niloy A. Singh, Taylor Moore, Cameron W. A. Snyder, Ravina Pandita, Myla Strawderman, Michiko Fujihara, Yuta Takamura, Ye Jian, Nicholas Battaglia, Naohiro Yano, Yuki Teramoto, Leggy A. Arnold, Russell Hopson, Keshav Kishor, Sneha Nayak, Debasmita Ojha, Ashoke Sharon, John M. Ashton, Jian Wang, Michael T. Milano, Hiroshi Miyamoto, David C. Linehan, Scott A. Gerber, Nada Kawar, Ajay P. Singh, Erdem D. Tabdanov, Nikolay V. Dokholyan, Hiroki Kakuta, Peter W. Jurutka, Nina F. Schor, Rachael B. Rowswell-Turner, Rakesh K. Singh, Richard G. Moore

**Affiliations:** † Wilmot Cancer Institute and Division of Gynecologic Oncology, Department of Obstetrics and Gynecology, University of Rochester Medical Center, Rochester New York 14624, United States; ‡ Department of Pediatrics, University of Rochester Medical Center, Rochester, New York 14642, United States; § Department of Biostatistics and Computational Biology, University of Rochester Medical Center, Rochester, New York 14624, United States; ∥ Division of Pharmaceutical Sciences, Okayama University Graduate School of Medicine, Dentistry and Pharmaceutical Sciences, Kita-ku, Okayama 700-8530, Japan; ⊥ Division of Surgery and of Microbiology and Immunology, University of Rochester Medical Center, Rochester, New York 14624, United States; # Department of Surgery, Division of Surgical Research, Rhode Island Hospital, Alpert Medical School of Brown University, Providence, Rhode Island 02903, United States; ∇ Department of Pathology and Laboratory Medicine, University of Rochester Medical Center, Rochester, New York 14624, United States; ○ Department of Chemistry and Biochemistry, University of Wisconsin Milwaukee, Milwaukee, Wisconsin 53211, United States; ◆ Department of Chemistry, Brown University, Providence, Rhode Island 02912, United States; ¶ Department of Chemistry, Birla Institute of Technology, Mesra, Ranchi 835215, India; †† Genomics Core Facility, Wilmot Cancer Center, University of Rochester Medical Center, Rochester, New York 14624, United States; ‡‡ Department of Pharmacology and Department of Biochemistry and Molecular Biology, Penn State College of Medicine, Penn State University, Hershey, Pennsylvania 17036, United States; §§ Department of Radiation Oncology, University of Rochester Medical Center, Rochester, New York 16424, United States; ∥∥ Center for Breast Health and Gynecologic Oncology, Mercy Medical Center, 271 Carew Street, Springfield, Massachusetts 01104, United States; ⊥⊥ Rutgers, The State University of New Jersey, 59 Dudley Road, New Brunswick, New Jersey 08019, United States; ## CytoMechanobiology Laboratory, Department of Pharmacology, Penn State College of Medicine, Pennsylvania State University, Hershey, Pennsylvania 17036, United States; ∇∇ School of Mathematical and Natural Sciences, Arizona State University, Health Futures Center, Phoenix, Arizona 85054, United States; ○○ University of Arizona College of Medicine, Phoenix, Arizona 85004, United States; ◆◆ Departments of Pediatrics, Neurology, and Neuroscience, University of Rochester Medical Center, Rochester, New York 14642, United States

## Abstract

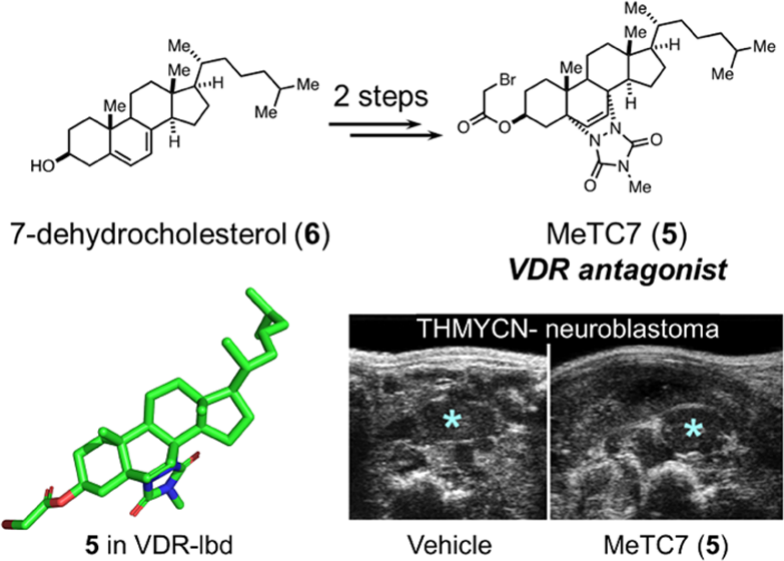

Vitamin-D receptor (VDR) mRNA is overexpressed in neuroblastoma and carcinomas of lung, pancreas, and ovaries and predicts poor prognoses. VDR antagonists may be able to inhibit tumors that overexpress VDR. However, the current antagonists are arduous to synthesize and are only partial antagonists, limiting their use. Here, we show that the VDR antagonist MeTC7 (**5**), which can be synthesized from 7-dehydrocholesterol (**6**) in two steps, inhibits VDR selectively, suppresses the viability of cancer cell-lines, and reduces the growth of the spontaneous transgenic TH-MYCN neuroblastoma and xenografts *in vivo*. The VDR selectivity of **5** against RXRα and PPAR-γ was confirmed, and docking studies using VDR-LBD indicated that **5** induces major changes in the binding motifs, which potentially result in VDR antagonistic effects. These data highlight the therapeutic benefits of targeting VDR for the treatment of malignancies and demonstrate the creation of selective VDR antagonists that are easy to synthesize.

## Introduction

**Figure 1:**
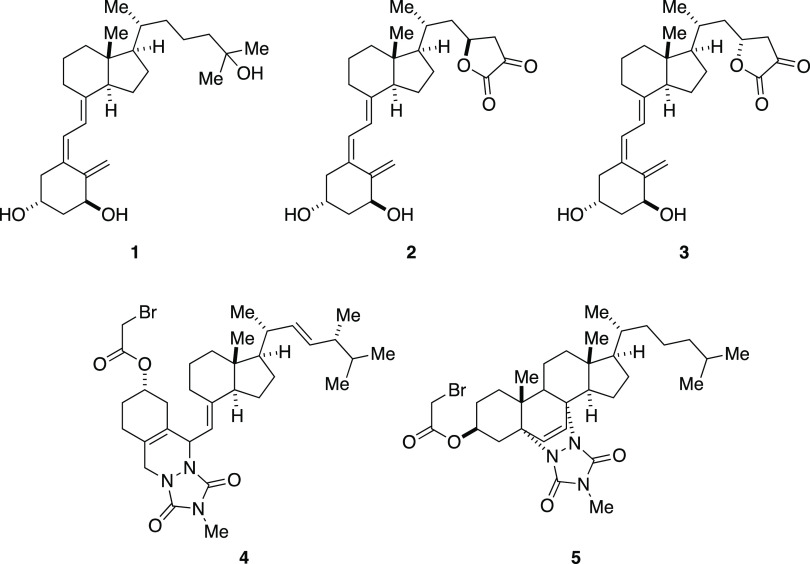
Chemical structures of calcitriol (**1**) and the representative literature-described VDR antagonists TEI-9647 (**2**), TEI-9648 (**3**), and MT19c (**4**). Chemical structure of the new VDR antagonist MeTC7 (**5**).

Carcinoma of ovaries and pancreas, neuroblastoma, and medulloblastoma remain life-threatening.^1^ Analyses of the cancer patient’s microarray databases demonstrate that Vitamin-D receptor (VDR) mRNA is overexpressed in the carcinomas of pancreas, ovaries, bladder, glioma, liver, and lungs and in neuroblastoma and predicts poor prognosis. VDR is also enriched in hyperplastic polyps and endometriosis and in early stages of tumorigenesis.^2−5^ Causes of VDR overexpression in malignancies, polyps, and other disease states are unclear and require further investigations. VDR, a class-III nuclear receptor (NR), mediates physiologic actions of calcitriol (**1**)^2^ (Figure 1), the hormonally active form of Vitamin-D. Calcitriol has been tested in human trials for the treatment of various malignancies,^6−12^ but it induces hypercalcemia, which is an undesirable side effect in patients.^13^**1** is currently used in the management of plaque psoriasis, hyperparathyroidism, and nephropathy.^14,15^

Further, in addition to VDR, RXRα, the heterodimerization partner, which is necessary for DNA binding and recruitment of coregulators, is altered in malignancies and predicts poor prognosis.^16,17^ Similarly, Importin-4, essential for nuclear internalization of VDR, is aberrantly altered in malignancies and exhibits poor prognosis.^18^ Furthermore, VDR was shown to induce MYCN overexpression.^19^ MYCN is overexpressed in over 70% of the malignancies.^20^ In addition, our ongoing studies and published literature show that Vitamin-D/VDR upregulates PD-L1 in cancer cells.^21^ Therefore, we postulate that a VDR antagonist is needed to block tumorigenesis orchestrated by aberrant VDR and the associated signaling nodes VDR/RXRα/Importin-4, VDR/MYCN, and VDR/PD-L1.

Progress in inhibiting VDR has remained hampered by the unavailability of pharmacologically pure VDR antagonists.^22,23^ Currently known VDR antagonists can exhibit residual agonistic effects,^22,23^ and their therapeutic effects have yet to be evaluated in animal models for malignancies. In addition, the synthesis of the literature-described VDR antagonists including TEI-9647 (**2**) and TEI-9648 (**3**) (Figure 1) requires multiple synthesis steps and is challenging. To overcome these problems, we have attempted to develop easily synthesized VDR antagonists.^24^ In this context, we had previously reported MT19c (**4**) (Figure 1) as a new class of VDR antagonists that actually showed very strong antitumor activity in animal models.^25^ However, the low VDR antagonist activity of **4** remained an issue.

In this report, we describe our efforts to identify a novel VDR antagonist MeTC7 (**5**). **5** can be synthesized from 7-dehydrocholesterol (7DHC) (**6**) in two steps. We investigate the VDR selectivity of **5** and perform *in silico* studies to understand how **5** affects the VDR-ligand-binding domain (VDR-LBD) versus calcitriol. *In vitro* and *in vivo* experiments using **5** were performed to understand the effect of VDR inhibition on RXRα and Importin-4 and MYCN expression, the critical VDR downstream signaling nodes, and to examine its effects on the growth of ovarian cancer, neuroblastoma, pancreatic cancer, and medulloblastoma cells. Based on the described role of VDR in MYCN’s expression in neuroblastoma,^19^ we examine the effects of **5** treatment on the growth of spontaneous neuroblastoma using a homozygous tyrosine hydroxylase (TH)-MYCN transgenic model^26^ and investigate its effects on the population of hematopoietic cells as a measure of off-target effects. Our studies show that **5** is a selective VDR antagonist endowed with promising antitumor effects against xenograft and transgenic spontaneous tumor models.

## Results

### Molecular Design and Synthesis of MeTC7 (**5**)

Our approach of developing a novel VDR antagonist pharmacophore, of which MT19c (**4**)^24,25^ is a previously described derivative, involves heterocyclizing the biological activity-endowed secosteroidal scaffolds (Vitamin-D2/D3 and ergocalciferol^24,25^) via Diels–Alder reactions with dienophiles (MTAD, PTAD) to (1) disable interactions with 1-α hydroxylase, which pivots the classical Vitamin-D signaling including calcium regulations; and (2) convert the purely carbonaceous scaffold to heteroatom-rich druglike pharmacophores carrying balanced charge/lipophilicity ratios. This strategy generates unique heterocycle-fused conformationally constrained novel pharmacophores in a short-path and atom-economy manner that can be variously derivatized further to generate bioactive compounds that are antagonists to VDR, are void of residual agonistic effects, and are highly nuclear receptor-selective. Our strategy differs from the one followed for the development of TEI-9647 (**2**) and TEI-9648 (**3**), which carry the Michael acceptor lactone ring in their side chains keeping the A-ring unaltered. Probably, residual VDR agonistic effects in **2** and **3** arise because the A-ring is accessible for interactions with 1-α hydroxylase; for similar reasons, **2** and **3** may also exhibit hypercalcemia if administered in animals. To improve upon the weak VDR antagonist activity of **4**, we derivatized 7DHC (**6**) to build our new pharmacophore and MeTC7 (**5**) as the key derivative. **5** was designed to carry out additional rigidity in the backbone structure compared to Vitamin-D2/D3 and ergocalciferol.

**Scheme 1:**
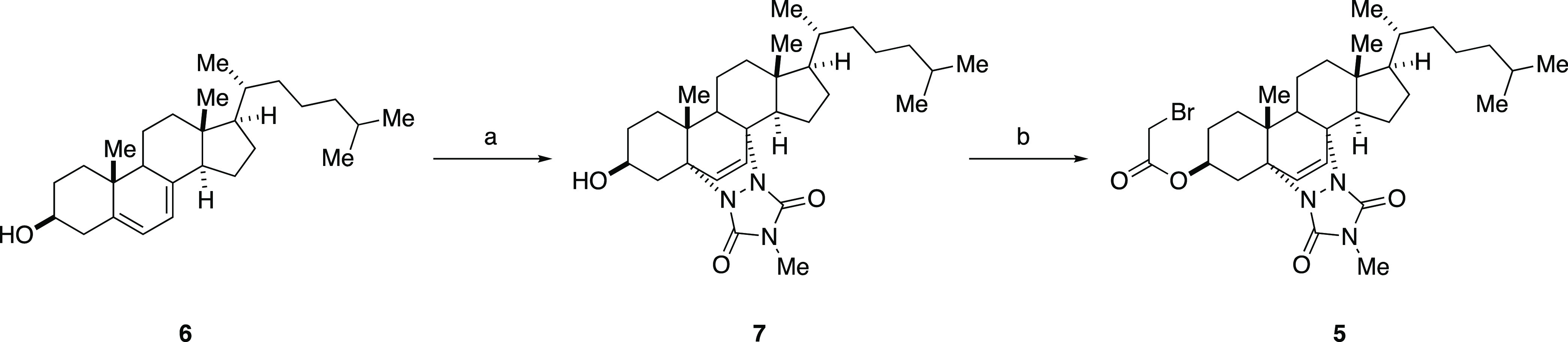
Reagents and Conditions. (a) MTAD, DCM, 0 °C; (b) Bromoacetic Acid, DCC, DCM, 0 °C to rt

MeTC7 (**5**) was synthesized by the method shown in Scheme 1. 7DHC (**6**) was reacted with *N*-methyl-1,2-4-triazolinedione (MTAD) in dichloromethane (DCM) at 0 °C to afford **7** at 52% yield. Then, **7** was reacted with bromoacetic acid in the presence of *N*,*N*′-dicyclohexylcarbodiimide (DCC) and 4-dimethylaminopyridine (DMAP) in anhydrous dichloromethane, and the reaction mixture was purified by a preparative thin-layer chromatography plate to give **5** at 67% yield. Characterization data for **5** and **7** are shown in Figure S1.

### MeTC7 (**5**): An NR-Selective VDR Antagonist

**Figure 2:**
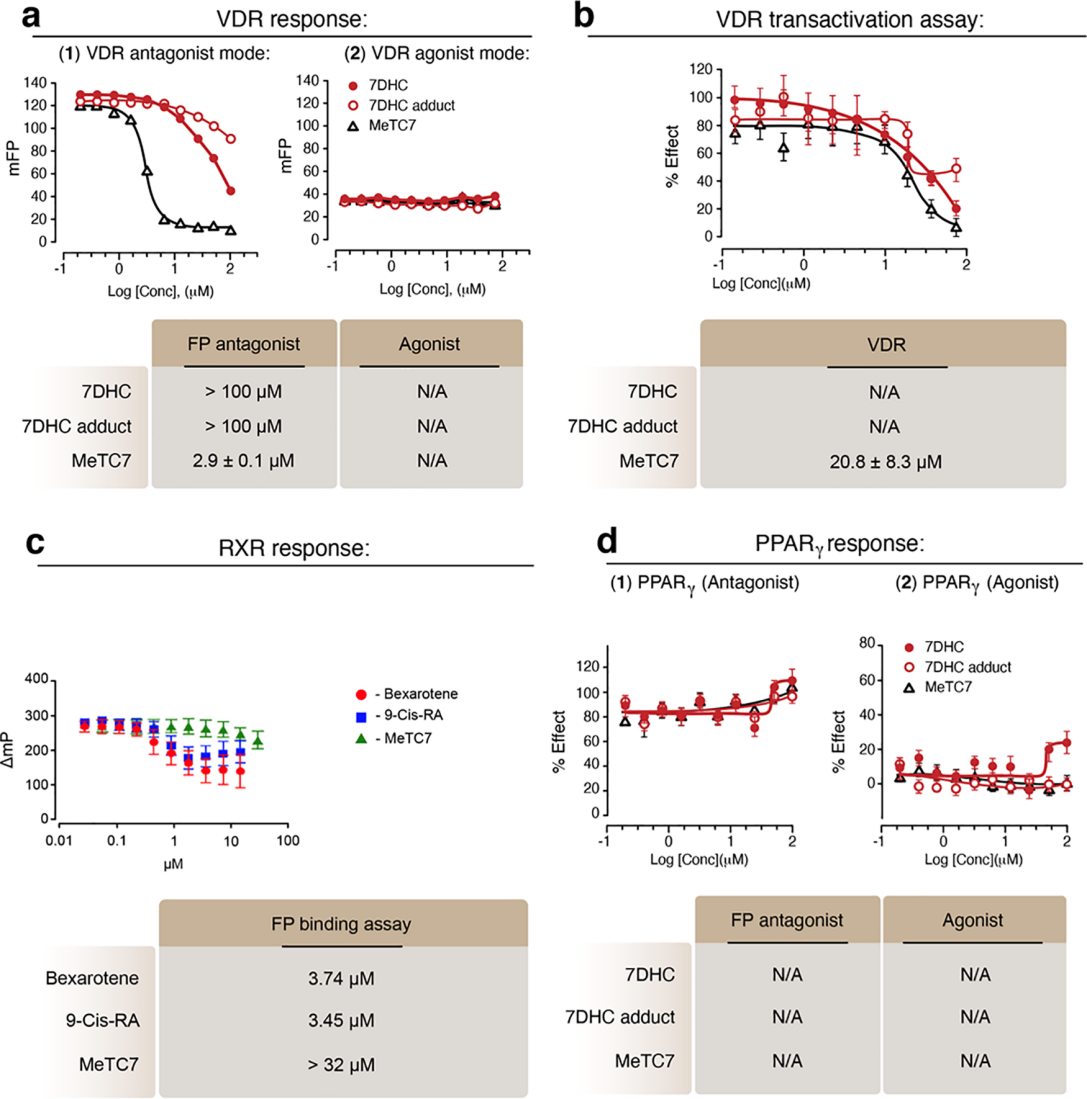
Pharmacologic characterizations of MeTC7 (**5**). (a) Fluorescence polarization assay showing VDR inhibition by **5** (left) without induction of agonistic effects (right). (b) **5** inhibited VDR transactivation in a HEK293 cell-based assay. (c) **5** did not bind to RXRα. (d) **5** did not exhibit agonistic or antagonistic effects against PPAR-γ.

MeTC7 (**5**) showed potent VDR inhibition (IC50 = 2.9 ± 0.1 μM) (Figure 2a, left) in a fluorescence polarization (FP) assay performed using VDR-LBD, SRC2-3 Alexa Fluor 647 and 10 nM of 1.^27,28^ 7-Dehydrocholesterol (7DHC, **6**) and 7DHC-adduct (**7**) did not inhibit VDR (IC_50_ > 100 μM). Fluorescence polarization studies showed that **5** is void of any VDR agonistic activity (Figure 2a, right). In a cell-based transactivation assay, **5** inhibits VDR transactivation in the concentration range of (IC_50_ = 20.8 + 8.3 μM) (Figure 2b). **6** and **7** did not inhibit VDR transactivation (IC_50_ > 100 μM) (Figure 2b). Since lack of NR selectivity is a major challenge in developing NR modulators,^29^ next, we investigate whether **5** binds RXRα, the heterodimeric binding partner of VDR. Fluorescence polarization assay^30^ showed that **5** treatment does not bind RXRα, while Bexarotene (IC_50_ = 3.74 μM) and 9-*cis*-retinoic acid (IC_50_ = 3.45 μM) showed potent binding (Figure 2c). Similarly, **5** did not show agonistic or antagonistic effects against PPARγ (Figure 2d), another NR, until the tested doses of 100 μM.

### MeTC7 (**5**) Disrupts the VDR-Ligand-Binding Domain *In Silico*

**Figure 3:**
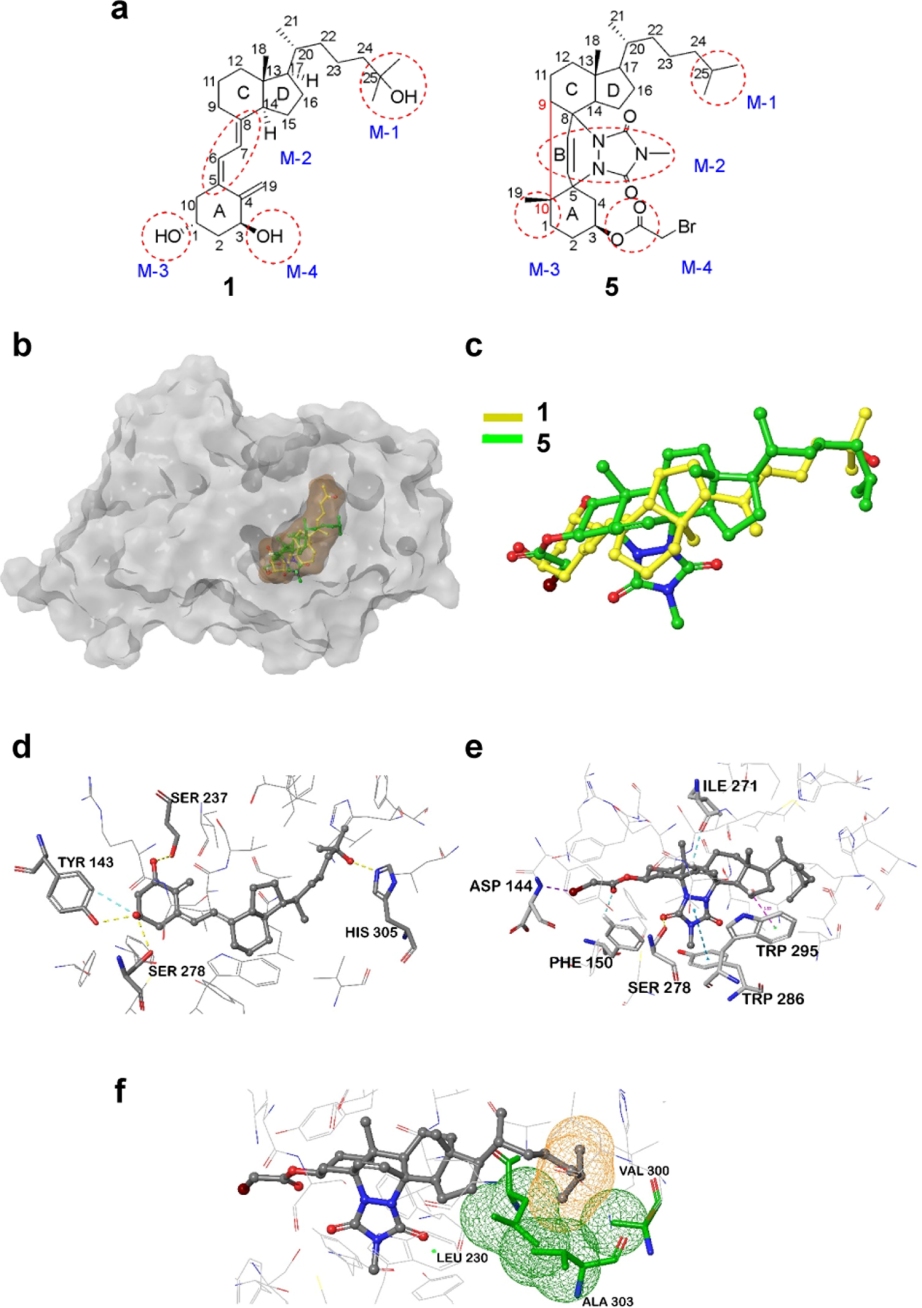
Putative 3D-binding modes of **5** in VDR-LBD with noncovalent interactions. (a) For *in silico* binding to VDR-LBD, **1** and **5** were marked into four structurally relevant zones (M1–M4). Binding motifs (M) in **1** and **5** are highlighted by red dotted circles. The bond connection between atoms 9 and 10 of **5** (red) is shown far to get better clarity of the M2 motif. However, atoms 9 and 10 are close to the normal C–C bond in **5** in which A and B rings fuse together to form the conformationally rigid ring system. (b) Surface view with the VDR-active site showing the binding mode overlay of **1** (yellow) (movie 1) and **5** (green) in LBD (brown) (movie 2). (c) Superposition overlay of **1** (yellow, cocrystal structure) and **5** (green) into the VDR-ligand-binding domain (LBD). (d) 3D-binding mode of **1** (crystallographic structure) showing major noncovalent interactions with VDR-LBD. (e) 3D-binding mode of **5** showing major noncovalent interactions such as C–H...π, C–H...O, and H-bonding. (f) **5** interacts with Leu230, Ala303, and Val380 through favorable van der Waal contacts shown by the mesh surface diagram (green for LBD residues and yellow for **5**).

To elucidate the structural mechanisms forming antagonistic attributes of **5**, *in silico* studies are performed to determine the interactions of **5** with VDR-ligand-binding domain (VDR-LBD) residues. The crystal structure of VDR-LBD (1DB1) cocrystallized with 2-α-(3-hydroxy-1-propyl) calcitriol^31^ is used for docking studies. **1** is visualized to consist of a ring-A, a conjugated linker, ring C, and ring D along with a flexible chain (Figure 3a, left). The simultaneous interactions mediated by C1, C3, and C25-hydroxyl groups are crucial for the super agonistic behavior of **1**.^31^ Synchronized interaction is possible only if the correct spacing exists between the hydroxyl groups, which is achieved by proper folding within the molecular structure of **1** to favor the correct orientation of the hydroxyl group. Structurally, **5** (Figure 3a, right) is much larger in size and is a highly conformationally rigid system compared to calcitriol. Three-dimensional (3D) binding of **5** (green) in LBD (brown) of VDR (white surface) is shown by the surface diagram and overlaid with **1** (yellow) in Figure 3b. Binding interactions of **5** with VDR-LBD are shown in Figure 3b. An overlaid superposition for **1** and **5** is shown in Figure 3c. To handle this large and rigid molecule, the induced-fit (IF) docking strategy^32−35^ was implemented to reveal the possible binding modes of **5** with VDR-LBD. **1** shows the involvement of the −OH group in H-bonding with VDR residues (Ser278 and His305) (Figure 3d). Binding of **5** shows the recruitment of C–H...π, C–H...O, and H-bonding with VDR-LBD active site residues Trp286, Tyr147, Asp144, and Ser237 (Figure 3e) due to the major changes in the binding motifs (M1–M4), which potentially result in an antagonistic effect.^32^ The shortening and removal of the C25-OH group near the M1 motif in **5** cause the loss of interaction with His305. His305 along with Arg274 residues play a crucial role in determining the agonistic behavior of **1** (Figure 3d). The loss of the conjugated linker system in **5** followed by the shortening distance (2.6 Å) in comparison to **1** (3.7 Å) between rings A and C appears to induce the antagonistic behavior in **5**. Thus, the triazolidine-dione moiety and the hydrophobic *N*-methyl group occupy a similar spatial position within VDR as was placed by the hydrophilic C1-OH of calcitriol. Thus, **5** loses H-bonding with Arg274; however, carbonyl group’s interaction with Ser237 supports the strong binding of **5** (Figure 3e,f). Further, the conjugated diene linker of **1** enters tightly into the hydrophobic cavity of the VDR and agonizes the system^31^ (Figure 3d). In contrast, the triazoline-3,5-dione moiety of **5** increases the volume of this VDR-LBD cavity and locks the conformational freedom of VDR due to the deeper binding (Figure 3e,f). Further, interactions of Tyr143 and Arg274 with 1,3-OH of the A-ring of **1** (A-ring subsite) were lost when **5** bonded with VDR-LBD (Figure 3e,f). **5** interacts with a strong H-bonding with a backbone of Asp144 and Ser237 in the ring-A subsite (Figure 3e). **5** utilizes only hydrophobic residues in the 25-OH-subsite, whereas **1** uses a hydrophilic interaction (Ser306 and His305) as well. **5** interacts with Leu230, Ala303, and Val380 through favorable van der Waal’s contacts shown by a mesh surface diagram (green for LBD residues and yellow for **5**). The videos (**1** and **2**) exhibit the interactions of **1** and **5** with VDR-LBD residues.

### VDR mRNA Overexpression Showed Poor Prognosis in Pancreatic, Lung, Breast, Liver, Ovarian, Cervical, And Bladder Cancers and in Glioma and Neuroblastoma Patients

Kaplan–Meier survival analyses at the system-selected expression cutoffs (microarray data and tools available at R2-Genomics Analysis and Visualization Platform https://hgserver1.amc.nl/cgi-bin/r2/main.cgi) showed that VDR mRNA enrichments correlated with increased mortalities in lung (*p* = 0.0043) and pancreatic cancer patients (*p* = 0.004), neuroblastoma patients (*p* = 1.7 × 10^–5^), breast cancer (*p* = 0.011), glioma (*p* = 0.0048), cervical cancer (*p* = 0.055), liver cancer (*p* = 0.048), ovarian cancer (*p* = 0.09), and bladder cancer (*p* = 0.05) patients (Figure S2A–I). The effect of disease stages on the association of VDR mRNA enrichments with decreased mortalities in cancer patients was analyzed. Among stage IIa/IIb pancreatic cancer patients, VDR mRNA enrichment was strongly associated with increased mortalities (data not shown). The identity of microarray databases analyzed is described in the Materials and Methods section.

### MeTC7 (**5**) Suppresses RXRα and Importin-4 Expressions in the Ovarian Cancer Cell-Line

**Figure 4:**
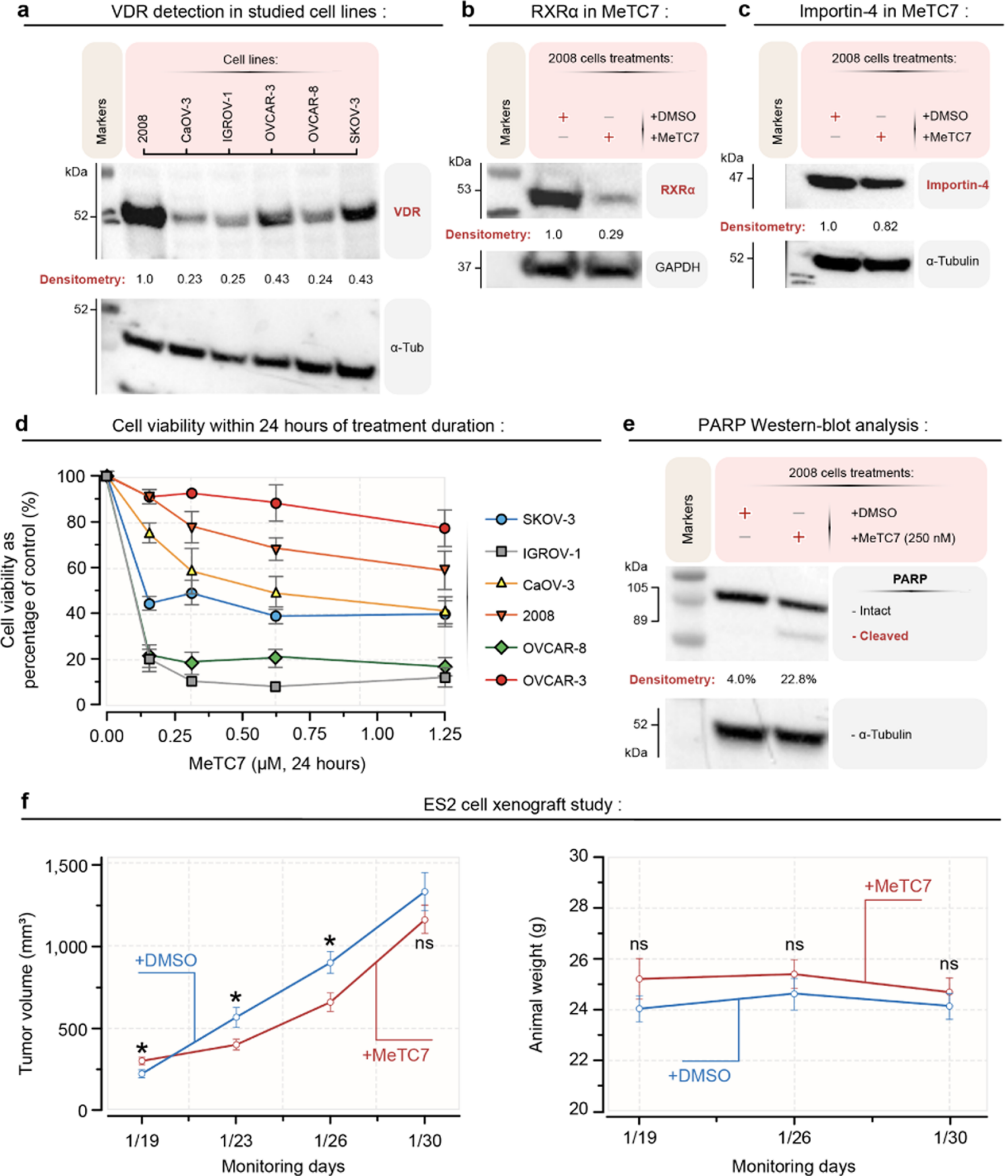
(a) VDR expression in a panel of 2008, IGROV-1, CaOV-3, OVCAR-3, OVCAR-8, and SKOV-3 ovarian cancer cell-lines. Expression of α-tubulin as a loading control is shown. Normalized western blot densitometric data are shown numerically. (b) Treatment with **5** (250 nM, 18 h) reduces the expression of RXR-α in 2008 cells. Expression of GAPDH as a loading control is shown. Normalized western blot densitometric data is shown numerically. (c) Treatment with **5** (250 nM, 18 h) reduces the expression of Importin-4 in 2008 cells. Expression of α-tubulin as a loading control is shown. Normalized western blot densitometric data are shown numerically. (d) **5** reduces the viability of SKOV-3, IGROV-1, CAOV-3, OVCAR-3, OVCAR-8, and 2008 ovarian cancer cell-lines during 24 h of treatment. Data + standard error of the mean (SEM) are expressed as the mean of the triplicate determinations as the % of absorbance by dimethylsulfoxide (DMSO)-treated cells set equal to 100%. (e) Treatment with **5** (250 nM, 18 h) increases cleaved PARP1 expression in 2008 cells. Expression of α-tubulin as a loading control is shown. Normalized western blot densitometric data are shown numerically. (f-left) **5** (*n* = 10, 10 mg/kg. M-F, IP) treatment reduces the growth of ES2 ovarian carcinoma-derived xenografts in NSG mice compared to the vehicle (*n* = 10). At the baseline, the **5** group has a statistically larger volume to begin with compared to the vehicle (*p* = 0.02). At the second and third time points, the **5** group has a statistically smaller tumor volume compared to the controls (*p* = 0.0303 and 0.0119, respectively). At the final time point, there is no statistical difference between the treatment groups with respect to the tumor volume. (f-right): There is no statistical difference in animal weights across time between treatment groups (*p* = 0.5437).

Screening a panel of SKOV-3, OVCAR-3, OVCAR-8, CaOV-3, IGROV-1, and 2008 ovarian cancer cell-lines by immunoblotting identifies 2008 and SKOV-3 cells as the high VDR expressor cell-lines suitable for VDR/**5** signaling studies (Figure 4a, upper). The expression of α-tubulin as the loading control in these cells is shown (Figure 4a, lower). **5** inhibited the expression of RXRα in 2008 ovarian cancer cell-lines (Figure 4b). RXRα expression correlates with poor prognosis in ovarian cancer patients (Figure S3). Importin-4 mediates nuclear translocation of VDR.^36^ VDR showed colocalization with Importin-4 in ovarian cancer tissues (Figure S4a) and indicated poor prognosis in neuroblastoma (Figure S4b). **5** (250 nM, 12 h) treatment suppressed Importin-4 expression in 2008 cell-lines (Figure 4c).

### MeTC7 (**5**) Inhibits the Viability of Ovarian Cancer Cells and Induces PARP1 Cleavage

A panel of SKOV-3, OVCAR-3, OVCAR-8, IGROV-1, CAOV-3, and 2008 ovarian cancer cell-lines showed a dose-dependent response to **5** treatment (Figure 4d). OVCAR-8, 2008, SKOV-3, CAOV-3, and IGROV-1 cells were sensitive to **5** treatment, while OVCAR-3 was relatively resistant against **5** treatment. Cleaved PARP1 expression in 2008 cells upon treatment with **5** was observed (Figure 4e).

### MeTC7 (**5**) Reduces the Growth of Xenografts Derived from Ovarian Cancer, Medulloblastoma, and Pancreatic Cancer Cells

**Figure 5:**
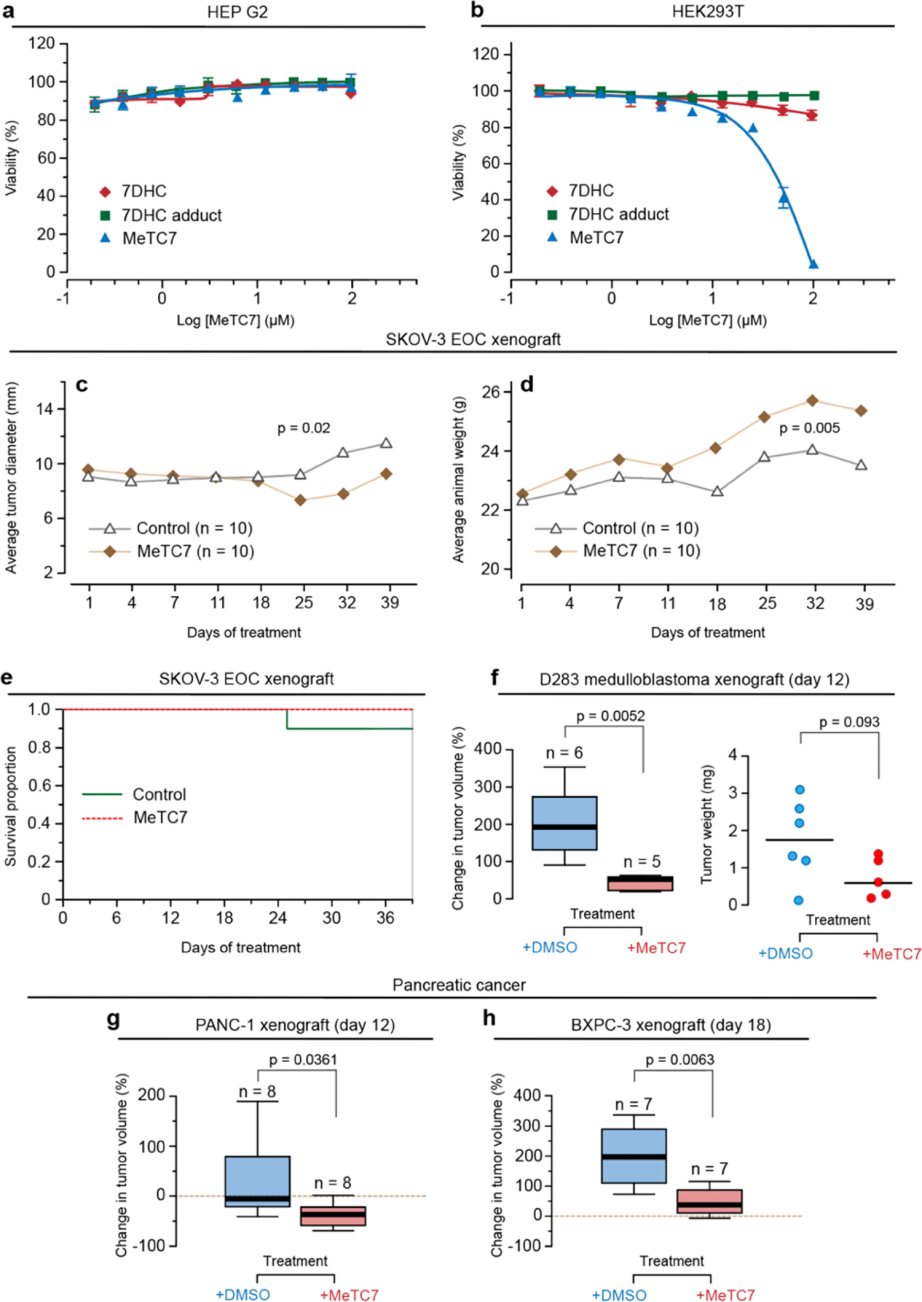
Selective antiproliferative functions and antitumor activities of MeTC7 (**5**) in xenograft animal models. (a) **5** does not inhibit the proliferation of HepG2 cells in the dose ranges tested. (b) **5** does not inhibit the proliferation of HEK293T cells in the dose ranges tested. (c) **5** (10 mg/kg, M-F, IP) treatment reduces the growth of SKOV-3 cell-derived xenografts in nude mice. (d) Animal weights of the mice undergoing treatment with vehicle or **5** increase during the period of observations. (e) The control group witnessed a death on day 25, indicated by the drop in the line. The dashed line for **5** indicates 100% survival. (f-left) **5** (10 mg/kg, M-F, IP) treatment reduces the growth of D283 medulloblastoma cell-derived xenografts in NSG mice. % change in average tumor volume in the treatment group was lower compared to the vehicle on day 12. (f-right) Mice were euthanized, and tumors were extracted and weighed. The tumor weights in the treatment group show a tendency to be smaller (unpaired *t*-test, *p* = 0.093). (g, h) **5** (10 mg/kg, M-F, IP) treatment reduces % change in the growth of PANC-1 and BXPC-3 cell-derived xenografts growing in NSG mice (PANC-1, day 12, *p* = 0.0361) and (BXPC-3, day 18, *p* = 0.0063). Statistical differences between the groups were analyzed using GraphPrism.

MeTC7 (**5**) treatment slowed the growth of the clear-cell ovarian carcinoma cell-line ES2-derived xenografts growing in NSG mice despite starting with significantly higher basal tumor sizes in the treatment group (*p* < 0.05 for the first two treatments, Figure 4f, left). The difference between tumor sizes in the control and treatment groups at the final treatment is not statistically significant. The difference between the animal weights in the treatment and control groups is not statistically significant (Figure 4f, right). While ovarian cancer cell-lines show sensitivity to **5** treatment, HepG2 (hepatocellular carcinoma) cell-lines and HEK293T (immortalized human embryonic kidney, HEK, cell-line) exhibit resistance to **5** treatment until 100 μM concentrations (Figure 5a,b). In addition to ES2, **5** treatment reduces the growth of SKOV-3 serous ovarian cancer cell-derived xenograft tumors (*p* = 0.02, Figure 5c). The rate of weight gain was significantly higher in the **5** group than in the control group over the time period (*p* = 0.005) (Figure 5d). Similar results were obtained using percentage change in weights relative to the baseline. The Kaplan–Meier analysis showed statistically greater survival prospects for the treatment groups (Figure 5e). Immunohistochemistry (IHC) shows reduced VDR expression in a randomly selected **5**-treated SKOV-3 xenograft tumor *in vivo* (Figure S5). **5** treatment also reduces the rate of growth of medulloblastoma D283 cell-derived xenografts in NSG mice, compared to the control (*p* = 0.032, Figure 5f). Harvested tumors post euthanasia showed a tendency to form smaller tumors in the treatment group than in the control (*p* = 0.093, Figure 5f, inset). Further, **5** treatment reduces the % change in the growth of PANC-1 (*p* = 0.036 on day 12) and BXPC-3 (*p* = 0.0063 on day 18) pancreatic cancer cell-derived xenografts in NSG mice (Figure 5g,h).

### MeTC7 (**5**) Inhibits the Growth of Neuroblastoma Cells and Their Xenografts

**Figure 6:**
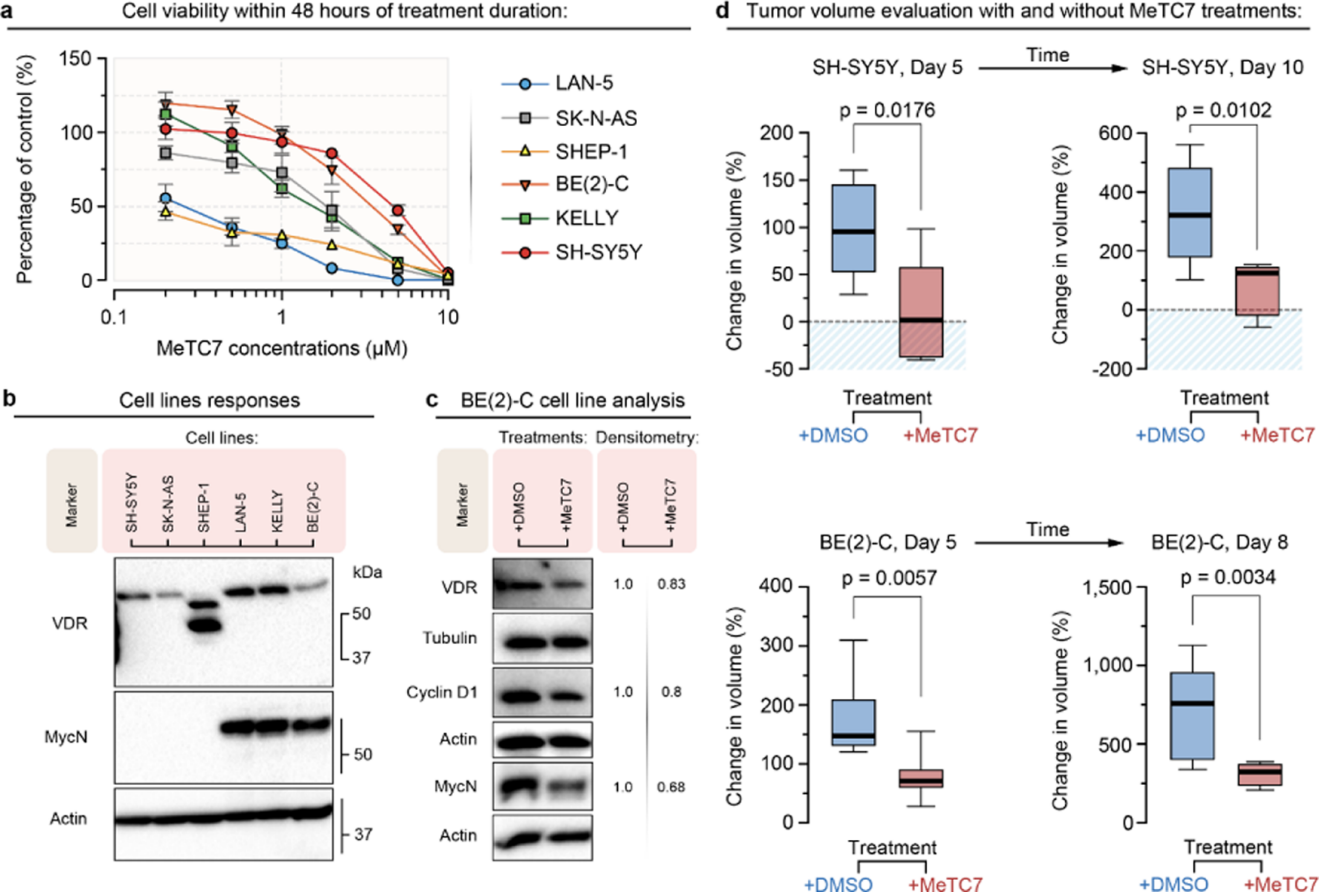
(a) MeTC7 (**5**) treatment for 48 h decreases the viability of LAN-5, SK-N-AS, SHEP-1, BE(2)-C, Kelly, and SH-SY5Y neuroblastoma cell-lines dose-dependently. Data represent ±SEM. (b) Immunoblot analysis shows relative VDR and MYCN levels in LAN-5, SK-N-AS, SHEP-1, BE(2)-C, Kelly, and SH-SY5Y cell-lines. Expression of β-actin as the protein loading control is shown. (c) **5** (1 μM, 24 h) treatment reduces VDR, MYCN, and cyclin-D expressions in the BE(2)-C cell-line. Expression of β-actin or α-tubulin as a protein loading control is shown. Normalized western blot densitometric data are shown numerically. (d-upper) **5** (10 mg/kg, M-F, IP) treatment reduced the growth rate of SH-SY5Y tumors growing in NSG mice. *T*-test analysis showed that % changes in tumor volumes are lower in the treatment group (day 5: *p* = 0.0176; day 10: *p* = 0.0102) than in the control (d-lower). **5** (10 mg/kg, M-F, IP) treatment reduced the growth rate of BE(2)-C xenograft tumors growing in NSG mice. *T*-test analysis showed that % changes in tumor volumes are lower in the treatment group (*p* = 0.0057 on day 5; *p* = 0.0034 on day 8) than in the control.

VDR mRNA overexpression is prognostic in neuroblastoma (Figure S2c). **5** treatment suppresses the viability of neuroblastoma cell-lines Lan-5, SK-N-AS, SHEP-1, BE(2)C, Kelly, and SH-SY5Y dose-dependently (Figure 6a). Immunoblotting shows that VDR and MYCN are expressed in neuroblastoma cell-lines (Figure 6b). Lan-5, Kelly, and BE(2)-C express both VDR and MYCN, whereas SK-N-AS and SHEP-1 are VDR-positive but MYCN-negative. **5** treatment reduces VDR and MYCN expressions in BE(2)C cell-lines (Figure 6c). *In vivo*, **5** (10 mg/kg, M-F, IP) treatment reduces the growth rate of the SH-SY5Y xenograft as measured on day 5 (*p* = 0.018) and day 10 (*p* = 0.012) in NSG mice (Figure 6d, upper). Similarly, % change in BE(2)C tumor volumes of **5**-treated mice is significantly lower than in vehicle-treated NSG mice (day 5, *p* = 0.0052; day 8, *p* = 0.0034) (Figure 6d, lower and Figure S6).

### MeTC7 (**5**) Reduces MYCN Expression and Blocks the Growth of TH-MYCN Transgene-Driven Spontaneous Neuroblastoma

**Figure 7:**
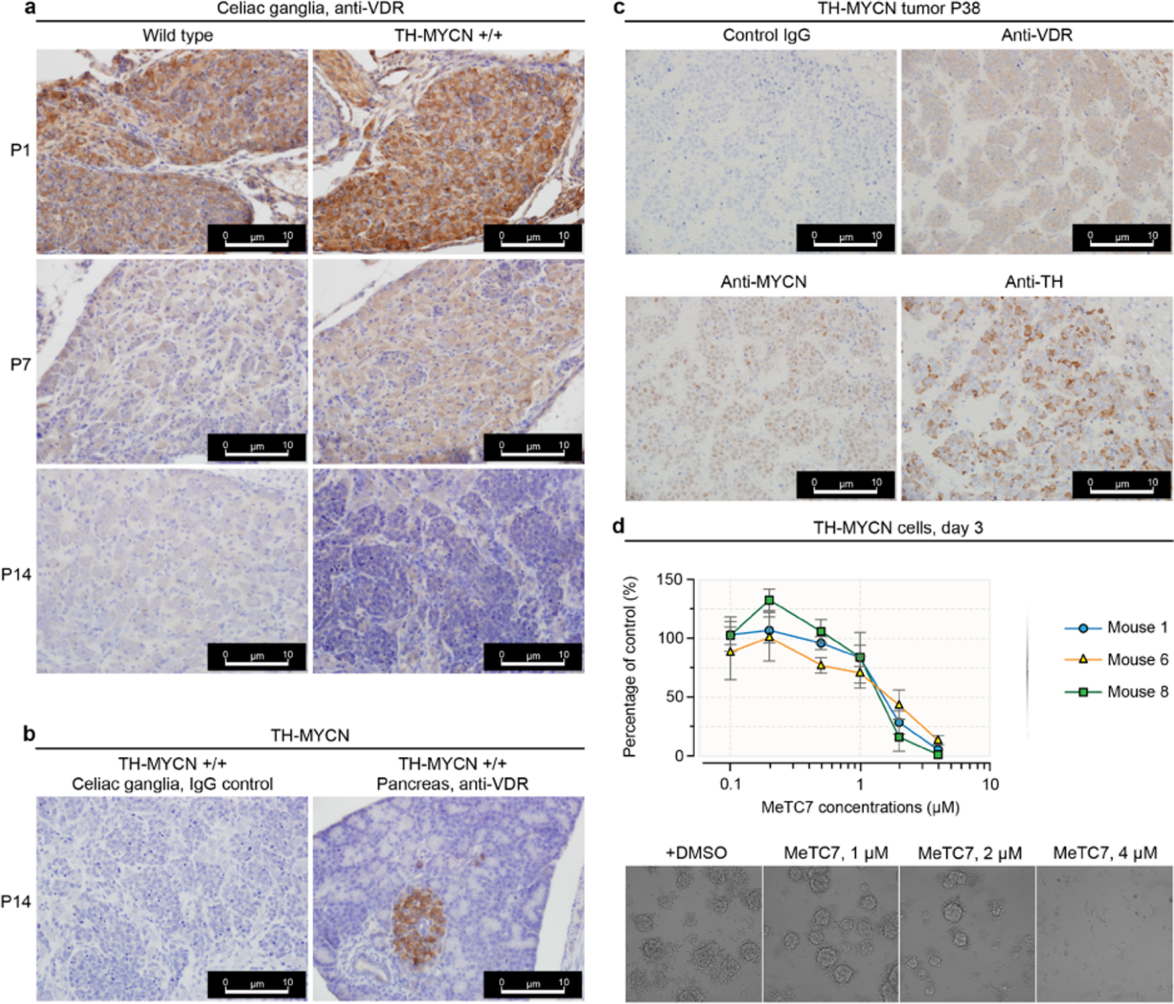
Immunohistochemistry in the tissues from homozygous TH-MYCN mice showed strong expressions of VDR, MYCN, and tyrosine hydroxylase (TH). (a) Compared to wild-type, celiac ganglia isolated from the TH-MYCN+/+ mice (postnatal days 1, 7, and 14) showed increased VDR expression. (b) Positive and negative controls used for IHC in postnatal mice. Serial section stained with normal immunoglobulin G (IgG) was used as a negative control for IHC staining. The expression of VDR in the pancreas of TH-MYCN mice was used as a positive control to verify the VDR antibody retrieval method and staining in the same sagittal section as the celiac ganglia. (c) Advanced TH-MYCN tumor isolated from a 5.5-week old mouse showed VDR, MYCN, and TH expressions in the tumors. (d-top) **5** treatment reduces the viability of TH-MYCN tumor cells isolated from the three independent mice (tags: 1, 6, and 8) during three days of treatment. Data represents ±SEM. (d-bottom) **5** treatment dose-dependently decreases the viability of murine neuroblastoma tumor spheres isolated from TH-MYCN mouse (tag: 1). Representative images of tumor spheres from the same cell viability experiment. Images were captured using an Olympus BX41 light microscope with an Olympus DP70 camera and CellSens digital software.

MYCN overexpression predicts poor overall and event-free survival in patients with ovarian cancer and neuroblastoma^37^ (Figure S7) and other malignancies.^38−45^ We investigate whether targeting VDR by **5** can inhibit MYCN expression and, in turn, control the MYCN-orchestrated neuroblastoma growth. Prior to testing antineuroblastoma activities of **5** using the well-established TH-MYCN+/+ transgenic mice, which spontaneously develop neuroblastoma and recapitulate human neuroblastoma disease closely,^26,46^ we establish via immunohistochemistry that celiac ganglia harvested from homozygous TH-MYCN+/+ mice exhibit positive expressions of VDR, MYCN, and TH antigens (Figure 7a–c). Similarly, an MTS cell viability assay run on the tumor cells derived from three independent homozygous TH-MYCN+/+ mice shows reduced viability of the spheroid cell’s viability upon **5** treatment (Figure 7d upper). Images of the tumor spheroids treated with vehicle or **5** are shown (Figure 7d lower).

**Figure 8:**
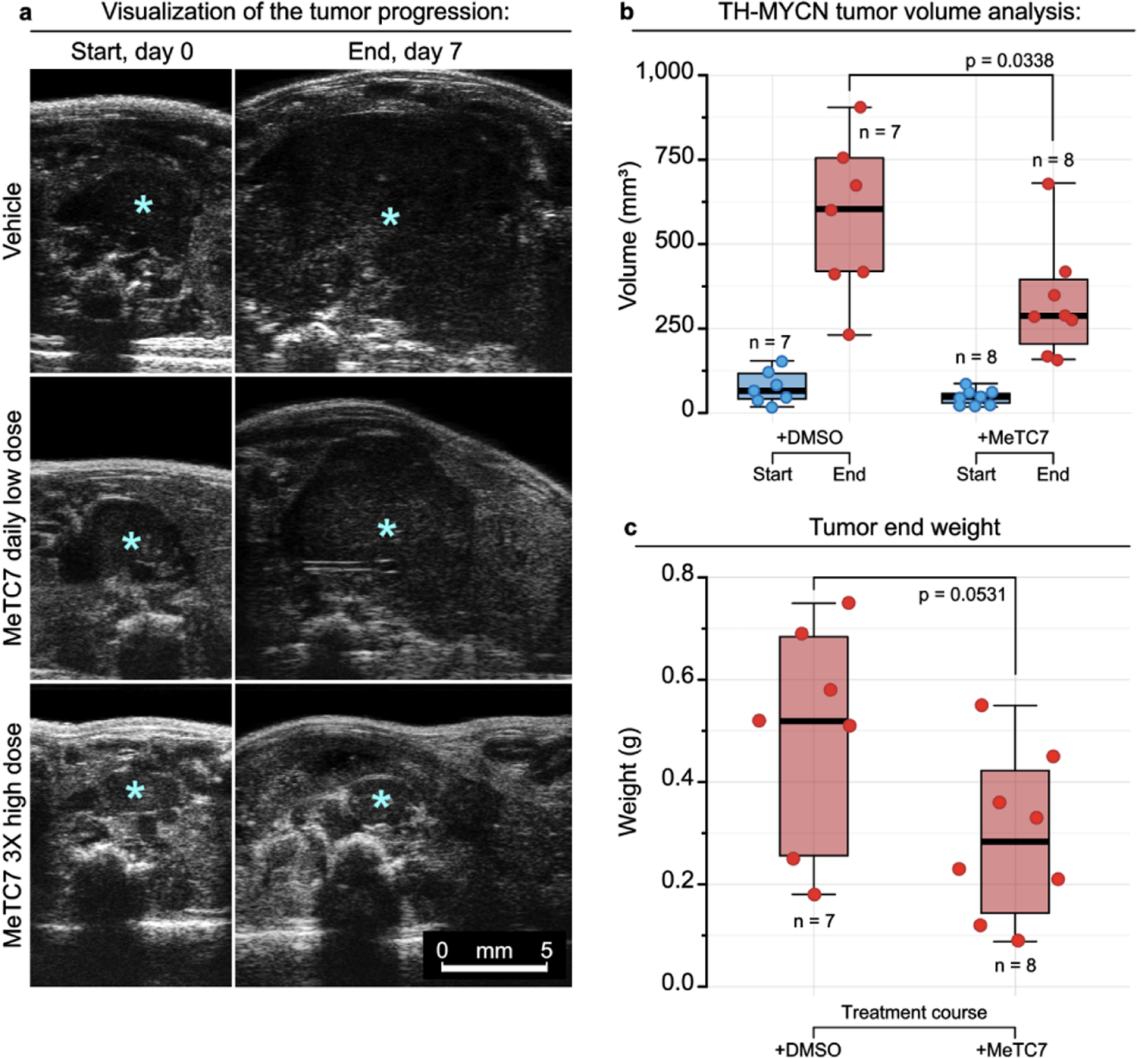
(a) Antitumor activity of MeTC7 (**5**) in the TH-MYCN model of spontaneous neuroblastoma. Compared to the vehicle (upper, *n* = 7), **5** (lower, 10 mg/kg, daily IP, *n* = 8) treatment reduced the growth of TH-MYCN-driven neuroblastoma in transgenic mice. Tumor burden was measured by an ultrasound imaging instrument. (b) **5** treatment dose-dependently reduced the TH-MYCN tumor volume at the end of the treatment. (c) Mice were euthanized, and extracted tumors were weighed. Tumor sizes and weights in the control versus treated groups were compared using a *T*-test.

Next, we investigate the effect of **5** against the growth of tumors in TH-MYCN+/+ transgenic mice. The response of the drug in alive mice was monitored using ultrasound. Images were reconstructed to capture the 3D tumor volume using inbuilt software. **5** reduces the tumor growth compared to the vehicle group (Figure 8a). Analysis of the estimated tumor volumes (*p* = 0.033) (Figure 8b) and harvested tumor weights (*p* = 0.053) (Figure 8c) exhibits the reduced tumor burden upon treatment with **5**.

### MeTC7 (**5**) Does Not Exhibit Off-Target Effects

Ruling out whether **5** treatment exerts off-target effects against hematopoietic cells, the common side effects of chemotherapies, the population of various CD45+ cell subtypes isolated from TH-MYCN mice was analyzed by flow cytometry using characterized markers. The analysis shows that **5** treatment does not affect populations of CD45+, CD4+, CD8+, macrophages, patrolling monocytes/DC+, and CD11b+ cells compared to the control (Figure S8).

### Proposed Mechanism of Action of MeTC7 against VDR

**Figure 9:**
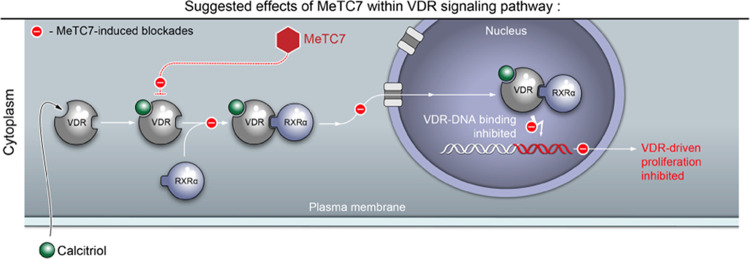
Cartoon outlining the putative mechanism of action of MeTC7 against VDR.

Based on the data described in the report above, the cartoon (Figure 9) summarizes the putative mechanism of action of MeTC7 against VDR.

## Discussion and Conclusions

Compared to agonists, the development of VDR antagonists has lagged behind.^23,24^ Following our previously described approach of the Diels–Alder modification of secosteroidal scaffolds that generated earlier classes of VDR antagonists [MT19c (**4**) and PT19c],^24,25,47^**5** was synthesized. **5** showed superior VDR inhibition than **4** and PT19c without incurring any agonistic activity and exhibited noteworthy NR selectivity against PPAR-γ and RXRα, the two closely related members of the VDR-NR family (Figure 2c,d). *In silico* studies show that heterocyclization of **6** by 1-methyl-1,2,4-triazolinedione instills enormous structural rigidity in **5**, which disrupts VDR-LBD. It has been shown that upon binding to the LBD of VDR, the antagonist complex converts into a transcriptionally inactive form.^35^ Loss of TYR143, HIS305, and ARG274 interactions, which are critical for hydrogen-binding interactions of **1**, may account for the antagonistic attributes of **5**, similar to the effects of mutations at HIS305 along with ARG274 residues, which was shown to generate antagonistic effects^35^ (Figure 3e–g). Structurally, **5** differs from the literature^23,24^-described VDR antagonists TEI-9647 (**2**) and TEI-9648 (**3**). **5** carries a highly constrained heteroatom-rich-tetracyclic ring system derived from the conjugated diene system of **6**, the precursor of Vitamin-D, whereas **2** and **3** retain the classic Vitamin-D scaffold but have the C25 carbon converted into a five-membered lactone ring. **5** sports an alkylating bromoacetoxy functionality, whereas a Michael acceptor ring likely forms the basis of **2** and **3** functions.

The rationale to identify a VDR antagonist such as MeTC7 (**5**) and investigate its antitumor effects stemmed from (1) VDR mRNA overexpression in ovarian, breast, lung, pancreatic, neuroblastoma, and bladder malignancies and association with poor prognosis; (2) the role of Vitamin-D/VDR in increased immune checkpoint inhibitor ligand PD-L1 expression in head-neck cancer, leukemia,^23^ and ovarian cancer cells (unpublished data); and (3) VDR’s role in expression of MYCN,^19^ an oncogene dysregulated in ∼70% of human cancers.^37−45^ Further, RXRα and Importin-4, the critical downstream signaling nodes of VDR, are also shown to be altered in malignant cells and predict poor prognoses in malignancies^16−18^ (Figures S3 and S4).

The anticancer effects of VDR antagonists are not well understood.^22,23,48,49^ Our study shows that ovarian cancer, neuroblastoma, medulloblastoma, and pancreatic cancers respond to **5** treatment *in vitro* and *in vivo* (Figures 4d,f, 5c–h, and 6a,d). *In vivo*, animals treated with **5** did not experience detrimental effects on their weights (Figures 4f and 5d) or general demeanors. **5** actions are VDR-dependent, as stably VDR knockdown SKOV-3 cells show diminished responses compared to stably VDR overexpressor SKOV-3 cell-lines, which respond better than their null vector or wild-type cell counterparts (data not shown). Importantly, **5** reduced VDR expression in the SKOV-3 cell-line-derived xenografts, demonstrating target engagement *in vivo* (Figure S5). Not only xenograft tumors but also syngeneic TH-MYCN murine transgenic spontaneous neuroblastoma that more closely recapitulates human neuroblastoma disease showed reduction in tumor growth (Figure 8). In terms of signaling, **5** reduced the expression of RXRα and Importin-4, the two pivotal nodes of the VDR signaling pathway (Figure 4b,c). The probable mechanism of action of MeTC7 is outlined in Figure 9. Since **5** does not directly inhibit RXRα (Figure 2c), it is likely that RXRα released post VDR inhibition is degraded. Decreased expression of RXRα is therapeutically important because RXRα is prognostic in renal cancer (*p* < 0.00056), melanoma (*p* = 0.034), ovarian cancer (*p* = 0.0017), endometrial cancer (*p* = 0.072), and thyroid cancer (*p* = 0.015) (www.proteinatlas.org). Similarly, **5** inhibits Importin-4 in 2008 ovarian cancer cells. Importin-4 executes nuclear internalization of VDR. VDR and Importin-4 colocalize in ovarian cancer tissues (Figure S4a), and we postulated that targeting the VDR/Importin-4 axis may be crucial to controlling VDR/Importin-4-orchestrated malignancies because, similar to VDR, Importin-4 mRNA expression independently predicts poor prognosis in neuroblastoma (Figure S4b) and hence the need to inhibit it. Further, **5** inhibits MYCN expression in neuroblastoma cells. MYCN expression is altered in over 70% of human cancers^38−45^ and predicts poor prognoses in ovarian cancer (*p* = 0.0033) and neuroblastoma (Figure S7). MYCN-driven cancers are aggressive and chemoresistant and await a targeted therapy.^20−22^ VDR is a key regulator of MYCN expression,^19^ and therefore, targeting the VDR/MYCN axis can be exploited to control such malignancies. For example, **5** treatment blocked the growth of TH-MYCN transgenic neuroblastoma *in vivo* and its spheroids *in vitro*. Next, we examine the effects of **5** on immune cells in TH-MYCN mice as a measure of off-target effects because immune cells express VDR and function calcitriol-dependently. The flow cytometric analysis of the tumors and immune cells isolated from mice carrying TH-MYCN tumors that were treated with vehicle/**5** showed unaltered populations of CD45^+^ CD4^+^, CD8^+^, macrophages, patrolling monocyte, DCs, and CD11b^+^ cells in mice (Figure S8), suggesting that VDR inhibition by **5** spares immune cells, which is notable because chemotherapies often cause indiscriminate cytotoxicity against normal hematopoietic cells, imposing life-threatening side effects.

Finally, notwithstanding the challenges associated with the currently known VDR agonists, the functions of VDR and its agonists remain an ongoing inquiry for cancer treatment. Notably, Sherman et al.^50^ showed that as an adjuvant Vitamin-D reprograms tumor stroma transcriptionally and enables chemotherapeutic responses in pancreatic ductal adenocarcinoma (PDA). Similarly, while the role of Vitamin-D/VDR in upregulation of PD-L1 on ovarian cancer (unpublished data) and leukemia and head and neck cancer cells^23^ is concerning in the context of malignancies, there lies a very promising opportunity to use **1** to convert a cold-tumor type like PDA and ovarian cancer into a PD-L1-enriched hot-tumor type that can be better targeted by immune checkpoint antibodies and/or **5**.

Compound **5** differs significantly from the previously reported VDR antagonists in that it can be synthesized in only two steps from readily available raw materials. This has enabled us to demonstrate the efficacy of VDR antagonists *in vivo*. It is anticipated that the data presented in this study will contribute to the creation of easily synthesized VDR antagonists structurally similar/dissimilar to **5**.

## Experimental Section

### Chemistry

Reagents and solvents were purchased from commercial sources without further purification. The final compounds were purified by preparative thin-layer chromatography (Analtech #Z513059). All compounds were >95% pure by HPLC analysis. The progress of reactions was monitored by thin-layer chromatography (TLC). NMR spectra were obtained from a 400 or 600 MHz Bruker spectrometer. Electrospray ionization mass spectrometry (ESI-MS) was performed on an Agilent 1100 LC-MS spectrometer. Melting points were determined with a Yanagimoto hot-stage melting point apparatus and are uncorrected. Purity of **5** was analyzed by a Dionex UltiMate 3000 LC system.

#### Synthesis of Adduct (**7**)

To a solution of 7DHC (**6**) (Sigma Aldrich, 300 mg, 0.7 mM) in dichloromethane was added *N*-methyl-1,2-4-triazolinedione (87 mg, 0.7 mM) at 0^°^C. During the following 4–5 h, the pink color of the reaction medium was discharged. The separated product (adduct) was filtered, washed with hexane (10 mL × 5), and dried in vacuum in a desiccator overnight. Weight: 205 mg (52%). NMR assignments: ^1^H NMR (600 MHz, DMSO) δ 6.33 (d, *J* = 8.3 Hz, 1H), 6.23 (dd, *J* = 8.3, 0.8 Hz, 1H), 4.63 (s, 1H), 4.04 (dq, *J* = 10.3, 5.3 Hz, 1H), 2.94 (ddd, *J* = 14.1, 5.0, 1.4 Hz, 1H), 2.78 (s, 3H), 2.46–2.38 (m, 1H), 2.08 (dd, *J* = 12.4, 6.5 Hz, 1H), 1.98–1.93 (m, 1H), 1.88–1.79 (m, 2H), 1.67 (dd, *J* = 10.4, 5.6 Hz, 1H), 1.65–1.60 (m, 1H), 1.57–1.50 (m, 2H), 1.53–1.41 (m, 2H), 1.41–1.23 (m, 2H), 1.23–1.06 (m, 6H), 1.05–0.97 (m, 1H), 0.91 (d, *J* = 6.5 Hz, 3H), 0.85 (d, *J* = 6.6 Hz, 3H), 0.84 (d, *J* = 6.6 Hz, 3H), 0.83 (s, 3H), 0.73 (s, 3H). ^13^C NMR (150 MHz, DMSO) δ 150.02, 147.64, 135.60, 128.01, 65.29, 64.84, 64.03, 54.67, 52.37, 49.14, 43.39, 40.28, 38.88, 37.72, 35.37, 34.67, 34.18, 33.61, 29.80, 27.36, 27.12, 24.64, 23.15, 22.63, 22.42, 22.37, 21.71, 18.71, 17.07, 12.59.

#### Synthesis and Characterizations of MeTC7 (**5**)

To a stirred solution of bromoacetic acid (36 mg, 0.13 equivalent) in anhydrous dichloromethane (DCM) maintained in an ice bath was added DCC (87 mg, 0.16 equivalent) and purged with nitrogen. The reaction mixture was stirred for 10 min. To the suspension formed was added adduct (**7**, 100 mg, 0.2 mM) and stirred. A catalytic amount of DMAP was also added and stirred overnight, during which the temperature of the reaction mixture was allowed to rise to room temperature. Dichloromethane was removed using the Buchi rotavapor, and the crude product obtained was purified using a preparative thin-layer chromatography plate. The band containing the product was collected, and the compound was stripped off the silica gel by washing with MeOH/DCM (9:1). The solvent was removed using a rotary evaporator, and the compound (**5**, MeTC7) was collected after drying under a vacuum in a desiccator as an off-white powder (83 mg, 67%) and stored at −20 °C. Purity of **5** was analyzed by a Dionex UltiMate 3000 LC system using Develosil 250 × 4.6 mm 100Diol-5, 5 μm LC column. A binary solvent system with solvent A (0.1% formic acid in water) and solvent B (0.1% formic acid in acetonitrile) was used with a linear gradient of 0% B to 50% B from 0 to 5 min; 50% B to 70% B from 5 to 7 min; isocratic elution of 70% B from 7 to 8 min; linear gradient of 70% B to 80% B from 8 to 10 min; 80% B to 85% B from 10 to 15 min; 85% B to 100% B from 15 to 30 min; 100% B to 50% B from 30 to 36 min at a flow rate of 1 mL/min. NMR assignments. ^1^H NMR (CDCl_3_, 600 MHz) δ 6.35 (d, *J* = 8.3 Hz, 1H), 6.14 (d, *J* = 8.3 Hz, 1H), 5.55 (tt, *J* = 11.0, 5.4 Hz, 1H), 3.82 (s, 2H), 3.2 (ddd, *J* = 13.8, 5.1, 1.4 Hz, 1H), 2.95 (s, 3H), 2.55–2.48 (m, 1H), 2.28–2.22 (m, 1H), 2.21–2.16 (m, 1H), 2.12–1.99 (m, 3H), 1.78–1.64 (m, 4H), 1.57–1.47 (m, 2H), 1.47–1.20 (m, 8H), 1.18–1.08 (m, 3H), 1.07–1.02 (m, 1H), 0.95 (s, 3H), 0.92 (d, *J* = 6.6 Hz, 3H), 0.87 (d, *J* = 6.6 Hz, 3H), 0.86 (d, *J* = 6.6 Hz, 3H), 0.77 (s, 3H). ^13^C NMR (150 MHz, CDCl_3_) δ 166.20, 150.62, 148.11, 134.72, 129.20, 72.59, 64.87, 64.70, 55.04, 52.79, 49.26, 43.95, 40.92, 39.46, 38.16, 35.86, 35.27, 33.55, 30.65, 28.03, 27.51, 26.19, 25.64, 25.05, 23.69, 23.19, 22.81, 22.57, 22.38, 18.94, 17.37, 12.93. HRMS: Calculated for [M + H]^+^: 618.2828, found: 618.2882. Purity of **5** was assessed by both elemental analysis and HPLC. Elemental analysis: calculated for C_32_H_48_BrN_3_O_4_: C, 62.13; H, 7.82; N, 6.79. Found: C, 62.288; H, 7.718; N, 6.712. HPLC: Retention time (RT): 19.62. Purity: 98.2%. Mp 177.1–178.0. ^1^H,^13^C,^1^H–^1^H COrrelated SpectroscopY (COSY), nuclear Overhauser effect spectroscopy (NOESY), multiplicity edited heteronuclear single quantum coherence (HSQC), heteronuclear multiple bond correlation (HMBC), and selective HMBC spectrograms of the adduct and **5** (each in CDCl_3_, at 600 MHz) are provided in the Supporting Information Section (Figure S1).

### Fluorescence Polarization (FP) Assay

The assay^27,28^ was conducted in 384-well black polystyrene microplates (Corning, #3573) using 20 μL of buffer per well (25 mM PIPES, 50 mM NaCl (Fisher), and 0.01% NP-40, at pH 6.75), 0.1 μM VDR-LBD, 17.5 nM Alexa Fluor 647-labeled SRC2-3, and 10 nM, 1,25(OH)_2_D_3_ or 5 μM PPARγ-LBD, Texas Red-labeled DRIP2 (7 nM) and rosiglitazone (1 μM). Then, 10 mM stock solutions of synthesized compounds made in DMSO were serially diluted (1:2) and added with a Tecan Freedom EVO liquid handling system using a 50H hydrophobic-coated pin tool that carried 100 nL (V&P Scientific). After 2 h of incubation, fluorescence polarization was detected at emission/excitation wavelengths of 635/685 nm (Alexa Fluor 647) and 596/615 nm (Texas Red). Three independent experiments were carried out in quadruplicate, and data were analyzed using nonlinear regression with a variable slope (GraphPrism).

### Transcription Assays^27,28^

Human embryonic kidney (HEK) 293T cells were cultured in 75 cm2 flasks (CellStar) coated in matrigel (BD Bioscience, #354234). Cells were grown in Dulbecco’s modified Eagle’s medium (DMEM)/high glucose (Hyclone, #SH3024301) media to which nonessential amino acids (Hyclone, #SH30238.01), 10 mM HEPES (Hyclone, #SH302237.01), 5 × 10^6^ units of penicillin and streptomycin (Hyclone, #SV30010), and 10% of heat-inactivated fetal bovine serum (Gibco, #10082147) were added. For the assay, cells at 70–80% confluency were transfected by lipid-based methods, where 2 mL of untreated DMEM/high glucose media (without additives) containing 0.7 μg of VDR-CMV plasmid, 16 μg of a CYP24A1-luciferase reporter gene, lipofectamine LTX (75 μL, Life Technologies, #15338020), and PLUSTM reagent (25 μL) were added to the flask. After 16 h of incubation at 37 °C with 5% CO_2_, the cells were harvested with 0.05% Trypsin (Hyclone, #SH3023601) and added to sterile white, optical-bottom 384-well plates (NUNC, #142762), plates that were pretreated with a 0.25% matrigel solution. To each well, 20 μL of cells was added to yield a final concentration of 15,000 cells per well. After 4 h, plated cells were treated with compounds in DMSO solution using a Tecan Freedom EVO liquid handling system with a 50H hydrophobic-coated pin tool. In the competitive inhibition assay, 1,25(OH)_2_D_3_ (10 nM) was also added to the assay wells containing **5**. After 16 h of incubation at 37 °C with 5% CO_2_, 20 μL of Bright-Glo Luciferase assay kit (Promega, Madison, WI) was added to each well and the luminescence was read. At least two independent experiments were performed in quadruplicate, and data were analyzed using nonlinear regression with variable slope (GraphPrism).

### Fluorescence Polarization Binding Assay against RXRα

Fluorescence polarization binding assays were done with an INFINITE pro200.^30^ The measurements were performed in 1% DMSO buffer (pH 7.9, 10 mM HEPES, 150 mM NaCl, 2 mM MgCl_2_). To a 384-well plate (Greiner 784076), RXRα-LBD (10 μL, 0.5 μM final concentration), CBTF-BODIPY (5 μL, 0.3 μM final concentration), and **5** (5 μL, 32, 16, 8, 4, 2, 1, 0.5, 0.25, 0.125, 0.0625, 0.03125 μM final concentration) were added, and the plate was incubated at 25 °C for 1 h. The excitation and emission wavelengths were read at 485 nm and 535 nm, respectively. The IC^50^ value of each test compound was calculated using Prism 8.

### Induced-Fit Molecular Docking Methods

The available crystal structure of Vitamin-D receptor (VDR) with **1** (PDB code: 1DB1)^31^ provided the platform for structural modeling and studies. Most of the modeling and simulation were carried out using the modeling suite from Schrödinger 2021.^32^ The crystallographic waters were removed to avoid conformational discrepancies associated with water sampling during the simulations. Overall, the implicit-water model was used during minimization, conformational search, molecular and induced-fit docking, loop refinement, and energetic calculation. A restrained minimization job with an RMSD constraint of 0.3 Å was carried out on the preprocessed structure using the OPLS3e force field to further refine the structure. The starting structures of **1** and **5** were obtained by performing a 5000-step conformational search with 0.05 kJ/mol convergence criteria using the Polak–Ribiere Conjugate Gradient (PRCG) method using the MMFF force field. The chemical structure of **5** is larger in size and conformationally rigid molecule than **1**. To handle this large and rigid molecule, the induced-fit (IF) docking strategy was implemented. Therefore, the IF docking protocol^33,34^ was used to conduct docking of **5** to the **1** site in VDR followed by side-chain refinement through Prime^32^ to allow receptor flexibility according to the binding mode. Overall docking strategies were checked by reference molecule **1**, and several docked poses were generated, and the best receptor-**5** docked complex was selected for the final minimization in using the OPLS3e force field to relax and optimize to reveal the possible binding mode of **5**.

### Cell Lines

SKOV-3 (ATCC, HTB77), OVCAR-3 (ATCC, HTB-161), OVCAR-8 (inherited from Laurent Brad’s previous laboratory), and CAOV-3 (ATCC, HTB75) ovarian cancer cells were grown in complete DMEM media (Gibco, 11965). IGROV-1 (Sigma, SCC203) and 2008 (kindly provided by Dr. François X. Claret, University of Texas M.D. Anderson Cancer Center) ovarian cancer cells were grown in complete RPMI medium (Gibco, 22400). ES2 (ATCC, CRL-1978) was grown in McCoy’s 5A complete medium (ATCC, 30-2007). BE(2)C (ATCC, CRL-2268), SH-EP1 (ATCC, CRL-2269), SH-SY5Y (ATCC, CRL-2266), KELLY (Sigma, 92110411), SK-N-AS (Sigma, 94092302), and LAN-5 (COG, http://www.cogcell.org) neuroblastoma cell-lines were maintained in RPMI1640 media (Gibco, 11875) supplemented with 10% heat-inactivated FBS. TH-MYCN+/+ cells were derived by mechanical dissociation of tumors obtained from TH-MYCN homozygous mice^51−53^ and were maintained in RPMI1640 media (Gibco, 11875) supplemented with 20% heat-inactivated FBS, 10–5 mM 2-mercaptoethanol, 1 mM sodium pyruvate, and 1× nonessential amino acids (Gibco, 11140076).

### Animals

All animal experiments were conducted at the University of Rochester under the approval of the Institutional Animal Care and Use Committee (IACUC). NSG mice, 6–8 weeks old, bred in-house were used in ES2, BE(2)C, and SH-SY5Y xenograft studies. TH-MYCN hemizygous mice (129×1/SvJ-Tg(TH-MYCN)41Waw/Nci)^51−53^ were initially obtained from the NCI Mouse Repository (strain code 01XD2) and maintained in a 129×1/SvJ background through cross-breeding with either wild-type 129×1/SvJ mice obtained from The Jackson Laboratory (stock number 000691) or other TH-MYCN hemizygous mice. TH-MYCN homozygous mice were identified through genotyping as previously described.^51−53^ All mice were maintained on a breeder diet (Labdiet 5021), and tumor-bearing mice were further supplemented with Diet Gel 67A (ClearH2O).

### VDR Expression Analyses

Survival analyses of patients diagnosed with pancreatic, lung, bladder, esophageal, and bladder cancers as well as neuroblastoma and other malignancies (Figure S2) were generated by analyzing the mRNA data available at the R2-Genomics Analysis and Visualization platform (http://hgserver1.amc.nl) or the Human Protein Atlas (https://www.proteinatlas.org/). Best system-recommended cutoffs were opted. Databases analyzed for this study include the following: bladder cancer: Higlund-308-custom-ilmnht12v3; breast cancer: TCGA-1097-rsem-tcgars; cervical cancer: TCGA-305-rsem-tcgars; Glioma: TCGA-540-Mas5.0-u133a; liver cancer: TCGA-371-rsem-tcgars; lung cancer: Bild-114-Mas5.0-u133p2; neuroblastoma: Virsteeg-88-Mas5.0-u133p2; ovarian cancer: Mcdonald-45 (fRMA-u133p2), Wong-77 (fRMA-u133p2), Mechta-Grigoriou-107-Mas5.0-u133p2; and pancreatic cancer: Badea-78(Mas5.0-u133p2), Wang-51 (Mas5.0-u133p2), TCGA-178-rsem-tcgars, and Yeh-132-custom-4hm44k.

### MTS Assay

Viability of ovarian cancer and neuroblastoma cell-lines exposed to **5** treatment was determined by the CellTiter 96 AQueous One Solution assay (Promega Corp, Madison, WI). Cells were seeded into a 96-well plate at 5,000 cells/100 μl/well density in a complete cell culture medium, allowed to attach overnight at 37 °C with 5% CO_2_, in a humidified incubator, and were treated with a complete medium containing the indicated concentration of **5** dissolved in DMSO (Figures 4d and 6a). The final concentration of DMSO did not exceed 0.2% (v/v). At planned hours, existing media were replaced with fresh RPMI media containing the MTS reagent (1:10 dilution) and incubated for 2–4 h. Absorbance was read at 490 nm using the iMark microplate reader (BioRad). Viability of HepG2 and HEK293T cells after **5** treatment was measured by the CellTiter-Glo (Promega) assay (Figure 5a,b). Cells were plated in quadruplicate in 384-well plates and treated with indicated concentrations of **5**, 7DHC (**6**), and 7DHC-adduct (**7**). Cells were incubated for 18 h at 37 °C. CellTiter Glo (Promega) was added. The number of live cells was quantified by luminescence using a Tecan M1000 plate reader. DMSO (negative) was used as the control. Data were analyzed using nonlinear regression with the variable slope (GraphPadPrism) assay.

### Immunohistochemical Analyses

Neuroblastoma tissues were fixed in 10% neutral buffered saline for several days and then dehydrated into paraffin using a Sakura VIP tissue processor and Sakura Tissue Tek 5 embedding center. Sections of 5–10 μm in thickness were cut using a Leica RM2265 microtome. Immunohistochemical stains were performed using the GBI Polink-2 antirabbit HRP Plus Detection System (GBI International, D39) or the Mouse-on-Mouse HRP-Polymer Bundle (BioCare Medical) and were counterstained with hematoxylin. Prior to primary antibody addition, sections were rehydrated, followed by 30 min antigen retrieval in sodium citrate buffer pH 6.0, and blocked of endogenous peroxidase with hydrogen peroxide. Primary antibodies used for immunohistochemistry were mouse rabbit anti-VDR (Abcam, ab3508), mouse anti-VDR (Santa Cruz Biotechnology, SC-13133), mouse anti-MYCN (Santa Cruz Biotechnology, SC-53993), rabbit anti-tyrosine hydroxylase (TH) (Millipore, AB152), normal rabbit IgG (Millipore, 12-370), and normal mouse IgG (Millipore, 12-371). Slides were visualized using an Olympus BX41 light microscope and imaged with an Olympus DP70 camera. Photographs were captured using CellSens digital software.

### Confocal Microscopy

Briefly, sixteen-bit images were acquired with a Nikon E800 microscope (Nikon Inc., Melville, NY) using a 40× PlanApo objective. A Spot II digital camera (Diagnostic Instruments, Sterling Heights MI) was used to acquire the images. The camera’s built-in green filter was used to increase the image contrast. Camera settings were based on the brightest slide. Images were acquired with the same settings. Image processing and analysis were performed using iVision (BioVision Technologies, version 10.4.11, Exton, PA.) image analysis software. Positive staining was defined through intensity thresholding, and integrated optical density (IOD) was calculated by examining the thresholded area multiplied by the mean. All measurements were performed in pixels. Confocal images were acquired with a Nikon C1si confocal (Nikon Inc., Melville, NY.) using diode lasers 402, 488, and 561. Serial optical sections were performed with EZ-C1 computer software (Nikon Inc., Melville, NY). Z series sections were collected at 0.3 μm with a 40× PlanApo lens and a scan zoom of 2. The gain settings were based on the brightest slide and kept constant between specimens. Deconvolution and projections were done in Elements (Nikon Inc. Melville, NY) computer software.

### Xenograft Animal Models

ES2, SH-SY5Y, and BE(2)C cells isolated from 70 to 80% confluent Petri dishes were spun down (1000 rpm, 5 min). Media was removed, and cells (calculated 250,000/mice for ES2 and 1 million/mice for SH-SY5Y and BE(2)C) were suspended in cold matrigel/serum-free RPMI media mix (1:1) and implanted subcutaneously in the right flank of the NSG mice. Prior to inoculation, NSG mice were shaved at the inoculation site using a clean shaving machine, and skin was disinfected and cleaned using commercially available alcohol swabs. SKOV-3, D283, and PANC-1 cells were grown to semiconfluence in complete DMEM media. BXPC-3 cells were cultured in complete RPMI media to 70–80% confluence. Trypsinized cells were harvested, centrifuged, and suspended in precooled matrigel/DMEM media (1:1) and subcutaneously implanted in nude mice (SKOV-3) or NSG mice (D283, PANC-1, BXPC-3) each at 1 million cells/animal rate. Once tumors became palpable, mice were treated with vehicle or **5**. Tumors in each case were allowed to grow until the volume [(length × width^2^)/0.5] in one or more mice reached 2000 mm^3^, and then the entire group of animals was sacrificed. Tumor sizes and animal weights were recorded periodically except in BE(2)C and ES2 animals, which necessitated alternate day monitoring due to rapid tumor growth. Tumors from the control and drug groups were harvested, weighed, snap-frozen, and stored in liquid nitrogen.

### Spontaneous TH-MYCN Transgenic Neuroblastoma Model

TH-MYCN hemizygous mice (129×1/SvJ-Tg(TH-MYCN)41Waw/Nci) were initially obtained from the NCI Mouse Repository (strain code 01XD2) and maintained in a 129×1/SvJ background though cross-breeding with either wild-type 129×1/SvJ mice obtained from The Jackson Laboratory (stock number 000691) or other TH-MYCN hemizygous mice. TH-MYCN homozygous mice were identified through genotyping as previously described.^52,53^ All mice were maintained on a breeder diet (Labdiet 5021), and tumor-bearing mice were further supplemented with Diet Gel 67A (ClearH2O). Control mice (*n* = 7) and **5** (10 mg/kg, *n* = 8) mice were treated intraperitoneally with indicated doses. Mice in the control and **5** (10 mg/kg) group received six treatments in total, whereas the mice in the **5** (100 mg/kg) group were given just three treatments to see the effect of escalated drug dose on the safety of animals at 10× dose and to monitor for changes in the tumor burden. The tumor burden in each mouse was estimated using ultrasound imaging instrumentation as described below.

### Ultrasound Imaging of TH-MYCN Mice

Tumors in vehicle/drug-treated groups were visualized by abdominal ultrasound using a Vevo 3100 Imaging System and MX550D transducer (FUJIFILM VisualSonics, Inc). Animals were anesthetized (1–3% isoflurane and oxygen mixture) and restrained on a heated stage with monitors for respiration and heartbeat. Ventral hair was removed with a depilatory cream prior to monitoring with an ultrasound probe. The 3D volume measurements were carried out using Amira 6.1 software with an XImagePAC extension (FEI).

### Statistical Analyses

To analyze the statistical difference between vehicle and **5**-treated ES2 xenograft tumors (Figure 4f), a repeated-measures analysis of variance was performed using maximum likelihood estimation with group, day, and the interaction between group and day as fixed effects. The correlation of repeated measures on the same subject over time was handled using an unstructured covariance, which was allowed to vary by treatment condition. Model assumptions were verified graphically. Analysis was conducted using SAS v9.4 Proc Mixed (Cary, NC). Assumptions made by the mixed model analysis were verified by examining the distribution of residuals, or unexplained variation. Ideally, the residuals are approximately normally distributed with a mean of zero and no obvious patterns. Finally, we used a nonparametric test (Wilcoxon rank sum test) to compare the tumor volumes between groups at each time point. Results corroborated those seen with the regression model, which makes more assumptions. The statistical differences between vehicle and **5**-treated SKOV-3 xenograft tumors, average animal weights, and tumor sizes were compared between the control and **5** groups at the baseline by Student’s *T*-test. Weights and tumor sizes were compared by group over the observation period using linear mixed effect regression. Random intercepts and slopes were included to model within-animal response trajectories. Group differences in the rate of weight change or tumor growth were tested by an interaction term between the treatment group and day of treatment. Residuals were examined to assess model fit. Animal survival was plotted using the Kaplan–Meier method. Two-tailed p-values less than 0.05 were considered statistically significant (Figure 5c–e). The difference between the % change in tumor volumes of D283 medulloblastoma and pancreatic cancer (PANC-1, BXPC-3) and neuroblastoma BE(2)C and SH-SY5Y xenografts treated with control or **5** treatment was analyzed by Student’s *T*-test (Figure 5f–h). Tumor weights in BE(2)C and SH-SY5Y xenografts were compared by Student’s *T*-test. TH-MYCN tumor sizes and weights in the control and treatment groups were compared by Student’s *T*-test (Figure 8b,c).

## Supporting Information Available

NMR data (1H–1H COSY, NOESY, multiplicity edited HSQC, HMBC and selective HMBC) characterization of MeTC7 (**5**) (Figure S1); Kaplan–Meier survival analyses of VDR mRNA and its association with survival among the patients diagnosed with lung cancer, pancreatic cancer, neuroblastoma, breast cancer, glioma, cervical cancer, liver cancer, ovarian cancer, and bladder cancer (Figure S2); Kaplan–Meier survival analyses of RXRα mRNA and its association with survival among patients diagnosed with ovarian cancer (Figure S3); VDR and Importin-4 show colocalization in ovarian cancer tissues and Kaplan–Meier survival analyses of Importin-4 mRNA and its association with survival in patients diagnosed with neuroblastoma (Figure S4); immunohistochemical analysis of xenograft tumors show that MeTC7 (**5**) treatment downregulated VDR in ovarian cancer *in vivo* (Figure S5); MeTC7 (**5**) treatment reduced the growth rate of BE(2)-C xenograft tumors growing in NSG mice (Figure S6); MYCN mRNA overexpression and its poor survival among patients diagnosed with ovarian cancer and neuroblastoma (Figure S7); effects of MeTC7 (**5**) treatment on immune cell markers of spontaneous TH-MYCN tumors treated with vehicle or 5 (Figure S8) (PDF)
Calcitriol (**1**) binding model in VDR-LBD (Movie S1) (MP4)
MeTC7 (**5**) binding model in VDR-LBD (Movie S2) (MP4)
Complies with format V.3.0 (PDF)
Structure of 5 in complex with VDR-LBD by Induced-Fit Docking **SMILES** codes for 4 and 5 (CSV)

## Abbreviations

7DHC: 7-dehydrocholesterol
Arg: arginine
ATCC: American Type Cell Culture
ARG: arginine
COSY NMR: 1H–1H COSY (COrrelated SpectroscopY)
DCC: *N*,*N*′-dicyclohexylcarbodiimide
DCM: dichloromethane
FP: fluorescence polarization
HIS: histidine
HMBC: heteronuclear multiple bond correlation
HSQC: heteronuclear single quantum coherence
LBD: ligand binding domain
mRNA: messenger ribonucleic acid
MTAD: *N*-methyl-1,2-4-triazolinedione
MYCN: v-myc myelocytomatosis viral-related oncogene, neuroblastoma derived
nM: nano molar
NOESY: nuclear Overhauser effect spectroscopy
NR: nuclear receptor
PDA: pancreatic ductal adenocarcinoma
PPARγ: peroxisome proliferator-activated receptor γ
PTAD: 4-phenyl-1,2,4-triazole-3,5-dione
RXRα: retinoic X-receptor α
Ser: serine
SRC: proto-oncogene tyrosine-protein kinase Src
TCGA: The Cancer Genome Atlas
TH-MYCN: tyrosine hydroxylase v-myc myelocytomatosis viral-related oncogene, neuroblastoma-derived
Trp: tryptophane
Tyr: tyrosine
VDR: Vitamin-D receptor
