# Supplementary material for: Identification of a Vitamin-D Receptor Antagonist, MeTC7, which Inhibits the Growth of Xenograft and Transgenic Tumors In Vivo
Source: J Med Chem. 2022 Apr 11;65(8):6039–55. doi: 10.1021/acs.jmedchem.1c01878 (PMC9059124; doi:10.1021/acs.jmedchem.1c01878)
Supplement: Supplementary file 1 — jm1c01878_si_001.pdf [file jm1c01878_si_001.pdf]

Supplementary Information

Identification of a Vitamin-D receptor antagonist, MeTC7, which inhibits the growth of xenograft and transgenic tumors *in vivo*

Negar Khazan[1], Kyu Kwang Kim[1], Jeanne N. Hansen[2], Niloy A. Singh[1], Taylor Moore[1], Cameron W. A. Snyder[1], Ravina Pandita[1], Myla Strawderman[3], Michiko Fujihara[4], Yuta Takamura[4], Ye Jian[5], Nicholas Battaglia[5], Naohiro Yano[6], Yuki Teramoto[7], Leggy A. Arnold[8], Russell Hopson[9], Keshav Kishor[10], Sneha Nayak[10], Debasmita Ojha[10], Ashoke Sharon[10], John M. Ashton[11], Jian Wang[12], Michael T. Milano[13], Hiroshi Miyamoto[7], David C. Linehan[5], Scott A. Gerber[5,13], Nada Kawar[14], Ajay P. Singh[15], Erdem D. Tabdanov[16]*, Nikolay V. Dokholyan[12], Hiroki Kakuta[4]*, Peter Jurutka[17], Nina F. Schor[18]*, Rachael B. Rowswell-Turner [1], Rakesh K. Singh[1]*, Richard G. Moore[1]

[1]Wilmot Cancer Institute and Division of Gynecologic Oncology, Department of Obstetrics and Gynecology, University of Rochester Medical Center, Rochester, NY14624, United States.

[2]Department of Pediatrics, University of Rochester Medical Center, Rochester, NY, USA; Current address: Department of Biology, Colgate University, Hamilton, NY 14624, United States.

[3]Department of Biostatistics and Computational Biology, University of Rochester Medical Center, Rochester, NY 14624, United States.

[4]Division of Pharmaceutical Sciences, Okayama University Graduate School of Medicine, Dentistry and Pharmaceutical Sciences, Kita-ku, Okayama 700-8530, Japan.

[5]Division of Surgery and of Microbiology and Immunology, University of Rochester Medical Center, Rochester NY, 14624, United States.

[6]Alpert Medical School of Brown University, Department of Surgery, Division of Surgical Research, Rhode Island Hospital, Providence, RI 02903, United States.

[7]Department of Pathology and Laboratory Medicine, University of Rochester Medical Center, Rochester, NY 14624, United States.

[8]Department of Chemistry and Biochemistry, University of Wisconsin Milwaukee, Milwaukee, WI 53211, United States.

[9]Department of Chemistry, Brown University, Providence, RI 02912, United States.

[10]Department of Chemistry, Birla Institute of Technology, Mesra, Ranchi 835215, India.

[11]Genomics Core facility, Wilmot Cancer Center, University of Rochester Medical Center, Rochester, NY 14624, United States.

[12]Department of Pharmacology and Department of Biochemistry and Molecular Biology, Penn State College of Medicine, Penn State University, PA 17036, United States.

[13]Department of Radiation Oncology, University of Rochester Medical Center, Rochester, NY 16424, United States.

[14]Center for Breast Health and Gynecologic Oncology, Mercy Medical Center, 271 Carew Street Springfield, MA 01104, United States.

[15]Rutgers, The State University of New Jersey, 59 Dudley Road, New Brunswick, NJ 08019, United States.

[16]CytoMechanobiology Laboratory, Department of Pharmacology, Penn State College of Medicine, Pennsylvania State University, Hershey, PA 17036, United States.

[17]School of Mathematical and Natural Sciences, Arizona State University, Health Futures Center, Phoenix, AZ 85054; University of Arizona College of Medicine, Phoenix, Arizona 85004, United States.

[18]Departments of Pediatrics, Neurology, and Neuroscience, University of Rochester Medical Center, Rochester, NY, 14642; Current address: NINDS, National Institute of Health, Bethesda, MD 20824, United States.

Corresponding Authors:

*(Lead contact): Rakesh K. Singh (Ph.D. MBA); E-mail: rakesh_singh@urmc.rochester.edu; Tel: 585-276-6281.
*Erdem D. Tabdanov (Ph.D.); E-mail: ekt5171@psu.edu; Tel: 717-531-0003, Ext: 4430.
*Hiroki Kakuta (Ph.D.); E-mail: kakuta-h@okayama-u.ac.jp; Tel: +81-(0)86-251-7963.
*Nina Schor (M.D., Ph.D.; Email: nina.schor@nih.gov; Tel: 301-496-9746.

Contents:

Supplementary Figure 1. NMR data (1H-1H COSY, NOESY, Multiplicity Edited HSQC, HMBC and Selective HMBC) characterization of MeTC7 (**5**). p. 3

Supplementary Figure 2. Kaplan Meier survival analyses of VDR mRNA and its association with survival among the patients diagnosed with lung cancer, pancreatic cancer, neuroblastoma, breast cancer, glioma, cervical cancer, liver cancer, ovarian cancer, and bladder cancer. p. 8

Supplementary Figure 3. Kaplan Meier survival analyses of RXRα mRNA and its association with survival among the patients diagnosed with ovarian cancer. p. 10

Supplementary Figure 4. (a) VDR and Importin-4 show co-localization in ovarian cancer tissues. (b) Kaplan Meier survival analyses of Importin-4 mRNA and its association with survival in the patients diagnosed with neuroblastoma. p. 11

Supplementary Figure 5. Immunohistochemical analysis of the xenograft tumors show that MeTC7 (**5**) treatment downregulated VDR in ovarian cancer in vivo. p. 12

Supplementary Figure 6. MeTC7 (**5**) treatment reduced the growth rate of BE(2)-C xenograft tumors growing in NSG mice. p. 13

Supplementary Figure 7. Kaplan Meier survival analyses of MYCN mRNA and its association with survival among the patients diagnosed with ovarian cancer and neuroblastoma. p. 14

Supplementary Figure 8. Effects of MeTC7 (**5**) treatment on the immune cell markers of the spontaneous TH-MYCN tumor treated with vehicle or **5**. p. 15

Supplementary Figure 1.

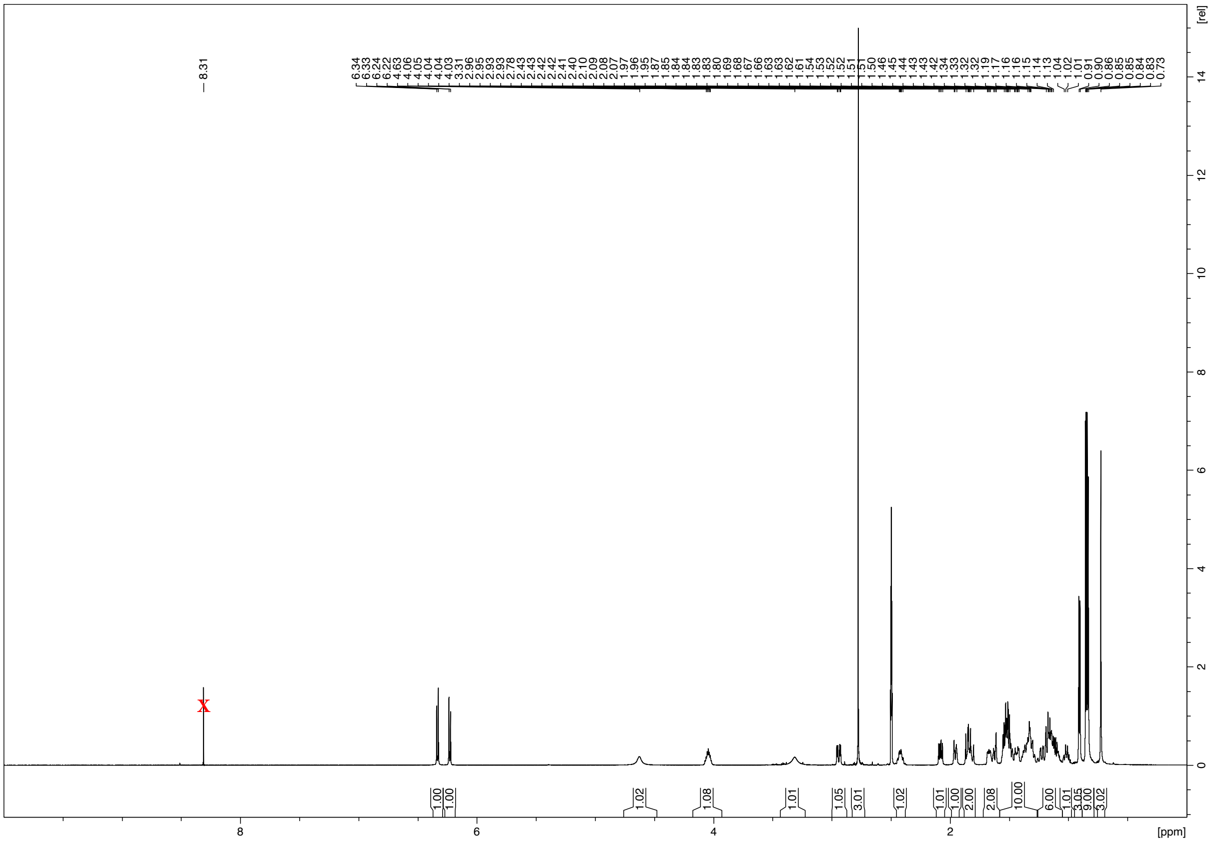

Supplementary Figure 1a: ^{1}H NMR ($CDCl_3$, 600 MHz) spectrum of **7**. X denotes chloroform.

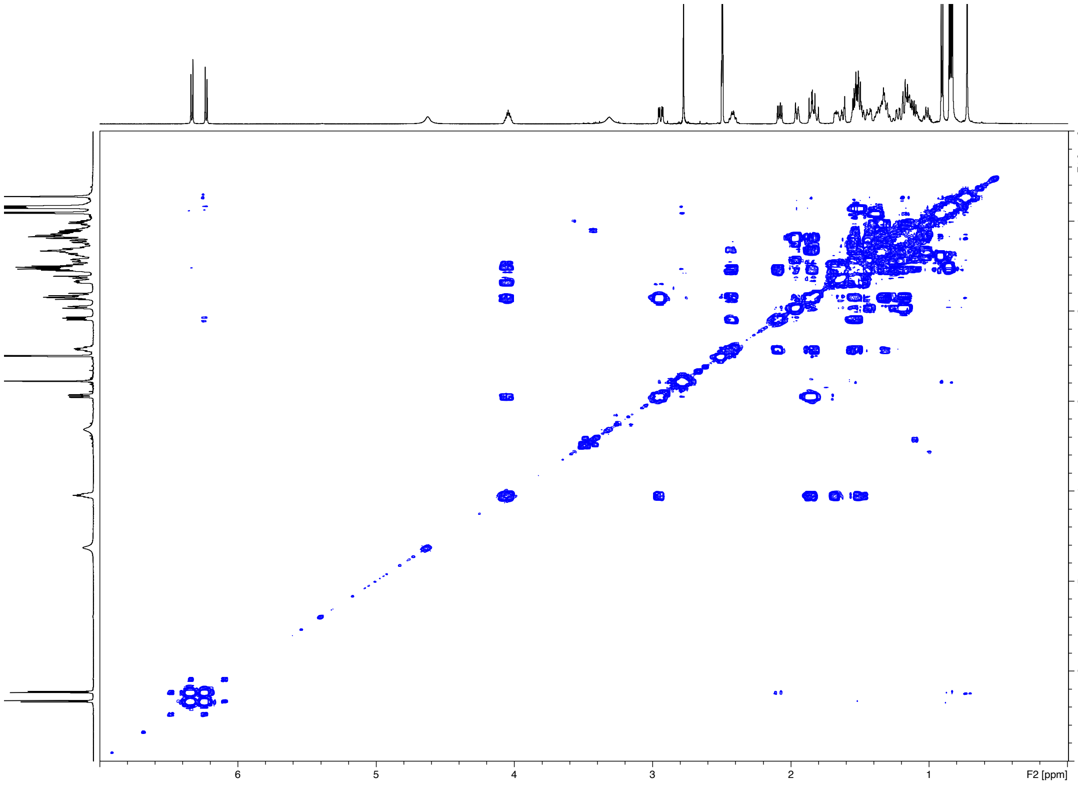

Supplementary Figure 1b: ^{1}H-^{1}H COSY NMR ($CDCl_3$, 600 MHz) spectrum of **7**.

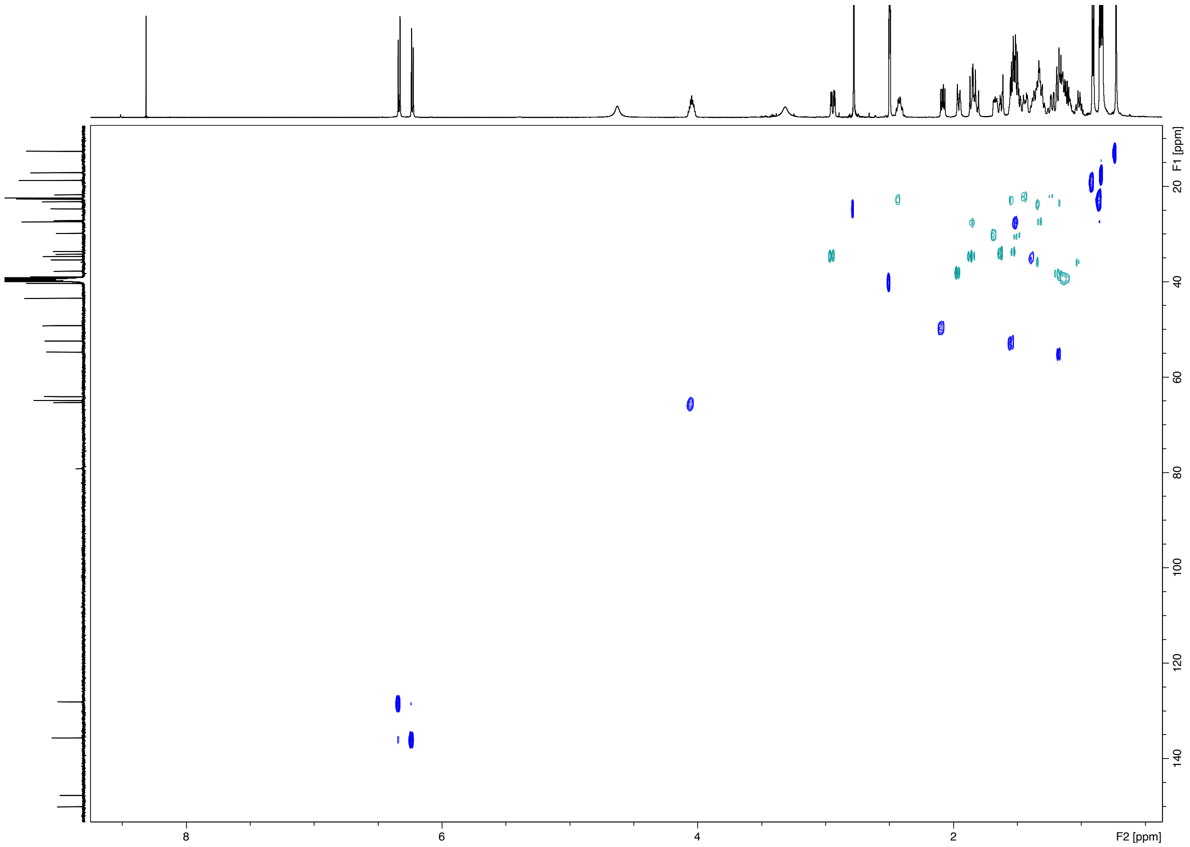

Supplementary Figure 1c. Multiplicity Edited HSQC ^{1}H-^{13}C (600 MHz, 150 MHz) spectrum of **7**.

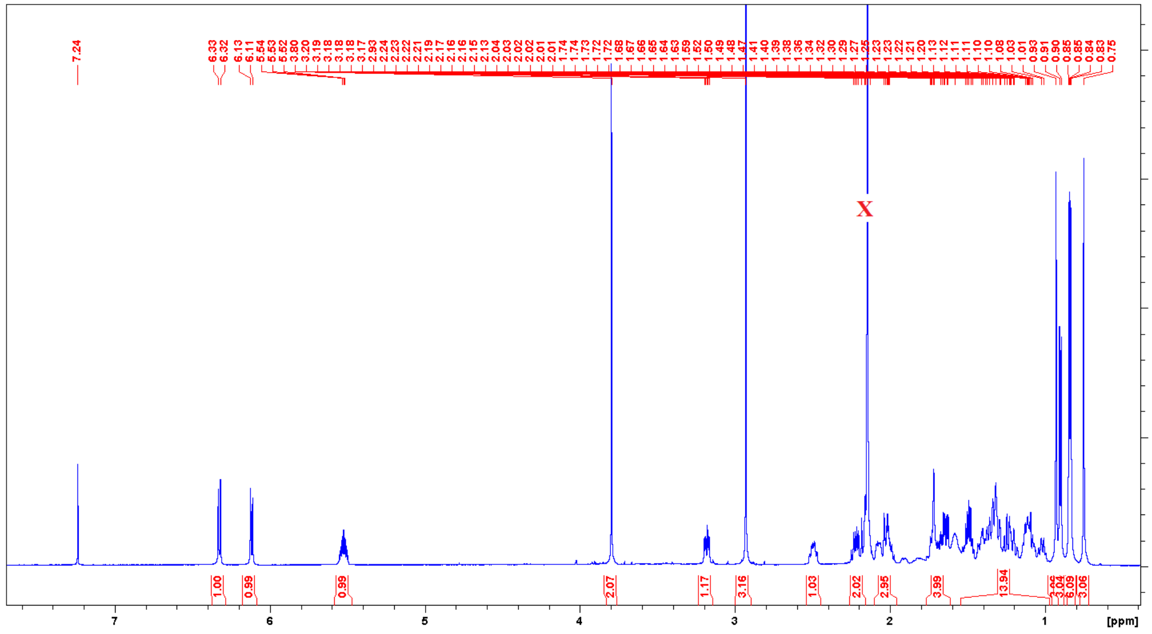

Supplementary Figure 1d. Multiplicity Edited HSQC ^{1}H-^{13}C (600 MHz, 150 MHz) spectrum of MeTC7 (**5**).

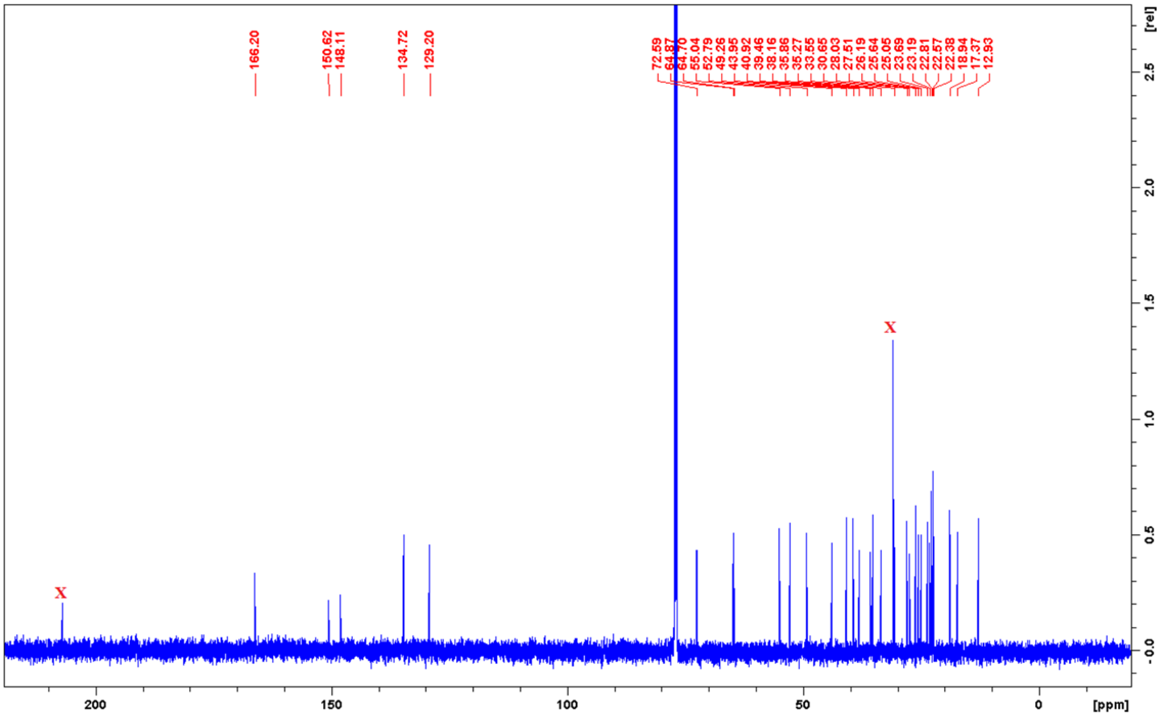

Supplementary Figure 1e. ^{13}C NMR ($CDCl_3$, 150 MHz) spectrum of MeTC7(**5**). X denotes chloroform.

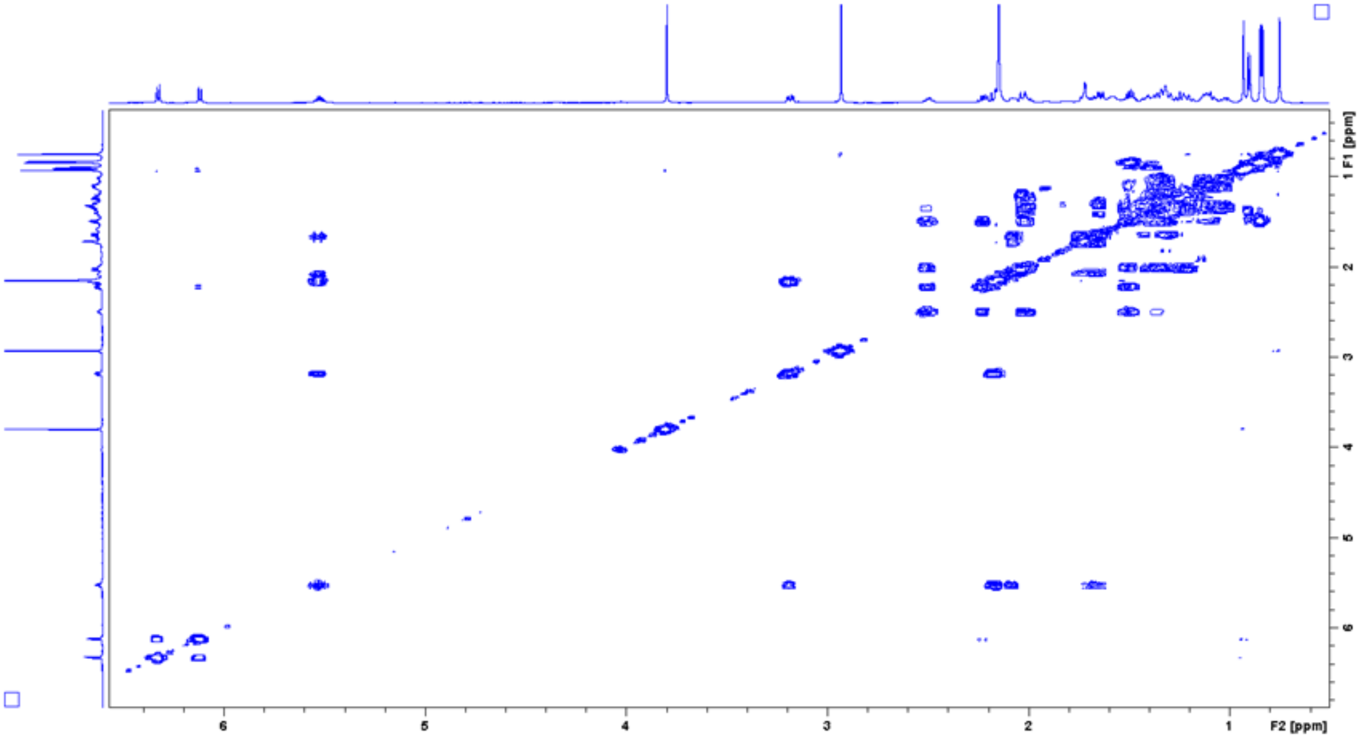

Supplementary Figure 1f. ^{1}H-^{1}H COSY NMR ($CDCl_3$, 600 MHz) spectrum of MeTC7(**5**).

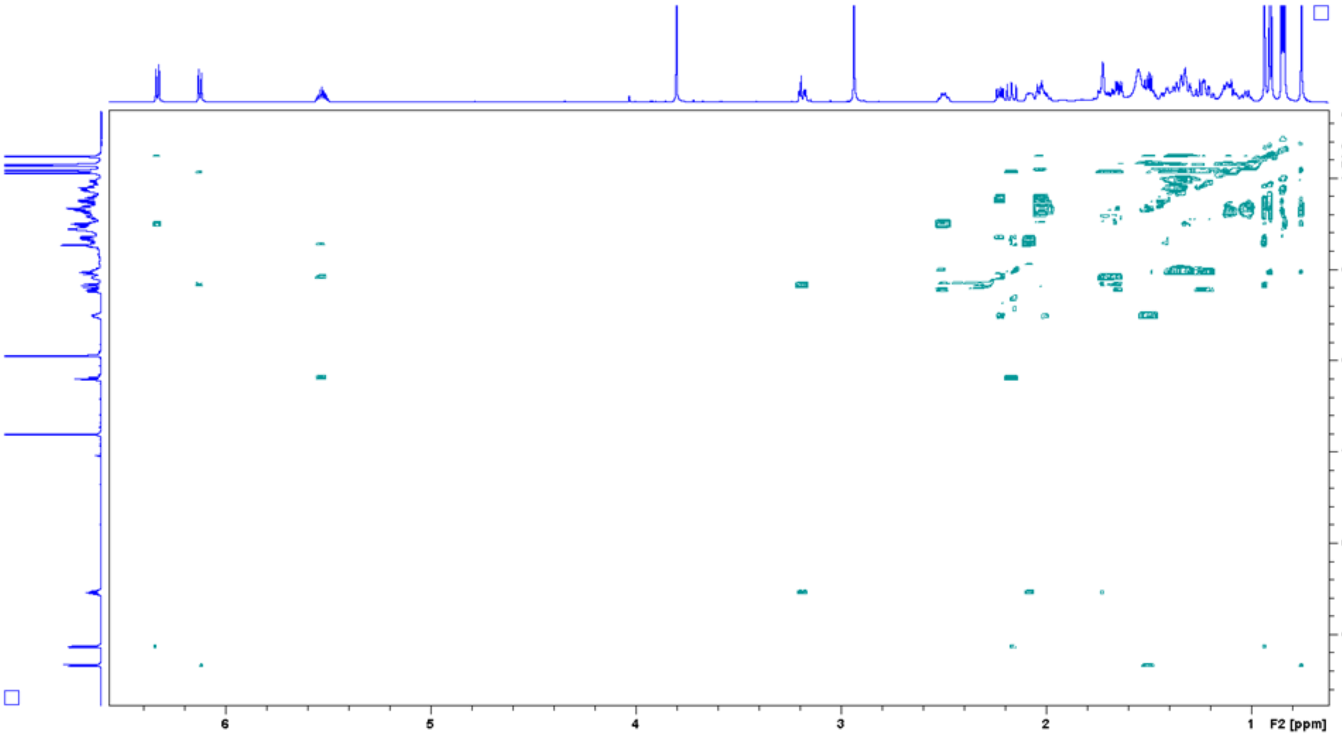

Supplementary Figure 1g. NOESY NMR ($CDCl_3$, 600 MHz) spectrum of MeTC7(**5**). Mixing time = 330 ms.

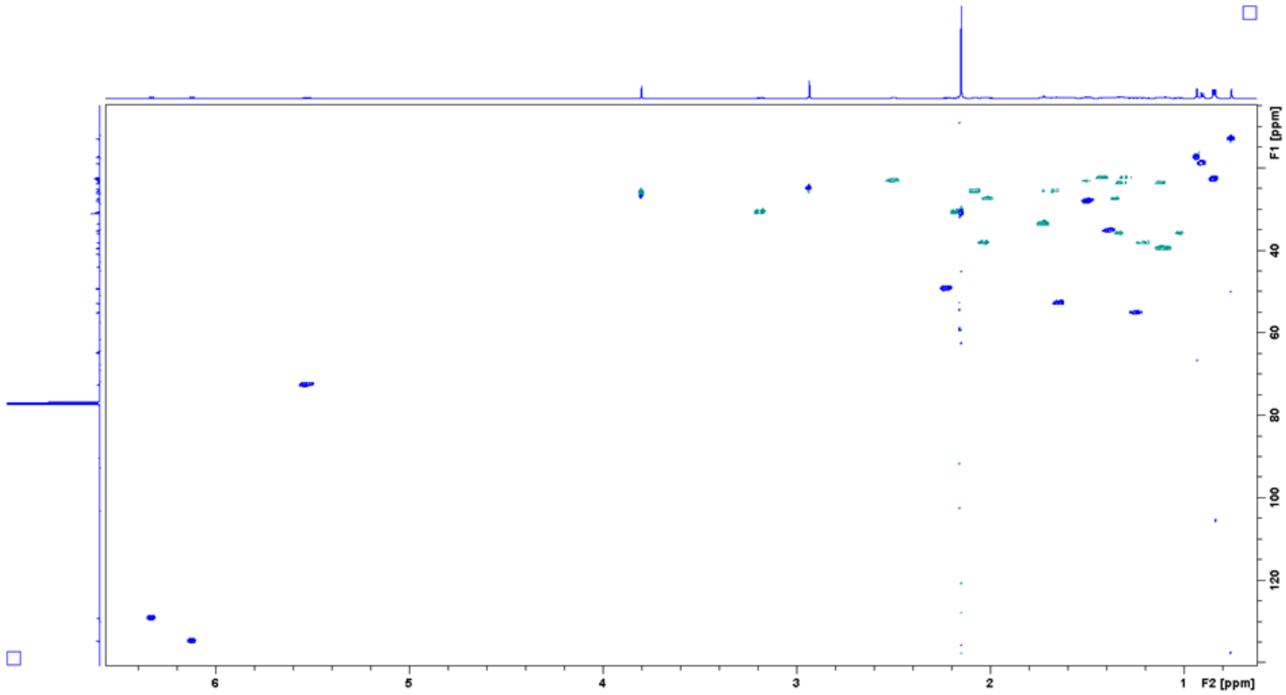

Supplementary Figure 1h. Multiplicity Edited HSQC (600 MHz, 150 MHz) spectrum of MeTC7(**5**).

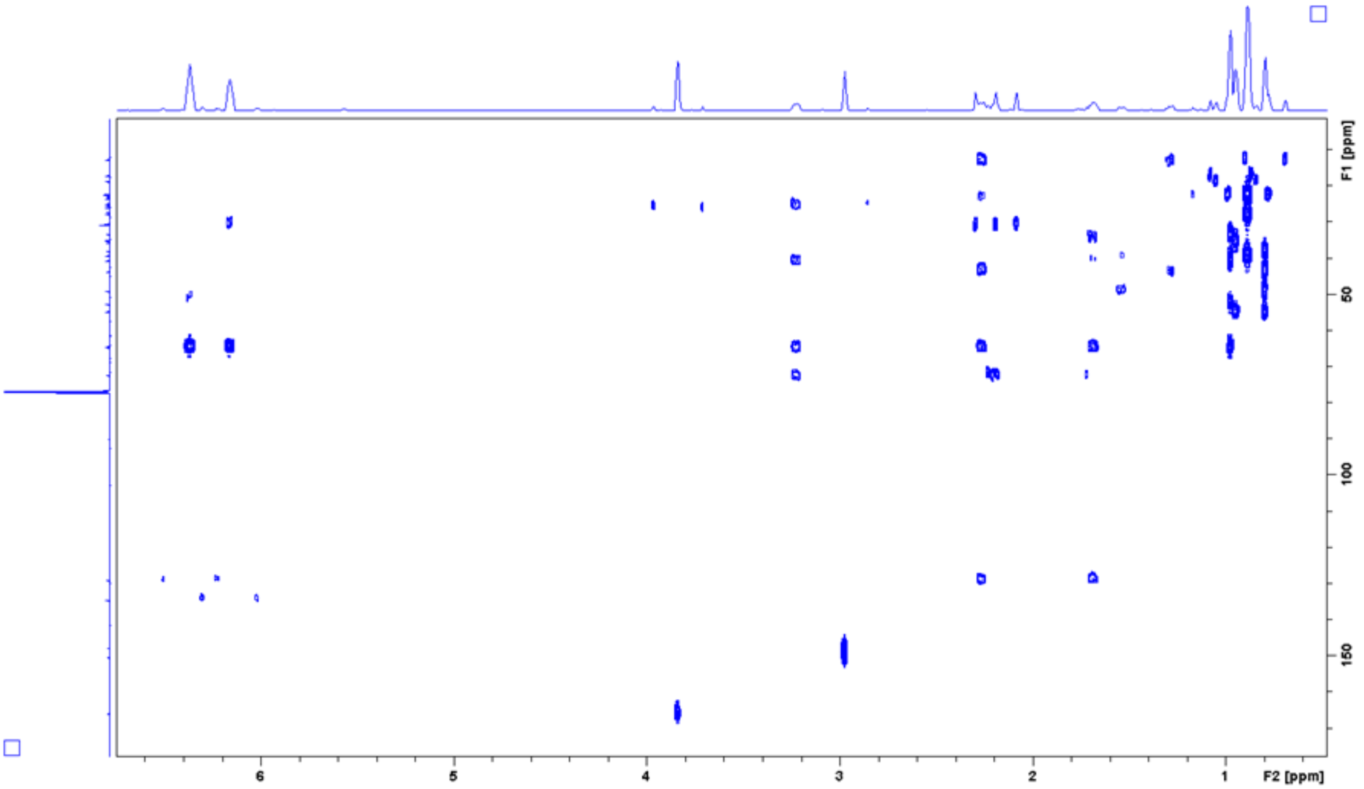

Supplementary Figure 1i. HMBC (600 MHz, 150 MHz) spectrum of MeTC7(**5**).

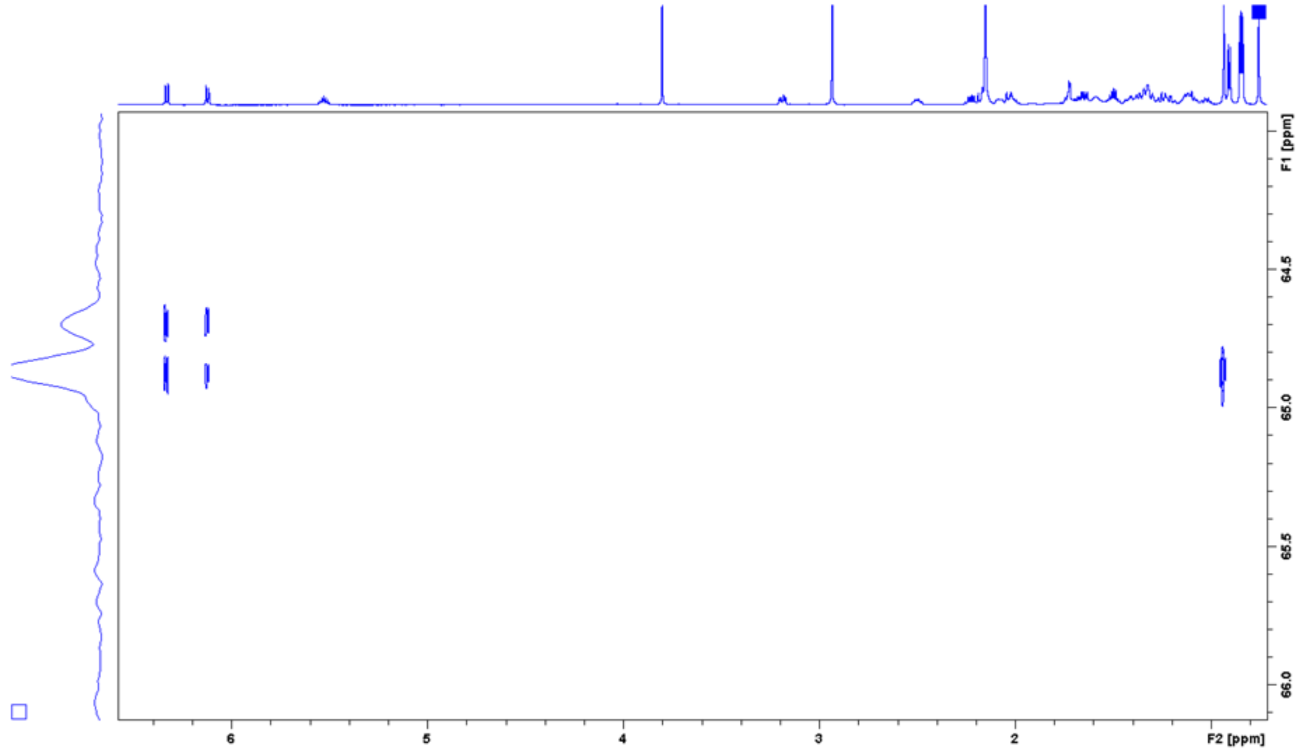

Supplementary Figure 1j. Selective HMBC (600 MHz, 150 MHz) spectrum of MeTC7(**5**).

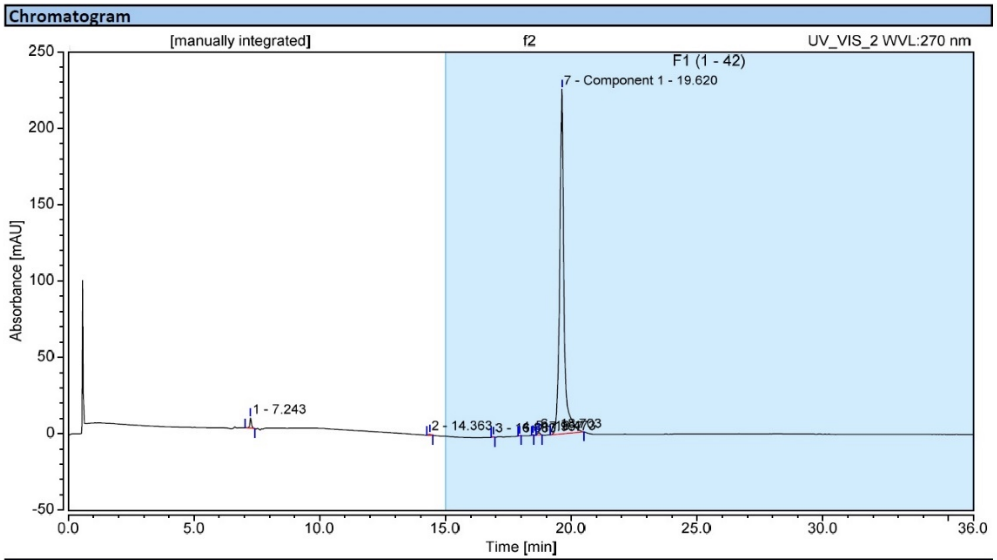

Integration Results

No.	Peak Name	Retention Time min	Area mAU*min	Height mAU	Relative Area %	Relative Height %	Amount
1		7.243	0.478	6.617	1.06	2.81	n.a.
2		14.363	0.068	0.602	0.15	0.26	n.a.
3		16.883	0.033	0.409	0.07	0.17	n.a.
4		17.957	0.019	0.243	0.04	0.10	n.a.
5		18.473	0.020	0.295	0.04	0.13	n.a.
6		18.703	0.190	1.726	0.42	0.73	n.a.
7	Component 1	19.620	44.374	225.634	98.21	95.80	n.a.
**Total:**			**45.182**	**235.527**	**100.00**	**100.00**	

Supplementary Figure 1k. HPLC trace of MeTC7(**5**). HPLC was performed by a Dionex UltiMate® 3000 LC system using Develosil® 250 x 4.6 mm 100Diol-5, 5 μm LC column. A binary solvent system with solvent A (0.1% formic acid in water) and solvent B (0.1% formic acid in acetonitrile) was used with linear gradient of 0% B to 50% B from 0–5 min; 50% B to 70% B from 5–7 min; isocratic elution of 70% B from 7–8 min; linear gradient of 70% B to 80% B from 8–10 min; 80% B to 85% B from 10–15 min; 85% B to 100% B from 15–30 min; 100% B to 50% B from 30–36 min at a flow rate of 1 mL/min.

Supplementary Figure 2.

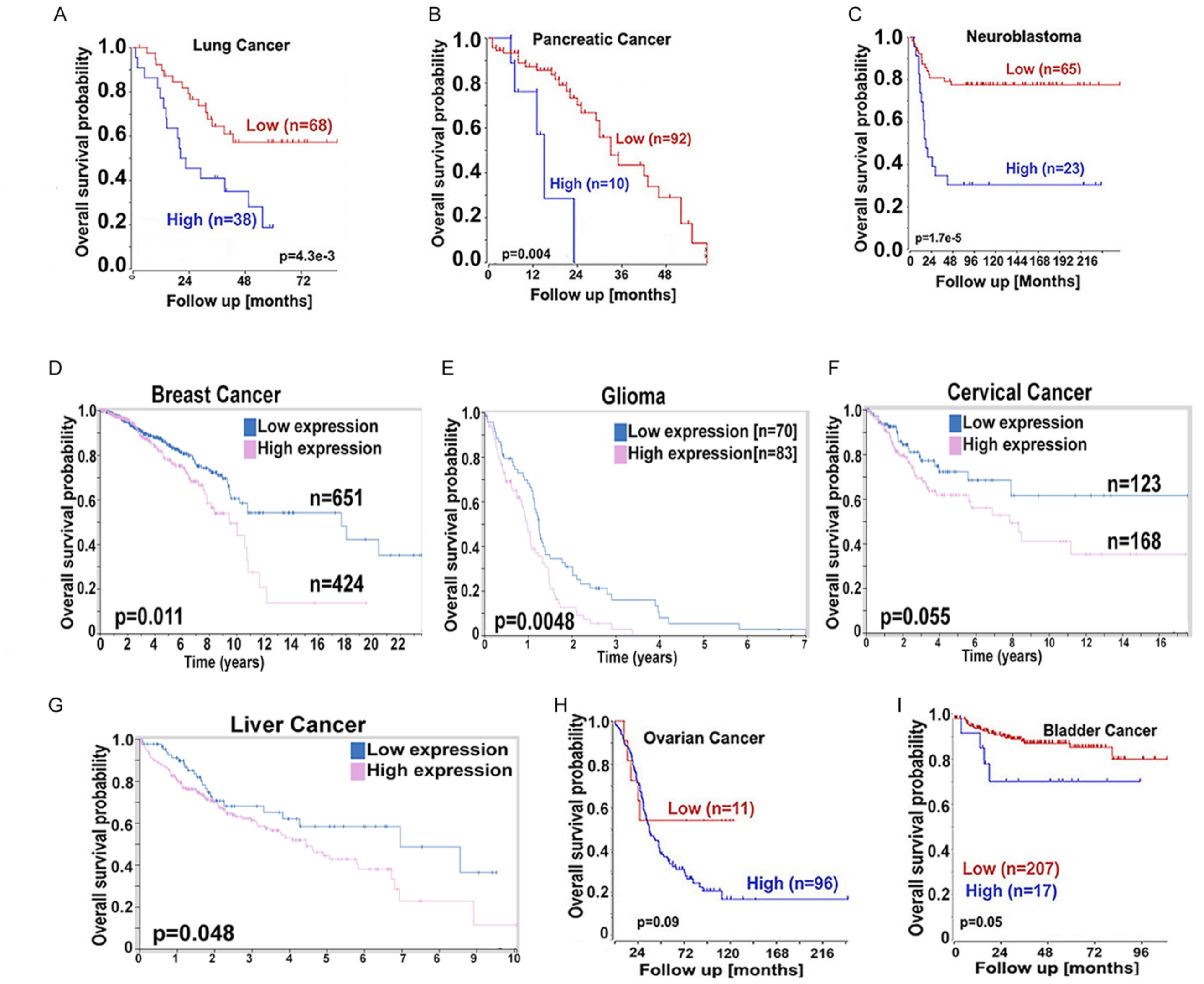

Supplementary Figure 2. Kaplan Meier survival analyses of mRNA in the tumors from the patients diagnosed with lung cancer (A), pancreatic cancer (B), neuroblastoma (C), breast cancer (D), glioma (E), cervical cancer (F), liver cancer (G), ovarian cancer (H), and bladder cancer (I) show poor prognosis associated with VDR mRNA overexpression at the system generated best expression cut-offs parameters. The mRNA data deposited at R2:Genomics Analysis and Visualization Platform (https://hgserver1.amc.nl/cgi-bin/r2/main.cgi) and the Human Protein Atlas (https://www.proteinatlas.org/) were analyzed. The system selected expression cut-offs were selected.

Supplementary Figure 3.

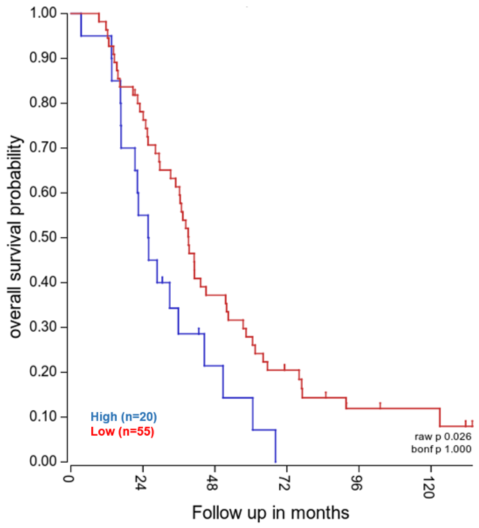

Supplementary Figure 3. Analysis of the ovarian cancer mRNA data (p=0.026, database: Pamula-Pilat-101-MAS5.0, cutoff: 397.9, R2-Genomics and Visualization Platform) showed that RXRα overexpression indicated poor prognosis at the platform suggested cutoff expression parameters.

Supplementary Figure 4.

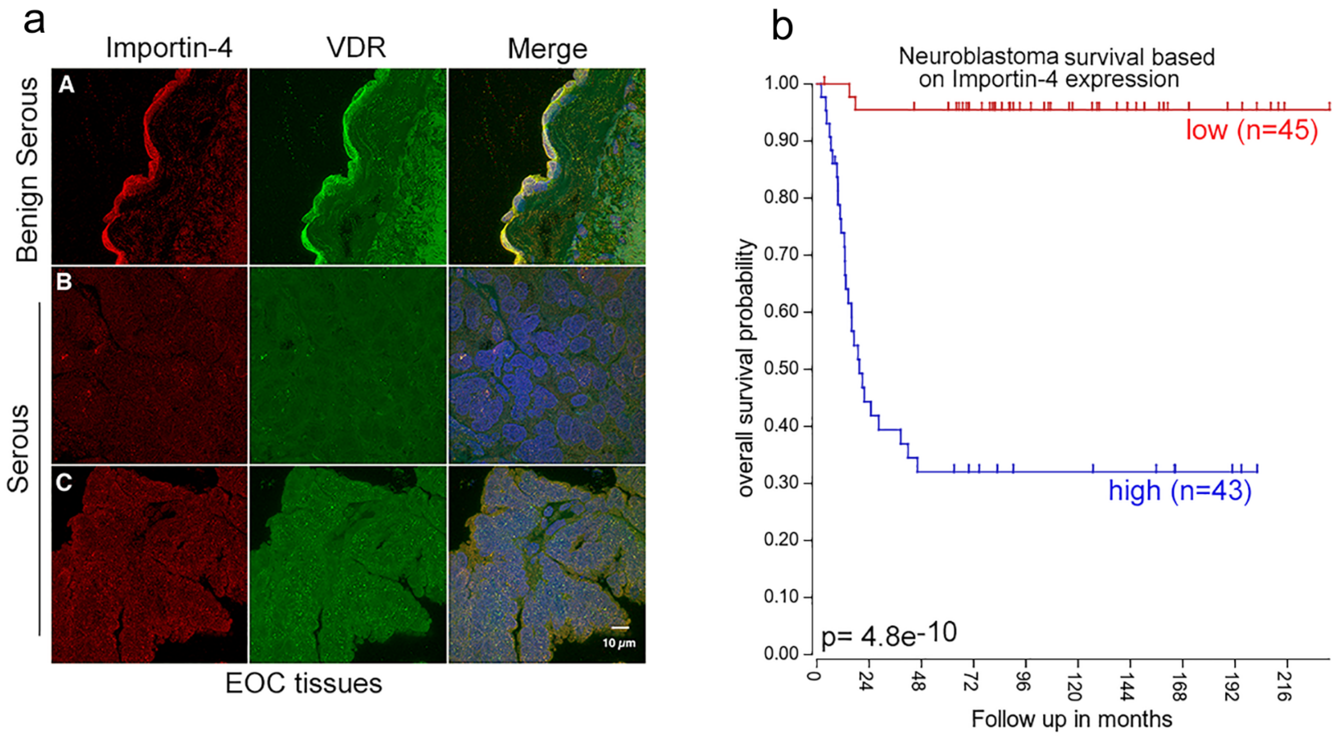

Supplementary Figure 4. (a) VDR and Importin-4 co-localize in ovarian cancer tissues. Ovarian cancer tissues obtained with IRB approval at Women and Infants Hospital of Rhode Island were processed and stained with VDR (Santa Cruz Biotechnology, catalog number: SC13133, dilution 1:100). Slides were washed with PBST and stained with DyLight594 (Vector Laboratories, catalog number: DI-2954). Slides were incubated for 1 hour, washed again with PBST in a light protected container and stained with Importin-4 antibody (Origene, catalog number: TA319673) overnight. Slides were washed again with PBST and stained with DyLight488 (Vector laboratories, catalog number: DI-1488) for one hour. Slides were repeatedly washed with PBST in a light protected container. Vectashiled mounting medium with DAPI (Vector laboratories, catalog number H-1200) was applied on slides and covered with glass coverslips and stored in 4°C in dark till examined under microscope. Co-localizations between VDR and Importin-4 were examined by confocal microscopy as described in the Material and Methods section. (**b**) Analysis of the neuroblastoma mRNA data (database: Verseteeg-88-MAS5.0-U133p2, expression cut 56.6, p= 4.8e-10, available at R2-Genomics and Visualization Platform) showed that Importin-4 (IPO4) overexpression strongly indicated poor prognosis.

Supplementary Figure 5.

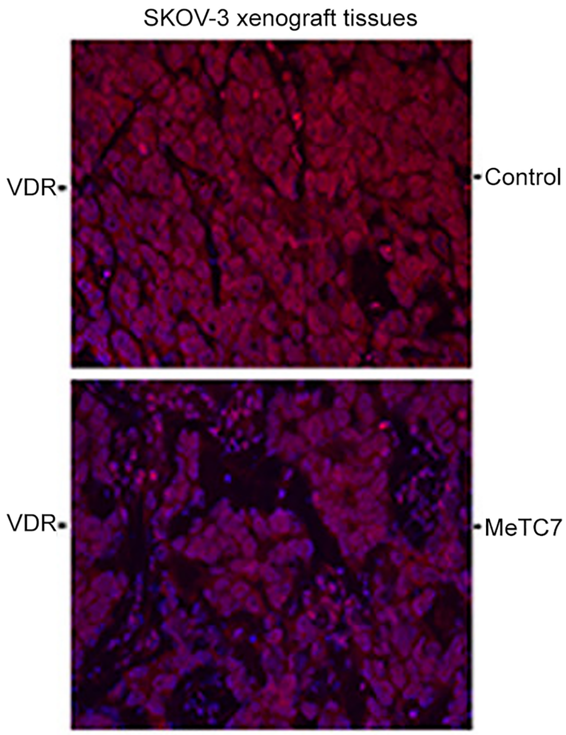

Supplementary Figure 5. 5 treatment downregulated VDR in ovarian cancer *in vivo*. Vehicle or MeTC7 (**5**) treated SKOV-3 ovarian cancer xenograft tissue harvested after euthanasia were formalin fixed and embedded as paraffin blocks. The slides (5µm) were stained with VDR (Santa Cruz Biotechnology, catalog number: SC13133) and DyLight594 (Vector Laboratories, catalog number: DI-2954). Vectashield mounting medium containing DAPI (Vector laboratories, catalog number: H-1200) was applied and cover-slipped. VDR expression was analyzed by confocal microscopy as described in the Material and Methods section.

Supplementary Figure 6.

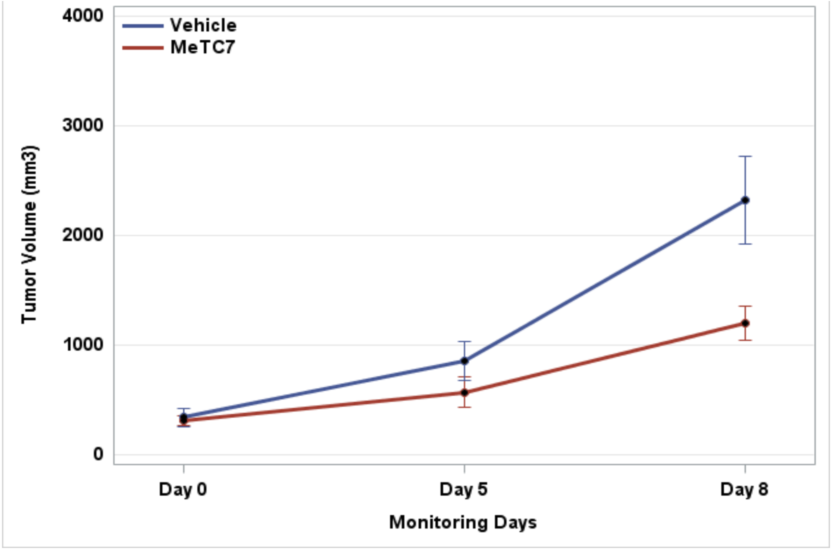

Supplementary Figure 6. MeTC7 (**5**) (10 mg/kg, M-F, IP, n=7) treatment reduced the growth rate of BE(2)-C xenograft tumors growing in NSG mice than vehicle (n=7). Repeated measures analysis of variance performed using maximum likelihood estimation with group, day, and the interaction between group and day as fixed effects was analyzed. The correlation of repeated measures on the same subject over time was handled using an unstructured covariance which was allowed to vary by treatment condition. Model assumptions were verified graphically. The tumor volumes in the treatment group were found to be statistically different from the control (p=0.0321). Tumor volumes were analyzed using SAS v9.4 Proc Mixed (Cary, NC).

Supplementary Figure 7.

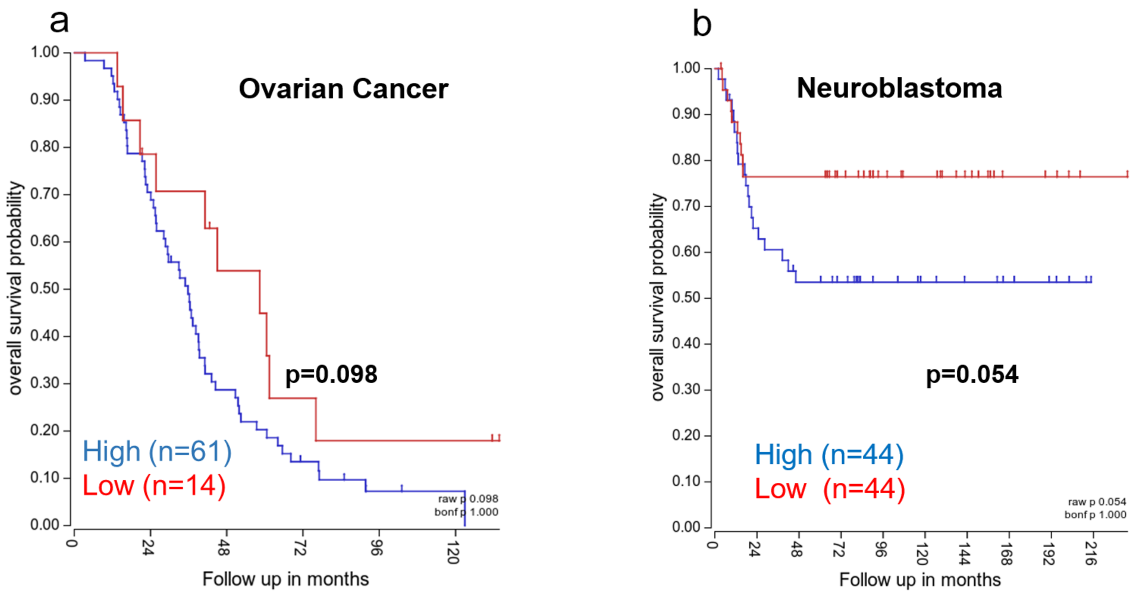

Supplementary Figure 7. (a) Analysis of the ovarian cancer microarray data (database: Pamula-Pilat-101-Mas5.0-U133P2, R2-genomics and visualization platform) showed that MYCN overexpression indicated poor prognosis. (**b**) Analysis of the neuroblastoma microarray data (database: Versteeg-88-MAS5.0-U133P2; R2-genomics and visualization platform) showed that MYCN overexpression indicated poor prognosis. Analysis is based on the platform suggested cutoff expression parameters.

Supplementary Figure 8.

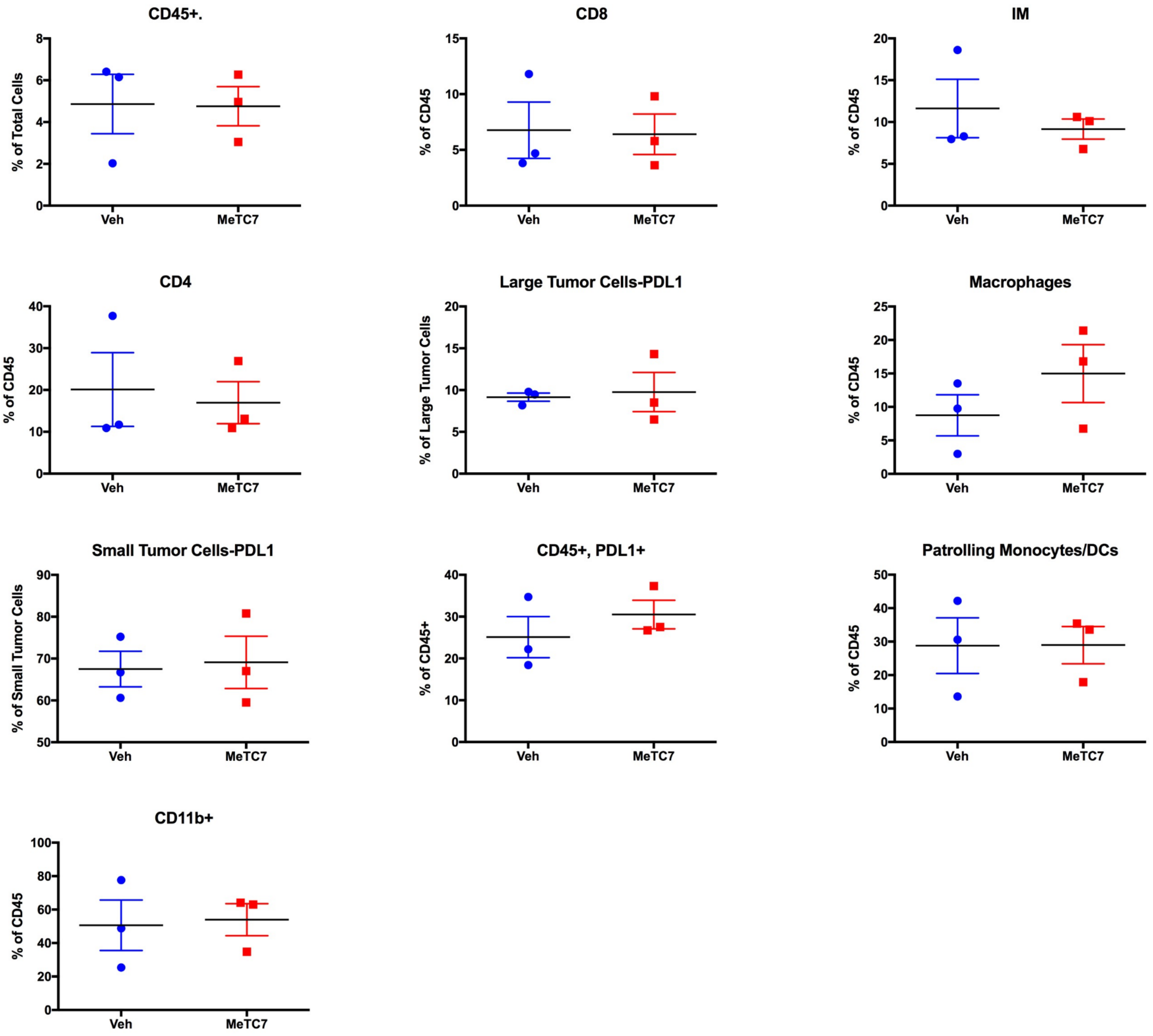

Supplementary Figure 8. Spontaneous TH-MYCN tumors treated with vehicle or MeTC7 (**5**) (10mg/kg, IP, once-daily) were isolated from the mice (described in **Figure 8**), broken into single cell suspension as described in Materials and Method section. The cells were stained with mouse flow-cytometric antibodies representing CD45, PD-L1, CD11b, CD4 and CD8 antigens and those representing patrolling monocytes/DCs, macrophages and inflammatory monocytes. Analysis of the data showed that **5** did not affect population of immune cells in the treated groups.
