# Supplementary material for: Identification of a Vitamin-D Receptor Antagonist, MeTC7, which Inhibits the Growth of Xenograft and Transgenic Tumors In Vivo
Source: J Med Chem. 2022 Apr 11;65(8):6039–55. doi: 10.1021/acs.jmedchem.1c01878 (PMC9059124; doi:10.1021/acs.jmedchem.1c01878)
Supplement: Supplementary file 4 — jm1c01878_si_004.pdf [file jm1c01878_si_004.pdf]

REMARK 4 COMPLIES WITH FORMAT V. 3.0, 1-DEC-2006

REMARK 888

REMARK 888 WRITTEN BY MAESTRO (A PRODUCT OF SCHRODINGER, LLC)

TITLE MeTC7-A

MODEL 1

ATOM 1 N LEU A 120 36.959 18.910 65.514 1.00 50.31
N

ATOM 2 CA LEU A 120 36.031 17.914 66.079 1.00 49.96
C

ATOM 3 C LEU A 120 35.027 17.568 64.955 1.00 49.44
C

ATOM 4 O LEU A 120 34.046 18.288 64.751 1.00 49.49
O

ATOM 5 CB LEU A 120 35.331 18.416 67.370 1.00 50.64
C

ATOM 6 CG LEU A 120 36.284 18.637 68.570 1.00 51.38
C

ATOM 7 CD1 LEU A 120 35.562 19.351 69.733 1.00 51.56
C

ATOM 8 CD2 LEU A 120 36.980 17.335 69.025 1.00 51.43
C

ATOM 9 H1 LEU A 120 37.735 19.051 66.145 1.00 0.00
H

ATOM 10 H2 LEU A 120 37.378 18.557 64.665 1.00 0.00
H

ATOM 11 HA LEU A 120 36.575 17.000 66.321 1.00 0.00
H

ATOM 12 HB3 LEU A 120 34.558 17.709 67.676 1.00 0.00
H

ATOM 13 HB2 LEU A 120 34.827 19.360 67.155 1.00 0.00
H

ATOM 14 HG LEU A 120 37.069 19.320 68.240 1.00 0.00
H

ATOM 15 HD11 LEU A 120 36.100 20.255 70.024 1.00 0.00
H

ATOM 16 HD12 LEU A 120 34.551 19.655 69.463 1.00 0.00

H
ATOM 17 HD13 LEU A 120 35.474 18.728 70.623 1.00 0.00
H
ATOM 18 HD21 LEU A 120 37.000 17.222 70.108 1.00 0.00
H
ATOM 19 HD22 LEU A 120 36.491 16.447 68.623 1.00 0.00
H
ATOM 20 HD23 LEU A 120 38.016 17.316 68.688 1.00 0.00
H
ATOM 21 N ARG A 121 35.322 16.477 64.227 1.00 48.14
N
ATOM 22 CA ARG A 121 34.563 15.993 63.075 1.00 47.01
C
ATOM 23 C ARG A 121 33.867 14.671 63.464 1.00 45.50
C
ATOM 24 O ARG A 121 34.547 13.644 63.500 1.00 45.36
O
ATOM 25 CB ARG A 121 35.548 15.776 61.904 1.00 48.45
C
ATOM 26 CG ARG A 121 36.153 17.083 61.354 1.00 49.89
C
ATOM 27 CD ARG A 121 37.302 16.863 60.357 1.00 52.32
C
ATOM 28 NE ARG A 121 36.883 16.075 59.190 1.00 53.49
N
ATOM 29 CZ ARG A 121 37.695 15.590 58.236 1.00 54.08
C
ATOM 30 NH1 ARG A 121 39.021 15.783 58.288 1.00 54.50
N
ATOM 31 NH2 ARG A 121 37.173 14.899 57.216 1.00 54.12
N1+
ATOM 32 H ARG A 121 36.143 15.939 64.464 1.00 0.00
H
ATOM 33 HA ARG A 121 33.850 16.745 62.745 1.00 0.00
H
ATOM 34 HB3 ARG A 121 35.033 15.258 61.094 1.00 0.00

H

ATOM 35 HB2 ARG A 121 36.356 15.113 62.219 1.00 0.00

H

ATOM 36 HG3 ARG A 121 36.579 17.636 62.190 1.00 0.00

H

ATOM 37 HG2 ARG A 121 35.387 17.734 60.931 1.00 0.00

H

ATOM 38 HD3 ARG A 121 38.155 16.407 60.861 1.00 0.00

H

ATOM 39 HD2 ARG A 121 37.638 17.828 59.976 1.00 0.00

H

ATOM 40 HE ARG A 121 35.881 15.971 59.069 1.00 0.00

H

ATOM 41 HH12 ARG A 121 39.625 15.415 57.568 1.00 0.00

H

ATOM 42 HH11 ARG A 121 39.424 16.302 59.055 1.00 0.00

H

ATOM 43 HH22 ARG A 121 37.768 14.531 56.488 1.00 0.00

H

ATOM 44 HH21 ARG A 121 36.179 14.714 57.172 1.00 0.00

H

ATOM 45 N PRO A 122 32.546 14.698 63.770 1.00 43.87

N

ATOM 46 CA PRO A 122 31.806 13.478 64.142 1.00 42.99

C

ATOM 47 C PRO A 122 31.606 12.525 62.952 1.00 42.25

C

ATOM 48 O PRO A 122 31.292 12.979 61.852 1.00 42.01

O

ATOM 49 CB PRO A 122 30.470 14.012 64.680 1.00 42.59

C

ATOM 50 CG PRO A 122 30.258 15.328 63.956 1.00 43.60

C

ATOM 51 CD PRO A 122 31.672 15.874 63.800 1.00 43.27

C

ATOM 52 HA PRO A 122 32.333 12.949 64.940 1.00 0.00

H
ATOM 53 HB3 PRO A 122 30.562 14.194 65.752 1.00 0.00
H
ATOM 54 HB2 PRO A 122 29.635 13.324 64.540 1.00 0.00
H
ATOM 55 HG3 PRO A 122 29.589 16.005 64.485 1.00 0.00
H
ATOM 56 HG2 PRO A 122 29.832 15.140 62.969 1.00 0.00
H
ATOM 57 HD2 PRO A 122 31.734 16.477 62.897 1.00 0.00
H
ATOM 58 HD3 PRO A 122 31.940 16.494 64.656 1.00 0.00
H
ATOM 59 N LYS A 123 31.803 11.222 63.206 1.00 41.53
N
ATOM 60 CA LYS A 123 31.603 10.147 62.235 1.00 41.30
C
ATOM 61 C LYS A 123 30.112 9.905 61.943 1.00 40.35
C
ATOM 62 O LYS A 123 29.282 10.026 62.845 1.00 39.21
O
ATOM 63 CB LYS A 123 32.245 8.851 62.771 1.00 42.56
C
ATOM 64 CG LYS A 123 33.776 8.918 62.902 1.00 45.26
C
ATOM 65 CD LYS A 123 34.375 7.598 63.411 1.00 46.98
C
ATOM 66 CE LYS A 123 35.901 7.656 63.559 1.00 47.93
C
ATOM 67 NZ LYS A 123 36.439 6.379 64.059 1.00 49.23
N1+
ATOM 68 H LYS A 123 32.058 10.933 64.139 1.00 0.00
H
ATOM 69 HA LYS A 123 32.102 10.428 61.307 1.00 0.00
H
ATOM 70 HB3 LYS A 123 32.000 8.027 62.098 1.00 0.00

H
ATOM 71 HB2 LYS A 123 31.804 8.591 63.735 1.00 0.00
H
ATOM 72 HG3 LYS A 123 34.055 9.724 63.581 1.00 0.00
H
ATOM 73 HG2 LYS A 123 34.212 9.167 61.934 1.00 0.00
H
ATOM 74 HD3 LYS A 123 34.106 6.792 62.727 1.00 0.00
H
ATOM 75 HD2 LYS A 123 33.925 7.345 64.372 1.00 0.00
H
ATOM 76 HE3 LYS A 123 36.185 8.451 64.251 1.00 0.00
H
ATOM 77 HE2 LYS A 123 36.367 7.882 62.599 1.00 0.00
H
ATOM 78 HZ1 LYS A 123 36.207 5.638 63.413 1.00 0.00
H
ATOM 79 HZ2 LYS A 123 37.443 6.445 64.148 1.00 0.00
H
ATOM 80 HZ3 LYS A 123 36.036 6.174 64.963 1.00 0.00
H
ATOM 81 N LEU A 124 29.819 9.506 60.695 1.00 39.48
N
ATOM 82 CA LEU A 124 28.513 8.998 60.277 1.00 38.64
C
ATOM 83 C LEU A 124 28.286 7.593 60.863 1.00 38.41
C
ATOM 84 O LEU A 124 29.072 6.689 60.576 1.00 39.12
O
ATOM 85 CB LEU A 124 28.464 8.931 58.735 1.00 37.76
C
ATOM 86 CG LEU A 124 28.360 10.295 58.023 1.00 37.83
C
ATOM 87 CD1 LEU A 124 28.795 10.168 56.557 1.00 37.98
C
ATOM 88 CD2 LEU A 124 26.951 10.912 58.136 1.00 36.97

C
ATOM 89 H LEU A 124 30.558 9.437 60.010 1.00 0.00
H
ATOM 90 HA LEU A 124 27.731 9.670 60.635 1.00 0.00
H
ATOM 91 HB3 LEU A 124 27.617 8.324 58.415 1.00 0.00
H
ATOM 92 HB2 LEU A 124 29.351 8.397 58.389 1.00 0.00
H
ATOM 93 HG LEU A 124 29.069 10.976 58.496 1.00 0.00
H
ATOM 94 HD11 LEU A 124 29.060 11.141 56.148 1.00 0.00
H
ATOM 95 HD12 LEU A 124 29.672 9.531 56.454 1.00 0.00
H
ATOM 96 HD13 LEU A 124 28.006 9.740 55.938 1.00 0.00
H
ATOM 97 HD21 LEU A 124 26.994 11.893 58.603 1.00 0.00
H
ATOM 98 HD22 LEU A 124 26.479 11.047 57.163 1.00 0.00
H
ATOM 99 HD23 LEU A 124 26.280 10.296 58.733 1.00 0.00
H
ATOM 100 N SER A 125 27.201 7.435 61.640 1.00 38.47
N
ATOM 101 CA SER A 125 26.721 6.141 62.134 1.00 39.40
C
ATOM 102 C SER A 125 26.125 5.288 60.998 1.00 40.52
C
ATOM 103 O SER A 125 25.767 5.834 59.954 1.00 39.53
O
ATOM 104 CB SER A 125 25.707 6.394 63.272 1.00 39.88
C
ATOM 105 OG SER A 125 24.461 6.870 62.798 1.00 40.18
O
ATOM 106 H SER A 125 26.609 8.225 61.846 1.00 0.00

H										
ATOM	107	HA	SER	A	125	27.575	5.604	62.552	1.00	0.00
H										
ATOM	108	HB3	SER	A	125	26.110	7.101	63.998	1.00	0.00
H										
ATOM	109	HB2	SER	A	125	25.523	5.466	63.815	1.00	0.00
H										
ATOM	110	HG	SER	A	125	23.880	7.011	63.551	1.00	0.00
H										
ATOM	111	N	GLU	A	126	25.995	3.970	61.229	1.00	41.33
N										
ATOM	112	CA	GLU	A	126	25.408	3.026	60.271	1.00	42.41
C										
ATOM	113	C	GLU	A	126	23.935	3.327	59.914	1.00	41.49
C										
ATOM	114	O	GLU	A	126	23.537	3.073	58.779	1.00	41.40
O										
ATOM	115	CB	GLU	A	126	25.640	1.579	60.770	1.00	45.02
C										
ATOM	116	CG	GLU	A	126	25.065	0.439	59.894	1.00	48.95
C										
ATOM	117	CD	GLU	A	126	25.572	0.418	58.445	1.00	51.93
C										
ATOM	118	OE1	GLU	A	126	24.817	-0.101	57.595	1.00	53.34
O										
ATOM	119	OE2	GLU	A	126	26.695	0.916	58.206	1.00	53.64
O1-										
ATOM	120	H	GLU	A	126	26.307	3.579	62.106	1.00	0.00
H										
ATOM	121	HA	GLU	A	126	25.975	3.151	59.349	1.00	0.00
H										
ATOM	122	HB3	GLU	A	126	25.226	1.480	61.774	1.00	0.00
H										
ATOM	123	HB2	GLU	A	126	26.713	1.417	60.886	1.00	0.00
H										
ATOM	124	HG3	GLU	A	126	23.975	0.486	59.884	1.00	0.00

H
ATOM 125 HG2 GLU A 126 25.317 -0.520 60.344 1.00 0.00
H
ATOM 126 N GLU A 127 23.179 3.919 60.857 1.00 40.27
N
ATOM 127 CA GLU A 127 21.832 4.445 60.633 1.00 39.52
C
ATOM 128 C GLU A 127 21.821 5.624 59.641 1.00 37.38
C
ATOM 129 O GLU A 127 21.017 5.609 58.711 1.00 36.24
O
ATOM 130 CB GLU A 127 21.192 4.806 61.990 1.00 41.53
C
ATOM 131 CG GLU A 127 19.780 5.426 61.894 1.00 45.78
C
ATOM 132 CD GLU A 127 19.142 5.694 63.258 1.00 47.87
C
ATOM 133 OE1 GLU A 127 19.886 6.095 64.181 1.00 49.93
O
ATOM 134 OE2 GLU A 127 17.908 5.514 63.346 1.00 50.45
O1-
ATOM 135 H GLU A 127 23.581 4.104 61.765 1.00 0.00
H
ATOM 136 HA GLU A 127 21.238 3.643 60.190 1.00 0.00
H
ATOM 137 HB3 GLU A 127 21.853 5.492 62.523 1.00 0.00
H
ATOM 138 HB2 GLU A 127 21.141 3.906 62.605 1.00 0.00
H
ATOM 139 HG3 GLU A 127 19.127 4.766 61.320 1.00 0.00
H
ATOM 140 HG2 GLU A 127 19.810 6.377 61.365 1.00 0.00
H
ATOM 141 N GLN A 128 22.726 6.597 59.842 1.00 34.92
N
ATOM 142 CA GLN A 128 22.895 7.770 58.979 1.00 33.94

C
ATOM 143 C GLN A 128 23.432 7.436 57.575 1.00 34.19
C
ATOM 144 O GLN A 128 22.998 8.063 56.610 1.00 32.45
O
ATOM 145 CB GLN A 128 23.750 8.831 59.698 1.00 33.20
C
ATOM 146 CG GLN A 128 23.023 9.454 60.911 1.00 32.00
C
ATOM 147 CD GLN A 128 23.704 10.697 61.494 1.00 32.20
C
ATOM 148 OE1 GLN A 128 23.032 11.567 62.041 1.00 33.66
O
ATOM 149 NE2 GLN A 128 25.031 10.797 61.393 1.00 30.80
N
ATOM 150 H GLN A 128 23.361 6.529 60.626 1.00 0.00
H
ATOM 151 HA GLN A 128 21.909 8.206 58.823 1.00 0.00
H
ATOM 152 HB3 GLN A 128 24.000 9.623 58.992 1.00 0.00
H
ATOM 153 HB2 GLN A 128 24.699 8.394 60.011 1.00 0.00
H
ATOM 154 HG3 GLN A 128 22.909 8.718 61.706 1.00 0.00
H
ATOM 155 HG2 GLN A 128 22.010 9.734 60.625 1.00 0.00
H
ATOM 156 HE22 GLN A 128 25.503 11.603 61.778 1.00 0.00
H
ATOM 157 HE21 GLN A 128 25.563 10.077 60.930 1.00 0.00
H
ATOM 158 N GLN A 129 24.310 6.422 57.478 1.00 33.75
N
ATOM 159 CA GLN A 129 24.789 5.846 56.219 1.00 35.00
C
ATOM 160 C GLN A 129 23.688 5.112 55.435 1.00 34.31

C
ATOM 161 O GLN A 129 23.671 5.207 54.210 1.00 33.78
O
ATOM 162 CB GLN A 129 25.977 4.901 56.486 1.00 37.89
C
ATOM 163 CG GLN A 129 27.258 5.643 56.915 1.00 42.41
C
ATOM 164 CD GLN A 129 28.475 4.742 57.147 1.00 45.15
C
ATOM 165 OE1 GLN A 129 29.604 5.206 57.016 1.00 46.46
O
ATOM 166 NE2 GLN A 129 28.279 3.467 57.496 1.00 46.27
N
ATOM 167 H GLN A 129 24.637 5.974 58.325 1.00 0.00
H
ATOM 168 HA GLN A 129 25.140 6.667 55.590 1.00 0.00
H
ATOM 169 HB3 GLN A 129 26.196 4.327 55.583 1.00 0.00
H
ATOM 170 HB2 GLN A 129 25.688 4.172 57.243 1.00 0.00
H
ATOM 171 HG3 GLN A 129 27.081 6.199 57.832 1.00 0.00
H
ATOM 172 HG2 GLN A 129 27.520 6.383 56.157 1.00 0.00
H
ATOM 173 HE22 GLN A 129 29.070 2.860 57.656 1.00 0.00
H
ATOM 174 HE21 GLN A 129 27.350 3.081 57.597 1.00 0.00
H
ATOM 175 N ARG A 130 22.786 4.419 56.152 1.00 33.44
N
ATOM 176 CA ARG A 130 21.629 3.729 55.582 1.00 34.05
C
ATOM 177 C ARG A 130 20.546 4.707 55.088 1.00 32.98
C
ATOM 178 O ARG A 130 19.961 4.453 54.038 1.00 31.44

O
ATOM 179 CB ARG A 130 21.093 2.700 56.600 1.00 37.04
C
ATOM 180 CG ARG A 130 19.909 1.862 56.089 1.00 41.36
C
ATOM 181 CD ARG A 130 19.526 0.727 57.048 1.00 45.69
C
ATOM 182 NE ARG A 130 18.278 0.071 56.634 1.00 49.25
N
ATOM 183 CZ ARG A 130 17.036 0.528 56.867 1.00 51.46
C
ATOM 184 NH1 ARG A 130 16.827 1.654 57.566 1.00 52.76
N
ATOM 185 NH2 ARG A 130 15.987 -0.154 56.391 1.00 52.73
N1+
ATOM 186 H ARG A 130 22.886 4.361 57.156 1.00 0.00
H
ATOM 187 HA ARG A 130 21.982 3.170 54.713 1.00 0.00
H
ATOM 188 HB3 ARG A 130 20.797 3.208 57.518 1.00 0.00
H
ATOM 189 HB2 ARG A 130 21.905 2.024 56.875 1.00 0.00
H
ATOM 190 HG3 ARG A 130 20.251 1.400 55.162 1.00 0.00
H
ATOM 191 HG2 ARG A 130 19.039 2.463 55.826 1.00 0.00
H
ATOM 192 HD3 ARG A 130 19.561 1.005 58.102 1.00 0.00
H
ATOM 193 HD2 ARG A 130 20.270 -0.059 56.920 1.00 0.00
H
ATOM 194 HE ARG A 130 18.388 -0.762 56.072 1.00 0.00
H
ATOM 195 HH12 ARG A 130 15.891 1.990 57.738 1.00 0.00
H
ATOM 196 HH11 ARG A 130 17.614 2.172 57.929 1.00 0.00

H

ATOM 197 HH22 ARG A 130 15.046 0.174 56.555 1.00 0.00

H

ATOM 198 HH21 ARG A 130 16.127 -1.003 55.864 1.00 0.00

H

ATOM 199 N ILE A 131 20.334 5.820 55.816 1.00 31.16

N

ATOM 200 CA ILE A 131 19.470 6.939 55.420 1.00 30.41

C

ATOM 201 C ILE A 131 19.954 7.613 54.121 1.00 29.21

C

ATOM 202 O ILE A 131 19.135 7.862 53.238 1.00 27.86

O

ATOM 203 CB ILE A 131 19.334 7.990 56.569 1.00 31.61

C

ATOM 204 CG1 ILE A 131 18.441 7.431 57.702 1.00 32.47

C

ATOM 205 CG2 ILE A 131 18.848 9.403 56.156 1.00 31.13

C

ATOM 206 CD1 ILE A 131 18.703 8.061 59.076 1.00 33.63

C

ATOM 207 H ILE A 131 20.837 5.939 56.686 1.00 0.00

H

ATOM 208 HA ILE A 131 18.480 6.523 55.217 1.00 0.00

H

ATOM 209 HB ILE A 131 20.330 8.125 56.989 1.00 0.00

H

ATOM 210 HG13 ILE A 131 18.588 6.355 57.801 1.00 0.00

H

ATOM 211 HG12 ILE A 131 17.389 7.553 57.440 1.00 0.00

H

ATOM 212 HG21 ILE A 131 18.754 10.062 57.020 1.00 0.00

H

ATOM 213 HG22 ILE A 131 19.543 9.900 55.478 1.00 0.00

H

ATOM 214 HG23 ILE A 131 17.876 9.366 55.667 1.00 0.00

H
ATOM 215 HD11 ILE A 131 18.115 7.564 59.846 1.00 0.00
H
ATOM 216 HD12 ILE A 131 19.748 7.972 59.365 1.00 0.00
H
ATOM 217 HD13 ILE A 131 18.450 9.119 59.089 1.00 0.00
H
ATOM 218 N ILE A 132 21.277 7.826 54.011 1.00 28.81
N
ATOM 219 CA ILE A 132 21.954 8.304 52.805 1.00 29.13
C
ATOM 220 C ILE A 132 21.814 7.335 51.615 1.00 29.37
C
ATOM 221 O ILE A 132 21.448 7.786 50.533 1.00 28.72
O
ATOM 222 CB ILE A 132 23.455 8.627 53.094 1.00 29.90
C
ATOM 223 CG1 ILE A 132 23.564 9.981 53.826 1.00 30.31
C
ATOM 224 CG2 ILE A 132 24.405 8.606 51.875 1.00 30.32
C
ATOM 225 CD1 ILE A 132 24.860 10.165 54.627 1.00 31.33
C
ATOM 226 H ILE A 132 21.878 7.600 54.792 1.00 0.00
H
ATOM 227 HA ILE A 132 21.459 9.233 52.512 1.00 0.00
H
ATOM 228 HB ILE A 132 23.823 7.866 53.783 1.00 0.00
H
ATOM 229 HG13 ILE A 132 22.727 10.090 54.513 1.00 0.00
H
ATOM 230 HG12 ILE A 132 23.460 10.789 53.103 1.00 0.00
H
ATOM 231 HG21 ILE A 132 25.416 8.905 52.147 1.00 0.00
H
ATOM 232 HG22 ILE A 132 24.498 7.607 51.452 1.00 0.00

H
ATOM 233 HG23 ILE A 132 24.060 9.281 51.091 1.00 0.00
H
ATOM 234 HD11 ILE A 132 24.881 11.145 55.106 1.00 0.00
H
ATOM 235 HD12 ILE A 132 24.958 9.413 55.409 1.00 0.00
H
ATOM 236 HD13 ILE A 132 25.736 10.093 53.988 1.00 0.00
H
ATOM 237 N ALA A 133 22.071 6.034 51.843 1.00 28.31
N
ATOM 238 CA ALA A 133 21.994 4.976 50.831 1.00 27.51
C
ATOM 239 C ALA A 133 20.584 4.768 50.250 1.00 26.94
C
ATOM 240 O ALA A 133 20.467 4.511 49.054 1.00 26.91
O
ATOM 241 CB ALA A 133 22.533 3.667 51.424 1.00 27.94
C
ATOM 242 H ALA A 133 22.376 5.744 52.763 1.00 0.00
H
ATOM 243 HA ALA A 133 22.650 5.267 50.008 1.00 0.00
H
ATOM 244 HB1 ALA A 133 22.521 2.864 50.686 1.00 0.00
H
ATOM 245 HB2 ALA A 133 23.564 3.784 51.760 1.00 0.00
H
ATOM 246 HB3 ALA A 133 21.940 3.341 52.279 1.00 0.00
H
ATOM 247 N ILE A 134 19.551 4.923 51.095 1.00 26.00
N
ATOM 248 CA ILE A 134 18.137 4.888 50.715 1.00 25.19
C
ATOM 249 C ILE A 134 17.725 6.087 49.831 1.00 24.84
C
ATOM 250 O ILE A 134 16.944 5.895 48.901 1.00 23.10

O

ATOM 251 CB ILE A 134 17.225 4.784 51.981 1.00 26.47

C

ATOM 252 CG1 ILE A 134 17.279 3.351 52.562 1.00 27.92

C

ATOM 253 CG2 ILE A 134 15.756 5.216 51.788 1.00 26.56

C

ATOM 254 CD1 ILE A 134 16.769 3.236 54.007 1.00 29.71

C

ATOM 255 H ILE A 134 19.740 5.110 52.071 1.00 0.00

H

ATOM 256 HA ILE A 134 17.986 3.988 50.115 1.00 0.00

H

ATOM 257 HB ILE A 134 17.645 5.453 52.734 1.00 0.00

H

ATOM 258 HG13 ILE A 134 18.302 2.973 52.535 1.00 0.00

H

ATOM 259 HG12 ILE A 134 16.706 2.679 51.923 1.00 0.00

H

ATOM 260 HG21 ILE A 134 15.164 5.065 52.690 1.00 0.00

H

ATOM 261 HG22 ILE A 134 15.681 6.277 51.554 1.00 0.00

H

ATOM 262 HG23 ILE A 134 15.277 4.655 50.985 1.00 0.00

H

ATOM 263 HD11 ILE A 134 17.099 2.301 54.457 1.00 0.00

H

ATOM 264 HD12 ILE A 134 17.137 4.052 54.629 1.00 0.00

H

ATOM 265 HD13 ILE A 134 15.680 3.246 54.047 1.00 0.00

H

ATOM 266 N LEU A 135 18.273 7.281 50.115 1.00 23.54

N

ATOM 267 CA LEU A 135 18.026 8.503 49.344 1.00 23.28

C

ATOM 268 C LEU A 135 18.810 8.549 48.018 1.00 22.05

C
ATOM 269 O LEU A 135 18.302 9.128 47.058 1.00 21.44
O
ATOM 270 CB LEU A 135 18.335 9.733 50.217 1.00 23.21
C
ATOM 271 CG LEU A 135 17.348 9.962 51.379 1.00 24.57
C
ATOM 272 CD1 LEU A 135 17.868 11.070 52.314 1.00 26.44
C
ATOM 273 CD2 LEU A 135 15.918 10.251 50.884 1.00 25.56
C
ATOM 274 H LEU A 135 18.907 7.367 50.898 1.00 0.00
H
ATOM 275 HA LEU A 135 16.968 8.528 49.078 1.00 0.00
H
ATOM 276 HB3 LEU A 135 18.324 10.630 49.601 1.00 0.00
H
ATOM 277 HB2 LEU A 135 19.351 9.643 50.605 1.00 0.00
H
ATOM 278 HG LEU A 135 17.293 9.049 51.970 1.00 0.00
H
ATOM 279 HD11 LEU A 135 17.941 10.706 53.339 1.00 0.00
H
ATOM 280 HD12 LEU A 135 18.861 11.416 52.025 1.00 0.00
H
ATOM 281 HD13 LEU A 135 17.227 11.951 52.320 1.00 0.00
H
ATOM 282 HD21 LEU A 135 15.386 10.922 51.557 1.00 0.00
H
ATOM 283 HD22 LEU A 135 15.913 10.704 49.892 1.00 0.00
H
ATOM 284 HD23 LEU A 135 15.339 9.329 50.826 1.00 0.00
H
ATOM 285 N LEU A 136 19.998 7.917 47.963 1.00 22.40
N
ATOM 286 CA LEU A 136 20.761 7.697 46.730 1.00 23.29

C

ATOM	287	C	LEU	A	136	20.029	6.722	45.795	1.00	23.50	C
ATOM	288	O	LEU	A	136	19.868	7.038	44.618	1.00	23.31	O
ATOM	289	CB	LEU	A	136	22.174	7.157	47.049	1.00	24.60	C
ATOM	290	CG	LEU	A	136	23.161	8.183	47.646	1.00	25.56	C
ATOM	291	CD1	LEU	A	136	24.458	7.474	48.092	1.00	26.67	C
ATOM	292	CD2	LEU	A	136	23.436	9.361	46.691	1.00	26.04	C
ATOM	293	H	LEU	A	136	20.377	7.500	48.803	1.00	0.00	H
ATOM	294	HA	LEU	A	136	20.845	8.650	46.206	1.00	0.00	H
ATOM	295	HB3	LEU	A	136	22.624	6.757	46.138	1.00	0.00	H
ATOM	296	HB2	LEU	A	136	22.079	6.308	47.726	1.00	0.00	H
ATOM	297	HG	LEU	A	136	22.709	8.609	48.539	1.00	0.00	H
ATOM	298	HD11	LEU	A	136	24.657	7.663	49.145	1.00	0.00	H
ATOM	299	HD12	LEU	A	136	24.400	6.392	47.971	1.00	0.00	H
ATOM	300	HD13	LEU	A	136	25.335	7.803	47.534	1.00	0.00	H
ATOM	301	HD21	LEU	A	136	24.494	9.607	46.606	1.00	0.00	H
ATOM	302	HD22	LEU	A	136	23.077	9.154	45.684	1.00	0.00	H
ATOM	303	HD23	LEU	A	136	22.930	10.263	47.037	1.00	0.00	H
ATOM	304	N	ASP	A	137	19.559	5.591	46.354	1.00	23.87	

N
ATOM 305 CA ASP A 137 18.733 4.576 45.693 1.00 23.95
C
ATOM 306 C ASP A 137 17.445 5.183 45.099 1.00 23.67
C
ATOM 307 O ASP A 137 17.125 4.903 43.944 1.00 22.79
O
ATOM 308 CB ASP A 137 18.424 3.409 46.667 1.00 26.93
C
ATOM 309 CG ASP A 137 17.591 2.265 46.080 1.00 31.10
C
ATOM 310 OD1 ASP A 137 16.748 1.726 46.830 1.00 34.78
O
ATOM 311 OD2 ASP A 137 17.844 1.898 44.912 1.00 34.13
O1-
ATOM 312 H ASP A 137 19.759 5.413 47.330 1.00 0.00
H
ATOM 313 HA ASP A 137 19.326 4.184 44.865 1.00 0.00
H
ATOM 314 HB3 ASP A 137 17.889 3.807 47.529 1.00 0.00
H
ATOM 315 HB2 ASP A 137 19.351 2.983 47.051 1.00 0.00
H
ATOM 316 N ALA A 138 16.771 6.042 45.885 1.00 22.31
N
ATOM 317 CA ALA A 138 15.584 6.792 45.484 1.00 22.31
C
ATOM 318 C ALA A 138 15.822 7.748 44.306 1.00 21.39
C
ATOM 319 O ALA A 138 14.940 7.868 43.459 1.00 20.83
O
ATOM 320 CB ALA A 138 15.034 7.563 46.693 1.00 23.04
C
ATOM 321 H ALA A 138 17.095 6.198 46.830 1.00 0.00
H
ATOM 322 HA ALA A 138 14.839 6.060 45.173 1.00 0.00

H
ATOM 323 HB1 ALA A 138 14.101 8.070 46.448 1.00 0.00
H
ATOM 324 HB2 ALA A 138 14.833 6.892 47.527 1.00 0.00
H
ATOM 325 HB3 ALA A 138 15.734 8.320 47.041 1.00 0.00
H
ATOM 326 N HIS A 139 16.996 8.392 44.261 1.00 21.06
N
ATOM 327 CA HIS A 139 17.383 9.284 43.168 1.00 21.28
C
ATOM 328 C HIS A 139 17.623 8.511 41.863 1.00 21.64
C
ATOM 329 O HIS A 139 17.076 8.888 40.829 1.00 21.17
O
ATOM 330 CB HIS A 139 18.618 10.118 43.555 1.00 21.21
C
ATOM 331 CG HIS A 139 18.882 11.299 42.647 1.00 22.53
C
ATOM 332 ND1 HIS A 139 20.152 11.783 42.381 1.00 25.49
N
ATOM 333 CD2 HIS A 139 18.027 12.105 41.925 1.00 19.49
C
ATOM 334 CE1 HIS A 139 20.019 12.839 41.573 1.00 20.82
C
ATOM 335 NE2 HIS A 139 18.753 13.089 41.256 1.00 23.99
N
ATOM 336 H HIS A 139 17.673 8.246 45.002 1.00 0.00
H
ATOM 337 HA HIS A 139 16.550 9.970 43.008 1.00 0.00
H
ATOM 338 HB3 HIS A 139 19.511 9.492 43.593 1.00 0.00
H
ATOM 339 HB2 HIS A 139 18.477 10.511 44.560 1.00 0.00
H
ATOM 340 HD1 HIS A 139 21.041 11.385 42.682 1.00 0.00

H
ATOM 341 HD2 HIS A 139 16.952 12.057 41.835 1.00 0.00
H
ATOM 342 HE1 HIS A 139 20.851 13.421 41.199 1.00 0.00
H
ATOM 343 N HIS A 140 18.367 7.399 41.940 1.00 21.50
N
ATOM 344 CA HIS A 140 18.691 6.541 40.793 1.00 22.20
C
ATOM 345 C HIS A 140 17.443 5.903 40.152 1.00 21.95
C
ATOM 346 O HIS A 140 17.412 5.734 38.933 1.00 21.87
O
ATOM 347 CB HIS A 140 19.712 5.460 41.208 1.00 23.76
C
ATOM 348 CG HIS A 140 20.982 5.961 41.859 1.00 25.80
C
ATOM 349 ND1 HIS A 140 21.708 5.188 42.773 1.00 27.26
N
ATOM 350 CD2 HIS A 140 21.620 7.177 41.706 1.00 26.22
C
ATOM 351 CE1 HIS A 140 22.723 5.959 43.144 1.00 26.08
C
ATOM 352 NE2 HIS A 140 22.719 7.150 42.545 1.00 27.71
N
ATOM 353 H HIS A 140 18.685 7.073 42.844 1.00 0.00
H
ATOM 354 HA HIS A 140 19.158 7.173 40.034 1.00 0.00
H
ATOM 355 HB3 HIS A 140 19.999 4.868 40.339 1.00 0.00
H
ATOM 356 HB2 HIS A 140 19.241 4.764 41.905 1.00 0.00
H
ATOM 357 HD2 HIS A 140 21.379 8.042 41.107 1.00 0.00
H
ATOM 358 HE1 HIS A 140 23.469 5.655 43.862 1.00 0.00

H
ATOM 359 HE2 HIS A 140 23.377 7.908 42.689 1.00 0.00
H
ATOM 360 N LYS A 141 16.431 5.604 40.983 1.00 20.41
N
ATOM 361 CA LYS A 141 15.123 5.079 40.589 1.00 21.89
C
ATOM 362 C LYS A 141 14.171 6.123 39.972 1.00 21.31
C
ATOM 363 O LYS A 141 13.145 5.717 39.424 1.00 22.47
O
ATOM 364 CB LYS A 141 14.480 4.419 41.828 1.00 23.28
C
ATOM 365 CG LYS A 141 15.060 3.032 42.144 1.00 26.83
C
ATOM 366 CD LYS A 141 14.595 2.491 43.503 1.00 30.63
C
ATOM 367 CE LYS A 141 14.956 1.011 43.693 1.00 33.64
C
ATOM 368 NZ LYS A 141 14.766 0.585 45.089 1.00 36.58
N1+
ATOM 369 H LYS A 141 16.561 5.754 41.974 1.00 0.00
H
ATOM 370 HA LYS A 141 15.282 4.310 39.830 1.00 0.00
H
ATOM 371 HB3 LYS A 141 13.407 4.291 41.678 1.00 0.00
H
ATOM 372 HB2 LYS A 141 14.582 5.081 42.689 1.00 0.00
H
ATOM 373 HG3 LYS A 141 16.150 3.068 42.129 1.00 0.00
H
ATOM 374 HG2 LYS A 141 14.769 2.339 41.354 1.00 0.00
H
ATOM 375 HD3 LYS A 141 13.517 2.623 43.606 1.00 0.00
H
ATOM 376 HD2 LYS A 141 15.051 3.085 44.296 1.00 0.00

H
ATOM 377 HE3 LYS A 141 15.996 0.832 43.420 1.00 0.00
H
ATOM 378 HE2 LYS A 141 14.345 0.388 43.040 1.00 0.00
H
ATOM 379 HZ1 LYS A 141 15.404 1.107 45.685 1.00 0.00
H
ATOM 380 HZ2 LYS A 141 13.815 0.757 45.380 1.00 0.00
H
ATOM 381 HZ3 LYS A 141 14.980 -0.397 45.175 1.00 0.00
H
ATOM 382 N THR A 142 14.500 7.421 40.081 1.00 20.65
N
ATOM 383 CA THR A 142 13.621 8.524 39.688 1.00 20.68
C
ATOM 384 C THR A 142 14.305 9.573 38.776 1.00 20.45
C
ATOM 385 O THR A 142 13.647 10.552 38.434 1.00 20.32
O
ATOM 386 CB THR A 142 13.026 9.235 40.943 1.00 20.48
C
ATOM 387 OG1 THR A 142 14.021 9.799 41.784 1.00 18.91
O
ATOM 388 CG2 THR A 142 12.151 8.310 41.804 1.00 19.94
C
ATOM 389 H THR A 142 15.375 7.695 40.514 1.00 0.00
H
ATOM 390 HA THR A 142 12.786 8.145 39.096 1.00 0.00
H
ATOM 391 HB THR A 142 12.386 10.055 40.619 1.00 0.00
H
ATOM 392 HG1 THR A 142 14.392 9.074 42.320 1.00 0.00
H
ATOM 393 HG21 THR A 142 11.749 8.845 42.662 1.00 0.00
H
ATOM 394 HG22 THR A 142 11.305 7.934 41.228 1.00 0.00

H
ATOM 395 HG23 THR A 142 12.700 7.448 42.179 1.00 0.00
H
ATOM 396 N TYR A 143 15.556 9.373 38.340 1.00 20.03
N
ATOM 397 CA TYR A 143 16.249 10.280 37.418 1.00 20.44
C
ATOM 398 C TYR A 143 16.845 9.432 36.285 1.00 20.75
C
ATOM 399 O TYR A 143 17.463 8.408 36.573 1.00 21.56
O
ATOM 400 CB TYR A 143 17.338 11.074 38.182 1.00 20.88
C
ATOM 401 CG TYR A 143 17.852 12.319 37.467 1.00 20.90
C
ATOM 402 CD1 TYR A 143 18.787 12.219 36.414 1.00 21.44
C
ATOM 403 CD2 TYR A 143 17.397 13.595 37.862 1.00 21.13
C
ATOM 404 CE1 TYR A 143 19.217 13.375 35.734 1.00 21.80
C
ATOM 405 CE2 TYR A 143 17.836 14.752 37.187 1.00 20.26
C
ATOM 406 CZ TYR A 143 18.728 14.641 36.107 1.00 22.15
C
ATOM 407 OH TYR A 143 19.114 15.755 35.419 1.00 21.28
O
ATOM 408 H TYR A 143 16.087 8.562 38.631 1.00 0.00
H
ATOM 409 HA TYR A 143 15.553 10.994 36.973 1.00 0.00
H
ATOM 410 HB3 TYR A 143 18.179 10.428 38.442 1.00 0.00
H
ATOM 411 HB2 TYR A 143 16.929 11.401 39.139 1.00 0.00
H
ATOM 412 HD1 TYR A 143 19.176 11.256 36.121 1.00 0.00

H
ATOM 413 HD2 TYR A 143 16.708 13.689 38.687 1.00 0.00
H
ATOM 414 HE1 TYR A 143 19.926 13.287 34.926 1.00 0.00
H
ATOM 415 HH TYR A 143 18.790 16.566 35.834 1.00 0.00
H
ATOM 416 HE2 TYR A 143 17.480 15.721 37.498 1.00 0.00
H
ATOM 417 N ASP A 144 16.673 9.866 35.028 1.00 20.48
N
ATOM 418 CA ASP A 144 17.257 9.229 33.842 1.00 21.36
C
ATOM 419 C ASP A 144 18.324 10.189 33.269 1.00 22.06
C
ATOM 420 O ASP A 144 17.946 11.112 32.550 1.00 21.75
O
ATOM 421 CB ASP A 144 16.181 8.884 32.772 1.00 21.65
C
ATOM 422 CG ASP A 144 16.753 8.296 31.472 1.00 22.22
C
ATOM 423 OD1 ASP A 144 17.881 7.758 31.517 1.00 22.12
O
ATOM 424 OD2 ASP A 144 16.046 8.380 30.446 1.00 23.20
O1-
ATOM 425 H ASP A 144 16.227 10.759 34.863 1.00 0.00
H
ATOM 426 HA ASP A 144 17.707 8.278 34.121 1.00 0.00
H
ATOM 427 HB3 ASP A 144 15.624 9.783 32.510 1.00 0.00
H
ATOM 428 HB2 ASP A 144 15.468 8.170 33.183 1.00 0.00
H
ATOM 429 N PRO A 145 19.627 9.966 33.551 1.00 21.93
N
ATOM 430 CA PRO A 145 20.683 10.864 33.051 1.00 23.05

C
ATOM 431 C PRO A 145 20.957 10.799 31.535 1.00 22.50
C
ATOM 432 O PRO A 145 21.726 11.626 31.050 1.00 23.61
O
ATOM 433 CB PRO A 145 21.906 10.478 33.894 1.00 23.45
C
ATOM 434 CG PRO A 145 21.696 9.006 34.216 1.00 24.95
C
ATOM 435 CD PRO A 145 20.183 8.893 34.380 1.00 23.26
C
ATOM 436 HA PRO A 145 20.418 11.902 33.258 1.00 0.00
H
ATOM 437 HB3 PRO A 145 21.905 11.062 34.815 1.00 0.00
H
ATOM 438 HB2 PRO A 145 22.859 10.664 33.395 1.00 0.00
H
ATOM 439 HG3 PRO A 145 22.253 8.667 35.090 1.00 0.00
H
ATOM 440 HG2 PRO A 145 22.015 8.403 33.364 1.00 0.00
H
ATOM 441 HD2 PRO A 145 19.845 7.900 34.081 1.00 0.00
H
ATOM 442 HD3 PRO A 145 19.896 9.056 35.419 1.00 0.00
H
ATOM 443 N THR A 146 20.354 9.851 30.803 1.00 22.03
N
ATOM 444 CA THR A 146 20.411 9.830 29.337 1.00 21.95
C
ATOM 445 C THR A 146 19.268 10.640 28.686 1.00 22.52
C
ATOM 446 O THR A 146 19.401 11.012 27.520 1.00 22.17
O
ATOM 447 CB THR A 146 20.380 8.374 28.786 1.00 21.96
C
ATOM 448 OG1 THR A 146 19.085 7.805 28.731 1.00 21.08

O
ATOM 449 CG2 THR A 146 21.340 7.407 29.499 1.00 23.14
C
ATOM 450 H THR A 146 19.780 9.134 31.228 1.00 0.00
H
ATOM 451 HA THR A 146 21.349 10.282 29.011 1.00 0.00
H
ATOM 452 HB THR A 146 20.711 8.426 27.748 1.00 0.00
H
ATOM 453 HG1 THR A 146 19.165 6.848 28.722 1.00 0.00
H
ATOM 454 HG21 THR A 146 21.360 6.436 29.003 1.00 0.00
H
ATOM 455 HG22 THR A 146 22.358 7.798 29.500 1.00 0.00
H
ATOM 456 HG23 THR A 146 21.046 7.239 30.536 1.00 0.00
H
ATOM 457 N TYR A 147 18.189 10.903 29.448 1.00 22.04
N
ATOM 458 CA TYR A 147 17.003 11.692 29.084 1.00 22.53
C
ATOM 459 C TYR A 147 16.167 11.069 27.943 1.00 23.10
C
ATOM 460 O TYR A 147 15.564 11.778 27.139 1.00 23.05
O
ATOM 461 CB TYR A 147 17.391 13.169 28.842 1.00 23.34
C
ATOM 462 CG TYR A 147 18.230 13.758 29.969 1.00 23.73
C
ATOM 463 CD1 TYR A 147 19.602 14.029 29.778 1.00 25.15
C
ATOM 464 CD2 TYR A 147 17.654 13.976 31.235 1.00 23.61
C
ATOM 465 CE1 TYR A 147 20.382 14.524 30.841 1.00 25.82
C
ATOM 466 CE2 TYR A 147 18.439 14.453 32.303 1.00 26.56

C
ATOM 467 CZ TYR A 147 19.803 14.732 32.107 1.00 26.11
C
ATOM 468 OH TYR A 147 20.566 15.196 33.137 1.00 29.64
O
ATOM 469 H TYR A 147 18.214 10.610 30.416 1.00 0.00
H
ATOM 470 HA TYR A 147 16.354 11.667 29.960 1.00 0.00
H
ATOM 471 HB3 TYR A 147 16.489 13.769 28.718 1.00 0.00
H
ATOM 472 HB2 TYR A 147 17.937 13.264 27.902 1.00 0.00
H
ATOM 473 HD1 TYR A 147 20.067 13.840 28.821 1.00 0.00
H
ATOM 474 HD2 TYR A 147 16.610 13.760 31.393 1.00 0.00
H
ATOM 475 HE1 TYR A 147 21.433 14.721 30.692 1.00 0.00
H
ATOM 476 HE2 TYR A 147 17.996 14.595 33.275 1.00 0.00
H
ATOM 477 HH TYR A 147 20.069 15.352 33.961 1.00 0.00
H
ATOM 478 N SER A 148 16.107 9.734 27.916 1.00 23.29
N
ATOM 479 CA SER A 148 15.460 8.912 26.889 1.00 23.65
C
ATOM 480 C SER A 148 13.922 9.016 26.861 1.00 24.65
C
ATOM 481 O SER A 148 13.340 8.878 25.785 1.00 24.62
O
ATOM 482 CB SER A 148 15.949 7.454 27.036 1.00 26.66
C
ATOM 483 OG SER A 148 15.411 6.802 28.172 1.00 29.82
O
ATOM 484 H SER A 148 16.500 9.224 28.698 1.00 0.00

H
ATOM 485 HA SER A 148 15.821 9.275 25.924 1.00 0.00
H
ATOM 486 HB3 SER A 148 17.036 7.414 27.077 1.00 0.00
H
ATOM 487 HB2 SER A 148 15.656 6.879 26.157 1.00 0.00
H
ATOM 488 HG SER A 148 15.684 7.274 28.969 1.00 0.00
H
ATOM 489 N ASP A 149 13.344 9.335 28.026 1.00 22.99
N
ATOM 490 CA ASP A 149 11.922 9.562 28.284 1.00 23.85
C
ATOM 491 C ASP A 149 11.320 10.716 27.464 1.00 24.00
C
ATOM 492 O ASP A 149 10.113 10.734 27.239 1.00 24.41
O
ATOM 493 CB ASP A 149 11.644 9.827 29.787 1.00 24.47
C
ATOM 494 CG ASP A 149 12.292 8.842 30.765 1.00 27.05
C
ATOM 495 OD1 ASP A 149 12.379 7.648 30.415 1.00 26.86
O
ATOM 496 OD2 ASP A 149 12.533 9.264 31.918 1.00 26.29
O1-
ATOM 497 H ASP A 149 13.942 9.339 28.839 1.00 0.00
H
ATOM 498 HA ASP A 149 11.395 8.653 27.990 1.00 0.00
H
ATOM 499 HB3 ASP A 149 10.567 9.821 29.963 1.00 0.00
H
ATOM 500 HB2 ASP A 149 11.999 10.829 30.036 1.00 0.00
H
ATOM 501 N PHE A 150 12.157 11.670 27.038 1.00 24.31
N
ATOM 502 CA PHE A 150 11.732 12.956 26.482 1.00 25.09

C
ATOM 503 C PHE A 150 11.001 12.869 25.128 1.00 25.91
C
ATOM 504 O PHE A 150 10.338 13.826 24.721 1.00 25.61
O
ATOM 505 CB PHE A 150 12.922 13.940 26.461 1.00 24.68
C
ATOM 506 CG PHE A 150 13.313 14.544 27.805 1.00 25.17
C
ATOM 507 CD1 PHE A 150 13.542 13.748 28.951 1.00 25.54
C
ATOM 508 CD2 PHE A 150 13.536 15.934 27.894 1.00 26.43
C
ATOM 509 CE1 PHE A 150 13.902 14.337 30.153 1.00 25.74
C
ATOM 510 CE2 PHE A 150 13.898 16.503 29.105 1.00 25.55
C
ATOM 511 CZ PHE A 150 14.069 15.711 30.232 1.00 24.63
C
ATOM 512 H PHE A 150 13.151 11.557 27.190 1.00 0.00
H
ATOM 513 HA PHE A 150 10.987 13.358 27.165 1.00 0.00
H
ATOM 514 HB3 PHE A 150 12.677 14.767 25.793 1.00 0.00
H
ATOM 515 HB2 PHE A 150 13.803 13.465 26.027 1.00 0.00
H
ATOM 516 HD1 PHE A 150 13.438 12.676 28.922 1.00 0.00
H
ATOM 517 HD2 PHE A 150 13.406 16.571 27.029 1.00 0.00
H
ATOM 518 HE1 PHE A 150 14.057 13.721 31.025 1.00 0.00
H
ATOM 519 HE2 PHE A 150 14.037 17.572 29.171 1.00 0.00
H
ATOM 520 HZ PHE A 150 14.351 16.172 31.168 1.00 0.00

H
ATOM 521 N CYS A 151 11.094 11.712 24.461 1.00 27.60
N
ATOM 522 CA CYS A 151 10.353 11.388 23.239 1.00 28.74
C
ATOM 523 C CYS A 151 8.844 11.144 23.476 1.00 28.90
C
ATOM 524 O CYS A 151 8.077 11.214 22.517 1.00 28.40
O
ATOM 525 CB CYS A 151 11.016 10.204 22.499 1.00 31.34
C
ATOM 526 SG CYS A 151 10.833 8.624 23.387 1.00 37.88
S
ATOM 527 H CYS A 151 11.660 10.965 24.838 1.00 0.00
H
ATOM 528 HA CYS A 151 10.425 12.257 22.583 1.00 0.00
H
ATOM 529 HB3 CYS A 151 12.076 10.400 22.337 1.00 0.00
H
ATOM 530 HB2 CYS A 151 10.568 10.086 21.512 1.00 0.00
H
ATOM 531 HG CYS A 151 11.528 7.889 22.514 1.00 0.00
H
ATOM 532 N GLN A 152 8.451 10.869 24.733 1.00 27.55
N
ATOM 533 CA GLN A 152 7.069 10.625 25.156 1.00 27.93
C
ATOM 534 C GLN A 152 6.240 11.922 25.265 1.00 27.73
C
ATOM 535 O GLN A 152 5.012 11.842 25.276 1.00 28.51
O
ATOM 536 CB GLN A 152 7.079 9.887 26.513 1.00 29.61
C
ATOM 537 CG GLN A 152 7.844 8.543 26.496 1.00 33.35
C
ATOM 538 CD GLN A 152 8.007 7.924 27.888 1.00 36.62

C

ATOM 539 OE1 GLN A 152 7.230 8.192 28.801 1.00 39.37

O

ATOM 540 NE2 GLN A 152 9.020 7.070 28.050 1.00 38.35

N

ATOM 541 H GLN A 152 9.144 10.834 25.468 1.00 0.00

H

ATOM 542 HA GLN A 152 6.586 9.983 24.417 1.00 0.00

H

ATOM 543 HB3 GLN A 152 6.051 9.701 26.830 1.00 0.00

H

ATOM 544 HB2 GLN A 152 7.505 10.543 27.272 1.00 0.00

H

ATOM 545 HG3 GLN A 152 8.842 8.678 26.079 1.00 0.00

H

ATOM 546 HG2 GLN A 152 7.331 7.831 25.850 1.00 0.00

H

ATOM 547 HE22 GLN A 152 9.187 6.647 28.951 1.00 0.00

H

ATOM 548 HE21 GLN A 152 9.649 6.864 27.287 1.00 0.00

H

ATOM 549 N PHE A 153 6.918 13.076 25.343 1.00 25.45

N

ATOM 550 CA PHE A 153 6.319 14.405 25.476 1.00 25.30

C

ATOM 551 C PHE A 153 5.903 14.956 24.099 1.00 25.61

C

ATOM 552 O PHE A 153 6.498 14.595 23.083 1.00 24.69

O

ATOM 553 CB PHE A 153 7.364 15.366 26.098 1.00 23.94

C

ATOM 554 CG PHE A 153 8.068 14.926 27.377 1.00 23.45

C

ATOM 555 CD1 PHE A 153 7.448 14.078 28.323 1.00 22.75

C

ATOM 556 CD2 PHE A 153 9.323 15.489 27.695 1.00 23.40

C
ATOM 557 CE1 PHE A 153 8.120 13.723 29.485 1.00 23.18
C
ATOM 558 CE2 PHE A 153 9.974 15.123 28.862 1.00 22.50
C
ATOM 559 CZ PHE A 153 9.384 14.233 29.745 1.00 22.91
C
ATOM 560 H PHE A 153 7.924 13.055 25.260 1.00 0.00
H
ATOM 561 HA PHE A 153 5.440 14.347 26.121 1.00 0.00
H
ATOM 562 HB3 PHE A 153 6.896 16.331 26.294 1.00 0.00
H
ATOM 563 HB2 PHE A 153 8.140 15.563 25.358 1.00 0.00
H
ATOM 564 HD1 PHE A 153 6.464 13.672 28.144 1.00 0.00
H
ATOM 565 HD2 PHE A 153 9.804 16.184 27.022 1.00 0.00
H
ATOM 566 HE1 PHE A 153 7.659 13.041 30.185 1.00 0.00
H
ATOM 567 HE2 PHE A 153 10.959 15.510 29.070 1.00 0.00
H
ATOM 568 HZ PHE A 153 9.915 13.947 30.638 1.00 0.00
H
ATOM 569 N ARG A 154 4.949 15.898 24.091 1.00 25.51
N
ATOM 570 CA ARG A 154 4.600 16.734 22.935 1.00 26.08
C
ATOM 571 C ARG A 154 5.820 17.601 22.550 1.00 27.20
C
ATOM 572 O ARG A 154 6.380 18.227 23.447 1.00 26.99
O
ATOM 573 CB ARG A 154 3.412 17.634 23.334 1.00 26.73
C
ATOM 574 CG ARG A 154 2.107 16.850 23.571 1.00 27.85

C
ATOM 575 CD ARG A 154 0.954 17.724 24.084 1.00 27.71
C
ATOM 576 NE ARG A 154 1.159 18.159 25.475 1.00 27.14
N
ATOM 577 CZ ARG A 154 0.410 19.067 26.122 1.00 28.47
C
ATOM 578 NH1 ARG A 154 -0.604 19.689 25.507 1.00 29.16
N
ATOM 579 NH2 ARG A 154 0.672 19.346 27.403 1.00 27.66
N1+
ATOM 580 H ARG A 154 4.527 16.172 24.972 1.00 0.00
H
ATOM 581 HA ARG A 154 4.293 16.065 22.132 1.00 0.00
H
ATOM 582 HB3 ARG A 154 3.234 18.363 22.542 1.00 0.00
H
ATOM 583 HB2 ARG A 154 3.668 18.211 24.224 1.00 0.00
H
ATOM 584 HG3 ARG A 154 2.309 16.103 24.339 1.00 0.00
H
ATOM 585 HG2 ARG A 154 1.799 16.285 22.691 1.00 0.00
H
ATOM 586 HD3 ARG A 154 -0.019 17.263 23.918 1.00 0.00
H
ATOM 587 HD2 ARG A 154 0.961 18.647 23.505 1.00 0.00
H
ATOM 588 HE ARG A 154 1.908 17.696 25.986 1.00 0.00
H
ATOM 589 HH12 ARG A 154 -1.211 20.325 26.012 1.00 0.00
H
ATOM 590 HH11 ARG A 154 -0.812 19.475 24.543 1.00 0.00
H
ATOM 591 HH22 ARG A 154 0.130 20.028 27.914 1.00 0.00
H
ATOM 592 HH21 ARG A 154 1.411 18.827 27.878 1.00 0.00

H
ATOM 593 N PRO A 155 6.267 17.567 21.273 1.00 27.28
N
ATOM 594 CA PRO A 155 7.609 18.056 20.888 1.00 28.12
C
ATOM 595 C PRO A 155 7.825 19.583 21.042 1.00 28.41
C
ATOM 596 O PRO A 155 6.847 20.334 21.048 1.00 27.77
O
ATOM 597 CB PRO A 155 7.739 17.607 19.420 1.00 28.83
C
ATOM 598 CG PRO A 155 6.310 17.503 18.913 1.00 29.19
C
ATOM 599 CD PRO A 155 5.561 16.985 20.132 1.00 28.61
C
ATOM 600 HA PRO A 155 8.334 17.518 21.501 1.00 0.00
H
ATOM 601 HB3 PRO A 155 8.212 16.624 19.387 1.00 0.00
H
ATOM 602 HB2 PRO A 155 8.344 18.275 18.805 1.00 0.00
H
ATOM 603 HG3 PRO A 155 6.205 16.865 18.035 1.00 0.00
H
ATOM 604 HG2 PRO A 155 5.944 18.498 18.654 1.00 0.00
H
ATOM 605 HD2 PRO A 155 4.507 17.265 20.097 1.00 0.00
H
ATOM 606 HD3 PRO A 155 5.632 15.898 20.188 1.00 0.00
H
ATOM 607 N PRO A 156 9.106 20.014 21.151 1.00 28.25
N
ATOM 608 CA PRO A 156 9.459 21.431 21.339 1.00 29.56
C
ATOM 609 C PRO A 156 9.313 22.258 20.047 1.00 30.08
C
ATOM 610 O PRO A 156 10.112 22.091 19.126 1.00 30.96

O
ATOM 611 CB PRO A 156 10.916 21.365 21.829 1.00 29.92
C
ATOM 612 CG PRO A 156 11.490 20.107 21.198 1.00 30.45
C
ATOM 613 CD PRO A 156 10.298 19.158 21.176 1.00 29.15
C
ATOM 614 HA PRO A 156 8.843 21.880 22.121 1.00 0.00
H
ATOM 615 HB3 PRO A 156 10.926 21.262 22.913 1.00 0.00
H
ATOM 616 HB2 PRO A 156 11.499 22.254 21.590 1.00 0.00
H
ATOM 617 HG3 PRO A 156 12.350 19.706 21.735 1.00 0.00
H
ATOM 618 HG2 PRO A 156 11.806 20.320 20.176 1.00 0.00
H
ATOM 619 HD2 PRO A 156 10.353 18.479 20.324 1.00 0.00
H
ATOM 620 HD3 PRO A 156 10.289 18.561 22.087 1.00 0.00
H
ATOM 621 N VAL A 157 8.308 23.146 20.017 1.00 30.75
N
ATOM 622 CA VAL A 157 8.096 24.107 18.932 1.00 31.52
C
ATOM 623 C VAL A 157 8.621 25.479 19.389 1.00 32.33
C
ATOM 624 O VAL A 157 8.313 25.900 20.503 1.00 33.09
O
ATOM 625 CB VAL A 157 6.586 24.247 18.576 1.00 31.97
C
ATOM 626 CG1 VAL A 157 6.296 25.293 17.476 1.00 32.48
C
ATOM 627 CG2 VAL A 157 5.983 22.894 18.157 1.00 32.24
C
ATOM 628 H VAL A 157 7.689 23.233 20.811 1.00 0.00

H
ATOM 629 HA VAL A 157 8.634 23.799 18.033 1.00 0.00
H
ATOM 630 HB VAL A 157 6.048 24.568 19.469 1.00 0.00
H
ATOM 631 HG11 VAL A 157 5.234 25.321 17.233 1.00 0.00
H
ATOM 632 HG12 VAL A 157 6.575 26.304 17.770 1.00 0.00
H
ATOM 633 HG13 VAL A 157 6.836 25.057 16.558 1.00 0.00
H
ATOM 634 HG21 VAL A 157 4.931 22.995 17.892 1.00 0.00
H
ATOM 635 HG22 VAL A 157 6.505 22.480 17.293 1.00 0.00
H
ATOM 636 HG23 VAL A 157 6.040 22.161 18.962 1.00 0.00
H
ATOM 637 N ARG A 158 9.393 26.147 18.518 1.00 33.83
N
ATOM 638 CA ARG A 158 9.967 27.470 18.768 1.00 36.19
C
ATOM 639 C ARG A 158 9.502 28.448 17.683 1.00 38.44
C
ATOM 640 O ARG A 158 9.644 28.165 16.493 1.00 39.20
O
ATOM 641 CB ARG A 158 11.505 27.374 18.824 1.00 35.86
C
ATOM 642 CG ARG A 158 12.022 26.608 20.055 1.00 34.74
C
ATOM 643 CD ARG A 158 13.554 26.528 20.092 1.00 35.06
C
ATOM 644 NE ARG A 158 14.029 25.892 21.328 1.00 32.63
N
ATOM 645 CZ ARG A 158 14.073 24.577 21.601 1.00 31.82
C
ATOM 646 NH1 ARG A 158 13.707 23.655 20.700 1.00 32.10

N
ATOM 647 NH2 ARG A 158 14.502 24.178 22.804 1.00 29.69
N1+
ATOM 648 H ARG A 158 9.589 25.747 17.611 1.00 0.00
H
ATOM 649 HA ARG A 158 9.624 27.849 19.731 1.00 0.00
H
ATOM 650 HB3 ARG A 158 11.923 28.382 18.852 1.00 0.00
H
ATOM 651 HB2 ARG A 158 11.884 26.912 17.912 1.00 0.00
H
ATOM 652 HG3 ARG A 158 11.617 25.596 20.056 1.00 0.00
H
ATOM 653 HG2 ARG A 158 11.658 27.082 20.968 1.00 0.00
H
ATOM 654 HD3 ARG A 158 13.969 27.535 20.044 1.00 0.00
H
ATOM 655 HD2 ARG A 158 13.946 25.987 19.231 1.00 0.00
H
ATOM 656 HE ARG A 158 14.272 26.543 22.069 1.00 0.00
H
ATOM 657 HH12 ARG A 158 13.751 22.672 20.932 1.00 0.00
H
ATOM 658 HH11 ARG A 158 13.383 23.936 19.787 1.00 0.00
H
ATOM 659 HH22 ARG A 158 14.558 23.192 23.028 1.00 0.00
H
ATOM 660 HH21 ARG A 158 14.812 24.864 23.488 1.00 0.00
H
ATOM 661 N VAL A 159 8.966 29.586 18.144 1.00 40.67
N
ATOM 662 CA VAL A 159 8.423 30.685 17.344 1.00 42.44
C
ATOM 663 C VAL A 159 9.383 31.894 17.316 1.00 43.24
C
ATOM 664 O VAL A 159 10.459 31.848 17.914 1.00 42.85

O
ATOM 665 CB VAL A 159 7.069 31.148 17.956 1.00 42.85
C
ATOM 666 CG1 VAL A 159 5.971 30.096 17.733 1.00 43.48
C
ATOM 667 CG2 VAL A 159 7.162 31.593 19.434 1.00 43.60
C
ATOM 668 H VAL A 159 8.890 29.714 19.142 1.00 0.00
H
ATOM 669 HA VAL A 159 8.262 30.361 16.314 1.00 0.00
H
ATOM 670 HB VAL A 159 6.716 32.018 17.404 1.00 0.00
H
ATOM 671 HG11 VAL A 159 5.031 30.399 18.194 1.00 0.00
H
ATOM 672 HG12 VAL A 159 5.780 29.958 16.668 1.00 0.00
H
ATOM 673 HG13 VAL A 159 6.255 29.127 18.137 1.00 0.00
H
ATOM 674 HG21 VAL A 159 6.175 31.784 19.852 1.00 0.00
H
ATOM 675 HG22 VAL A 159 7.627 30.837 20.064 1.00 0.00
H
ATOM 676 HG23 VAL A 159 7.739 32.512 19.543 1.00 0.00
H
ATOM 677 N ASN A 160 8.968 32.962 16.614 1.00 44.06
N
ATOM 678 CA ASN A 160 9.648 34.257 16.580 1.00 44.92
C
ATOM 679 C ASN A 160 9.426 35.000 17.910 1.00 45.24
C
ATOM 680 O ASN A 160 8.335 35.519 18.151 1.00 45.27
O
ATOM 681 CB ASN A 160 9.144 35.082 15.372 1.00 46.00
C
ATOM 682 CG ASN A 160 9.507 34.446 14.026 1.00 47.25

C
ATOM 683 OD1 ASN A 160 8.765 33.615 13.509 1.00 47.25
O
ATOM 684 ND2 ASN A 160 10.650 34.832 13.454 1.00 48.36
N
ATOM 685 H ASN A 160 8.084 32.919 16.126 1.00 0.00
H
ATOM 686 HA ASN A 160 10.714 34.063 16.438 1.00 0.00
H
ATOM 687 HB3 ASN A 160 9.569 36.087 15.404 1.00 0.00
H
ATOM 688 HB2 ASN A 160 8.061 35.206 15.418 1.00 0.00
H
ATOM 689 HD22 ASN A 160 10.926 34.434 12.569 1.00 0.00
H
ATOM 690 HD21 ASN A 160 11.243 35.518 13.898 1.00 0.00
H
ATOM 691 N ASP A 161 10.478 35.012 18.741 1.00 45.02
N
ATOM 692 CA ASP A 161 10.541 35.656 20.051 1.00 44.79
C
ATOM 693 C ASP A 161 12.010 35.633 20.507 1.00 44.74
C
ATOM 694 O ASP A 161 12.663 34.592 20.416 1.00 44.64
O
ATOM 695 CB ASP A 161 9.569 35.014 21.086 1.00 44.26
C
ATOM 696 CG ASP A 161 9.702 35.493 22.541 1.00 44.34
C
ATOM 697 OD1 ASP A 161 9.967 36.699 22.748 1.00 43.26
O
ATOM 698 OD2 ASP A 161 9.472 34.639 23.427 1.00 43.90
O1-
ATOM 699 H ASP A 161 11.324 34.535 18.461 1.00 0.00
H
ATOM 700 HA ASP A 161 10.255 36.699 19.904 1.00 0.00

H
ATOM 701 HB3 ASP A 161 9.716 33.936 21.070 1.00 0.00
H
ATOM 702 HB2 ASP A 161 8.535 35.183 20.787 1.00 0.00
H
ATOM 703 N GLY A 162 12.482 36.780 21.011 1.00 44.73
N
ATOM 704 CA GLY A 162 13.797 36.922 21.628 1.00 44.74
C
ATOM 705 C GLY A 162 13.800 38.053 22.669 1.00 44.56
C
ATOM 706 O GLY A 162 14.862 38.334 23.222 1.00 45.10
O
ATOM 707 H GLY A 162 11.875 37.585 21.032 1.00 0.00
H
ATOM 708 HA3 GLY A 162 14.537 37.137 20.857 1.00 0.00
H
ATOM 709 HA2 GLY A 162 14.098 35.998 22.125 1.00 0.00
H
ATOM 710 N GLY A 163 12.651 38.708 22.925 1.00 44.30
N
ATOM 711 CA GLY A 163 12.547 39.857 23.824 1.00 43.74
C
ATOM 712 C GLY A 163 11.914 39.473 25.169 1.00 43.23
C
ATOM 713 O GLY A 163 12.161 40.165 26.155 1.00 43.53
O
ATOM 714 H GLY A 163 11.794 38.398 22.489 1.00 0.00
H
ATOM 715 HA3 GLY A 163 11.913 40.606 23.350 1.00 0.00
H
ATOM 716 HA2 GLY A 163 13.513 40.335 23.995 1.00 0.00
H
ATOM 717 N GLY A 164 11.101 38.399 25.221 1.00 42.43
N
ATOM 718 CA GLY A 164 10.384 37.975 26.428 1.00 40.70

C

ATOM 719 C GLY A 164 9.233 38.928 26.802 1.00 39.47

C

ATOM 720 O GLY A 164 8.847 38.978 27.970 1.00 40.10

O

ATOM 721 H GLY A 164 10.932 37.857 24.383 1.00 0.00

H

ATOM 722 HA3 GLY A 164 11.076 37.879 27.266 1.00 0.00

H

ATOM 723 HA2 GLY A 164 9.963 36.986 26.247 1.00 0.00

H

ATOM 724 N SER A 216 8.704 39.696 25.830 1.00 36.82

N

ATOM 725 CA SER A 216 7.559 40.594 25.988 1.00 35.37

C

ATOM 726 C SER A 216 6.276 39.778 26.217 1.00 34.15

C

ATOM 727 O SER A 216 5.936 38.965 25.361 1.00 32.50

O

ATOM 728 CB SER A 216 7.465 41.492 24.736 1.00 35.39

C

ATOM 729 OG SER A 216 6.385 42.402 24.813 1.00 35.41

O

ATOM 730 H SER A 216 9.075 39.613 24.895 1.00 0.00

H

ATOM 731 HA SER A 216 7.740 41.230 26.857 1.00 0.00

H

ATOM 732 HB3 SER A 216 7.346 40.888 23.835 1.00 0.00

H

ATOM 733 HB2 SER A 216 8.385 42.063 24.612 1.00 0.00

H

ATOM 734 HG SER A 216 5.568 41.923 24.660 1.00 0.00

H

ATOM 735 N VAL A 217 5.612 39.999 27.364 1.00 33.39

N

ATOM 736 CA VAL A 217 4.508 39.189 27.899 1.00 33.22

C
ATOM 737 C VAL A 217 3.305 38.989 26.946 1.00 32.19
C
ATOM 738 O VAL A 217 2.716 37.909 26.952 1.00 31.92
O
ATOM 739 CB VAL A 217 4.023 39.763 29.265 1.00 33.21
C
ATOM 740 CG1 VAL A 217 2.748 39.115 29.847 1.00 35.52
C
ATOM 741 CG2 VAL A 217 5.148 39.686 30.316 1.00 35.70
C
ATOM 742 H VAL A 217 5.973 40.696 28.000 1.00 0.00
H
ATOM 743 HA VAL A 217 4.923 38.196 28.083 1.00 0.00
H
ATOM 744 HB VAL A 217 3.797 40.821 29.121 1.00 0.00
H
ATOM 745 HG11 VAL A 217 2.535 39.495 30.847 1.00 0.00
H
ATOM 746 HG12 VAL A 217 1.866 39.324 29.243 1.00 0.00
H
ATOM 747 HG13 VAL A 217 2.860 38.033 29.925 1.00 0.00
H
ATOM 748 HG21 VAL A 217 4.829 40.101 31.272 1.00 0.00
H
ATOM 749 HG22 VAL A 217 5.452 38.653 30.490 1.00 0.00
H
ATOM 750 HG23 VAL A 217 6.034 40.242 30.007 1.00 0.00
H
ATOM 751 N THR A 218 2.996 39.996 26.110 1.00 31.30
N
ATOM 752 CA THR A 218 1.981 39.909 25.054 1.00 30.58
C
ATOM 753 C THR A 218 2.354 38.894 23.951 1.00 30.30
C
ATOM 754 O THR A 218 1.493 38.123 23.529 1.00 30.29

O											
ATOM	755	CB	THR	A	218	1.747	41.290	24.383	1.00	30.65	C
ATOM	756	OG1	THR	A	218	1.355	42.222	25.371	1.00	30.30	O
ATOM	757	CG2	THR	A	218	0.695	41.320	23.257	1.00	31.23	C
ATOM	758	H	THR	A	218	3.499	40.869	26.173	1.00	0.00	H
ATOM	759	HA	THR	A	218	1.046	39.580	25.513	1.00	0.00	H
ATOM	760	HB	THR	A	218	2.692	41.652	23.974	1.00	0.00	H
ATOM	761	HG1	THR	A	218	1.072	43.030	24.937	1.00	0.00	H
ATOM	762	HG21	THR	A	218	0.518	42.339	22.911	1.00	0.00	H
ATOM	763	HG22	THR	A	218	1.011	40.743	22.387	1.00	0.00	H
ATOM	764	HG23	THR	A	218	-0.258	40.914	23.597	1.00	0.00	H
ATOM	765	N	LEU	A	219	3.635	38.891	23.541	1.00	29.44	N
ATOM	766	CA	LEU	A	219	4.210	37.977	22.552	1.00	29.87	C
ATOM	767	C	LEU	A	219	4.355	36.537	23.086	1.00	29.21	C
ATOM	768	O	LEU	A	219	4.235	35.600	22.296	1.00	29.73	O
ATOM	769	CB	LEU	A	219	5.559	38.559	22.064	1.00	31.48	C
ATOM	770	CG	LEU	A	219	6.124	37.932	20.771	1.00	33.59	C
ATOM	771	CD1	LEU	A	219	5.185	38.133	19.563	1.00	34.93	C
ATOM	772	CD2	LEU	A	219	7.545	38.453	20.480	1.00	35.57	

C
ATOM 773 H LEU A 219 4.292 39.519 23.983 1.00 0.00
H
ATOM 774 HA LEU A 219 3.517 37.946 21.710 1.00 0.00
H
ATOM 775 HB3 LEU A 219 6.297 38.455 22.860 1.00 0.00
H
ATOM 776 HB2 LEU A 219 5.452 39.632 21.902 1.00 0.00
H
ATOM 777 HG LEU A 219 6.229 36.861 20.940 1.00 0.00
H
ATOM 778 HD11 LEU A 219 5.730 38.365 18.647 1.00 0.00
H
ATOM 779 HD12 LEU A 219 4.606 37.231 19.366 1.00 0.00
H
ATOM 780 HD13 LEU A 219 4.475 38.942 19.729 1.00 0.00
H
ATOM 781 HD21 LEU A 219 8.193 37.635 20.169 1.00 0.00
H
ATOM 782 HD22 LEU A 219 7.559 39.199 19.685 1.00 0.00
H
ATOM 783 HD23 LEU A 219 8.005 38.911 21.356 1.00 0.00
H
ATOM 784 N GLU A 220 4.567 36.385 24.409 1.00 28.82
N
ATOM 785 CA GLU A 220 4.573 35.100 25.112 1.00 29.09
C
ATOM 786 C GLU A 220 3.183 34.442 25.084 1.00 29.07
C
ATOM 787 O GLU A 220 3.043 33.372 24.494 1.00 27.80
O
ATOM 788 CB GLU A 220 5.051 35.263 26.574 1.00 31.33
C
ATOM 789 CG GLU A 220 6.485 35.797 26.751 1.00 32.76
C
ATOM 790 CD GLU A 220 6.926 35.874 28.219 1.00 35.67

C
ATOM 791 OE1 GLU A 220 8.149 36.015 28.428 1.00 37.03
O
ATOM 792 OE2 GLU A 220 6.052 35.803 29.111 1.00 36.27
O1-
ATOM 793 H GLU A 220 4.686 37.206 24.986 1.00 0.00
H
ATOM 794 HA GLU A 220 5.274 34.437 24.601 1.00 0.00
H
ATOM 795 HB3 GLU A 220 4.980 34.294 27.070 1.00 0.00
H
ATOM 796 HB2 GLU A 220 4.366 35.917 27.113 1.00 0.00
H
ATOM 797 HG3 GLU A 220 6.579 36.791 26.326 1.00 0.00
H
ATOM 798 HG2 GLU A 220 7.187 35.173 26.199 1.00 0.00
H
ATOM 799 N LEU A 221 2.196 35.106 25.713 1.00 28.79
N
ATOM 800 CA LEU A 221 0.832 34.614 25.938 1.00 29.82
C
ATOM 801 C LEU A 221 -0.000 34.383 24.663 1.00 29.77
C
ATOM 802 O LEU A 221 -0.904 33.548 24.698 1.00 30.04
O
ATOM 803 CB LEU A 221 0.089 35.586 26.882 1.00 30.05
C
ATOM 804 CG LEU A 221 0.579 35.589 28.347 1.00 31.65
C
ATOM 805 CD1 LEU A 221 -0.157 36.672 29.164 1.00 32.22
C
ATOM 806 CD2 LEU A 221 0.473 34.204 29.019 1.00 31.89
C
ATOM 807 H LEU A 221 2.411 35.994 26.148 1.00 0.00
H
ATOM 808 HA LEU A 221 0.920 33.643 26.424 1.00 0.00

H
ATOM 809 HB3 LEU A 221 -0.973 35.344 26.891 1.00 0.00
H
ATOM 810 HB2 LEU A 221 0.155 36.595 26.470 1.00 0.00
H
ATOM 811 HG LEU A 221 1.635 35.865 28.341 1.00 0.00
H
ATOM 812 HD11 LEU A 221 0.538 37.218 29.801 1.00 0.00
H
ATOM 813 HD12 LEU A 221 -0.649 37.403 28.522 1.00 0.00
H
ATOM 814 HD13 LEU A 221 -0.928 36.250 29.809 1.00 0.00
H
ATOM 815 HD21 LEU A 221 0.099 34.268 30.040 1.00 0.00
H
ATOM 816 HD22 LEU A 221 -0.190 33.530 28.476 1.00 0.00
H
ATOM 817 HD23 LEU A 221 1.455 33.732 29.069 1.00 0.00
H
ATOM 818 N SER A 222 0.313 35.100 23.571 1.00 31.28
N
ATOM 819 CA SER A 222 -0.343 34.942 22.269 1.00 32.25
C
ATOM 820 C SER A 222 0.138 33.719 21.459 1.00 32.84
C
ATOM 821 O SER A 222 -0.503 33.406 20.455 1.00 33.76
O
ATOM 822 CB SER A 222 -0.215 36.257 21.469 1.00 34.81
C
ATOM 823 OG SER A 222 1.113 36.517 21.057 1.00 39.03
O
ATOM 824 H SER A 222 1.058 35.781 23.618 1.00 0.00
H
ATOM 825 HA SER A 222 -1.408 34.791 22.452 1.00 0.00
H
ATOM 826 HB3 SER A 222 -0.580 37.100 22.056 1.00 0.00

H
ATOM 827 HB2 SER A 222 -0.840 36.212 20.576 1.00 0.00
H
ATOM 828 HG SER A 222 1.584 36.913 21.796 1.00 0.00
H
ATOM 829 N GLN A 223 1.235 33.064 21.884 1.00 31.84
N
ATOM 830 CA GLN A 223 1.871 31.964 21.156 1.00 31.75
C
ATOM 831 C GLN A 223 1.945 30.699 22.026 1.00 30.53
C
ATOM 832 O GLN A 223 1.271 29.721 21.701 1.00 30.69
O
ATOM 833 CB GLN A 223 3.247 32.416 20.617 1.00 34.71
C
ATOM 834 CG GLN A 223 3.138 33.590 19.620 1.00 38.65
C
ATOM 835 CD GLN A 223 4.458 33.908 18.921 1.00 42.46
C
ATOM 836 OE1 GLN A 223 4.646 33.562 17.758 1.00 45.44
O
ATOM 837 NE2 GLN A 223 5.378 34.570 19.625 1.00 44.57
N
ATOM 838 H GLN A 223 1.700 33.369 22.728 1.00 0.00
H
ATOM 839 HA GLN A 223 1.268 31.691 20.288 1.00 0.00
H
ATOM 840 HB3 GLN A 223 3.728 31.568 20.126 1.00 0.00
H
ATOM 841 HB2 GLN A 223 3.900 32.703 21.443 1.00 0.00
H
ATOM 842 HG3 GLN A 223 2.787 34.490 20.123 1.00 0.00
H
ATOM 843 HG2 GLN A 223 2.395 33.357 18.856 1.00 0.00
H
ATOM 844 HE22 GLN A 223 6.262 34.816 19.200 1.00 0.00

H
ATOM 845 HE21 GLN A 223 5.185 34.852 20.577 1.00 0.00
H
ATOM 846 N LEU A 224 2.734 30.751 23.118 1.00 28.64
N
ATOM 847 CA LEU A 224 2.959 29.688 24.119 1.00 27.07
C
ATOM 848 C LEU A 224 3.450 28.349 23.509 1.00 26.40
C
ATOM 849 O LEU A 224 2.933 27.282 23.839 1.00 25.81
O
ATOM 850 CB LEU A 224 1.693 29.496 24.996 1.00 27.70
C
ATOM 851 CG LEU A 224 1.238 30.730 25.800 1.00 27.88
C
ATOM 852 CD1 LEU A 224 -0.160 30.509 26.418 1.00 29.53
C
ATOM 853 CD2 LEU A 224 2.266 31.107 26.877 1.00 27.54
C
ATOM 854 H LEU A 224 3.142 31.647 23.363 1.00 0.00
H
ATOM 855 HA LEU A 224 3.769 30.032 24.762 1.00 0.00
H
ATOM 856 HB3 LEU A 224 1.852 28.674 25.693 1.00 0.00
H
ATOM 857 HB2 LEU A 224 0.878 29.189 24.345 1.00 0.00
H
ATOM 858 HG LEU A 224 1.144 31.571 25.113 1.00 0.00
H
ATOM 859 HD11 LEU A 224 -0.884 31.212 26.006 1.00 0.00
H
ATOM 860 HD12 LEU A 224 -0.543 29.507 26.228 1.00 0.00
H
ATOM 861 HD13 LEU A 224 -0.166 30.641 27.498 1.00 0.00
H
ATOM 862 HD21 LEU A 224 1.835 31.749 27.643 1.00 0.00

H
ATOM 863 HD22 LEU A 224 2.654 30.218 27.374 1.00 0.00
H
ATOM 864 HD23 LEU A 224 3.113 31.644 26.453 1.00 0.00
H
ATOM 865 N SER A 225 4.426 28.428 22.600 1.00 25.74
N
ATOM 866 CA SER A 225 4.872 27.334 21.735 1.00 25.83
C
ATOM 867 C SER A 225 5.665 26.211 22.433 1.00 25.18
C
ATOM 868 O SER A 225 5.520 25.056 22.031 1.00 25.89
O
ATOM 869 CB SER A 225 5.625 27.943 20.540 1.00 26.31
C
ATOM 870 OG SER A 225 6.917 28.405 20.882 1.00 29.36
O
ATOM 871 H SER A 225 4.852 29.327 22.424 1.00 0.00
H
ATOM 872 HA SER A 225 3.968 26.872 21.330 1.00 0.00
H
ATOM 873 HB3 SER A 225 5.049 28.764 20.114 1.00 0.00
H
ATOM 874 HB2 SER A 225 5.732 27.195 19.755 1.00 0.00
H
ATOM 875 HG SER A 225 7.495 27.638 20.962 1.00 0.00
H
ATOM 876 N MET A 226 6.458 26.548 23.454 1.00 24.58
N
ATOM 877 CA MET A 226 7.229 25.562 24.213 1.00 24.51
C
ATOM 878 C MET A 226 6.421 24.990 25.392 1.00 24.00
C
ATOM 879 O MET A 226 6.848 23.986 25.968 1.00 24.19
O
ATOM 880 CB MET A 226 8.555 26.191 24.689 1.00 24.70

C
ATOM 881 CG MET A 226 9.641 26.299 23.607 1.00 25.85
C
ATOM 882 SD MET A 226 10.333 24.734 22.987 1.00 27.27
S
ATOM 883 CE MET A 226 11.017 23.998 24.499 1.00 28.07
C
ATOM 884 H MET A 226 6.516 27.526 23.734 1.00 0.00
H
ATOM 885 HA MET A 226 7.474 24.709 23.577 1.00 0.00
H
ATOM 886 HB3 MET A 226 8.960 25.617 25.519 1.00 0.00
H
ATOM 887 HB2 MET A 226 8.370 27.184 25.096 1.00 0.00
H
ATOM 888 HG3 MET A 226 10.472 26.890 23.989 1.00 0.00
H
ATOM 889 HG2 MET A 226 9.251 26.862 22.758 1.00 0.00
H
ATOM 890 HE1 MET A 226 11.659 23.153 24.250 1.00 0.00
H
ATOM 891 HE2 MET A 226 11.614 24.721 25.052 1.00 0.00
H
ATOM 892 HE3 MET A 226 10.218 23.637 25.146 1.00 0.00
H
ATOM 893 N LEU A 227 5.277 25.612 25.733 1.00 23.05
N
ATOM 894 CA LEU A 227 4.464 25.238 26.896 1.00 23.85
C
ATOM 895 C LEU A 227 3.977 23.773 26.912 1.00 23.65
C
ATOM 896 O LEU A 227 4.188 23.145 27.944 1.00 24.44
O
ATOM 897 CB LEU A 227 3.338 26.267 27.179 1.00 24.02
C
ATOM 898 CG LEU A 227 2.525 26.025 28.478 1.00 24.67

C
ATOM 899 CD1 LEU A 227 3.406 26.029 29.746 1.00 25.75
C
ATOM 900 CD2 LEU A 227 1.356 27.020 28.596 1.00 25.81
C
ATOM 901 H LEU A 227 4.943 26.372 25.157 1.00 0.00
H
ATOM 902 HA LEU A 227 5.156 25.306 27.736 1.00 0.00
H
ATOM 903 HB3 LEU A 227 2.649 26.295 26.337 1.00 0.00
H
ATOM 904 HB2 LEU A 227 3.765 27.265 27.236 1.00 0.00
H
ATOM 905 HG LEU A 227 2.064 25.038 28.413 1.00 0.00
H
ATOM 906 HD11 LEU A 227 2.945 26.562 30.577 1.00 0.00
H
ATOM 907 HD12 LEU A 227 3.596 25.012 30.090 1.00 0.00
H
ATOM 908 HD13 LEU A 227 4.372 26.504 29.579 1.00 0.00
H
ATOM 909 HD21 LEU A 227 0.436 26.500 28.866 1.00 0.00
H
ATOM 910 HD22 LEU A 227 1.530 27.777 29.360 1.00 0.00
H
ATOM 911 HD23 LEU A 227 1.162 27.553 27.666 1.00 0.00
H
ATOM 912 N PRO A 228 3.456 23.204 25.794 1.00 24.00
N
ATOM 913 CA PRO A 228 3.127 21.764 25.733 1.00 23.78
C
ATOM 914 C PRO A 228 4.270 20.810 26.110 1.00 23.69
C
ATOM 915 O PRO A 228 4.027 19.826 26.801 1.00 23.20
O
ATOM 916 CB PRO A 228 2.658 21.549 24.285 1.00 24.23

C
ATOM 917 CG PRO A 228 2.118 22.903 23.864 1.00 24.58
C
ATOM 918 CD PRO A 228 3.064 23.876 24.554 1.00 23.63
C
ATOM 919 HA PRO A 228 2.294 21.585 26.411 1.00 0.00
H
ATOM 920 HB3 PRO A 228 1.908 20.762 24.207 1.00 0.00
H
ATOM 921 HB2 PRO A 228 3.495 21.272 23.641 1.00 0.00
H
ATOM 922 HG3 PRO A 228 1.112 23.026 24.266 1.00 0.00
H
ATOM 923 HG2 PRO A 228 2.069 23.039 22.782 1.00 0.00
H
ATOM 924 HD2 PRO A 228 3.949 24.034 23.938 1.00 0.00
H
ATOM 925 HD3 PRO A 228 2.579 24.837 24.712 1.00 0.00
H
ATOM 926 N HIS A 229 5.494 21.133 25.674 1.00 22.38
N
ATOM 927 CA HIS A 229 6.683 20.340 25.965 1.00 22.16
C
ATOM 928 C HIS A 229 7.094 20.468 27.440 1.00 21.32
C
ATOM 929 O HIS A 229 7.241 19.457 28.127 1.00 20.38
O
ATOM 930 CB HIS A 229 7.817 20.742 24.998 1.00 22.65
C
ATOM 931 CG HIS A 229 8.968 19.771 24.960 1.00 23.54
C
ATOM 932 ND1 HIS A 229 8.793 18.435 24.663 1.00 24.88
N
ATOM 933 CD2 HIS A 229 10.316 19.925 25.163 1.00 23.99
C
ATOM 934 CE1 HIS A 229 9.979 17.835 24.738 1.00 25.42

C
ATOM 935 NE2 HIS A 229 10.950 18.689 25.032 1.00 23.32
N
ATOM 936 H HIS A 229 5.638 22.001 25.181 1.00 0.00
H
ATOM 937 HA HIS A 229 6.426 19.294 25.790 1.00 0.00
H
ATOM 938 HB3 HIS A 229 8.205 21.732 25.244 1.00 0.00
H
ATOM 939 HB2 HIS A 229 7.426 20.815 23.982 1.00 0.00
H
ATOM 940 HD1 HIS A 229 7.901 18.036 24.362 1.00 0.00
H
ATOM 941 HD2 HIS A 229 10.874 20.827 25.369 1.00 0.00
H
ATOM 942 HE1 HIS A 229 10.141 16.781 24.553 1.00 0.00
H
ATOM 943 N LEU A 230 7.216 21.716 27.911 1.00 20.90
N
ATOM 944 CA LEU A 230 7.595 22.039 29.286 1.00 21.22
C
ATOM 945 C LEU A 230 6.539 21.630 30.333 1.00 21.08
C
ATOM 946 O LEU A 230 6.912 21.249 31.444 1.00 19.50
O
ATOM 947 CB LEU A 230 7.970 23.528 29.367 1.00 21.98
C
ATOM 948 CG LEU A 230 9.460 23.788 29.017 1.00 23.32
C
ATOM 949 CD1 LEU A 230 9.691 25.082 28.211 1.00 25.98
C
ATOM 950 CD2 LEU A 230 10.361 23.700 30.271 1.00 24.89
C
ATOM 951 H LEU A 230 7.026 22.501 27.297 1.00 0.00
H
ATOM 952 HA LEU A 230 8.491 21.467 29.515 1.00 0.00

H
ATOM 953 HB3 LEU A 230 7.762 23.898 30.371 1.00 0.00
H
ATOM 954 HB2 LEU A 230 7.300 24.094 28.719 1.00 0.00
H
ATOM 955 HG LEU A 230 9.769 22.984 28.348 1.00 0.00
H
ATOM 956 HD11 LEU A 230 10.221 24.865 27.285 1.00 0.00
H
ATOM 957 HD12 LEU A 230 8.761 25.579 27.941 1.00 0.00
H
ATOM 958 HD13 LEU A 230 10.298 25.810 28.744 1.00 0.00
H
ATOM 959 HD21 LEU A 230 10.722 24.673 30.604 1.00 0.00
H
ATOM 960 HD22 LEU A 230 9.844 23.251 31.120 1.00 0.00
H
ATOM 961 HD23 LEU A 230 11.243 23.087 30.085 1.00 0.00
H
ATOM 962 N ALA A 231 5.254 21.653 29.959 1.00 20.40
N
ATOM 963 CA ALA A 231 4.150 21.139 30.762 1.00 21.50
C
ATOM 964 C ALA A 231 4.273 19.628 30.989 1.00 21.12
C
ATOM 965 O ALA A 231 4.222 19.196 32.138 1.00 21.78
O
ATOM 966 CB ALA A 231 2.808 21.479 30.107 1.00 22.95
C
ATOM 967 H ALA A 231 4.997 22.053 29.059 1.00 0.00
H
ATOM 968 HA ALA A 231 4.182 21.635 31.733 1.00 0.00
H
ATOM 969 HB1 ALA A 231 1.978 21.086 30.689 1.00 0.00
H
ATOM 970 HB2 ALA A 231 2.667 22.558 30.033 1.00 0.00

H

ATOM	971	HB3	ALA	A	231	2.730	21.064	29.101	1.00	0.00	H
ATOM	972	N	ASP	A	232	4.478	18.856	29.911	1.00	20.87	N
ATOM	973	CA	ASP	A	232	4.703	17.404	29.975	1.00	21.48	C
ATOM	974	C	ASP	A	232	5.955	17.049	30.789	1.00	20.43	C
ATOM	975	O	ASP	A	232	5.886	16.179	31.655	1.00	21.59	O
ATOM	976	CB	ASP	A	232	4.741	16.700	28.599	1.00	21.87	C
ATOM	977	CG	ASP	A	232	3.501	16.902	27.727	1.00	25.14	C
ATOM	978	OD1	ASP	A	232	2.460	17.358	28.249	1.00	26.65	O
ATOM	979	OD2	ASP	A	232	3.591	16.555	26.532	1.00	25.49	O1-
ATOM	980	H	ASP	A	232	4.505	19.277	28.989	1.00	0.00	H
ATOM	981	HA	ASP	A	232	3.854	16.986	30.519	1.00	0.00	H
ATOM	982	HB3	ASP	A	232	4.883	15.629	28.744	1.00	0.00	H
ATOM	983	HB2	ASP	A	232	5.600	17.085	28.051	1.00	0.00	H
ATOM	984	N	LEU	A	233	7.042	17.788	30.543	1.00	21.20	N
ATOM	985	CA	LEU	A	233	8.317	17.726	31.255	1.00	20.50	C
ATOM	986	C	LEU	A	233	8.151	17.858	32.786	1.00	20.58	C
ATOM	987	O	LEU	A	233	8.552	16.951	33.522	1.00	18.39	O
ATOM	988	CB	LEU	A	233	9.233	18.806	30.636	1.00	20.92	

C
ATOM 989 CG LEU A 233 10.624 19.008 31.252 1.00 20.96
C
ATOM 990 CD1 LEU A 233 11.503 17.776 31.017 1.00 22.15
C
ATOM 991 CD2 LEU A 233 11.291 20.279 30.699 1.00 22.47
C
ATOM 992 H LEU A 233 6.999 18.452 29.774 1.00 0.00
H
ATOM 993 HA LEU A 233 8.751 16.746 31.055 1.00 0.00
H
ATOM 994 HB3 LEU A 233 8.715 19.749 30.732 1.00 0.00
H
ATOM 995 HB2 LEU A 233 9.338 18.625 29.564 1.00 0.00
H
ATOM 996 HG LEU A 233 10.506 19.174 32.319 1.00 0.00
H
ATOM 997 HD11 LEU A 233 11.350 17.391 30.012 1.00 0.00
H
ATOM 998 HD12 LEU A 233 12.563 18.010 31.098 1.00 0.00
H
ATOM 999 HD13 LEU A 233 11.280 16.977 31.725 1.00 0.00
H
ATOM 1000 HD21 LEU A 233 12.317 20.112 30.370 1.00 0.00
H
ATOM 1001 HD22 LEU A 233 10.751 20.679 29.842 1.00 0.00
H
ATOM 1002 HD23 LEU A 233 11.325 21.070 31.447 1.00 0.00
H
ATOM 1003 N VAL A 234 7.527 18.953 33.253 1.00 20.09
N
ATOM 1004 CA VAL A 234 7.306 19.180 34.683 1.00 20.15
C
ATOM 1005 C VAL A 234 6.243 18.220 35.274 1.00 19.97
C
ATOM 1006 O VAL A 234 6.453 17.706 36.372 1.00 19.99

O
ATOM 1007 CB VAL A 234 6.986 20.671 35.013 1.00 21.02
C
ATOM 1008 CG1 VAL A 234 5.581 21.148 34.610 1.00 23.20
C
ATOM 1009 CG2 VAL A 234 7.238 21.018 36.491 1.00 21.83
C
ATOM 1010 H VAL A 234 7.210 19.674 32.609 1.00 0.00
H
ATOM 1011 HA VAL A 234 8.246 18.941 35.186 1.00 0.00
H
ATOM 1012 HB VAL A 234 7.684 21.276 34.432 1.00 0.00
H
ATOM 1013 HG11 VAL A 234 5.470 22.221 34.773 1.00 0.00
H
ATOM 1014 HG12 VAL A 234 5.396 20.967 33.556 1.00 0.00
H
ATOM 1015 HG13 VAL A 234 4.800 20.651 35.183 1.00 0.00
H
ATOM 1016 HG21 VAL A 234 7.064 22.078 36.684 1.00 0.00
H
ATOM 1017 HG22 VAL A 234 6.588 20.452 37.160 1.00 0.00
H
ATOM 1018 HG23 VAL A 234 8.270 20.810 36.770 1.00 0.00
H
ATOM 1019 N SER A 235 5.178 17.913 34.515 1.00 18.23
N
ATOM 1020 CA SER A 235 4.129 16.947 34.870 1.00 19.68
C
ATOM 1021 C SER A 235 4.676 15.525 35.088 1.00 19.46
C
ATOM 1022 O SER A 235 4.167 14.784 35.929 1.00 19.95
O
ATOM 1023 CB SER A 235 3.053 16.950 33.762 1.00 21.15
C
ATOM 1024 OG SER A 235 1.910 16.160 34.049 1.00 26.38

O
ATOM 1025 H SER A 235 5.070 18.373 33.612 1.00 0.00
H
ATOM 1026 HA SER A 235 3.674 17.279 35.804 1.00 0.00
H
ATOM 1027 HB3 SER A 235 3.481 16.606 32.820 1.00 0.00
H
ATOM 1028 HB2 SER A 235 2.701 17.963 33.592 1.00 0.00
H
ATOM 1029 HG SER A 235 2.202 15.251 34.193 1.00 0.00
H
ATOM 1030 N TYR A 236 5.696 15.160 34.309 1.00 19.05
N
ATOM 1031 CA TYR A 236 6.406 13.901 34.436 1.00 19.13
C
ATOM 1032 C TYR A 236 7.350 13.923 35.650 1.00 18.86
C
ATOM 1033 O TYR A 236 7.398 12.954 36.406 1.00 18.39
O
ATOM 1034 CB TYR A 236 7.121 13.627 33.103 1.00 19.07
C
ATOM 1035 CG TYR A 236 7.852 12.307 33.012 1.00 19.18
C
ATOM 1036 CD1 TYR A 236 7.137 11.095 33.109 1.00 20.38
C
ATOM 1037 CD2 TYR A 236 9.242 12.289 32.799 1.00 20.55
C
ATOM 1038 CE1 TYR A 236 7.815 9.868 33.005 1.00 21.48
C
ATOM 1039 CE2 TYR A 236 9.910 11.062 32.656 1.00 20.66
C
ATOM 1040 CZ TYR A 236 9.203 9.849 32.781 1.00 21.96
C
ATOM 1041 OH TYR A 236 9.853 8.654 32.711 1.00 21.78
O
ATOM 1042 H TYR A 236 6.002 15.776 33.560 1.00 0.00

H

ATOM	1043	HA	TYR	A	236	5.676	13.105	34.602	1.00	0.00
H										
ATOM	1044	HB3	TYR	A	236	7.814	14.439	32.877	1.00	0.00
H										
ATOM	1045	HB2	TYR	A	236	6.388	13.635	32.295	1.00	0.00
H										
ATOM	1046	HD1	TYR	A	236	6.068	11.097	33.267	1.00	0.00
H										
ATOM	1047	HD2	TYR	A	236	9.799	13.212	32.731	1.00	0.00
H										
ATOM	1048	HE1	TYR	A	236	7.267	8.940	33.090	1.00	0.00
H										
ATOM	1049	HE2	TYR	A	236	10.969	11.075	32.460	1.00	0.00
H										
ATOM	1050	HH	TYR	A	236	10.761	8.722	32.380	1.00	0.00
H										
ATOM	1051	N	SER	A	237	8.035	15.052	35.876	1.00	17.93
N										
ATOM	1052	CA	SER	A	237	8.938	15.203	37.013	1.00	18.19
C										
ATOM	1053	C	SER	A	237	8.223	15.252	38.373	1.00	18.73
C										
ATOM	1054	O	SER	A	237	8.808	14.804	39.358	1.00	19.34
O										
ATOM	1055	CB	SER	A	237	9.833	16.427	36.841	1.00	18.72
C										
ATOM	1056	OG	SER	A	237	10.928	16.316	37.739	1.00	19.76
O										
ATOM	1057	H	SER	A	237	7.969	15.826	35.223	1.00	0.00
H										
ATOM	1058	HA	SER	A	237	9.582	14.321	37.010	1.00	0.00
H										
ATOM	1059	HB3	SER	A	237	9.278	17.346	37.035	1.00	0.00
H										
ATOM	1060	HB2	SER	A	237	10.190	16.490	35.815	1.00	0.00

H
ATOM 1061 HG SER A 237 10.580 16.259 38.638 1.00 0.00
H
ATOM 1062 N ILE A 238 6.977 15.744 38.417 1.00 18.89
N
ATOM 1063 CA ILE A 238 6.098 15.657 39.587 1.00 20.25
C
ATOM 1064 C ILE A 238 5.984 14.203 40.101 1.00 20.17
C
ATOM 1065 O ILE A 238 6.102 13.964 41.301 1.00 20.62
O
ATOM 1066 CB ILE A 238 4.687 16.242 39.259 1.00 21.21
C
ATOM 1067 CG1 ILE A 238 4.723 17.788 39.216 1.00 23.61
C
ATOM 1068 CG2 ILE A 238 3.525 15.777 40.164 1.00 23.76
C
ATOM 1069 CD1 ILE A 238 3.559 18.426 38.434 1.00 24.70
C
ATOM 1070 H ILE A 238 6.600 16.207 37.595 1.00 0.00
H
ATOM 1071 HA ILE A 238 6.551 16.249 40.385 1.00 0.00
H
ATOM 1072 HB ILE A 238 4.441 15.903 38.253 1.00 0.00
H
ATOM 1073 HG13 ILE A 238 5.659 18.129 38.773 1.00 0.00
H
ATOM 1074 HG12 ILE A 238 4.735 18.180 40.234 1.00 0.00
H
ATOM 1075 HG21 ILE A 238 2.584 16.202 39.829 1.00 0.00
H
ATOM 1076 HG22 ILE A 238 3.374 14.698 40.136 1.00 0.00
H
ATOM 1077 HG23 ILE A 238 3.682 16.073 41.202 1.00 0.00
H
ATOM 1078 HD11 ILE A 238 3.933 18.985 37.575 1.00 0.00

H
ATOM 1079 HD12 ILE A 238 2.851 17.689 38.057 1.00 0.00
H
ATOM 1080 HD13 ILE A 238 3.002 19.122 39.061 1.00 0.00
H
ATOM 1081 N GLN A 239 5.853 13.248 39.171 1.00 20.04
N
ATOM 1082 CA GLN A 239 5.767 11.817 39.475 1.00 19.72
C
ATOM 1083 C GLN A 239 7.097 11.241 40.000 1.00 20.24
C
ATOM 1084 O GLN A 239 7.063 10.323 40.819 1.00 19.92
O
ATOM 1085 CB GLN A 239 5.292 11.046 38.228 1.00 21.35
C
ATOM 1086 CG GLN A 239 3.914 11.499 37.704 1.00 21.87
C
ATOM 1087 CD GLN A 239 3.599 10.918 36.324 1.00 23.58
C
ATOM 1088 OE1 GLN A 239 3.814 9.734 36.075 1.00 26.39
O
ATOM 1089 NE2 GLN A 239 3.069 11.747 35.423 1.00 22.31
N
ATOM 1090 H GLN A 239 5.833 13.493 38.191 1.00 0.00
H
ATOM 1091 HA GLN A 239 5.017 11.686 40.257 1.00 0.00
H
ATOM 1092 HB3 GLN A 239 5.251 9.979 38.454 1.00 0.00
H
ATOM 1093 HB2 GLN A 239 6.032 11.146 37.435 1.00 0.00
H
ATOM 1094 HG3 GLN A 239 3.869 12.586 37.638 1.00 0.00
H
ATOM 1095 HG2 GLN A 239 3.131 11.202 38.402 1.00 0.00
H
ATOM 1096 HE22 GLN A 239 2.830 11.408 34.503 1.00 0.00

H
ATOM 1097 HE21 GLN A 239 2.915 12.718 35.655 1.00 0.00
H
ATOM 1098 N LYS A 240 8.221 11.812 39.562 1.00 19.37
N
ATOM 1099 CA LYS A 240 9.557 11.468 40.035 1.00 19.49
C
ATOM 1100 C LYS A 240 9.818 12.003 41.454 1.00 20.03
C
ATOM 1101 O LYS A 240 10.321 11.253 42.291 1.00 20.81
O
ATOM 1102 CB LYS A 240 10.589 11.932 38.990 1.00 19.27
C
ATOM 1103 CG LYS A 240 10.496 11.087 37.706 1.00 19.70
C
ATOM 1104 CD LYS A 240 11.394 11.569 36.557 1.00 20.85
C
ATOM 1105 CE LYS A 240 11.753 10.413 35.611 1.00 20.78
C
ATOM 1106 NZ LYS A 240 12.625 10.858 34.518 1.00 21.79
N1+
ATOM 1107 H LYS A 240 8.150 12.583 38.912 1.00 0.00
H
ATOM 1108 HA LYS A 240 9.618 10.379 40.101 1.00 0.00
H
ATOM 1109 HB3 LYS A 240 11.593 11.842 39.402 1.00 0.00
H
ATOM 1110 HB2 LYS A 240 10.466 12.990 38.765 1.00 0.00
H
ATOM 1111 HG3 LYS A 240 9.467 11.050 37.345 1.00 0.00
H
ATOM 1112 HG2 LYS A 240 10.757 10.059 37.964 1.00 0.00
H
ATOM 1113 HD3 LYS A 240 12.296 12.049 36.938 1.00 0.00
H
ATOM 1114 HD2 LYS A 240 10.864 12.339 35.994 1.00 0.00

H
ATOM 1115 HE3 LYS A 240 10.848 9.976 35.189 1.00 0.00
H
ATOM 1116 HE2 LYS A 240 12.269 9.620 36.154 1.00 0.00
H
ATOM 1117 HZ1 LYS A 240 12.170 11.601 34.010 1.00 0.00
H
ATOM 1118 HZ2 LYS A 240 13.499 11.199 34.894 1.00 0.00
H
ATOM 1119 HZ3 LYS A 240 12.800 10.092 33.878 1.00 0.00
H
ATOM 1120 N VAL A 241 9.393 13.244 41.731 1.00 19.95
N
ATOM 1121 CA VAL A 241 9.421 13.849 43.066 1.00 21.18
C
ATOM 1122 C VAL A 241 8.518 13.088 44.067 1.00 22.58
C
ATOM 1123 O VAL A 241 8.948 12.832 45.192 1.00 22.01
O
ATOM 1124 CB VAL A 241 9.035 15.361 43.018 1.00 22.53
C
ATOM 1125 CG1 VAL A 241 8.790 16.031 44.387 1.00 23.49
C
ATOM 1126 CG2 VAL A 241 10.096 16.175 42.254 1.00 22.43
C
ATOM 1127 H VAL A 241 8.994 13.807 40.985 1.00 0.00
H
ATOM 1128 HA VAL A 241 10.445 13.773 43.437 1.00 0.00
H
ATOM 1129 HB VAL A 241 8.101 15.451 42.460 1.00 0.00
H
ATOM 1130 HG11 VAL A 241 8.620 17.103 44.283 1.00 0.00
H
ATOM 1131 HG12 VAL A 241 7.912 15.627 44.886 1.00 0.00
H
ATOM 1132 HG13 VAL A 241 9.642 15.899 45.055 1.00 0.00

H
ATOM 1133 HG21 VAL A 241 9.828 17.232 42.220 1.00 0.00
H
ATOM 1134 HG22 VAL A 241 11.071 16.098 42.733 1.00 0.00
H
ATOM 1135 HG23 VAL A 241 10.212 15.836 41.226 1.00 0.00
H
ATOM 1136 N ILE A 242 7.326 12.667 43.621 1.00 22.44
N
ATOM 1137 CA ILE A 242 6.394 11.841 44.394 1.00 23.64
C
ATOM 1138 C ILE A 242 6.965 10.451 44.735 1.00 23.45
C
ATOM 1139 O ILE A 242 6.814 10.016 45.876 1.00 25.50
O
ATOM 1140 CB ILE A 242 5.001 11.728 43.699 1.00 24.32
C
ATOM 1141 CG1 ILE A 242 4.245 13.075 43.802 1.00 25.64
C
ATOM 1142 CG2 ILE A 242 4.090 10.581 44.201 1.00 24.95
C
ATOM 1143 CD1 ILE A 242 3.096 13.231 42.794 1.00 25.41
C
ATOM 1144 H ILE A 242 7.065 12.844 42.659 1.00 0.00
H
ATOM 1145 HA ILE A 242 6.237 12.339 45.345 1.00 0.00
H
ATOM 1146 HB ILE A 242 5.188 11.540 42.642 1.00 0.00
H
ATOM 1147 HG13 ILE A 242 4.932 13.910 43.665 1.00 0.00
H
ATOM 1148 HG12 ILE A 242 3.850 13.195 44.810 1.00 0.00
H
ATOM 1149 HG21 ILE A 242 3.120 10.598 43.705 1.00 0.00
H
ATOM 1150 HG22 ILE A 242 4.513 9.598 44.000 1.00 0.00

H
ATOM 1151 HG23 ILE A 242 3.912 10.660 45.274 1.00 0.00
H
ATOM 1152 HD11 ILE A 242 2.827 14.281 42.679 1.00 0.00
H
ATOM 1153 HD12 ILE A 242 3.369 12.852 41.810 1.00 0.00
H
ATOM 1154 HD13 ILE A 242 2.203 12.702 43.125 1.00 0.00
H
ATOM 1155 N GLY A 243 7.641 9.806 43.769 1.00 22.76
N
ATOM 1156 CA GLY A 243 8.297 8.510 43.958 1.00 23.14
C
ATOM 1157 C GLY A 243 9.496 8.629 44.911 1.00 23.29
C
ATOM 1158 O GLY A 243 9.672 7.770 45.774 1.00 24.26
O
ATOM 1159 H GLY A 243 7.709 10.223 42.850 1.00 0.00
H
ATOM 1160 HA3 GLY A 243 8.646 8.146 42.992 1.00 0.00
H
ATOM 1161 HA2 GLY A 243 7.583 7.780 44.341 1.00 0.00
H
ATOM 1162 N PHE A 244 10.282 9.713 44.780 1.00 22.21
N
ATOM 1163 CA PHE A 244 11.422 10.052 45.632 1.00 23.08
C
ATOM 1164 C PHE A 244 11.015 10.311 47.094 1.00 23.33
C
ATOM 1165 O PHE A 244 11.688 9.817 47.998 1.00 22.96
O
ATOM 1166 CB PHE A 244 12.182 11.243 45.005 1.00 21.55
C
ATOM 1167 CG PHE A 244 13.407 11.731 45.761 1.00 23.20
C
ATOM 1168 CD1 PHE A 244 14.652 11.101 45.561 1.00 25.04

C

ATOM 1169 CD2 PHE A 244 13.285 12.697 46.782 1.00 24.78

C

ATOM 1170 CE1 PHE A 244 15.749 11.471 46.326 1.00 25.46

C

ATOM 1171 CE2 PHE A 244 14.391 13.050 47.542 1.00 24.71

C

ATOM 1172 CZ PHE A 244 15.618 12.441 47.310 1.00 24.22

C

ATOM 1173 H PHE A 244 10.084 10.372 44.038 1.00 0.00

H

ATOM 1174 HA PHE A 244 12.096 9.193 45.629 1.00 0.00

H

ATOM 1175 HB3 PHE A 244 11.502 12.085 44.870 1.00 0.00

H

ATOM 1176 HB2 PHE A 244 12.510 10.962 44.003 1.00 0.00

H

ATOM 1177 HD1 PHE A 244 14.754 10.334 44.810 1.00 0.00

H

ATOM 1178 HD2 PHE A 244 12.327 13.154 46.982 1.00 0.00

H

ATOM 1179 HE1 PHE A 244 16.704 10.996 46.165 1.00 0.00

H

ATOM 1180 HE2 PHE A 244 14.296 13.791 48.321 1.00 0.00

H

ATOM 1181 HZ PHE A 244 16.474 12.712 47.905 1.00 0.00

H

ATOM 1182 N ALA A 245 9.903 11.046 47.284 1.00 23.25

N

ATOM 1183 CA ALA A 245 9.305 11.353 48.583 1.00 23.37

C

ATOM 1184 C ALA A 245 8.826 10.111 49.339 1.00 25.09

C

ATOM 1185 O ALA A 245 9.099 9.994 50.530 1.00 24.87

O

ATOM 1186 CB ALA A 245 8.144 12.332 48.400 1.00 22.36

C
ATOM 1187 H ALA A 245 9.421 11.426 46.480 1.00 0.00
H
ATOM 1188 HA ALA A 245 10.069 11.844 49.181 1.00 0.00
H
ATOM 1189 HB1 ALA A 245 7.712 12.619 49.359 1.00 0.00
H
ATOM 1190 HB2 ALA A 245 8.484 13.242 47.908 1.00 0.00
H
ATOM 1191 HB3 ALA A 245 7.346 11.903 47.793 1.00 0.00
H
ATOM 1192 N LYS A 246 8.170 9.186 48.619 1.00 26.15
N
ATOM 1193 CA LYS A 246 7.703 7.892 49.126 1.00 28.66
C
ATOM 1194 C LYS A 246 8.831 6.913 49.516 1.00 29.21
C
ATOM 1195 O LYS A 246 8.530 5.884 50.120 1.00 29.75
O
ATOM 1196 CB LYS A 246 6.729 7.282 48.098 1.00 30.93
C
ATOM 1197 CG LYS A 246 5.385 8.037 48.031 1.00 34.75
C
ATOM 1198 CD LYS A 246 4.501 7.632 46.837 1.00 37.28
C
ATOM 1199 CE LYS A 246 3.984 6.185 46.873 1.00 40.02
C
ATOM 1200 NZ LYS A 246 3.099 5.948 48.027 1.00 42.15
N1+
ATOM 1201 H LYS A 246 7.990 9.366 47.641 1.00 0.00
H
ATOM 1202 HA LYS A 246 7.142 8.085 50.040 1.00 0.00
H
ATOM 1203 HB3 LYS A 246 6.522 6.242 48.353 1.00 0.00
H
ATOM 1204 HB2 LYS A 246 7.202 7.265 47.115 1.00 0.00

H
ATOM 1205 HG3 LYS A 246 5.564 9.110 47.974 1.00 0.00
H
ATOM 1206 HG2 LYS A 246 4.842 7.892 48.965 1.00 0.00
H
ATOM 1207 HD3 LYS A 246 5.064 7.780 45.914 1.00 0.00
H
ATOM 1208 HD2 LYS A 246 3.656 8.319 46.771 1.00 0.00
H
ATOM 1209 HE3 LYS A 246 4.812 5.477 46.900 1.00 0.00
H
ATOM 1210 HE2 LYS A 246 3.422 5.974 45.963 1.00 0.00
H
ATOM 1211 HZ1 LYS A 246 3.608 6.116 48.883 1.00 0.00
H
ATOM 1212 HZ2 LYS A 246 2.309 6.575 47.981 1.00 0.00
H
ATOM 1213 HZ3 LYS A 246 2.771 4.993 48.013 1.00 0.00
H
ATOM 1214 N MET A 247 10.092 7.254 49.201 1.00 28.68
N
ATOM 1215 CA MET A 247 11.295 6.523 49.596 1.00 29.45
C
ATOM 1216 C MET A 247 12.145 7.279 50.642 1.00 28.75
C
ATOM 1217 O MET A 247 13.201 6.765 51.001 1.00 28.61
O
ATOM 1218 CB MET A 247 12.104 6.154 48.332 1.00 30.97
C
ATOM 1219 CG MET A 247 11.408 5.104 47.450 1.00 34.76
C
ATOM 1220 SD MET A 247 12.397 4.510 46.048 1.00 39.72
S
ATOM 1221 CE MET A 247 12.066 5.811 44.832 1.00 39.07
C
ATOM 1222 H MET A 247 10.254 8.115 48.696 1.00 0.00

H
ATOM 1223 HA MET A 247 11.019 5.589 50.088 1.00 0.00
H
ATOM 1224 HB3 MET A 247 13.080 5.758 48.614 1.00 0.00
H
ATOM 1225 HB2 MET A 247 12.298 7.053 47.746 1.00 0.00
H
ATOM 1226 HG3 MET A 247 10.459 5.484 47.072 1.00 0.00
H
ATOM 1227 HG2 MET A 247 11.167 4.233 48.060 1.00 0.00
H
ATOM 1228 HE1 MET A 247 12.652 5.639 43.930 1.00 0.00
H
ATOM 1229 HE2 MET A 247 11.013 5.812 44.555 1.00 0.00
H
ATOM 1230 HE3 MET A 247 12.328 6.790 45.230 1.00 0.00
H
ATOM 1231 N ILE A 248 11.684 8.436 51.161 1.00 27.31
N
ATOM 1232 CA ILE A 248 12.308 9.122 52.304 1.00 28.07
C
ATOM 1233 C ILE A 248 12.040 8.323 53.608 1.00 28.99
C
ATOM 1234 O ILE A 248 10.875 8.005 53.859 1.00 28.97
O
ATOM 1235 CB ILE A 248 11.745 10.569 52.491 1.00 27.49
C
ATOM 1236 CG1 ILE A 248 12.172 11.473 51.313 1.00 26.51
C
ATOM 1237 CG2 ILE A 248 12.133 11.242 53.834 1.00 28.63
C
ATOM 1238 CD1 ILE A 248 11.469 12.836 51.279 1.00 25.47
C
ATOM 1239 H ILE A 248 10.809 8.822 50.834 1.00 0.00
H
ATOM 1240 HA ILE A 248 13.366 9.220 52.081 1.00 0.00

H
ATOM 1241 HB ILE A 248 10.659 10.507 52.469 1.00 0.00
H
ATOM 1242 HG13 ILE A 248 11.986 10.969 50.366 1.00 0.00
H
ATOM 1243 HG12 ILE A 248 13.247 11.637 51.350 1.00 0.00
H
ATOM 1244 HG21 ILE A 248 11.754 12.260 53.904 1.00 0.00
H
ATOM 1245 HG22 ILE A 248 11.722 10.724 54.701 1.00 0.00
H
ATOM 1246 HG23 ILE A 248 13.216 11.285 53.952 1.00 0.00
H
ATOM 1247 HD11 ILE A 248 11.605 13.304 50.306 1.00 0.00
H
ATOM 1248 HD12 ILE A 248 10.397 12.743 51.462 1.00 0.00
H
ATOM 1249 HD13 ILE A 248 11.882 13.518 52.021 1.00 0.00
H
ATOM 1250 N PRO A 249 13.092 8.020 54.412 1.00 30.64
N
ATOM 1251 CA PRO A 249 12.956 7.382 55.741 1.00 31.91
C
ATOM 1252 C PRO A 249 11.934 8.034 56.700 1.00 32.16
C
ATOM 1253 O PRO A 249 12.166 9.139 57.195 1.00 34.02
O
ATOM 1254 CB PRO A 249 14.389 7.410 56.308 1.00 32.36
C
ATOM 1255 CG PRO A 249 15.275 7.366 55.079 1.00 32.23
C
ATOM 1256 CD PRO A 249 14.508 8.220 54.081 1.00 30.61
C
ATOM 1257 HA PRO A 249 12.674 6.343 55.559 1.00 0.00
H
ATOM 1258 HB3 PRO A 249 14.588 6.581 56.988 1.00 0.00

H
ATOM 1259 HB2 PRO A 249 14.586 8.336 56.851 1.00 0.00
H
ATOM 1260 HG3 PRO A 249 15.334 6.340 54.715 1.00 0.00
H
ATOM 1261 HG2 PRO A 249 16.290 7.716 55.255 1.00 0.00
H
ATOM 1262 HD2 PRO A 249 14.758 9.275 54.203 1.00 0.00
H
ATOM 1263 HD3 PRO A 249 14.765 7.917 53.067 1.00 0.00
H
ATOM 1264 N GLY A 250 10.824 7.316 56.938 1.00 32.30
N
ATOM 1265 CA GLY A 250 9.771 7.686 57.885 1.00 33.31
C
ATOM 1266 C GLY A 250 8.641 8.522 57.260 1.00 33.48
C
ATOM 1267 O GLY A 250 7.712 8.880 57.982 1.00 33.45
O
ATOM 1268 H GLY A 250 10.713 6.426 56.474 1.00 0.00
H
ATOM 1269 HA3 GLY A 250 10.182 8.220 58.742 1.00 0.00
H
ATOM 1270 HA2 GLY A 250 9.337 6.766 58.276 1.00 0.00
H
ATOM 1271 N PHE A 251 8.684 8.824 55.949 1.00 33.51
N
ATOM 1272 CA PHE A 251 7.637 9.568 55.234 1.00 33.69
C
ATOM 1273 C PHE A 251 6.305 8.805 55.103 1.00 34.57
C
ATOM 1274 O PHE A 251 5.254 9.440 55.056 1.00 35.10
O
ATOM 1275 CB PHE A 251 8.189 10.026 53.870 1.00 31.35
C
ATOM 1276 CG PHE A 251 7.262 10.829 52.969 1.00 29.89

C
ATOM 1277 CD1 PHE A 251 7.160 12.225 53.132 1.00 30.12
C
ATOM 1278 CD2 PHE A 251 6.414 10.187 52.040 1.00 30.05
C
ATOM 1279 CE1 PHE A 251 6.322 12.962 52.307 1.00 29.28
C
ATOM 1280 CE2 PHE A 251 5.581 10.941 51.224 1.00 28.78
C
ATOM 1281 CZ PHE A 251 5.546 12.324 51.350 1.00 28.48
C
ATOM 1282 H PHE A 251 9.471 8.515 55.393 1.00 0.00
H
ATOM 1283 HA PHE A 251 7.423 10.465 55.816 1.00 0.00
H
ATOM 1284 HB3 PHE A 251 8.544 9.163 53.306 1.00 0.00
H
ATOM 1285 HB2 PHE A 251 9.066 10.646 54.057 1.00 0.00
H
ATOM 1286 HD1 PHE A 251 7.748 12.719 53.888 1.00 0.00
H
ATOM 1287 HD2 PHE A 251 6.430 9.112 51.946 1.00 0.00
H
ATOM 1288 HE1 PHE A 251 6.274 14.036 52.407 1.00 0.00
H
ATOM 1289 HE2 PHE A 251 4.953 10.450 50.495 1.00 0.00
H
ATOM 1290 HZ PHE A 251 4.897 12.908 50.718 1.00 0.00
H
ATOM 1291 N ARG A 252 6.372 7.463 55.077 1.00 36.83
N
ATOM 1292 CA ARG A 252 5.213 6.566 55.045 1.00 39.19
C
ATOM 1293 C ARG A 252 4.430 6.523 56.373 1.00 39.61
C
ATOM 1294 O ARG A 252 3.262 6.138 56.352 1.00 40.31

O

ATOM 1295 CB ARG A 252 5.673 5.152 54.639 1.00 41.06

C

ATOM 1296 CG ARG A 252 6.358 5.095 53.262 1.00 44.96

C

ATOM 1297 CD ARG A 252 6.658 3.659 52.815 1.00 47.63

C

ATOM 1298 NE ARG A 252 7.472 3.640 51.594 1.00 50.79

N

ATOM 1299 CZ ARG A 252 7.786 2.558 50.863 1.00 51.79

C

ATOM 1300 NH1 ARG A 252 7.363 1.334 51.208 1.00 52.82

N

ATOM 1301 NH2 ARG A 252 8.538 2.716 49.767 1.00 52.64

N1+

ATOM 1302 H ARG A 252 7.276 7.017 55.130 1.00 0.00

H

ATOM 1303 HA ARG A 252 4.527 6.934 54.279 1.00 0.00

H

ATOM 1304 HB3 ARG A 252 4.810 4.484 54.627 1.00 0.00

H

ATOM 1305 HB2 ARG A 252 6.352 4.757 55.397 1.00 0.00

H

ATOM 1306 HG3 ARG A 252 7.249 5.722 53.200 1.00 0.00

H

ATOM 1307 HG2 ARG A 252 5.643 5.516 52.553 1.00 0.00

H

ATOM 1308 HD3 ARG A 252 5.713 3.204 52.514 1.00 0.00

H

ATOM 1309 HD2 ARG A 252 7.063 3.042 53.617 1.00 0.00

H

ATOM 1310 HE ARG A 252 7.834 4.536 51.287 1.00 0.00

H

ATOM 1311 HH12 ARG A 252 7.608 0.526 50.652 1.00 0.00

H

ATOM 1312 HH11 ARG A 252 6.799 1.211 52.036 1.00 0.00

H
ATOM 1313 HH22 ARG A 252 8.798 1.923 49.199 1.00 0.00
H
ATOM 1314 HH21 ARG A 252 8.856 3.640 49.506 1.00 0.00
H
ATOM 1315 N ASP A 253 5.071 6.932 57.483 1.00 39.96
N
ATOM 1316 CA ASP A 253 4.483 7.023 58.827 1.00 40.45
C
ATOM 1317 C ASP A 253 3.475 8.183 58.965 1.00 39.90
C
ATOM 1318 O ASP A 253 2.627 8.129 59.855 1.00 39.68
O
ATOM 1319 CB ASP A 253 5.546 7.111 59.954 1.00 42.53
C
ATOM 1320 CG ASP A 253 6.701 6.102 59.868 1.00 44.84
C
ATOM 1321 OD1 ASP A 253 6.516 5.035 59.242 1.00 47.60
O
ATOM 1322 OD2 ASP A 253 7.745 6.396 60.489 1.00 45.66
O1-
ATOM 1323 H ASP A 253 6.036 7.224 57.414 1.00 0.00
H
ATOM 1324 HA ASP A 253 3.918 6.103 58.991 1.00 0.00
H
ATOM 1325 HB3 ASP A 253 5.063 6.989 60.924 1.00 0.00
H
ATOM 1326 HB2 ASP A 253 5.986 8.107 59.931 1.00 0.00
H
ATOM 1327 N LEU A 254 3.570 9.191 58.078 1.00 38.00
N
ATOM 1328 CA LEU A 254 2.626 10.303 57.941 1.00 37.81
C
ATOM 1329 C LEU A 254 1.257 9.835 57.418 1.00 37.76
C
ATOM 1330 O LEU A 254 1.170 8.796 56.761 1.00 37.49

O
ATOM 1331 CB LEU A 254 3.208 11.326 56.938 1.00 36.51
C
ATOM 1332 CG LEU A 254 4.524 12.005 57.366 1.00 36.86
C
ATOM 1333 CD1 LEU A 254 5.157 12.782 56.195 1.00 35.66
C
ATOM 1334 CD2 LEU A 254 4.310 12.912 58.585 1.00 36.85
C
ATOM 1335 H LEU A 254 4.298 9.151 57.379 1.00 0.00
H
ATOM 1336 HA LEU A 254 2.480 10.767 58.918 1.00 0.00
H
ATOM 1337 HB3 LEU A 254 2.473 12.105 56.728 1.00 0.00
H
ATOM 1338 HB2 LEU A 254 3.364 10.816 55.990 1.00 0.00
H
ATOM 1339 HG LEU A 254 5.238 11.231 57.651 1.00 0.00
H
ATOM 1340 HD11 LEU A 254 6.155 12.408 55.974 1.00 0.00
H
ATOM 1341 HD12 LEU A 254 4.571 12.702 55.278 1.00 0.00
H
ATOM 1342 HD13 LEU A 254 5.257 13.843 56.415 1.00 0.00
H
ATOM 1343 HD21 LEU A 254 5.209 13.486 58.806 1.00 0.00
H
ATOM 1344 HD22 LEU A 254 3.489 13.613 58.424 1.00 0.00
H
ATOM 1345 HD23 LEU A 254 4.075 12.329 59.474 1.00 0.00
H
ATOM 1346 N THR A 255 0.224 10.659 57.656 1.00 38.14
N
ATOM 1347 CA THR A 255 -1.089 10.524 57.018 1.00 39.08
C
ATOM 1348 C THR A 255 -0.995 10.819 55.504 1.00 39.76

C
ATOM 1349 O THR A 255 -0.149 11.615 55.090 1.00 39.15
O
ATOM 1350 CB THR A 255 -2.139 11.483 57.657 1.00 39.68
C
ATOM 1351 OG1 THR A 255 -2.073 12.817 57.186 1.00 39.47
O
ATOM 1352 CG2 THR A 255 -2.154 11.478 59.194 1.00 40.24
C
ATOM 1353 H THR A 255 0.368 11.505 58.190 1.00 0.00
H
ATOM 1354 HA THR A 255 -1.430 9.496 57.155 1.00 0.00
H
ATOM 1355 HB THR A 255 -3.120 11.123 57.342 1.00 0.00
H
ATOM 1356 HG1 THR A 255 -2.521 13.395 57.810 1.00 0.00
H
ATOM 1357 HG21 THR A 255 -2.983 12.072 59.581 1.00 0.00
H
ATOM 1358 HG22 THR A 255 -2.266 10.464 59.580 1.00 0.00
H
ATOM 1359 HG23 THR A 255 -1.234 11.894 59.608 1.00 0.00
H
ATOM 1360 N SER A 256 -1.876 10.189 54.709 1.00 40.24
N
ATOM 1361 CA SER A 256 -1.965 10.387 53.257 1.00 40.92
C
ATOM 1362 C SER A 256 -2.297 11.835 52.835 1.00 40.23
C
ATOM 1363 O SER A 256 -1.873 12.249 51.756 1.00 40.28
O
ATOM 1364 CB SER A 256 -2.944 9.355 52.663 1.00 41.53
C
ATOM 1365 OG SER A 256 -4.284 9.611 53.041 1.00 43.52
O
ATOM 1366 H SER A 256 -2.553 9.555 55.109 1.00 0.00

										H
ATOM	1367	HA	SER	A	256	-0.975	10.162	52.854	1.00	0.00 H
ATOM	1368	HB3	SER	A	256	-2.672	8.345	52.971	1.00	0.00 H
ATOM	1369	HB2	SER	A	256	-2.888	9.373	51.574	1.00	0.00 H
ATOM	1370	HG	SER	A	256	-4.851	8.957	52.625	1.00	0.00 H
ATOM	1371	N	GLU	A	257	-2.988	12.585	53.714	1.00	39.55 N
ATOM	1372	CA	GLU	A	257	-3.231	14.020	53.592	1.00	38.71 C
ATOM	1373	C	GLU	A	257	-1.927	14.831	53.673	1.00	36.50 C
ATOM	1374	O	GLU	A	257	-1.646	15.586	52.744	1.00	36.20 O
ATOM	1375	CB	GLU	A	257	-4.282	14.451	54.638	1.00	41.43 C
ATOM	1376	CG	GLU	A	257	-4.581	15.967	54.677	1.00	44.63 C
ATOM	1377	CD	GLU	A	257	-5.675	16.362	55.673	1.00	47.39 C
ATOM	1378	OE1	GLU	A	257	-6.225	15.464	56.349	1.00	48.76 O
ATOM	1379	OE2	GLU	A	257	-5.940	17.581	55.752	1.00	48.99 O1-
ATOM	1380	H	GLU	A	257	-3.296	12.161	54.577	1.00	0.00 H
ATOM	1381	HA	GLU	A	257	-3.661	14.195	52.604	1.00	0.00 H
ATOM	1382	HB3	GLU	A	257	-3.963	14.126	55.629	1.00	0.00 H
ATOM	1383	HB2	GLU	A	257	-5.208	13.912	54.432	1.00	0.00 H
ATOM	1384	HG3	GLU	A	257	-4.882	16.310	53.686	1.00	0.00

H
ATOM 1385 HG2 GLU A 257 -3.686 16.528 54.945 1.00 0.00
H
ATOM 1386 N ASP A 258 -1.147 14.635 54.753 1.00 34.59
N
ATOM 1387 CA ASP A 258 0.137 15.311 54.994 1.00 33.04
C
ATOM 1388 C ASP A 258 1.187 15.029 53.912 1.00 32.48
C
ATOM 1389 O ASP A 258 1.874 15.966 53.513 1.00 31.37
O
ATOM 1390 CB ASP A 258 0.749 15.063 56.395 1.00 34.07
C
ATOM 1391 CG ASP A 258 -0.142 15.433 57.585 1.00 35.11
C
ATOM 1392 OD1 ASP A 258 0.325 15.210 58.722 1.00 35.63
O
ATOM 1393 OD2 ASP A 258 -1.253 15.962 57.362 1.00 34.86
O1-
ATOM 1394 H ASP A 258 -1.452 13.999 55.479 1.00 0.00
H
ATOM 1395 HA ASP A 258 -0.080 16.380 54.936 1.00 0.00
H
ATOM 1396 HB3 ASP A 258 1.673 15.636 56.484 1.00 0.00
H
ATOM 1397 HB2 ASP A 258 0.997 14.005 56.486 1.00 0.00
H
ATOM 1398 N GLN A 259 1.259 13.777 53.422 1.00 31.90
N
ATOM 1399 CA GLN A 259 2.127 13.371 52.312 1.00 32.81
C
ATOM 1400 C GLN A 259 1.869 14.166 51.018 1.00 32.59
C
ATOM 1401 O GLN A 259 2.824 14.653 50.416 1.00 32.51
O
ATOM 1402 CB GLN A 259 1.995 11.855 52.052 1.00 32.98

C
ATOM 1403 CG GLN A 259 2.591 10.977 53.170 1.00 34.94
C
ATOM 1404 CD GLN A 259 2.459 9.479 52.880 1.00 37.19
C
ATOM 1405 OE1 GLN A 259 2.657 9.033 51.751 1.00 39.55
O
ATOM 1406 NE2 GLN A 259 2.151 8.683 53.906 1.00 37.81
N
ATOM 1407 H GLN A 259 0.661 13.061 53.812 1.00 0.00
H
ATOM 1408 HA GLN A 259 3.154 13.578 52.617 1.00 0.00
H
ATOM 1409 HB3 GLN A 259 2.490 11.610 51.111 1.00 0.00
H
ATOM 1410 HB2 GLN A 259 0.943 11.601 51.912 1.00 0.00
H
ATOM 1411 HG3 GLN A 259 2.111 11.206 54.117 1.00 0.00
H
ATOM 1412 HG2 GLN A 259 3.648 11.207 53.307 1.00 0.00
H
ATOM 1413 HE22 GLN A 259 2.066 7.687 53.768 1.00 0.00
H
ATOM 1414 HE21 GLN A 259 2.001 9.061 54.832 1.00 0.00
H
ATOM 1415 N ILE A 260 0.586 14.315 50.647 1.00 32.68
N
ATOM 1416 CA ILE A 260 0.144 15.060 49.467 1.00 32.42
C
ATOM 1417 C ILE A 260 0.281 16.589 49.622 1.00 31.57
C
ATOM 1418 O ILE A 260 0.666 17.244 48.654 1.00 30.43
O
ATOM 1419 CB ILE A 260 -1.318 14.676 49.078 1.00 33.97
C
ATOM 1420 CG1 ILE A 260 -1.409 13.208 48.590 1.00 34.59

C
ATOM 1421 CG2 ILE A 260 -2.025 15.621 48.080 1.00 33.83
C
ATOM 1422 CD1 ILE A 260 -0.826 12.938 47.192 1.00 36.85
C
ATOM 1423 H ILE A 260 -0.145 13.899 51.209 1.00 0.00
H
ATOM 1424 HA ILE A 260 0.800 14.778 48.644 1.00 0.00
H
ATOM 1425 HB ILE A 260 -1.907 14.717 49.996 1.00 0.00
H
ATOM 1426 HG13 ILE A 260 -2.455 12.901 48.593 1.00 0.00
H
ATOM 1427 HG12 ILE A 260 -0.915 12.546 49.301 1.00 0.00
H
ATOM 1428 HG21 ILE A 260 -2.999 15.226 47.790 1.00 0.00
H
ATOM 1429 HG22 ILE A 260 -2.204 16.610 48.504 1.00 0.00
H
ATOM 1430 HG23 ILE A 260 -1.432 15.748 47.175 1.00 0.00
H
ATOM 1431 HD11 ILE A 260 -0.836 11.870 46.976 1.00 0.00
H
ATOM 1432 HD12 ILE A 260 -1.410 13.430 46.413 1.00 0.00
H
ATOM 1433 HD13 ILE A 260 0.206 13.275 47.103 1.00 0.00
H
ATOM 1434 N VAL A 261 0.008 17.125 50.826 1.00 29.87
N
ATOM 1435 CA VAL A 261 0.161 18.546 51.159 1.00 28.91
C
ATOM 1436 C VAL A 261 1.631 19.011 51.087 1.00 27.91
C
ATOM 1437 O VAL A 261 1.892 20.059 50.494 1.00 28.13
O
ATOM 1438 CB VAL A 261 -0.446 18.879 52.557 1.00 28.91

C
ATOM 1439 CG1 VAL A 261 -0.003 20.222 53.173 1.00 30.09
C
ATOM 1440 CG2 VAL A 261 -1.985 18.847 52.508 1.00 31.46
C
ATOM 1441 H VAL A 261 -0.320 16.526 51.574 1.00 0.00
H
ATOM 1442 HA VAL A 261 -0.393 19.113 50.408 1.00 0.00
H
ATOM 1443 HB VAL A 261 -0.133 18.095 53.248 1.00 0.00
H
ATOM 1444 HG11 VAL A 261 -0.538 20.417 54.101 1.00 0.00
H
ATOM 1445 HG12 VAL A 261 1.059 20.241 53.419 1.00 0.00
H
ATOM 1446 HG13 VAL A 261 -0.207 21.053 52.497 1.00 0.00
H
ATOM 1447 HG21 VAL A 261 -2.411 18.924 53.509 1.00 0.00
H
ATOM 1448 HG22 VAL A 261 -2.376 19.674 51.916 1.00 0.00
H
ATOM 1449 HG23 VAL A 261 -2.363 17.928 52.064 1.00 0.00
H
ATOM 1450 N LEU A 262 2.554 18.202 51.639 1.00 27.17
N
ATOM 1451 CA LEU A 262 4.006 18.388 51.550 1.00 25.81
C
ATOM 1452 C LEU A 262 4.507 18.371 50.095 1.00 25.46
C
ATOM 1453 O LEU A 262 5.323 19.217 49.738 1.00 24.78
O
ATOM 1454 CB LEU A 262 4.725 17.286 52.366 1.00 26.02
C
ATOM 1455 CG LEU A 262 4.644 17.442 53.903 1.00 25.71
C
ATOM 1456 CD1 LEU A 262 4.993 16.120 54.621 1.00 26.98

C
ATOM 1457 CD2 LEU A 262 5.479 18.628 54.421 1.00 26.30
C
ATOM 1458 H LEU A 262 2.249 17.366 52.123 1.00 0.00
H
ATOM 1459 HA LEU A 262 4.245 19.367 51.969 1.00 0.00
H
ATOM 1460 HB3 LEU A 262 5.778 17.232 52.086 1.00 0.00
H
ATOM 1461 HB2 LEU A 262 4.304 16.322 52.074 1.00 0.00
H
ATOM 1462 HG LEU A 262 3.609 17.669 54.155 1.00 0.00
H
ATOM 1463 HD11 LEU A 262 5.873 16.199 55.258 1.00 0.00
H
ATOM 1464 HD12 LEU A 262 4.165 15.797 55.252 1.00 0.00
H
ATOM 1465 HD13 LEU A 262 5.186 15.308 53.922 1.00 0.00
H
ATOM 1466 HD21 LEU A 262 4.851 19.316 54.986 1.00 0.00
H
ATOM 1467 HD22 LEU A 262 6.292 18.328 55.079 1.00 0.00
H
ATOM 1468 HD23 LEU A 262 5.928 19.188 53.605 1.00 0.00
H
ATOM 1469 N LEU A 263 3.995 17.424 49.289 1.00 25.71
N
ATOM 1470 CA LEU A 263 4.390 17.210 47.898 1.00 26.15
C
ATOM 1471 C LEU A 263 3.930 18.307 46.930 1.00 26.42
C
ATOM 1472 O LEU A 263 4.751 18.744 46.127 1.00 25.91
O
ATOM 1473 CB LEU A 263 3.913 15.818 47.427 1.00 27.20
C
ATOM 1474 CG LEU A 263 4.861 14.688 47.870 1.00 29.34

C
ATOM 1475 CD1 LEU A 263 4.216 13.298 47.674 1.00 30.14
C
ATOM 1476 CD2 LEU A 263 6.228 14.831 47.169 1.00 31.00
C
ATOM 1477 H LEU A 263 3.324 16.767 49.664 1.00 0.00
H
ATOM 1478 HA LEU A 263 5.481 17.239 47.878 1.00 0.00
H
ATOM 1479 HB3 LEU A 263 3.829 15.785 46.339 1.00 0.00
H
ATOM 1480 HB2 LEU A 263 2.904 15.639 47.800 1.00 0.00
H
ATOM 1481 HG LEU A 263 5.040 14.800 48.941 1.00 0.00
H
ATOM 1482 HD11 LEU A 263 4.839 12.613 47.102 1.00 0.00
H
ATOM 1483 HD12 LEU A 263 4.026 12.818 48.633 1.00 0.00
H
ATOM 1484 HD13 LEU A 263 3.256 13.363 47.162 1.00 0.00
H
ATOM 1485 HD21 LEU A 263 6.577 13.910 46.718 1.00 0.00
H
ATOM 1486 HD22 LEU A 263 6.204 15.574 46.373 1.00 0.00
H
ATOM 1487 HD23 LEU A 263 6.994 15.147 47.875 1.00 0.00
H
ATOM 1488 N LYS A 264 2.657 18.732 47.011 1.00 25.69
N
ATOM 1489 CA LYS A 264 2.093 19.764 46.133 1.00 26.59
C
ATOM 1490 C LYS A 264 2.718 21.154 46.338 1.00 26.97
C
ATOM 1491 O LYS A 264 2.959 21.853 45.354 1.00 27.99
O
ATOM 1492 CB LYS A 264 0.560 19.827 46.287 1.00 29.36

C
ATOM 1493 CG LYS A 264 -0.181 18.659 45.615 1.00 31.84
C
ATOM 1494 CD LYS A 264 -1.702 18.865 45.621 1.00 35.05
C
ATOM 1495 CE LYS A 264 -2.462 17.781 44.845 1.00 37.95
C
ATOM 1496 NZ LYS A 264 -3.899 18.086 44.786 1.00 39.41
N1+
ATOM 1497 H LYS A 264 2.027 18.317 47.685 1.00 0.00
H
ATOM 1498 HA LYS A 264 2.321 19.464 45.110 1.00 0.00
H
ATOM 1499 HB3 LYS A 264 0.201 20.744 45.815 1.00 0.00
H
ATOM 1500 HB2 LYS A 264 0.283 19.903 47.340 1.00 0.00
H
ATOM 1501 HG3 LYS A 264 0.067 17.720 46.109 1.00 0.00
H
ATOM 1502 HG2 LYS A 264 0.162 18.560 44.586 1.00 0.00
H
ATOM 1503 HD3 LYS A 264 -1.936 19.842 45.195 1.00 0.00
H
ATOM 1504 HD2 LYS A 264 -2.056 18.887 46.652 1.00 0.00
H
ATOM 1505 HE3 LYS A 264 -2.323 16.804 45.304 1.00 0.00
H
ATOM 1506 HE2 LYS A 264 -2.089 17.707 43.824 1.00 0.00
H
ATOM 1507 HZ1 LYS A 264 -4.360 17.363 44.238 1.00 0.00
H
ATOM 1508 HZ2 LYS A 264 -4.292 18.116 45.714 1.00 0.00
H
ATOM 1509 HZ3 LYS A 264 -4.034 18.973 44.319 1.00 0.00
H
ATOM 1510 N SER A 265 2.988 21.509 47.605 1.00 26.64

N
ATOM 1511 CA SER A 265 3.602 22.776 48.003 1.00 27.75
C
ATOM 1512 C SER A 265 5.111 22.877 47.695 1.00 26.92
C
ATOM 1513 O SER A 265 5.601 23.997 47.558 1.00 28.28
O
ATOM 1514 CB SER A 265 3.280 23.040 49.489 1.00 28.81
C
ATOM 1515 OG SER A 265 3.911 22.110 50.345 1.00 33.64
O
ATOM 1516 H SER A 265 2.762 20.870 48.355 1.00 0.00
H
ATOM 1517 HA SER A 265 3.112 23.566 47.429 1.00 0.00
H
ATOM 1518 HB3 SER A 265 2.203 23.004 49.660 1.00 0.00
H
ATOM 1519 HB2 SER A 265 3.604 24.042 49.774 1.00 0.00
H
ATOM 1520 HG SER A 265 3.367 21.316 50.388 1.00 0.00
H
ATOM 1521 N SER A 266 5.808 21.733 47.575 1.00 25.17
N
ATOM 1522 CA SER A 266 7.244 21.651 47.281 1.00 23.91
C
ATOM 1523 C SER A 266 7.557 21.232 45.829 1.00 23.51
C
ATOM 1524 O SER A 266 8.737 21.168 45.494 1.00 23.30
O
ATOM 1525 CB SER A 266 7.906 20.690 48.295 1.00 24.45
C
ATOM 1526 OG SER A 266 7.592 19.334 48.037 1.00 24.60
O
ATOM 1527 H SER A 266 5.335 20.851 47.712 1.00 0.00
H
ATOM 1528 HA SER A 266 7.701 22.631 47.427 1.00 0.00

H
ATOM 1529 HB3 SER A 266 7.608 20.938 49.315 1.00 0.00
H
ATOM 1530 HB2 SER A 266 8.991 20.796 48.259 1.00 0.00
H
ATOM 1531 HG SER A 266 6.751 19.138 48.464 1.00 0.00
H
ATOM 1532 N ALA A 267 6.531 20.933 45.014 1.00 22.67
N
ATOM 1533 CA ALA A 267 6.669 20.308 43.695 1.00 23.39
C
ATOM 1534 C ALA A 267 7.707 20.998 42.797 1.00 23.51
C
ATOM 1535 O ALA A 267 8.685 20.363 42.414 1.00 23.55
O
ATOM 1536 CB ALA A 267 5.295 20.228 43.010 1.00 24.51
C
ATOM 1537 H ALA A 267 5.601 20.926 45.407 1.00 0.00
H
ATOM 1538 HA ALA A 267 7.021 19.288 43.863 1.00 0.00
H
ATOM 1539 HB1 ALA A 267 5.384 19.861 41.987 1.00 0.00
H
ATOM 1540 HB2 ALA A 267 4.633 19.544 43.542 1.00 0.00
H
ATOM 1541 HB3 ALA A 267 4.800 21.199 42.974 1.00 0.00
H
ATOM 1542 N ILE A 268 7.505 22.302 42.565 1.00 22.01
N
ATOM 1543 CA ILE A 268 8.390 23.176 41.795 1.00 22.23
C
ATOM 1544 C ILE A 268 9.779 23.387 42.445 1.00 22.05
C
ATOM 1545 O ILE A 268 10.780 23.434 41.737 1.00 20.90
O
ATOM 1546 CB ILE A 268 7.705 24.554 41.529 1.00 23.63

C
ATOM 1547 CG1 ILE A 268 8.538 25.540 40.686 1.00 24.85
C
ATOM 1548 CG2 ILE A 268 7.229 25.267 42.810 1.00 25.18
C
ATOM 1549 CD1 ILE A 268 8.849 25.039 39.272 1.00 25.69
C
ATOM 1550 H ILE A 268 6.700 22.737 42.986 1.00 0.00
H
ATOM 1551 HA ILE A 268 8.554 22.692 40.831 1.00 0.00
H
ATOM 1552 HB ILE A 268 6.816 24.349 40.934 1.00 0.00
H
ATOM 1553 HG13 ILE A 268 9.461 25.800 41.201 1.00 0.00
H
ATOM 1554 HG12 ILE A 268 7.998 26.481 40.594 1.00 0.00
H
ATOM 1555 HG21 ILE A 268 6.655 26.157 42.553 1.00 0.00
H
ATOM 1556 HG22 ILE A 268 6.581 24.645 43.426 1.00 0.00
H
ATOM 1557 HG23 ILE A 268 8.066 25.596 43.426 1.00 0.00
H
ATOM 1558 HD11 ILE A 268 8.773 25.864 38.565 1.00 0.00
H
ATOM 1559 HD12 ILE A 268 9.860 24.641 39.211 1.00 0.00
H
ATOM 1560 HD13 ILE A 268 8.156 24.265 38.939 1.00 0.00
H
ATOM 1561 N GLU A 269 9.835 23.480 43.776 1.00 20.40
N
ATOM 1562 CA GLU A 269 11.063 23.731 44.535 1.00 20.63
C
ATOM 1563 C GLU A 269 12.091 22.592 44.421 1.00 21.33
C
ATOM 1564 O GLU A 269 13.272 22.869 44.218 1.00 20.99

O
ATOM 1565 CB GLU A 269 10.733 24.018 46.013 1.00 20.38
C
ATOM 1566 CG GLU A 269 9.869 25.275 46.231 1.00 22.10
C
ATOM 1567 CD GLU A 269 9.635 25.601 47.710 1.00 23.72
C
ATOM 1568 OE1 GLU A 269 10.490 25.233 48.544 1.00 22.73
O
ATOM 1569 OE2 GLU A 269 8.578 26.196 48.008 1.00 24.58
O1-
ATOM 1570 H GLU A 269 8.993 23.361 44.321 1.00 0.00
H
ATOM 1571 HA GLU A 269 11.526 24.627 44.116 1.00 0.00
H
ATOM 1572 HB3 GLU A 269 11.667 24.125 46.564 1.00 0.00
H
ATOM 1573 HB2 GLU A 269 10.228 23.158 46.455 1.00 0.00
H
ATOM 1574 HG3 GLU A 269 8.902 25.155 45.743 1.00 0.00
H
ATOM 1575 HG2 GLU A 269 10.352 26.132 45.764 1.00 0.00
H
ATOM 1576 N VAL A 270 11.625 21.348 44.595 1.00 20.39
N
ATOM 1577 CA VAL A 270 12.453 20.142 44.513 1.00 20.83
C
ATOM 1578 C VAL A 270 12.904 19.912 43.058 1.00 20.52
C
ATOM 1579 O VAL A 270 14.057 19.563 42.805 1.00 20.87
O
ATOM 1580 CB VAL A 270 11.668 18.888 44.988 1.00 21.55
C
ATOM 1581 CG1 VAL A 270 12.477 17.574 44.920 1.00 21.95
C
ATOM 1582 CG2 VAL A 270 11.137 19.066 46.417 1.00 23.25

C
ATOM 1583 H VAL A 270 10.637 21.215 44.797 1.00 0.00
H
ATOM 1584 HA VAL A 270 13.340 20.275 45.134 1.00 0.00
H
ATOM 1585 HB VAL A 270 10.795 18.775 44.341 1.00 0.00
H
ATOM 1586 HG11 VAL A 270 11.908 16.739 45.330 1.00 0.00
H
ATOM 1587 HG12 VAL A 270 12.752 17.302 43.901 1.00 0.00
H
ATOM 1588 HG13 VAL A 270 13.399 17.653 45.496 1.00 0.00
H
ATOM 1589 HG21 VAL A 270 10.346 18.345 46.605 1.00 0.00
H
ATOM 1590 HG22 VAL A 270 11.924 18.931 47.159 1.00 0.00
H
ATOM 1591 HG23 VAL A 270 10.695 20.043 46.597 1.00 0.00
H
ATOM 1592 N ILE A 271 11.987 20.200 42.132 1.00 20.29
N
ATOM 1593 CA ILE A 271 12.214 20.322 40.703 1.00 20.71
C
ATOM 1594 C ILE A 271 13.350 21.321 40.359 1.00 20.86
C
ATOM 1595 O ILE A 271 14.247 20.960 39.600 1.00 20.78
O
ATOM 1596 CB ILE A 271 10.845 20.642 40.019 1.00 21.89
C
ATOM 1597 CG1 ILE A 271 10.119 19.334 39.625 1.00 24.21
C
ATOM 1598 CG2 ILE A 271 10.847 21.689 38.906 1.00 23.03
C
ATOM 1599 CD1 ILE A 271 8.604 19.462 39.381 1.00 27.85
C
ATOM 1600 H ILE A 271 11.061 20.468 42.444 1.00 0.00

H

ATOM 1601 HA ILE A 271 12.556 19.346 40.356 1.00 0.00
H
ATOM 1602 HB ILE A 271 10.222 21.104 40.772 1.00 0.00
H
ATOM 1603 HG13 ILE A 271 10.259 18.606 40.423 1.00 0.00
H
ATOM 1604 HG12 ILE A 271 10.592 18.891 38.754 1.00 0.00
H
ATOM 1605 HG21 ILE A 271 9.896 21.753 38.380 1.00 0.00
H
ATOM 1606 HG22 ILE A 271 11.063 22.683 39.294 1.00 0.00
H
ATOM 1607 HG23 ILE A 271 11.631 21.458 38.206 1.00 0.00
H
ATOM 1608 HD11 ILE A 271 8.328 19.076 38.401 1.00 0.00
H
ATOM 1609 HD12 ILE A 271 8.045 18.888 40.121 1.00 0.00
H
ATOM 1610 HD13 ILE A 271 8.256 20.493 39.448 1.00 0.00
H
ATOM 1611 N MET A 272 13.356 22.512 40.975 1.00 21.17
N
ATOM 1612 CA MET A 272 14.417 23.514 40.826 1.00 21.57
C
ATOM 1613 C MET A 272 15.788 23.052 41.363 1.00 20.82
C
ATOM 1614 O MET A 272 16.800 23.387 40.743 1.00 22.20
O
ATOM 1615 CB MET A 272 13.987 24.859 41.449 1.00 22.31
C
ATOM 1616 CG MET A 272 12.958 25.620 40.600 1.00 24.16
C
ATOM 1617 SD MET A 272 12.140 26.968 41.492 1.00 26.22
S
ATOM 1618 CE MET A 272 11.261 27.771 40.121 1.00 26.38

C
ATOM 1619 H MET A 272 12.554 22.774 41.543 1.00 0.00
H
ATOM 1620 HA MET A 272 14.557 23.677 39.755 1.00 0.00
H
ATOM 1621 HB3 MET A 272 14.861 25.502 41.568 1.00 0.00
H
ATOM 1622 HB2 MET A 272 13.601 24.708 42.457 1.00 0.00
H
ATOM 1623 HG3 MET A 272 12.188 24.945 40.230 1.00 0.00
H
ATOM 1624 HG2 MET A 272 13.447 26.033 39.717 1.00 0.00
H
ATOM 1625 HE1 MET A 272 10.458 28.402 40.502 1.00 0.00
H
ATOM 1626 HE2 MET A 272 11.945 28.398 39.553 1.00 0.00
H
ATOM 1627 HE3 MET A 272 10.828 27.034 39.447 1.00 0.00
H
ATOM 1628 N LEU A 273 15.815 22.264 42.450 1.00 21.17
N
ATOM 1629 CA LEU A 273 17.038 21.614 42.949 1.00 21.28
C
ATOM 1630 C LEU A 273 17.558 20.558 41.958 1.00 20.96
C
ATOM 1631 O LEU A 273 18.742 20.576 41.620 1.00 20.75
O
ATOM 1632 CB LEU A 273 16.811 20.963 44.336 1.00 22.98
C
ATOM 1633 CG LEU A 273 16.553 21.960 45.480 1.00 25.62
C
ATOM 1634 CD1 LEU A 273 16.072 21.225 46.748 1.00 25.83
C
ATOM 1635 CD2 LEU A 273 17.790 22.833 45.765 1.00 26.65
C
ATOM 1636 H LEU A 273 14.946 22.062 42.931 1.00 0.00

H

ATOM 1637 HA LEU A 273 17.813 22.377 43.039 1.00 0.00

H

ATOM 1638 HB3 LEU A 273 17.675 20.348 44.595 1.00 0.00

H

ATOM 1639 HB2 LEU A 273 15.976 20.268 44.273 1.00 0.00

H

ATOM 1640 HG LEU A 273 15.748 22.628 45.173 1.00 0.00

H

ATOM 1641 HD11 LEU A 273 16.330 21.754 47.666 1.00 0.00

H

ATOM 1642 HD12 LEU A 273 14.987 21.120 46.740 1.00 0.00

H

ATOM 1643 HD13 LEU A 273 16.499 20.225 46.821 1.00 0.00

H

ATOM 1644 HD21 LEU A 273 17.930 23.039 46.826 1.00 0.00

H

ATOM 1645 HD22 LEU A 273 18.710 22.375 45.401 1.00 0.00

H

ATOM 1646 HD23 LEU A 273 17.690 23.794 45.265 1.00 0.00

H

ATOM 1647 N ARG A 274 16.645 19.696 41.485 1.00 19.69

N

ATOM 1648 CA ARG A 274 16.880 18.604 40.532 1.00 19.52

C

ATOM 1649 C ARG A 274 17.305 19.082 39.123 1.00 19.10

C

ATOM 1650 O ARG A 274 17.747 18.277 38.301 1.00 19.83

O

ATOM 1651 CB ARG A 274 15.610 17.718 40.515 1.00 19.75

C

ATOM 1652 CG ARG A 274 15.702 16.435 39.662 1.00 19.52

C

ATOM 1653 CD ARG A 274 14.603 15.402 39.955 1.00 19.72

C

ATOM 1654 NE ARG A 274 14.787 14.792 41.283 1.00 18.11

N
ATOM 1655 CZ ARG A 274 14.558 13.517 41.634 1.00 19.01
C
ATOM 1656 NH1 ARG A 274 14.172 12.595 40.745 1.00 18.53
N
ATOM 1657 NH2 ARG A 274 14.722 13.156 42.909 1.00 19.51
N1+
ATOM 1658 H ARG A 274 15.695 19.769 41.841 1.00 0.00
H
ATOM 1659 HA ARG A 274 17.705 18.005 40.921 1.00 0.00
H
ATOM 1660 HB3 ARG A 274 14.755 18.304 40.177 1.00 0.00
H
ATOM 1661 HB2 ARG A 274 15.392 17.438 41.546 1.00 0.00
H
ATOM 1662 HG3 ARG A 274 16.654 15.967 39.923 1.00 0.00
H
ATOM 1663 HG2 ARG A 274 15.753 16.642 38.594 1.00 0.00
H
ATOM 1664 HD3 ARG A 274 14.527 14.682 39.140 1.00 0.00
H
ATOM 1665 HD2 ARG A 274 13.635 15.902 40.008 1.00 0.00
H
ATOM 1666 HE ARG A 274 15.097 15.438 42.000 1.00 0.00
H
ATOM 1667 HH12 ARG A 274 14.052 11.624 41.020 1.00 0.00
H
ATOM 1668 HH11 ARG A 274 14.052 12.823 39.767 1.00 0.00
H
ATOM 1669 HH22 ARG A 274 14.544 12.206 43.203 1.00 0.00
H
ATOM 1670 HH21 ARG A 274 15.026 13.826 43.604 1.00 0.00
H
ATOM 1671 N SER A 275 17.162 20.385 38.858 1.00 19.73
N
ATOM 1672 CA SER A 275 17.575 21.022 37.615 1.00 19.93

C
ATOM 1673 C SER A 275 19.099 21.160 37.501 1.00 19.90
C
ATOM 1674 O SER A 275 19.621 21.205 36.389 1.00 20.21
O
ATOM 1675 CB SER A 275 16.822 22.365 37.482 1.00 20.51
C
ATOM 1676 OG SER A 275 17.479 23.500 37.988 1.00 20.38
O
ATOM 1677 H SER A 275 16.743 20.974 39.564 1.00 0.00
H
ATOM 1678 HA SER A 275 17.260 20.378 36.791 1.00 0.00
H
ATOM 1679 HB3 SER A 275 15.865 22.328 37.998 1.00 0.00
H
ATOM 1680 HB2 SER A 275 16.605 22.539 36.431 1.00 0.00
H
ATOM 1681 HG SER A 275 17.457 23.461 38.958 1.00 0.00
H
ATOM 1682 N ASN A 276 19.805 21.209 38.641 1.00 20.28
N
ATOM 1683 CA ASN A 276 21.243 21.482 38.667 1.00 19.85
C
ATOM 1684 C ASN A 276 22.073 20.381 37.984 1.00 21.26
C
ATOM 1685 O ASN A 276 23.129 20.675 37.435 1.00 19.72
O
ATOM 1686 CB ASN A 276 21.723 21.740 40.107 1.00 20.77
C
ATOM 1687 CG ASN A 276 22.850 22.773 40.175 1.00 23.39
C
ATOM 1688 OD1 ASN A 276 23.997 22.439 40.443 1.00 26.18
O
ATOM 1689 ND2 ASN A 276 22.542 24.043 39.941 1.00 21.92
N
ATOM 1690 H ASN A 276 19.336 21.085 39.531 1.00 0.00

H
ATOM 1691 HA ASN A 276 21.440 22.369 38.064 1.00 0.00
H
ATOM 1692 HB3 ASN A 276 21.999 20.816 40.619 1.00 0.00
H
ATOM 1693 HB2 ASN A 276 20.896 22.162 40.675 1.00 0.00
H
ATOM 1694 HD22 ASN A 276 23.237 24.772 40.017 1.00 0.00
H
ATOM 1695 HD21 ASN A 276 21.568 24.306 39.748 1.00 0.00
H
ATOM 1696 N GLU A 277 21.534 19.158 37.967 1.00 20.52
N
ATOM 1697 CA GLU A 277 22.080 17.978 37.304 1.00 21.75
C
ATOM 1698 C GLU A 277 22.127 18.108 35.766 1.00 21.79
C
ATOM 1699 O GLU A 277 23.042 17.559 35.157 1.00 22.41
O
ATOM 1700 CB GLU A 277 21.260 16.735 37.735 1.00 22.91
C
ATOM 1701 CG GLU A 277 21.537 16.195 39.160 1.00 26.24
C
ATOM 1702 CD GLU A 277 21.299 17.190 40.296 1.00 28.07
C
ATOM 1703 OE1 GLU A 277 20.235 17.847 40.275 1.00 30.43
O
ATOM 1704 OE2 GLU A 277 22.210 17.313 41.142 1.00 26.56
O1-
ATOM 1705 H GLU A 277 20.687 18.999 38.500 1.00 0.00
H
ATOM 1706 HA GLU A 277 23.111 17.849 37.638 1.00 0.00
H
ATOM 1707 HB3 GLU A 277 21.442 15.921 37.031 1.00 0.00
H
ATOM 1708 HB2 GLU A 277 20.196 16.956 37.648 1.00 0.00

H

ATOM 1709 HG3 GLU A 277 22.564 15.834 39.217 1.00 0.00
H
ATOM 1710 HG2 GLU A 277 20.899 15.329 39.344 1.00 0.00
H
ATOM 1711 N SER A 278 21.163 18.821 35.167 1.00 19.68
N
ATOM 1712 CA SER A 278 21.073 19.043 33.722 1.00 20.64
C
ATOM 1713 C SER A 278 21.474 20.478 33.320 1.00 21.58
C
ATOM 1714 O SER A 278 21.708 20.733 32.137 1.00 22.05
O
ATOM 1715 CB SER A 278 19.648 18.665 33.271 1.00 20.93
C
ATOM 1716 OG SER A 278 18.658 19.284 34.074 1.00 22.00
O
ATOM 1717 H SER A 278 20.431 19.271 35.704 1.00 0.00
H
ATOM 1718 HA SER A 278 21.760 18.383 33.189 1.00 0.00
H
ATOM 1719 HB3 SER A 278 19.509 17.586 33.318 1.00 0.00
H
ATOM 1720 HB2 SER A 278 19.489 18.942 32.228 1.00 0.00
H
ATOM 1721 HG SER A 278 17.795 18.932 33.782 1.00 0.00
H
ATOM 1722 N PHE A 279 21.560 21.403 34.284 1.00 21.92
N
ATOM 1723 CA PHE A 279 22.044 22.765 34.079 1.00 23.24
C
ATOM 1724 C PHE A 279 23.560 22.772 33.829 1.00 24.55
C
ATOM 1725 O PHE A 279 24.293 22.068 34.524 1.00 24.49
O
ATOM 1726 CB PHE A 279 21.670 23.637 35.296 1.00 23.08

C
ATOM 1727 CG PHE A 279 21.913 25.123 35.102 1.00 24.02
C
ATOM 1728 CD1 PHE A 279 21.064 25.855 34.248 1.00 24.96
C
ATOM 1729 CD2 PHE A 279 23.056 25.754 35.641 1.00 24.94
C
ATOM 1730 CE1 PHE A 279 21.334 27.186 33.976 1.00 24.93
C
ATOM 1731 CE2 PHE A 279 23.293 27.097 35.376 1.00 25.50
C
ATOM 1732 CZ PHE A 279 22.435 27.809 34.548 1.00 24.47
C
ATOM 1733 H PHE A 279 21.286 21.149 35.225 1.00 0.00
H
ATOM 1734 HA PHE A 279 21.553 23.179 33.204 1.00 0.00
H
ATOM 1735 HB3 PHE A 279 22.210 23.297 36.179 1.00 0.00
H
ATOM 1736 HB2 PHE A 279 20.612 23.512 35.522 1.00 0.00
H
ATOM 1737 HD1 PHE A 279 20.206 25.382 33.792 1.00 0.00
H
ATOM 1738 HD2 PHE A 279 23.737 25.204 36.272 1.00 0.00
H
ATOM 1739 HE1 PHE A 279 20.683 27.727 33.307 1.00 0.00
H
ATOM 1740 HE2 PHE A 279 24.150 27.592 35.809 1.00 0.00
H
ATOM 1741 HZ PHE A 279 22.633 28.849 34.338 1.00 0.00
H
ATOM 1742 N THR A 280 24.016 23.608 32.895 1.00 24.73
N
ATOM 1743 CA THR A 280 25.430 23.912 32.718 1.00 26.87
C
ATOM 1744 C THR A 280 25.620 25.428 32.527 1.00 27.44

C
ATOM 1745 O THR A 280 24.775 26.087 31.916 1.00 26.28
O
ATOM 1746 CB THR A 280 26.050 23.125 31.530 1.00 27.76
C
ATOM 1747 OG1 THR A 280 27.447 23.340 31.510 1.00 31.72
O
ATOM 1748 CG2 THR A 280 25.502 23.482 30.134 1.00 27.79
C
ATOM 1749 H THR A 280 23.371 24.191 32.378 1.00 0.00
H
ATOM 1750 HA THR A 280 25.968 23.638 33.629 1.00 0.00
H
ATOM 1751 HB THR A 280 25.891 22.060 31.701 1.00 0.00
H
ATOM 1752 HG1 THR A 280 27.814 23.016 30.680 1.00 0.00
H
ATOM 1753 HG21 THR A 280 25.928 22.831 29.370 1.00 0.00
H
ATOM 1754 HG22 THR A 280 24.419 23.365 30.093 1.00 0.00
H
ATOM 1755 HG23 THR A 280 25.736 24.508 29.848 1.00 0.00
H
ATOM 1756 N MET A 281 26.738 25.940 33.063 1.00 28.33
N
ATOM 1757 CA MET A 281 27.133 27.348 33.011 1.00 31.03
C
ATOM 1758 C MET A 281 27.845 27.745 31.701 1.00 31.28
C
ATOM 1759 O MET A 281 28.112 28.932 31.521 1.00 30.87
O
ATOM 1760 CB MET A 281 27.992 27.675 34.252 1.00 33.43
C
ATOM 1761 CG MET A 281 27.203 27.591 35.571 1.00 36.11
C
ATOM 1762 SD MET A 281 28.173 27.767 37.097 1.00 39.85

S
ATOM 1763 CE MET A 281 28.926 29.399 36.860 1.00 40.40
C
ATOM 1764 H MET A 281 27.391 25.318 33.518 1.00 0.00
H
ATOM 1765 HA MET A 281 26.230 27.957 33.059 1.00 0.00
H
ATOM 1766 HB3 MET A 281 28.391 28.685 34.156 1.00 0.00
H
ATOM 1767 HB2 MET A 281 28.857 27.012 34.295 1.00 0.00
H
ATOM 1768 HG3 MET A 281 26.687 26.633 35.640 1.00 0.00
H
ATOM 1769 HG2 MET A 281 26.424 28.352 35.571 1.00 0.00
H
ATOM 1770 HE1 MET A 281 29.456 29.697 37.764 1.00 0.00
H
ATOM 1771 HE2 MET A 281 29.642 29.376 36.039 1.00 0.00
H
ATOM 1772 HE3 MET A 281 28.169 30.149 36.642 1.00 0.00
H
ATOM 1773 N ASP A 282 28.112 26.772 30.807 1.00 31.72
N
ATOM 1774 CA ASP A 282 28.717 26.958 29.477 1.00 32.91
C
ATOM 1775 C ASP A 282 27.915 27.897 28.555 1.00 32.02
C
ATOM 1776 O ASP A 282 28.517 28.612 27.755 1.00 31.15
O
ATOM 1777 CB ASP A 282 28.963 25.623 28.726 1.00 35.93
C
ATOM 1778 CG ASP A 282 29.658 24.524 29.536 1.00 39.91
C
ATOM 1779 OD1 ASP A 282 30.469 24.868 30.424 1.00 42.35
O
ATOM 1780 OD2 ASP A 282 29.400 23.344 29.211 1.00 42.45

O1-
ATOM 1781 H ASP A 282 27.888 25.817 31.049 1.00 0.00
H
ATOM 1782 HA ASP A 282 29.688 27.431 29.641 1.00 0.00
H
ATOM 1783 HB3 ASP A 282 29.552 25.801 27.826 1.00 0.00
H
ATOM 1784 HB2 ASP A 282 27.996 25.230 28.406 1.00 0.00
H
ATOM 1785 N ASP A 283 26.580 27.873 28.695 1.00 29.87
N
ATOM 1786 CA ASP A 283 25.631 28.671 27.914 1.00 28.59
C
ATOM 1787 C ASP A 283 24.420 29.129 28.755 1.00 27.92
C
ATOM 1788 O ASP A 283 23.576 29.850 28.223 1.00 27.21
O
ATOM 1789 CB ASP A 283 25.190 27.980 26.592 1.00 29.84
C
ATOM 1790 CG ASP A 283 24.795 26.498 26.692 1.00 31.11
C
ATOM 1791 OD1 ASP A 283 24.505 26.021 27.812 1.00 28.99
O
ATOM 1792 OD2 ASP A 283 24.716 25.869 25.614 1.00 32.79
O1-
ATOM 1793 H ASP A 283 26.170 27.209 29.335 1.00 0.00
H
ATOM 1794 HA ASP A 283 26.131 29.601 27.636 1.00 0.00
H
ATOM 1795 HB3 ASP A 283 26.031 28.043 25.900 1.00 0.00
H
ATOM 1796 HB2 ASP A 283 24.362 28.522 26.133 1.00 0.00
H
ATOM 1797 N MET A 284 24.356 28.720 30.038 1.00 26.84
N
ATOM 1798 CA MET A 284 23.281 29.001 30.998 1.00 27.62

C
ATOM 1799 C MET A 284 21.934 28.409 30.539 1.00 27.20
C
ATOM 1800 O MET A 284 20.954 29.141 30.392 1.00 27.86
O
ATOM 1801 CB MET A 284 23.204 30.514 31.339 1.00 29.95
C
ATOM 1802 CG MET A 284 24.503 31.111 31.907 1.00 33.34
C
ATOM 1803 SD MET A 284 24.967 30.498 33.550 1.00 36.23
S
ATOM 1804 CE MET A 284 26.542 31.364 33.761 1.00 35.78
C
ATOM 1805 H MET A 284 25.099 28.138 30.395 1.00 0.00
H
ATOM 1806 HA MET A 284 23.547 28.471 31.911 1.00 0.00
H
ATOM 1807 HB3 MET A 284 22.413 30.676 32.071 1.00 0.00
H
ATOM 1808 HB2 MET A 284 22.906 31.088 30.462 1.00 0.00
H
ATOM 1809 HG3 MET A 284 24.394 32.193 31.978 1.00 0.00
H
ATOM 1810 HG2 MET A 284 25.333 30.935 31.223 1.00 0.00
H
ATOM 1811 HE1 MET A 284 27.002 31.085 34.707 1.00 0.00
H
ATOM 1812 HE2 MET A 284 27.230 31.108 32.955 1.00 0.00
H
ATOM 1813 HE3 MET A 284 26.387 32.443 33.756 1.00 0.00
H
ATOM 1814 N SER A 285 21.908 27.091 30.325 1.00 25.58
N
ATOM 1815 CA SER A 285 20.703 26.389 29.909 1.00 24.62
C
ATOM 1816 C SER A 285 20.626 25.024 30.600 1.00 24.72

C
ATOM 1817 O SER A 285 21.660 24.427 30.925 1.00 24.18
O
ATOM 1818 CB SER A 285 20.669 26.325 28.365 1.00 24.98
C
ATOM 1819 OG SER A 285 21.679 25.503 27.808 1.00 25.94
O
ATOM 1820 H SER A 285 22.710 26.509 30.534 1.00 0.00
H
ATOM 1821 HA SER A 285 19.824 26.948 30.239 1.00 0.00
H
ATOM 1822 HB3 SER A 285 20.745 27.326 27.939 1.00 0.00
H
ATOM 1823 HB2 SER A 285 19.708 25.925 28.040 1.00 0.00
H
ATOM 1824 HG SER A 285 22.554 25.882 27.981 1.00 0.00
H
ATOM 1825 N TRP A 286 19.391 24.559 30.807 1.00 24.17
N
ATOM 1826 CA TRP A 286 19.097 23.214 31.277 1.00 24.74
C
ATOM 1827 C TRP A 286 19.113 22.296 30.051 1.00 25.34
C
ATOM 1828 O TRP A 286 18.281 22.466 29.161 1.00 25.37
O
ATOM 1829 CB TRP A 286 17.753 23.197 32.019 1.00 23.76
C
ATOM 1830 CG TRP A 286 17.658 24.040 33.248 1.00 21.76
C
ATOM 1831 CD1 TRP A 286 18.041 23.636 34.475 1.00 21.47
C
ATOM 1832 CD2 TRP A 286 17.195 25.417 33.406 1.00 23.03
C
ATOM 1833 NE1 TRP A 286 17.803 24.639 35.389 1.00 21.88
N
ATOM 1834 CE2 TRP A 286 17.298 25.771 34.786 1.00 22.96

C
ATOM 1835 CE3 TRP A 286 16.676 26.399 32.529 1.00 23.54
C
ATOM 1836 CZ2 TRP A 286 16.910 27.033 35.268 1.00 24.15
C
ATOM 1837 CZ3 TRP A 286 16.281 27.668 33.001 1.00 22.92
C
ATOM 1838 CH2 TRP A 286 16.400 27.986 34.367 1.00 23.66
C
ATOM 1839 H TRP A 286 18.598 25.074 30.438 1.00 0.00
H
ATOM 1840 HA TRP A 286 19.858 22.878 31.976 1.00 0.00
H
ATOM 1841 HB3 TRP A 286 17.554 22.180 32.347 1.00 0.00
H
ATOM 1842 HB2 TRP A 286 16.937 23.482 31.356 1.00 0.00
H
ATOM 1843 HD1 TRP A 286 18.426 22.650 34.693 1.00 0.00
H
ATOM 1844 HE1 TRP A 286 17.898 24.467 36.398 1.00 0.00
H
ATOM 1845 HE3 TRP A 286 16.563 26.166 31.482 1.00 0.00
H
ATOM 1846 HZ2 TRP A 286 17.001 27.267 36.316 1.00 0.00
H
ATOM 1847 HZ3 TRP A 286 15.881 28.396 32.310 1.00 0.00
H
ATOM 1848 HH2 TRP A 286 16.095 28.959 34.721 1.00 0.00
H
ATOM 1849 N THR A 287 20.093 21.398 29.988 1.00 25.53
N
ATOM 1850 CA THR A 287 20.399 20.603 28.810 1.00 27.14
C
ATOM 1851 C THR A 287 20.090 19.124 29.082 1.00 27.53
C
ATOM 1852 O THR A 287 20.761 18.502 29.905 1.00 27.40

O

ATOM	1853	CB	THR	A	287	21.902	20.751	28.465	1.00	27.77	C
ATOM	1854	OG1	THR	A	287	22.196	22.127	28.296	1.00	30.32	O
ATOM	1855	CG2	THR	A	287	22.334	20.035	27.173	1.00	29.69	C
ATOM	1856	H	THR	A	287	20.720	21.246	30.779	1.00	0.00	H
ATOM	1857	HA	THR	A	287	19.818	20.926	27.948	1.00	0.00	H
ATOM	1858	HB	THR	A	287	22.514	20.385	29.293	1.00	0.00	H
ATOM	1859	HG1	THR	A	287	21.948	22.605	29.099	1.00	0.00	H
ATOM	1860	HG21	THR	A	287	23.378	20.247	26.938	1.00	0.00	H
ATOM	1861	HG22	THR	A	287	22.241	18.952	27.257	1.00	0.00	H
ATOM	1862	HG23	THR	A	287	21.732	20.352	26.320	1.00	0.00	H
ATOM	1863	N	CYS	A	288	19.084	18.585	28.387	1.00	28.08	N
ATOM	1864	CA	CYS	A	288	18.688	17.184	28.479	1.00	30.34	C
ATOM	1865	C	CYS	A	288	19.042	16.477	27.176	1.00	33.66	C
ATOM	1866	O	CYS	A	288	18.162	16.028	26.443	1.00	33.19	O
ATOM	1867	CB	CYS	A	288	17.206	17.028	28.836	1.00	29.32	C
ATOM	1868	SG	CYS	A	288	17.093	17.207	30.622	1.00	28.01	S
ATOM	1869	H	CYS	A	288	18.553	19.139	27.716	1.00	0.00	H
ATOM	1870	HA	CYS	A	288	19.273	16.671	29.242	1.00	0.00	

H
ATOM 1871 HB3 CYS A 288 16.818 16.043 28.579 1.00 0.00
H
ATOM 1872 HB2 CYS A 288 16.586 17.763 28.328 1.00 0.00
H
ATOM 1873 HG CYS A 288 17.744 16.073 30.914 1.00 0.00
H
ATOM 1874 N GLY A 289 20.345 16.360 26.919 1.00 37.69
N
ATOM 1875 CA GLY A 289 20.816 15.636 25.743 1.00 41.85
C
ATOM 1876 C GLY A 289 20.994 16.636 24.596 1.00 44.11
C
ATOM 1877 O GLY A 289 21.807 17.555 24.701 1.00 45.10
O
ATOM 1878 H GLY A 289 21.047 16.792 27.502 1.00 0.00
H
ATOM 1879 HA3 GLY A 289 20.140 14.823 25.467 1.00 0.00
H
ATOM 1880 HA2 GLY A 289 21.780 15.182 25.972 1.00 0.00
H
ATOM 1881 N ASN A 290 20.266 16.408 23.490 1.00 46.33
N
ATOM 1882 CA ASN A 290 20.326 17.167 22.231 1.00 47.32
C
ATOM 1883 C ASN A 290 19.929 18.657 22.348 1.00 47.22
C
ATOM 1884 O ASN A 290 19.392 19.084 23.370 1.00 47.49
O
ATOM 1885 CB ASN A 290 19.508 16.414 21.146 1.00 49.56
C
ATOM 1886 CG ASN A 290 18.019 16.215 21.468 1.00 51.07
C
ATOM 1887 OD1 ASN A 290 17.277 17.180 21.622 1.00 52.29
O
ATOM 1888 ND2 ASN A 290 17.572 14.961 21.550 1.00 51.45

N

ATOM 1889 H ASN A 290 19.612 15.639 23.496 1.00 0.00

H

ATOM 1890 HA ASN A 290 21.373 17.136 21.926 1.00 0.00

H

ATOM 1891 HB3 ASN A 290 19.965 15.437 20.976 1.00 0.00

H

ATOM 1892 HB2 ASN A 290 19.576 16.935 20.191 1.00 0.00

H

ATOM 1893 HD22 ASN A 290 16.597 14.791 21.749 1.00 0.00

H

ATOM 1894 HD21 ASN A 290 18.193 14.179 21.404 1.00 0.00

H

ATOM 1895 N GLN A 291 20.193 19.406 21.263 1.00 46.24

N

ATOM 1896 CA GLN A 291 19.924 20.841 21.112 1.00 45.60

C

ATOM 1897 C GLN A 291 18.436 21.245 21.154 1.00 43.20

C

ATOM 1898 O GLN A 291 18.146 22.394 21.489 1.00 43.27

O

ATOM 1899 CB GLN A 291 20.570 21.331 19.794 1.00 47.46

C

ATOM 1900 CG GLN A 291 22.114 21.286 19.761 1.00 50.94

C

ATOM 1901 CD GLN A 291 22.793 22.239 20.750 1.00 52.59

C

ATOM 1902 OE1 GLN A 291 22.240 23.272 21.121 1.00 54.12

O

ATOM 1903 NE2 GLN A 291 24.020 21.908 21.157 1.00 53.69

N

ATOM 1904 H GLN A 291 20.620 18.963 20.463 1.00 0.00

H

ATOM 1905 HA GLN A 291 20.401 21.346 21.953 1.00 0.00

H

ATOM 1906 HB3 GLN A 291 20.245 22.350 19.574 1.00 0.00

H
ATOM 1907 HB2 GLN A 291 20.189 20.727 18.969 1.00 0.00
H
ATOM 1908 HG3 GLN A 291 22.456 21.553 18.761 1.00 0.00
H
ATOM 1909 HG2 GLN A 291 22.463 20.268 19.938 1.00 0.00
H
ATOM 1910 HE22 GLN A 291 24.527 22.517 21.781 1.00 0.00
H
ATOM 1911 HE21 GLN A 291 24.460 21.061 20.827 1.00 0.00
H
ATOM 1912 N ASP A 292 17.527 20.312 20.824 1.00 40.92
N
ATOM 1913 CA ASP A 292 16.071 20.508 20.875 1.00 38.58
C
ATOM 1914 C ASP A 292 15.539 20.431 22.316 1.00 35.80
C
ATOM 1915 O ASP A 292 14.636 21.192 22.665 1.00 34.04
O
ATOM 1916 CB ASP A 292 15.289 19.532 19.961 1.00 41.69
C
ATOM 1917 CG ASP A 292 15.838 19.404 18.533 1.00 44.13
C
ATOM 1918 OD1 ASP A 292 15.635 18.319 17.949 1.00 45.48
O
ATOM 1919 OD2 ASP A 292 16.379 20.410 18.022 1.00 45.78
O1-
ATOM 1920 H ASP A 292 17.837 19.391 20.547 1.00 0.00
H
ATOM 1921 HA ASP A 292 15.863 21.518 20.514 1.00 0.00
H
ATOM 1922 HB3 ASP A 292 14.245 19.838 19.894 1.00 0.00
H
ATOM 1923 HB2 ASP A 292 15.301 18.540 20.413 1.00 0.00
H
ATOM 1924 N TYR A 293 16.164 19.563 23.124 1.00 33.09

N
ATOM 1925 CA TYR A 293 15.968 19.406 24.568 1.00 31.43
C
ATOM 1926 C TYR A 293 17.012 20.213 25.374 1.00 30.28
C
ATOM 1927 O TYR A 293 17.343 19.842 26.505 1.00 30.56
O
ATOM 1928 CB TYR A 293 16.034 17.907 24.942 1.00 31.64
C
ATOM 1929 CG TYR A 293 15.061 16.948 24.272 1.00 31.43
C
ATOM 1930 CD1 TYR A 293 13.789 17.368 23.826 1.00 32.22
C
ATOM 1931 CD2 TYR A 293 15.436 15.596 24.121 1.00 32.09
C
ATOM 1932 CE1 TYR A 293 12.909 16.447 23.227 1.00 32.84
C
ATOM 1933 CE2 TYR A 293 14.562 14.678 23.510 1.00 31.78
C
ATOM 1934 CZ TYR A 293 13.294 15.101 23.073 1.00 33.14
C
ATOM 1935 OH TYR A 293 12.428 14.196 22.537 1.00 32.75
O
ATOM 1936 H TYR A 293 16.863 18.949 22.720 1.00 0.00
H
ATOM 1937 HA TYR A 293 14.992 19.799 24.856 1.00 0.00
H
ATOM 1938 HB3 TYR A 293 15.888 17.787 26.016 1.00 0.00
H
ATOM 1939 HB2 TYR A 293 17.045 17.555 24.730 1.00 0.00
H
ATOM 1940 HD1 TYR A 293 13.471 18.394 23.939 1.00 0.00
H
ATOM 1941 HD2 TYR A 293 16.399 15.261 24.479 1.00 0.00
H
ATOM 1942 HE1 TYR A 293 11.943 16.787 22.887 1.00 0.00

H
ATOM 1943 HE2 TYR A 293 14.853 13.643 23.409 1.00 0.00
H
ATOM 1944 HH TYR A 293 11.514 14.400 22.768 1.00 0.00
H
ATOM 1945 N LYS A 294 17.510 21.314 24.800 1.00 29.44
N
ATOM 1946 CA LYS A 294 18.247 22.339 25.521 1.00 27.92
C
ATOM 1947 C LYS A 294 17.237 23.454 25.803 1.00 27.57
C
ATOM 1948 O LYS A 294 16.749 24.076 24.863 1.00 27.85
O
ATOM 1949 CB LYS A 294 19.459 22.803 24.688 1.00 31.41
C
ATOM 1950 CG LYS A 294 20.324 23.864 25.394 1.00 32.77
C
ATOM 1951 CD LYS A 294 21.561 24.300 24.587 1.00 36.06
C
ATOM 1952 CE LYS A 294 22.733 23.308 24.632 1.00 37.32
C
ATOM 1953 NZ LYS A 294 23.418 23.330 25.934 1.00 37.49
N1+
ATOM 1954 H LYS A 294 17.182 21.572 23.880 1.00 0.00
H
ATOM 1955 HA LYS A 294 18.619 21.958 26.469 1.00 0.00
H
ATOM 1956 HB3 LYS A 294 19.114 23.204 23.736 1.00 0.00
H
ATOM 1957 HB2 LYS A 294 20.069 21.931 24.451 1.00 0.00
H
ATOM 1958 HG3 LYS A 294 20.630 23.492 26.373 1.00 0.00
H
ATOM 1959 HG2 LYS A 294 19.722 24.750 25.594 1.00 0.00
H
ATOM 1960 HD3 LYS A 294 21.898 25.275 24.942 1.00 0.00

H
ATOM 1961 HD2 LYS A 294 21.279 24.450 23.546 1.00 0.00
H
ATOM 1962 HE3 LYS A 294 23.466 23.579 23.872 1.00 0.00
H
ATOM 1963 HE2 LYS A 294 22.403 22.294 24.406 1.00 0.00
H
ATOM 1964 HZ1 LYS A 294 22.775 23.088 26.677 1.00 0.00
H
ATOM 1965 HZ2 LYS A 294 24.185 22.676 25.930 1.00 0.00
H
ATOM 1966 HZ3 LYS A 294 23.781 24.262 26.105 1.00 0.00
H
ATOM 1967 N TYR A 295 16.903 23.650 27.076 1.00 25.07
N
ATOM 1968 CA TYR A 295 15.884 24.583 27.533 1.00 24.97
C
ATOM 1969 C TYR A 295 16.620 25.857 27.962 1.00 25.69
C
ATOM 1970 O TYR A 295 17.206 25.918 29.046 1.00 24.37
O
ATOM 1971 CB TYR A 295 15.047 23.935 28.656 1.00 24.19
C
ATOM 1972 CG TYR A 295 14.337 22.645 28.254 1.00 24.03
C
ATOM 1973 CD1 TYR A 295 12.962 22.636 27.939 1.00 23.33
C
ATOM 1974 CD2 TYR A 295 15.055 21.433 28.212 1.00 23.84
C
ATOM 1975 CE1 TYR A 295 12.315 21.423 27.621 1.00 24.85
C
ATOM 1976 CE2 TYR A 295 14.411 20.225 27.901 1.00 24.18
C
ATOM 1977 CZ TYR A 295 13.041 20.214 27.610 1.00 24.74
C
ATOM 1978 OH TYR A 295 12.444 19.020 27.345 1.00 24.72

O
ATOM 1979 H TYR A 295 17.379 23.129 27.812 1.00 0.00
H
ATOM 1980 HA TYR A 295 15.197 24.824 26.719 1.00 0.00
H
ATOM 1981 HB3 TYR A 295 14.303 24.648 29.017 1.00 0.00
H
ATOM 1982 HB2 TYR A 295 15.676 23.714 29.517 1.00 0.00
H
ATOM 1983 HD1 TYR A 295 12.392 23.553 27.969 1.00 0.00
H
ATOM 1984 HD2 TYR A 295 16.111 21.415 28.422 1.00 0.00
H
ATOM 1985 HE1 TYR A 295 11.254 21.421 27.433 1.00 0.00
H
ATOM 1986 HE2 TYR A 295 14.974 19.306 27.883 1.00 0.00
H
ATOM 1987 HH TYR A 295 11.801 19.027 26.614 1.00 0.00
H
ATOM 1988 N ARG A 296 16.666 26.808 27.026 1.00 27.37
N
ATOM 1989 CA ARG A 296 17.321 28.106 27.151 1.00 29.21
C
ATOM 1990 C ARG A 296 16.367 29.137 27.777 1.00 28.74
C
ATOM 1991 O ARG A 296 15.153 28.923 27.789 1.00 28.04
O
ATOM 1992 CB ARG A 296 17.743 28.574 25.739 1.00 31.67
C
ATOM 1993 CG ARG A 296 18.588 27.551 24.951 1.00 36.71
C
ATOM 1994 CD ARG A 296 18.799 27.972 23.489 1.00 40.42
C
ATOM 1995 NE ARG A 296 19.433 26.910 22.695 1.00 44.23
N
ATOM 1996 CZ ARG A 296 18.824 25.811 22.221 1.00 46.02

C
ATOM 1997 NH1 ARG A 296 17.525 25.579 22.437 1.00 46.77
N
ATOM 1998 NH2 ARG A 296 19.528 24.915 21.524 1.00 46.52
N1+
ATOM 1999 H ARG A 296 16.176 26.639 26.154 1.00 0.00
H
ATOM 2000 HA ARG A 296 18.202 28.015 27.789 1.00 0.00
H
ATOM 2001 HB3 ARG A 296 18.285 29.519 25.801 1.00 0.00
H
ATOM 2002 HB2 ARG A 296 16.841 28.793 25.165 1.00 0.00
H
ATOM 2003 HG3 ARG A 296 18.178 26.541 24.977 1.00 0.00
H
ATOM 2004 HG2 ARG A 296 19.556 27.493 25.451 1.00 0.00
H
ATOM 2005 HD3 ARG A 296 19.541 28.771 23.488 1.00 0.00
H
ATOM 2006 HD2 ARG A 296 17.904 28.387 23.023 1.00 0.00
H
ATOM 2007 HE ARG A 296 20.424 27.025 22.533 1.00 0.00
H
ATOM 2008 HH12 ARG A 296 17.110 24.707 22.141 1.00 0.00
H
ATOM 2009 HH11 ARG A 296 16.965 26.179 23.038 1.00 0.00
H
ATOM 2010 HH22 ARG A 296 19.085 24.046 21.242 1.00 0.00
H
ATOM 2011 HH21 ARG A 296 20.521 25.011 21.355 1.00 0.00
H
ATOM 2012 N VAL A 297 16.919 30.280 28.208 1.00 29.11
N
ATOM 2013 CA VAL A 297 16.163 31.409 28.762 1.00 30.41
C
ATOM 2014 C VAL A 297 15.093 31.954 27.785 1.00 30.24

C
ATOM 2015 O VAL A 297 13.969 32.214 28.203 1.00 29.94
O
ATOM 2016 CB VAL A 297 17.114 32.561 29.207 1.00 30.71
C
ATOM 2017 CG1 VAL A 297 16.404 33.845 29.697 1.00 33.35
C
ATOM 2018 CG2 VAL A 297 18.101 32.073 30.287 1.00 32.09
C
ATOM 2019 H VAL A 297 17.921 30.387 28.194 1.00 0.00
H
ATOM 2020 HA VAL A 297 15.639 31.036 29.641 1.00 0.00
H
ATOM 2021 HB VAL A 297 17.712 32.855 28.343 1.00 0.00
H
ATOM 2022 HG11 VAL A 297 17.119 34.570 30.086 1.00 0.00
H
ATOM 2023 HG12 VAL A 297 15.861 34.348 28.895 1.00 0.00
H
ATOM 2024 HG13 VAL A 297 15.689 33.633 30.490 1.00 0.00
H
ATOM 2025 HG21 VAL A 297 18.754 32.878 30.625 1.00 0.00
H
ATOM 2026 HG22 VAL A 297 17.575 31.687 31.159 1.00 0.00
H
ATOM 2027 HG23 VAL A 297 18.750 31.276 29.922 1.00 0.00
H
ATOM 2028 N SER A 298 15.422 32.036 26.490 1.00 30.44
N
ATOM 2029 CA SER A 298 14.457 32.364 25.439 1.00 30.65
C
ATOM 2030 C SER A 298 13.441 31.243 25.120 1.00 30.99
C
ATOM 2031 O SER A 298 12.449 31.529 24.452 1.00 31.34
O
ATOM 2032 CB SER A 298 15.242 32.813 24.188 1.00 31.86

C
ATOM 2033 OG SER A 298 15.968 31.747 23.605 1.00 33.35
O
ATOM 2034 H SER A 298 16.353 31.818 26.164 1.00 0.00
H
ATOM 2035 HA SER A 298 13.879 33.223 25.787 1.00 0.00
H
ATOM 2036 HB3 SER A 298 15.931 33.620 24.439 1.00 0.00
H
ATOM 2037 HB2 SER A 298 14.558 33.213 23.438 1.00 0.00
H
ATOM 2038 HG SER A 298 16.406 32.070 22.813 1.00 0.00
H
ATOM 2039 N ASP A 299 13.662 30.000 25.564 1.00 29.88
N
ATOM 2040 CA ASP A 299 12.735 28.888 25.316 1.00 29.41
C
ATOM 2041 C ASP A 299 11.605 28.863 26.350 1.00 28.20
C
ATOM 2042 O ASP A 299 10.479 28.509 26.008 1.00 28.75
O
ATOM 2043 CB ASP A 299 13.424 27.509 25.222 1.00 31.09
C
ATOM 2044 CG ASP A 299 14.514 27.421 24.156 1.00 34.22
C
ATOM 2045 OD1 ASP A 299 14.398 28.108 23.120 1.00 34.74
O
ATOM 2046 OD2 ASP A 299 15.384 26.539 24.301 1.00 36.25
O1-
ATOM 2047 H ASP A 299 14.440 29.812 26.183 1.00 0.00
H
ATOM 2048 HA ASP A 299 12.249 29.052 24.352 1.00 0.00
H
ATOM 2049 HB3 ASP A 299 12.681 26.742 25.004 1.00 0.00
H
ATOM 2050 HB2 ASP A 299 13.860 27.265 26.191 1.00 0.00

H
ATOM 2051 N VAL A 300 11.904 29.264 27.587 1.00 27.61
N
ATOM 2052 CA VAL A 300 10.917 29.344 28.659 1.00 26.53
C
ATOM 2053 C VAL A 300 10.013 30.602 28.560 1.00 26.97
C
ATOM 2054 O VAL A 300 8.856 30.533 28.982 1.00 26.32
O
ATOM 2055 CB VAL A 300 11.602 29.238 30.045 1.00 27.74
C
ATOM 2056 CG1 VAL A 300 12.170 27.828 30.289 1.00 29.57
C
ATOM 2057 CG2 VAL A 300 12.714 30.266 30.258 1.00 27.69
C
ATOM 2058 H VAL A 300 12.855 29.523 27.814 1.00 0.00
H
ATOM 2059 HA VAL A 300 10.246 28.487 28.564 1.00 0.00
H
ATOM 2060 HB VAL A 300 10.845 29.425 30.805 1.00 0.00
H
ATOM 2061 HG11 VAL A 300 12.620 27.752 31.279 1.00 0.00
H
ATOM 2062 HG12 VAL A 300 11.391 27.072 30.228 1.00 0.00
H
ATOM 2063 HG13 VAL A 300 12.939 27.570 29.559 1.00 0.00
H
ATOM 2064 HG21 VAL A 300 13.089 30.228 31.279 1.00 0.00
H
ATOM 2065 HG22 VAL A 300 13.559 30.065 29.604 1.00 0.00
H
ATOM 2066 HG23 VAL A 300 12.360 31.275 30.057 1.00 0.00
H
ATOM 2067 N THR A 301 10.478 31.686 27.909 1.00 26.55
N
ATOM 2068 CA THR A 301 9.615 32.823 27.558 1.00 27.35

C
ATOM 2069 C THR A 301 8.590 32.426 26.480 1.00 27.20
C
ATOM 2070 O THR A 301 7.407 32.727 26.631 1.00 27.90
O
ATOM 2071 CB THR A 301 10.416 34.047 27.053 1.00 27.90
C
ATOM 2072 OG1 THR A 301 11.185 33.799 25.888 1.00 29.74
O
ATOM 2073 CG2 THR A 301 11.354 34.611 28.118 1.00 29.44
C
ATOM 2074 H THR A 301 11.438 31.740 27.595 1.00 0.00
H
ATOM 2075 HA THR A 301 9.057 33.127 28.445 1.00 0.00
H
ATOM 2076 HB THR A 301 9.700 34.824 26.785 1.00 0.00
H
ATOM 2077 HG1 THR A 301 10.613 33.867 25.109 1.00 0.00
H
ATOM 2078 HG21 THR A 301 11.894 35.471 27.727 1.00 0.00
H
ATOM 2079 HG22 THR A 301 10.795 34.932 28.996 1.00 0.00
H
ATOM 2080 HG23 THR A 301 12.083 33.874 28.447 1.00 0.00
H
ATOM 2081 N LYS A 302 9.013 31.610 25.500 1.00 26.49
N
ATOM 2082 CA LYS A 302 8.142 30.945 24.522 1.00 27.28
C
ATOM 2083 C LYS A 302 7.139 29.945 25.148 1.00 27.81
C
ATOM 2084 O LYS A 302 6.445 29.255 24.406 1.00 27.95
O
ATOM 2085 CB LYS A 302 8.996 30.263 23.427 1.00 28.36
C
ATOM 2086 CG LYS A 302 9.782 31.235 22.533 1.00 29.79

C
ATOM 2087 CD LYS A 302 10.677 30.494 21.522 1.00 30.72
C
ATOM 2088 CE LYS A 302 11.641 31.416 20.758 1.00 32.93
C
ATOM 2089 NZ LYS A 302 12.775 31.833 21.594 1.00 33.88
N1+
ATOM 2090 H LYS A 302 9.998 31.379 25.459 1.00 0.00
H
ATOM 2091 HA LYS A 302 7.539 31.721 24.046 1.00 0.00
H
ATOM 2092 HB3 LYS A 302 8.351 29.672 22.776 1.00 0.00
H
ATOM 2093 HB2 LYS A 302 9.678 29.550 23.886 1.00 0.00
H
ATOM 2094 HG3 LYS A 302 10.376 31.912 23.145 1.00 0.00
H
ATOM 2095 HG2 LYS A 302 9.077 31.871 21.997 1.00 0.00
H
ATOM 2096 HD3 LYS A 302 10.040 29.982 20.803 1.00 0.00
H
ATOM 2097 HD2 LYS A 302 11.246 29.712 22.030 1.00 0.00
H
ATOM 2098 HE3 LYS A 302 11.118 32.298 20.393 1.00 0.00
H
ATOM 2099 HE2 LYS A 302 12.044 30.903 19.885 1.00 0.00
H
ATOM 2100 HZ1 LYS A 302 13.307 32.538 21.103 1.00 0.00
H
ATOM 2101 HZ2 LYS A 302 12.428 32.219 22.461 1.00 0.00
H
ATOM 2102 HZ3 LYS A 302 13.362 31.039 21.806 1.00 0.00
H
ATOM 2103 N ALA A 303 7.062 29.866 26.479 1.00 26.44
N
ATOM 2104 CA ALA A 303 6.106 29.076 27.231 1.00 27.88

C
ATOM 2105 C ALA A 303 5.374 29.883 28.319 1.00 28.91
C
ATOM 2106 O ALA A 303 4.613 29.278 29.070 1.00 29.81
O
ATOM 2107 CB ALA A 303 6.844 27.885 27.822 1.00 27.38
C
ATOM 2108 H ALA A 303 7.685 30.434 27.039 1.00 0.00
H
ATOM 2109 HA ALA A 303 5.328 28.707 26.563 1.00 0.00
H
ATOM 2110 HB1 ALA A 303 6.171 27.275 28.417 1.00 0.00
H
ATOM 2111 HB2 ALA A 303 7.274 27.252 27.053 1.00 0.00
H
ATOM 2112 HB3 ALA A 303 7.654 28.235 28.456 1.00 0.00
H
ATOM 2113 N GLY A 304 5.535 31.214 28.360 1.00 29.66
N
ATOM 2114 CA GLY A 304 4.654 32.072 29.158 1.00 30.57
C
ATOM 2115 C GLY A 304 5.256 32.551 30.481 1.00 31.32
C
ATOM 2116 O GLY A 304 4.541 33.223 31.222 1.00 32.07
O
ATOM 2117 H GLY A 304 6.183 31.675 27.728 1.00 0.00
H
ATOM 2118 HA3 GLY A 304 3.715 31.572 29.389 1.00 0.00
H
ATOM 2119 HA2 GLY A 304 4.377 32.947 28.580 1.00 0.00
H
ATOM 2120 N HIS A 305 6.521 32.241 30.795 1.00 31.10
N
ATOM 2121 CA HIS A 305 7.176 32.728 32.012 1.00 30.95
C
ATOM 2122 C HIS A 305 8.348 33.635 31.628 1.00 31.77

C
ATOM 2123 O HIS A 305 9.317 33.187 31.017 1.00 31.65
O
ATOM 2124 CB HIS A 305 7.628 31.565 32.900 1.00 30.92
C
ATOM 2125 CG HIS A 305 6.516 30.890 33.676 1.00 30.70
C
ATOM 2126 ND1 HIS A 305 6.398 30.993 35.051 1.00 31.24
N
ATOM 2127 CD2 HIS A 305 5.467 30.087 33.285 1.00 31.47
C
ATOM 2128 CE1 HIS A 305 5.344 30.269 35.429 1.00 31.89
C
ATOM 2129 NE2 HIS A 305 4.724 29.700 34.405 1.00 30.48
N
ATOM 2130 H HIS A 305 7.103 31.718 30.151 1.00 0.00
H
ATOM 2131 HA HIS A 305 6.494 33.324 32.624 1.00 0.00
H
ATOM 2132 HB3 HIS A 305 8.377 31.909 33.616 1.00 0.00
H
ATOM 2133 HB2 HIS A 305 8.130 30.838 32.272 1.00 0.00
H
ATOM 2134 HD1 HIS A 305 6.931 31.640 35.642 1.00 0.00
H
ATOM 2135 HD2 HIS A 305 5.195 29.759 32.291 1.00 0.00
H
ATOM 2136 HE1 HIS A 305 5.007 30.179 36.453 1.00 0.00
H
ATOM 2137 N SER A 306 8.203 34.908 31.989 1.00 32.01
N
ATOM 2138 CA SER A 306 9.123 36.012 31.707 1.00 33.97
C
ATOM 2139 C SER A 306 10.436 35.951 32.514 1.00 34.00
C
ATOM 2140 O SER A 306 10.536 35.204 33.489 1.00 33.09

O
ATOM 2141 CB SER A 306 8.358 37.336 31.934 1.00 34.42
C
ATOM 2142 OG SER A 306 8.115 37.569 33.310 1.00 37.62
O
ATOM 2143 H SER A 306 7.348 35.173 32.457 1.00 0.00
H
ATOM 2144 HA SER A 306 9.392 35.950 30.653 1.00 0.00
H
ATOM 2145 HB3 SER A 306 7.410 37.329 31.395 1.00 0.00
H
ATOM 2146 HB2 SER A 306 8.930 38.176 31.540 1.00 0.00
H
ATOM 2147 HG SER A 306 7.776 38.465 33.430 1.00 0.00
H
ATOM 2148 N LEU A 307 11.413 36.778 32.095 1.00 34.07
N
ATOM 2149 CA LEU A 307 12.737 36.938 32.717 1.00 34.93
C
ATOM 2150 C LEU A 307 12.709 37.397 34.191 1.00 33.80
C
ATOM 2151 O LEU A 307 13.708 37.213 34.885 1.00 33.69
O
ATOM 2152 CB LEU A 307 13.620 37.876 31.854 1.00 36.84
C
ATOM 2153 CG LEU A 307 14.409 37.164 30.728 1.00 39.29
C
ATOM 2154 CD1 LEU A 307 13.505 36.584 29.622 1.00 40.58
C
ATOM 2155 CD2 LEU A 307 15.507 38.083 30.150 1.00 40.01
C
ATOM 2156 H LEU A 307 11.244 37.357 31.286 1.00 0.00
H
ATOM 2157 HA LEU A 307 13.197 35.953 32.730 1.00 0.00
H
ATOM 2158 HB3 LEU A 307 14.356 38.352 32.503 1.00 0.00

H
ATOM 2159 HB2 LEU A 307 13.028 38.698 31.449 1.00 0.00
H
ATOM 2160 HG LEU A 307 14.932 36.324 31.187 1.00 0.00
H
ATOM 2161 HD11 LEU A 307 13.887 36.772 28.619 1.00 0.00
H
ATOM 2162 HD12 LEU A 307 13.429 35.502 29.726 1.00 0.00
H
ATOM 2163 HD13 LEU A 307 12.496 36.992 29.655 1.00 0.00
H
ATOM 2164 HD21 LEU A 307 16.487 37.610 30.231 1.00 0.00
H
ATOM 2165 HD22 LEU A 307 15.351 38.324 29.099 1.00 0.00
H
ATOM 2166 HD23 LEU A 307 15.570 39.034 30.680 1.00 0.00
H
ATOM 2167 N GLU A 308 11.558 37.913 34.656 1.00 33.38
N
ATOM 2168 CA GLU A 308 11.245 38.226 36.052 1.00 32.81
C
ATOM 2169 C GLU A 308 11.295 36.994 36.983 1.00 31.88
C
ATOM 2170 O GLU A 308 11.591 37.163 38.164 1.00 30.22
O
ATOM 2171 CB GLU A 308 9.857 38.898 36.103 1.00 37.03
C
ATOM 2172 CG GLU A 308 9.758 40.184 35.256 1.00 42.70
C
ATOM 2173 CD GLU A 308 8.331 40.725 35.205 1.00 45.49
C
ATOM 2174 OE1 GLU A 308 7.571 40.231 34.342 1.00 46.94
O
ATOM 2175 OE2 GLU A 308 8.028 41.622 36.020 1.00 47.58
O1-
ATOM 2176 H GLU A 308 10.787 38.023 34.013 1.00 0.00

H

ATOM	2177	HA	GLU	A	308	11.990	38.941	36.407	1.00	0.00	H
ATOM	2178	HB3	GLU	A	308	9.605	39.134	37.138	1.00	0.00	H
ATOM	2179	HB2	GLU	A	308	9.100	38.188	35.771	1.00	0.00	H
ATOM	2180	HG3	GLU	A	308	10.077	40.007	34.229	1.00	0.00	H
ATOM	2181	HG2	GLU	A	308	10.423	40.949	35.658	1.00	0.00	H
ATOM	2182	N	LEU	A	309	11.117	35.776	36.435	1.00	29.69	N
ATOM	2183	CA	LEU	A	309	11.426	34.524	37.139	1.00	29.34	C
ATOM	2184	C	LEU	A	309	12.749	33.919	36.672	1.00	28.31	C
ATOM	2185	O	LEU	A	309	13.508	33.439	37.518	1.00	26.92	O
ATOM	2186	CB	LEU	A	309	10.287	33.496	36.987	1.00	28.48	C
ATOM	2187	CG	LEU	A	309	10.473	32.180	37.800	1.00	28.85	C
ATOM	2188	CD1	LEU	A	309	9.144	31.623	38.332	1.00	29.55	C
ATOM	2189	CD2	LEU	A	309	11.268	31.085	37.055	1.00	29.07	C
ATOM	2190	H	LEU	A	309	10.887	35.708	35.451	1.00	0.00	H
ATOM	2191	HA	LEU	A	309	11.536	34.720	38.207	1.00	0.00	H
ATOM	2192	HB3	LEU	A	309	10.089	33.271	35.938	1.00	0.00	H
ATOM	2193	HB2	LEU	A	309	9.402	34.014	37.338	1.00	0.00	H
ATOM	2194	HG	LEU	A	309	11.045	32.443	38.693	1.00	0.00	

H
ATOM 2195 HD11 LEU A 309 9.264 31.305 39.367 1.00 0.00
H
ATOM 2196 HD12 LEU A 309 8.351 32.367 38.324 1.00 0.00
H
ATOM 2197 HD13 LEU A 309 8.780 30.770 37.759 1.00 0.00
H
ATOM 2198 HD21 LEU A 309 10.749 30.127 37.014 1.00 0.00
H
ATOM 2199 HD22 LEU A 309 11.496 31.362 36.029 1.00 0.00
H
ATOM 2200 HD23 LEU A 309 12.217 30.906 37.560 1.00 0.00
H
ATOM 2201 N ILE A 310 13.007 33.907 35.354 1.00 28.87
N
ATOM 2202 CA ILE A 310 14.119 33.122 34.814 1.00 30.36
C
ATOM 2203 C ILE A 310 15.489 33.655 35.282 1.00 30.97
C
ATOM 2204 O ILE A 310 16.346 32.846 35.630 1.00 30.03
O
ATOM 2205 CB ILE A 310 14.160 33.025 33.260 1.00 31.31
C
ATOM 2206 CG1 ILE A 310 12.778 32.901 32.590 1.00 32.43
C
ATOM 2207 CG2 ILE A 310 15.065 31.866 32.790 1.00 32.83
C
ATOM 2208 CD1 ILE A 310 11.828 31.833 33.160 1.00 33.95
C
ATOM 2209 H ILE A 310 12.324 34.266 34.700 1.00 0.00
H
ATOM 2210 HA ILE A 310 14.014 32.109 35.209 1.00 0.00
H
ATOM 2211 HB ILE A 310 14.603 33.942 32.865 1.00 0.00
H
ATOM 2212 HG13 ILE A 310 12.904 32.767 31.518 1.00 0.00

H
ATOM 2213 HG12 ILE A 310 12.289 33.860 32.654 1.00 0.00
H
ATOM 2214 HG21 ILE A 310 15.039 31.761 31.708 1.00 0.00
H
ATOM 2215 HG22 ILE A 310 16.106 32.023 33.068 1.00 0.00
H
ATOM 2216 HG23 ILE A 310 14.750 30.911 33.213 1.00 0.00
H
ATOM 2217 HD11 ILE A 310 11.184 31.432 32.377 1.00 0.00
H
ATOM 2218 HD12 ILE A 310 12.359 30.997 33.614 1.00 0.00
H
ATOM 2219 HD13 ILE A 310 11.165 32.264 33.910 1.00 0.00
H
ATOM 2220 N GLU A 311 15.676 34.983 35.339 1.00 31.83
N
ATOM 2221 CA GLU A 311 16.973 35.547 35.736 1.00 33.28
C
ATOM 2222 C GLU A 311 17.347 35.329 37.223 1.00 31.76
C
ATOM 2223 O GLU A 311 18.494 34.952 37.467 1.00 31.24
O
ATOM 2224 CB GLU A 311 17.121 37.012 35.281 1.00 35.65
C
ATOM 2225 CG GLU A 311 17.108 37.171 33.749 1.00 41.13
C
ATOM 2226 CD GLU A 311 17.297 38.622 33.304 1.00 43.32
C
ATOM 2227 OE1 GLU A 311 18.137 38.832 32.403 1.00 46.68
O
ATOM 2228 OE2 GLU A 311 16.588 39.493 33.854 1.00 45.68
O1-
ATOM 2229 H GLU A 311 14.949 35.644 35.096 1.00 0.00
H
ATOM 2230 HA GLU A 311 17.727 34.991 35.173 1.00 0.00

H
ATOM 2231 HB3 GLU A 311 18.050 37.427 35.678 1.00 0.00
H
ATOM 2232 HB2 GLU A 311 16.319 37.617 35.701 1.00 0.00
H
ATOM 2233 HG3 GLU A 311 16.164 36.812 33.338 1.00 0.00
H
ATOM 2234 HG2 GLU A 311 17.893 36.558 33.305 1.00 0.00
H
ATOM 2235 N PRO A 312 16.375 35.411 38.166 1.00 30.94
N
ATOM 2236 CA PRO A 312 16.517 34.826 39.514 1.00 29.89
C
ATOM 2237 C PRO A 312 16.772 33.307 39.570 1.00 28.28
C
ATOM 2238 O PRO A 312 17.497 32.870 40.462 1.00 28.14
O
ATOM 2239 CB PRO A 312 15.202 35.200 40.215 1.00 30.62
C
ATOM 2240 CG PRO A 312 14.782 36.493 39.546 1.00 31.93
C
ATOM 2241 CD PRO A 312 15.186 36.266 38.096 1.00 31.29
C
ATOM 2242 HA PRO A 312 17.342 35.343 40.007 1.00 0.00
H
ATOM 2243 HB3 PRO A 312 15.310 35.304 41.295 1.00 0.00
H
ATOM 2244 HB2 PRO A 312 14.437 34.445 40.026 1.00 0.00
H
ATOM 2245 HG3 PRO A 312 15.360 37.320 39.962 1.00 0.00
H
ATOM 2246 HG2 PRO A 312 13.725 36.724 39.675 1.00 0.00
H
ATOM 2247 HD2 PRO A 312 14.391 35.755 37.561 1.00 0.00
H
ATOM 2248 HD3 PRO A 312 15.364 37.221 37.605 1.00 0.00

H
ATOM 2249 N LEU A 313 16.178 32.522 38.654 1.00 26.85
N
ATOM 2250 CA LEU A 313 16.385 31.071 38.564 1.00 26.51
C
ATOM 2251 C LEU A 313 17.831 30.747 38.148 1.00 25.16
C
ATOM 2252 O LEU A 313 18.499 29.975 38.832 1.00 24.33
O
ATOM 2253 CB LEU A 313 15.366 30.417 37.591 1.00 27.97
C
ATOM 2254 CG LEU A 313 14.647 29.146 38.110 1.00 31.36
C
ATOM 2255 CD1 LEU A 313 13.744 28.551 37.006 1.00 29.96
C
ATOM 2256 CD2 LEU A 313 15.583 28.072 38.708 1.00 30.03
C
ATOM 2257 H LEU A 313 15.517 32.925 37.999 1.00 0.00
H
ATOM 2258 HA LEU A 313 16.228 30.663 39.564 1.00 0.00
H
ATOM 2259 HB3 LEU A 313 15.847 30.194 36.639 1.00 0.00
H
ATOM 2260 HB2 LEU A 313 14.593 31.140 37.341 1.00 0.00
H
ATOM 2261 HG LEU A 313 13.991 29.474 38.918 1.00 0.00
H
ATOM 2262 HD11 LEU A 313 12.735 28.365 37.372 1.00 0.00
H
ATOM 2263 HD12 LEU A 313 13.649 29.216 36.146 1.00 0.00
H
ATOM 2264 HD13 LEU A 313 14.125 27.604 36.623 1.00 0.00
H
ATOM 2265 HD21 LEU A 313 15.312 27.062 38.401 1.00 0.00
H
ATOM 2266 HD22 LEU A 313 16.618 28.222 38.414 1.00 0.00

H
ATOM 2267 HD23 LEU A 313 15.542 28.090 39.798 1.00 0.00
H
ATOM 2268 N ILE A 314 18.306 31.366 37.059 1.00 25.12
N
ATOM 2269 CA ILE A 314 19.659 31.179 36.529 1.00 25.36
C
ATOM 2270 C ILE A 314 20.739 31.616 37.544 1.00 25.44
C
ATOM 2271 O ILE A 314 21.706 30.883 37.756 1.00 25.07
O
ATOM 2272 CB ILE A 314 19.853 31.931 35.175 1.00 26.51
C
ATOM 2273 CG1 ILE A 314 18.910 31.399 34.067 1.00 28.21
C
ATOM 2274 CG2 ILE A 314 21.308 32.002 34.655 1.00 26.75
C
ATOM 2275 CD1 ILE A 314 19.265 30.020 33.498 1.00 29.90
C
ATOM 2276 H ILE A 314 17.682 31.980 36.538 1.00 0.00
H
ATOM 2277 HA ILE A 314 19.800 30.110 36.359 1.00 0.00
H
ATOM 2278 HB ILE A 314 19.547 32.965 35.349 1.00 0.00
H
ATOM 2279 HG13 ILE A 314 18.883 32.119 33.249 1.00 0.00
H
ATOM 2280 HG12 ILE A 314 17.889 31.342 34.434 1.00 0.00
H
ATOM 2281 HG21 ILE A 314 21.350 32.457 33.666 1.00 0.00
H
ATOM 2282 HG22 ILE A 314 21.950 32.602 35.301 1.00 0.00
H
ATOM 2283 HG23 ILE A 314 21.755 31.011 34.583 1.00 0.00
H
ATOM 2284 HD11 ILE A 314 18.545 29.722 32.735 1.00 0.00

H
ATOM 2285 HD12 ILE A 314 20.247 30.018 33.025 1.00 0.00
H
ATOM 2286 HD13 ILE A 314 19.248 29.254 34.272 1.00 0.00
H
ATOM 2287 N LYS A 315 20.511 32.753 38.222 1.00 25.44
N
ATOM 2288 CA LYS A 315 21.339 33.225 39.334 1.00 26.52
C
ATOM 2289 C LYS A 315 21.362 32.234 40.516 1.00 25.66
C
ATOM 2290 O LYS A 315 22.417 32.063 41.128 1.00 25.67
O
ATOM 2291 CB LYS A 315 20.861 34.634 39.745 1.00 28.77
C
ATOM 2292 CG LYS A 315 21.688 35.306 40.857 1.00 34.86
C
ATOM 2293 CD LYS A 315 21.249 36.758 41.111 1.00 37.34
C
ATOM 2294 CE LYS A 315 22.096 37.485 42.167 1.00 39.12
C
ATOM 2295 NZ LYS A 315 21.946 36.886 43.505 1.00 41.56
N1+
ATOM 2296 H LYS A 315 19.679 33.295 38.029 1.00 0.00
H
ATOM 2297 HA LYS A 315 22.361 33.312 38.960 1.00 0.00
H
ATOM 2298 HB3 LYS A 315 19.814 34.594 40.050 1.00 0.00
H
ATOM 2299 HB2 LYS A 315 20.892 35.275 38.862 1.00 0.00
H
ATOM 2300 HG3 LYS A 315 22.743 35.286 40.583 1.00 0.00
H
ATOM 2301 HG2 LYS A 315 21.597 34.734 41.780 1.00 0.00
H
ATOM 2302 HD3 LYS A 315 20.200 36.776 41.407 1.00 0.00

H
ATOM 2303 HD2 LYS A 315 21.303 37.317 40.175 1.00 0.00
H
ATOM 2304 HE3 LYS A 315 21.794 38.531 42.225 1.00 0.00
H
ATOM 2305 HE2 LYS A 315 23.149 37.474 41.885 1.00 0.00
H
ATOM 2306 HZ1 LYS A 315 22.248 35.923 43.480 1.00 0.00
H
ATOM 2307 HZ2 LYS A 315 22.510 37.398 44.169 1.00 0.00
H
ATOM 2308 HZ3 LYS A 315 20.977 36.927 43.787 1.00 0.00
H
ATOM 2309 N PHE A 316 20.214 31.610 40.812 1.00 23.92
N
ATOM 2310 CA PHE A 316 20.110 30.544 41.801 1.00 23.22
C
ATOM 2311 C PHE A 316 20.929 29.302 41.401 1.00 22.36
C
ATOM 2312 O PHE A 316 21.723 28.826 42.210 1.00 22.35
O
ATOM 2313 CB PHE A 316 18.632 30.255 42.159 1.00 24.22
C
ATOM 2314 CG PHE A 316 18.422 29.041 43.043 1.00 26.30
C
ATOM 2315 CD1 PHE A 316 18.661 29.136 44.427 1.00 27.43
C
ATOM 2316 CD2 PHE A 316 18.209 27.771 42.470 1.00 26.87
C
ATOM 2317 CE1 PHE A 316 18.637 27.995 45.210 1.00 28.46
C
ATOM 2318 CE2 PHE A 316 18.219 26.634 43.266 1.00 27.51
C
ATOM 2319 CZ PHE A 316 18.443 26.749 44.632 1.00 28.27
C
ATOM 2320 H PHE A 316 19.367 31.833 40.303 1.00 0.00

H
ATOM 2321 HA PHE A 316 20.573 30.916 42.717 1.00 0.00
H
ATOM 2322 HB3 PHE A 316 18.024 30.138 41.265 1.00 0.00
H
ATOM 2323 HB2 PHE A 316 18.220 31.121 42.680 1.00 0.00
H
ATOM 2324 HD1 PHE A 316 18.870 30.093 44.881 1.00 0.00
H
ATOM 2325 HD2 PHE A 316 18.072 27.689 41.405 1.00 0.00
H
ATOM 2326 HE1 PHE A 316 18.777 28.086 46.275 1.00 0.00
H
ATOM 2327 HE2 PHE A 316 18.067 25.661 42.823 1.00 0.00
H
ATOM 2328 HZ PHE A 316 18.453 25.870 45.257 1.00 0.00
H
ATOM 2329 N GLN A 317 20.788 28.841 40.149 1.00 21.28
N
ATOM 2330 CA GLN A 317 21.508 27.674 39.630 1.00 21.35
C
ATOM 2331 C GLN A 317 23.035 27.840 39.632 1.00 21.92
C
ATOM 2332 O GLN A 317 23.738 26.919 40.051 1.00 20.68
O
ATOM 2333 CB GLN A 317 21.009 27.284 38.221 1.00 21.98
C
ATOM 2334 CG GLN A 317 19.553 26.791 38.125 1.00 22.78
C
ATOM 2335 CD GLN A 317 19.191 25.736 39.166 1.00 25.08
C
ATOM 2336 OE1 GLN A 317 19.979 24.842 39.459 1.00 26.08
O
ATOM 2337 NE2 GLN A 317 17.990 25.804 39.723 1.00 22.14
N
ATOM 2338 H GLN A 317 20.112 29.289 39.534 1.00 0.00

H
ATOM 2339 HA GLN A 317 21.315 26.861 40.329 1.00 0.00
H
ATOM 2340 HB3 GLN A 317 21.643 26.478 37.851 1.00 0.00
H
ATOM 2341 HB2 GLN A 317 21.152 28.112 37.526 1.00 0.00
H
ATOM 2342 HG3 GLN A 317 19.383 26.370 37.134 1.00 0.00
H
ATOM 2343 HG2 GLN A 317 18.874 27.633 38.214 1.00 0.00
H
ATOM 2344 HE22 GLN A 317 17.695 25.052 40.339 1.00 0.00
H
ATOM 2345 HE21 GLN A 317 17.330 26.511 39.447 1.00 0.00
H
ATOM 2346 N VAL A 318 23.516 29.027 39.240 1.00 22.58
N
ATOM 2347 CA VAL A 318 24.919 29.433 39.356 1.00 23.76
C
ATOM 2348 C VAL A 318 25.424 29.421 40.816 1.00 24.71
C
ATOM 2349 O VAL A 318 26.548 28.980 41.052 1.00 26.38
O
ATOM 2350 CB VAL A 318 25.133 30.844 38.726 1.00 24.56
C
ATOM 2351 CG1 VAL A 318 26.456 31.553 39.089 1.00 26.05
C
ATOM 2352 CG2 VAL A 318 25.008 30.776 37.194 1.00 23.60
C
ATOM 2353 H VAL A 318 22.877 29.740 38.913 1.00 0.00
H
ATOM 2354 HA VAL A 318 25.516 28.710 38.797 1.00 0.00
H
ATOM 2355 HB VAL A 318 24.326 31.489 39.077 1.00 0.00
H
ATOM 2356 HG11 VAL A 318 26.578 32.473 38.517 1.00 0.00

H
ATOM 2357 HG12 VAL A 318 26.500 31.834 40.142 1.00 0.00
H
ATOM 2358 HG13 VAL A 318 27.312 30.915 38.873 1.00 0.00
H
ATOM 2359 HG21 VAL A 318 25.020 31.772 36.750 1.00 0.00
H
ATOM 2360 HG22 VAL A 318 25.833 30.213 36.763 1.00 0.00
H
ATOM 2361 HG23 VAL A 318 24.089 30.289 36.874 1.00 0.00
H
ATOM 2362 N GLY A 319 24.576 29.856 41.765 1.00 25.34
N
ATOM 2363 CA GLY A 319 24.881 29.863 43.196 1.00 26.42
C
ATOM 2364 C GLY A 319 24.811 28.457 43.820 1.00 26.70
C
ATOM 2365 O GLY A 319 25.467 28.225 44.834 1.00 26.15
O
ATOM 2366 H GLY A 319 23.668 30.205 41.490 1.00 0.00
H
ATOM 2367 HA3 GLY A 319 24.158 30.505 43.699 1.00 0.00
H
ATOM 2368 HA2 GLY A 319 25.867 30.298 43.369 1.00 0.00
H
ATOM 2369 N LEU A 320 24.036 27.534 43.222 1.00 25.08
N
ATOM 2370 CA LEU A 320 23.885 26.139 43.640 1.00 26.49
C
ATOM 2371 C LEU A 320 25.051 25.263 43.137 1.00 26.16
C
ATOM 2372 O LEU A 320 25.481 24.373 43.871 1.00 25.62
O
ATOM 2373 CB LEU A 320 22.498 25.632 43.172 1.00 26.17
C
ATOM 2374 CG LEU A 320 22.077 24.231 43.671 1.00 29.00

C
ATOM 2375 CD1 LEU A 320 22.034 24.145 45.208 1.00 28.44
C
ATOM 2376 CD2 LEU A 320 20.728 23.808 43.055 1.00 27.28
C
ATOM 2377 H LEU A 320 23.494 27.807 42.412 1.00 0.00
H
ATOM 2378 HA LEU A 320 23.906 26.116 44.730 1.00 0.00
H
ATOM 2379 HB3 LEU A 320 22.468 25.651 42.083 1.00 0.00
H
ATOM 2380 HB2 LEU A 320 21.737 26.342 43.498 1.00 0.00
H
ATOM 2381 HG LEU A 320 22.815 23.510 43.316 1.00 0.00
H
ATOM 2382 HD11 LEU A 320 21.223 23.510 45.568 1.00 0.00
H
ATOM 2383 HD12 LEU A 320 22.964 23.730 45.595 1.00 0.00
H
ATOM 2384 HD13 LEU A 320 21.898 25.128 45.659 1.00 0.00
H
ATOM 2385 HD21 LEU A 320 20.743 22.755 42.774 1.00 0.00
H
ATOM 2386 HD22 LEU A 320 19.899 23.945 43.750 1.00 0.00
H
ATOM 2387 HD23 LEU A 320 20.485 24.383 42.160 1.00 0.00
H
ATOM 2388 N LYS A 321 25.582 25.571 41.936 1.00 26.97
N
ATOM 2389 CA LYS A 321 26.824 25.009 41.388 1.00 29.33
C
ATOM 2390 C LYS A 321 28.059 25.375 42.233 1.00 30.36
C
ATOM 2391 O LYS A 321 28.937 24.531 42.406 1.00 30.00
O
ATOM 2392 CB LYS A 321 27.028 25.499 39.935 1.00 30.33

C
ATOM 2393 CG LYS A 321 26.089 24.886 38.881 1.00 33.36
C
ATOM 2394 CD LYS A 321 26.422 23.437 38.496 1.00 33.34
C
ATOM 2395 CE LYS A 321 25.568 22.955 37.314 1.00 33.36
C
ATOM 2396 NZ LYS A 321 25.838 21.547 36.983 1.00 32.77
N1+
ATOM 2397 H LYS A 321 25.151 26.301 41.385 1.00 0.00
H
ATOM 2398 HA LYS A 321 26.727 23.923 41.390 1.00 0.00
H
ATOM 2399 HB3 LYS A 321 28.052 25.300 39.615 1.00 0.00
H
ATOM 2400 HB2 LYS A 321 26.926 26.585 39.909 1.00 0.00
H
ATOM 2401 HG3 LYS A 321 26.116 25.510 37.987 1.00 0.00
H
ATOM 2402 HG2 LYS A 321 25.063 24.918 39.235 1.00 0.00
H
ATOM 2403 HD3 LYS A 321 26.259 22.784 39.353 1.00 0.00
H
ATOM 2404 HD2 LYS A 321 27.480 23.357 38.242 1.00 0.00
H
ATOM 2405 HE3 LYS A 321 25.765 23.564 36.431 1.00 0.00
H
ATOM 2406 HE2 LYS A 321 24.508 23.063 37.545 1.00 0.00
H
ATOM 2407 HZ1 LYS A 321 25.261 21.283 36.193 1.00 0.00
H
ATOM 2408 HZ2 LYS A 321 26.811 21.424 36.746 1.00 0.00
H
ATOM 2409 HZ3 LYS A 321 25.598 20.962 37.771 1.00 0.00
H
ATOM 2410 N LYS A 322 28.088 26.615 42.755 1.00 30.82

N

ATOM 2411 CA LYS A 322 29.158 27.165 43.592 1.00 32.19
C

ATOM 2412 C LYS A 322 29.239 26.587 45.018 1.00 32.03
C

ATOM 2413 O LYS A 322 30.258 26.806 45.673 1.00 32.87
O

ATOM 2414 CB LYS A 322 29.044 28.703 43.602 1.00 33.78
C

ATOM 2415 CG LYS A 322 29.555 29.344 42.300 1.00 37.24
C

ATOM 2416 CD LYS A 322 29.267 30.852 42.228 1.00 39.61
C

ATOM 2417 CE LYS A 322 29.784 31.510 40.940 1.00 41.25
C

ATOM 2418 NZ LYS A 322 31.256 31.509 40.873 1.00 43.80
N1+

ATOM 2419 H LYS A 322 27.324 27.244 42.553 1.00 0.00
H

ATOM 2420 HA LYS A 322 30.104 26.903 43.113 1.00 0.00
H

ATOM 2421 HB3 LYS A 322 29.634 29.120 44.421 1.00 0.00
H

ATOM 2422 HB2 LYS A 322 28.012 28.996 43.799 1.00 0.00
H

ATOM 2423 HG3 LYS A 322 29.111 28.847 41.438 1.00 0.00
H

ATOM 2424 HG2 LYS A 322 30.628 29.168 42.224 1.00 0.00
H

ATOM 2425 HD3 LYS A 322 29.699 31.352 43.096 1.00 0.00
H

ATOM 2426 HD2 LYS A 322 28.190 31.012 42.295 1.00 0.00
H

ATOM 2427 HE3 LYS A 322 29.438 32.542 40.887 1.00 0.00
H

ATOM 2428 HE2 LYS A 322 29.387 30.995 40.064 1.00 0.00

H
ATOM 2429 HZ1 LYS A 322 31.595 30.557 40.891 1.00 0.00
H
ATOM 2430 HZ2 LYS A 322 31.557 31.955 40.018 1.00 0.00
H
ATOM 2431 HZ3 LYS A 322 31.632 32.013 41.664 1.00 0.00
H
ATOM 2432 N LEU A 323 28.218 25.831 45.465 1.00 30.51
N
ATOM 2433 CA LEU A 323 28.282 25.042 46.701 1.00 31.09
C
ATOM 2434 C LEU A 323 29.165 23.787 46.547 1.00 31.05
C
ATOM 2435 O LEU A 323 29.756 23.359 47.539 1.00 31.05
O
ATOM 2436 CB LEU A 323 26.867 24.625 47.153 1.00 30.29
C
ATOM 2437 CG LEU A 323 25.884 25.765 47.490 1.00 31.62
C
ATOM 2438 CD1 LEU A 323 24.526 25.180 47.934 1.00 30.41
C
ATOM 2439 CD2 LEU A 323 26.446 26.764 48.521 1.00 31.32
C
ATOM 2440 H LEU A 323 27.402 25.694 44.885 1.00 0.00
H
ATOM 2441 HA LEU A 323 28.735 25.656 47.481 1.00 0.00
H
ATOM 2442 HB3 LEU A 323 26.957 23.991 48.036 1.00 0.00
H
ATOM 2443 HB2 LEU A 323 26.418 24.004 46.378 1.00 0.00
H
ATOM 2444 HG LEU A 323 25.701 26.318 46.570 1.00 0.00
H
ATOM 2445 HD11 LEU A 323 23.738 25.483 47.247 1.00 0.00
H
ATOM 2446 HD12 LEU A 323 24.531 24.090 47.943 1.00 0.00

H
ATOM 2447 HD13 LEU A 323 24.228 25.499 48.933 1.00 0.00
H
ATOM 2448 HD21 LEU A 323 25.680 27.130 49.202 1.00 0.00
H
ATOM 2449 HD22 LEU A 323 27.235 26.324 49.131 1.00 0.00
H
ATOM 2450 HD23 LEU A 323 26.866 27.636 48.018 1.00 0.00
H
ATOM 2451 N ASN A 324 29.243 23.238 45.317 1.00 30.91
N
ATOM 2452 CA ASN A 324 30.031 22.062 44.910 1.00 32.42
C
ATOM 2453 C ASN A 324 29.665 20.810 45.734 1.00 32.07
C
ATOM 2454 O ASN A 324 30.539 20.182 46.333 1.00 32.62
O
ATOM 2455 CB ASN A 324 31.549 22.385 44.933 1.00 36.14
C
ATOM 2456 CG ASN A 324 31.948 23.460 43.917 1.00 38.53
C
ATOM 2457 OD1 ASN A 324 31.936 23.216 42.713 1.00 43.04
O
ATOM 2458 ND2 ASN A 324 32.315 24.651 44.395 1.00 40.87
N
ATOM 2459 H ASN A 324 28.734 23.683 44.565 1.00 0.00
H
ATOM 2460 HA ASN A 324 29.736 21.866 43.877 1.00 0.00
H
ATOM 2461 HB3 ASN A 324 32.119 21.492 44.676 1.00 0.00
H
ATOM 2462 HB2 ASN A 324 31.874 22.673 45.934 1.00 0.00
H
ATOM 2463 HD22 ASN A 324 32.596 25.383 43.760 1.00 0.00
H
ATOM 2464 HD21 ASN A 324 32.296 24.835 45.388 1.00 0.00

H
ATOM 2465 N LEU A 325 28.357 20.508 45.777 1.00 29.80
N
ATOM 2466 CA LEU A 325 27.775 19.442 46.591 1.00 27.98
C
ATOM 2467 C LEU A 325 28.043 18.046 46.015 1.00 27.42
C
ATOM 2468 O LEU A 325 28.093 17.876 44.796 1.00 27.50
O
ATOM 2469 CB LEU A 325 26.248 19.654 46.714 1.00 28.15
C
ATOM 2470 CG LEU A 325 25.825 21.017 47.296 1.00 28.88
C
ATOM 2471 CD1 LEU A 325 24.290 21.145 47.334 1.00 29.23
C
ATOM 2472 CD2 LEU A 325 26.466 21.284 48.670 1.00 28.09
C
ATOM 2473 H LEU A 325 27.702 21.070 45.252 1.00 0.00
H
ATOM 2474 HA LEU A 325 28.225 19.495 47.583 1.00 0.00
H
ATOM 2475 HB3 LEU A 325 25.827 18.862 47.336 1.00 0.00
H
ATOM 2476 HB2 LEU A 325 25.788 19.536 45.731 1.00 0.00
H
ATOM 2477 HG LEU A 325 26.182 21.791 46.619 1.00 0.00
H
ATOM 2478 HD11 LEU A 325 23.925 21.477 48.305 1.00 0.00
H
ATOM 2479 HD12 LEU A 325 23.947 21.872 46.599 1.00 0.00
H
ATOM 2480 HD13 LEU A 325 23.792 20.201 47.110 1.00 0.00
H
ATOM 2481 HD21 LEU A 325 25.924 22.044 49.231 1.00 0.00
H
ATOM 2482 HD22 LEU A 325 26.506 20.381 49.280 1.00 0.00

H

ATOM 2483 HD23 LEU A 325 27.488 21.649 48.559 1.00 0.00

H

ATOM 2484 N HIS A 326 28.136 17.068 46.926 1.00 26.59

N

ATOM 2485 CA HIS A 326 28.100 15.637 46.621 1.00 25.79

C

ATOM 2486 C HIS A 326 26.644 15.191 46.383 1.00 25.33

C

ATOM 2487 O HIS A 326 25.720 15.905 46.779 1.00 23.44

O

ATOM 2488 CB HIS A 326 28.730 14.859 47.795 1.00 27.17

C

ATOM 2489 CG HIS A 326 30.173 15.184 48.121 1.00 27.36

C

ATOM 2490 ND1 HIS A 326 30.912 14.409 48.994 1.00 28.09

N

ATOM 2491 CD2 HIS A 326 31.036 16.179 47.705 1.00 28.85

C

ATOM 2492 CE1 HIS A 326 32.134 14.937 49.069 1.00 28.02

C

ATOM 2493 NE2 HIS A 326 32.281 16.020 48.319 1.00 29.20

N

ATOM 2494 H HIS A 326 28.095 17.309 47.910 1.00 0.00

H

ATOM 2495 HA HIS A 326 28.677 15.443 45.714 1.00 0.00

H

ATOM 2496 HB3 HIS A 326 28.679 13.789 47.591 1.00 0.00

H

ATOM 2497 HB2 HIS A 326 28.144 15.019 48.701 1.00 0.00

H

ATOM 2498 HD1 HIS A 326 30.580 13.587 49.488 1.00 0.00

H

ATOM 2499 HD2 HIS A 326 30.863 16.996 47.019 1.00 0.00

H

ATOM 2500 HE1 HIS A 326 32.921 14.522 49.681 1.00 0.00

H
ATOM 2501 N GLU A 327 26.448 14.013 45.760 1.00 24.49
N
ATOM 2502 CA GLU A 327 25.108 13.460 45.513 1.00 24.94
C
ATOM 2503 C GLU A 327 24.354 13.149 46.821 1.00 24.29
C
ATOM 2504 O GLU A 327 23.165 13.446 46.900 1.00 24.42
O
ATOM 2505 CB GLU A 327 25.174 12.231 44.579 1.00 27.30
C
ATOM 2506 CG GLU A 327 23.777 11.776 44.088 1.00 29.69
C
ATOM 2507 CD GLU A 327 23.768 10.518 43.218 1.00 32.63
C
ATOM 2508 OE1 GLU A 327 24.809 9.830 43.137 1.00 31.56
O
ATOM 2509 OE2 GLU A 327 22.679 10.238 42.671 1.00 32.79
O1-
ATOM 2510 H GLU A 327 27.234 13.448 45.468 1.00 0.00
H
ATOM 2511 HA GLU A 327 24.545 14.235 44.988 1.00 0.00
H
ATOM 2512 HB3 GLU A 327 25.673 11.410 45.098 1.00 0.00
H
ATOM 2513 HB2 GLU A 327 25.796 12.464 43.714 1.00 0.00
H
ATOM 2514 HG3 GLU A 327 23.314 12.583 43.519 1.00 0.00
H
ATOM 2515 HG2 GLU A 327 23.111 11.580 44.928 1.00 0.00
H
ATOM 2516 N GLU A 328 25.075 12.623 47.827 1.00 22.79
N
ATOM 2517 CA GLU A 328 24.609 12.354 49.189 1.00 23.11
C
ATOM 2518 C GLU A 328 24.040 13.600 49.894 1.00 23.46

C
ATOM 2519 O GLU A 328 23.011 13.494 50.559 1.00 23.51
O
ATOM 2520 CB GLU A 328 25.748 11.744 50.038 1.00 24.71
C
ATOM 2521 CG GLU A 328 26.358 10.434 49.491 1.00 25.97
C
ATOM 2522 CD GLU A 328 27.617 10.619 48.638 1.00 28.78
C
ATOM 2523 OE1 GLU A 328 28.663 10.081 49.064 1.00 29.81
O
ATOM 2524 OE2 GLU A 328 27.530 11.301 47.594 1.00 27.61
O1-
ATOM 2525 H GLU A 328 26.041 12.365 47.649 1.00 0.00
H
ATOM 2526 HA GLU A 328 23.812 11.613 49.118 1.00 0.00
H
ATOM 2527 HB3 GLU A 328 25.368 11.547 51.041 1.00 0.00
H
ATOM 2528 HB2 GLU A 328 26.536 12.487 50.174 1.00 0.00
H
ATOM 2529 HG3 GLU A 328 25.616 9.885 48.916 1.00 0.00
H
ATOM 2530 HG2 GLU A 328 26.625 9.793 50.331 1.00 0.00
H
ATOM 2531 N GLU A 329 24.699 14.754 49.698 1.00 22.58
N
ATOM 2532 CA GLU A 329 24.278 16.059 50.210 1.00 23.12
C
ATOM 2533 C GLU A 329 23.051 16.613 49.469 1.00 22.73
C
ATOM 2534 O GLU A 329 22.175 17.196 50.108 1.00 22.26
O
ATOM 2535 CB GLU A 329 25.461 17.046 50.123 1.00 23.27
C
ATOM 2536 CG GLU A 329 26.649 16.626 51.005 1.00 24.71

C
ATOM 2537 CD GLU A 329 27.871 17.532 50.868 1.00 26.09
C
ATOM 2538 OE1 GLU A 329 28.551 17.725 51.898 1.00 27.57
O
ATOM 2539 OE2 GLU A 329 28.150 17.985 49.737 1.00 26.68
O1-
ATOM 2540 H GLU A 329 25.528 14.754 49.121 1.00 0.00
H
ATOM 2541 HA GLU A 329 24.006 15.936 51.258 1.00 0.00
H
ATOM 2542 HB3 GLU A 329 25.141 18.046 50.414 1.00 0.00
H
ATOM 2543 HB2 GLU A 329 25.784 17.131 49.085 1.00 0.00
H
ATOM 2544 HG3 GLU A 329 26.965 15.617 50.750 1.00 0.00
H
ATOM 2545 HG2 GLU A 329 26.337 16.604 52.050 1.00 0.00
H
ATOM 2546 N HIS A 330 23.002 16.409 48.143 1.00 22.72
N
ATOM 2547 CA HIS A 330 21.930 16.881 47.272 1.00 22.97
C
ATOM 2548 C HIS A 330 20.596 16.139 47.488 1.00 23.43
C
ATOM 2549 O HIS A 330 19.556 16.795 47.496 1.00 22.10
O
ATOM 2550 CB HIS A 330 22.407 16.818 45.809 1.00 24.13
C
ATOM 2551 CG HIS A 330 21.537 17.594 44.855 1.00 25.22
C
ATOM 2552 ND1 HIS A 330 20.430 17.024 44.222 1.00 25.99
N
ATOM 2553 CD2 HIS A 330 21.637 18.918 44.478 1.00 25.76
C
ATOM 2554 CE1 HIS A 330 19.901 18.009 43.515 1.00 26.73

C
ATOM 2555 NE2 HIS A 330 20.576 19.154 43.622 1.00 25.08
N
ATOM 2556 H HIS A 330 23.762 15.920 47.688 1.00 0.00
H
ATOM 2557 HA HIS A 330 21.768 17.932 47.517 1.00 0.00
H
ATOM 2558 HB3 HIS A 330 22.474 15.783 45.469 1.00 0.00
H
ATOM 2559 HB2 HIS A 330 23.416 17.225 45.731 1.00 0.00
H
ATOM 2560 HD2 HIS A 330 22.346 19.686 44.748 1.00 0.00
H
ATOM 2561 HE1 HIS A 330 19.005 17.894 42.926 1.00 0.00
H
ATOM 2562 HE2 HIS A 330 20.349 20.023 43.158 1.00 0.00
H
ATOM 2563 N VAL A 331 20.640 14.811 47.694 1.00 22.22
N
ATOM 2564 CA VAL A 331 19.454 13.992 47.975 1.00 23.00
C
ATOM 2565 C VAL A 331 18.903 14.191 49.404 1.00 22.12
C
ATOM 2566 O VAL A 331 17.694 14.069 49.593 1.00 21.17
O
ATOM 2567 CB VAL A 331 19.697 12.479 47.722 1.00 22.74
C
ATOM 2568 CG1 VAL A 331 20.031 12.198 46.248 1.00 25.60
C
ATOM 2569 CG2 VAL A 331 20.716 11.813 48.666 1.00 25.97
C
ATOM 2570 H VAL A 331 21.527 14.325 47.652 1.00 0.00
H
ATOM 2571 HA VAL A 331 18.663 14.311 47.297 1.00 0.00
H
ATOM 2572 HB VAL A 331 18.746 11.974 47.890 1.00 0.00

H
ATOM 2573 HG11 VAL A 331 20.172 11.131 46.073 1.00 0.00
H
ATOM 2574 HG12 VAL A 331 19.226 12.537 45.597 1.00 0.00
H
ATOM 2575 HG13 VAL A 331 20.939 12.703 45.926 1.00 0.00
H
ATOM 2576 HG21 VAL A 331 20.851 10.760 48.421 1.00 0.00
H
ATOM 2577 HG22 VAL A 331 21.686 12.290 48.587 1.00 0.00
H
ATOM 2578 HG23 VAL A 331 20.412 11.861 49.711 1.00 0.00
H
ATOM 2579 N LEU A 332 19.770 14.573 50.360 1.00 21.61
N
ATOM 2580 CA LEU A 332 19.369 15.049 51.686 1.00 21.81
C
ATOM 2581 C LEU A 332 18.653 16.410 51.610 1.00 21.82
C
ATOM 2582 O LEU A 332 17.657 16.591 52.306 1.00 22.23
O
ATOM 2583 CB LEU A 332 20.606 15.140 52.605 1.00 22.40
C
ATOM 2584 CG LEU A 332 21.072 13.789 53.189 1.00 22.71
C
ATOM 2585 CD1 LEU A 332 22.535 13.860 53.674 1.00 23.21
C
ATOM 2586 CD2 LEU A 332 20.117 13.275 54.286 1.00 23.85
C
ATOM 2587 H LEU A 332 20.754 14.640 50.140 1.00 0.00
H
ATOM 2588 HA LEU A 332 18.661 14.333 52.108 1.00 0.00
H
ATOM 2589 HB3 LEU A 332 20.412 15.822 53.435 1.00 0.00
H
ATOM 2590 HB2 LEU A 332 21.416 15.599 52.039 1.00 0.00

H
ATOM 2591 HG LEU A 332 21.051 13.064 52.374 1.00 0.00
H
ATOM 2592 HD11 LEU A 332 23.116 13.028 53.282 1.00 0.00
H
ATOM 2593 HD12 LEU A 332 23.034 14.768 53.344 1.00 0.00
H
ATOM 2594 HD13 LEU A 332 22.620 13.831 54.757 1.00 0.00
H
ATOM 2595 HD21 LEU A 332 19.796 12.256 54.076 1.00 0.00
H
ATOM 2596 HD22 LEU A 332 20.577 13.263 55.272 1.00 0.00
H
ATOM 2597 HD23 LEU A 332 19.222 13.891 54.370 1.00 0.00
H
ATOM 2598 N LEU A 333 19.148 17.320 50.751 1.00 21.45
N
ATOM 2599 CA LEU A 333 18.583 18.652 50.523 1.00 22.43
C
ATOM 2600 C LEU A 333 17.208 18.620 49.826 1.00 21.61
C
ATOM 2601 O LEU A 333 16.344 19.412 50.197 1.00 21.39
O
ATOM 2602 CB LEU A 333 19.624 19.513 49.769 1.00 23.63
C
ATOM 2603 CG LEU A 333 19.252 20.998 49.544 1.00 26.05
C
ATOM 2604 CD1 LEU A 333 18.906 21.725 50.859 1.00 26.27
C
ATOM 2605 CD2 LEU A 333 20.367 21.729 48.765 1.00 26.03
C
ATOM 2606 H LEU A 333 19.979 17.095 50.220 1.00 0.00
H
ATOM 2607 HA LEU A 333 18.430 19.093 51.510 1.00 0.00
H
ATOM 2608 HB3 LEU A 333 19.831 19.056 48.802 1.00 0.00

H
ATOM 2609 HB2 LEU A 333 20.562 19.473 50.320 1.00 0.00
H
ATOM 2610 HG LEU A 333 18.365 21.030 48.911 1.00 0.00
H
ATOM 2611 HD11 LEU A 333 19.194 22.773 50.832 1.00 0.00
H
ATOM 2612 HD12 LEU A 333 17.832 21.700 51.045 1.00 0.00
H
ATOM 2613 HD13 LEU A 333 19.402 21.273 51.719 1.00 0.00
H
ATOM 2614 HD21 LEU A 333 19.959 22.259 47.904 1.00 0.00
H
ATOM 2615 HD22 LEU A 333 20.892 22.462 49.378 1.00 0.00
H
ATOM 2616 HD23 LEU A 333 21.121 21.039 48.385 1.00 0.00
H
ATOM 2617 N MET A 334 17.020 17.695 48.870 1.00 21.27
N
ATOM 2618 CA MET A 334 15.721 17.425 48.243 1.00 20.93
C
ATOM 2619 C MET A 334 14.704 16.834 49.236 1.00 21.48
C
ATOM 2620 O MET A 334 13.549 17.257 49.241 1.00 21.52
O
ATOM 2621 CB MET A 334 15.868 16.499 47.015 1.00 21.53
C
ATOM 2622 CG MET A 334 16.465 17.187 45.776 1.00 22.31
C
ATOM 2623 SD MET A 334 16.291 16.237 44.237 1.00 24.84
S
ATOM 2624 CE MET A 334 17.483 14.903 44.543 1.00 24.19
C
ATOM 2625 H MET A 334 17.817 17.150 48.555 1.00 0.00
H
ATOM 2626 HA MET A 334 15.303 18.375 47.905 1.00 0.00

H
ATOM 2627 HB3 MET A 334 14.890 16.101 46.739 1.00 0.00
H
ATOM 2628 HB2 MET A 334 16.471 15.630 47.281 1.00 0.00
H
ATOM 2629 HG3 MET A 334 17.518 17.423 45.933 1.00 0.00
H
ATOM 2630 HG2 MET A 334 15.961 18.140 45.612 1.00 0.00
H
ATOM 2631 HE1 MET A 334 17.641 14.324 43.633 1.00 0.00
H
ATOM 2632 HE2 MET A 334 18.446 15.306 44.855 1.00 0.00
H
ATOM 2633 HE3 MET A 334 17.121 14.231 45.320 1.00 0.00
H
ATOM 2634 N ALA A 335 15.149 15.895 50.082 1.00 21.64
N
ATOM 2635 CA ALA A 335 14.316 15.245 51.094 1.00 21.82
C
ATOM 2636 C ALA A 335 13.839 16.214 52.190 1.00 22.81
C
ATOM 2637 O ALA A 335 12.678 16.136 52.592 1.00 22.95
O
ATOM 2638 CB ALA A 335 15.078 14.053 51.689 1.00 22.44
C
ATOM 2639 H ALA A 335 16.128 15.639 50.078 1.00 0.00
H
ATOM 2640 HA ALA A 335 13.431 14.858 50.587 1.00 0.00
H
ATOM 2641 HB1 ALA A 335 14.466 13.513 52.411 1.00 0.00
H
ATOM 2642 HB2 ALA A 335 15.357 13.343 50.910 1.00 0.00
H
ATOM 2643 HB3 ALA A 335 15.992 14.366 52.195 1.00 0.00
H
ATOM 2644 N ILE A 336 14.726 17.134 52.610 1.00 21.90

N
ATOM 2645 CA ILE A 336 14.429 18.237 53.529 1.00 22.98
C
ATOM 2646 C ILE A 336 13.490 19.281 52.891 1.00 23.92
C
ATOM 2647 O ILE A 336 12.607 19.779 53.584 1.00 23.55
O
ATOM 2648 CB ILE A 336 15.739 18.926 54.031 1.00 23.77
C
ATOM 2649 CG1 ILE A 336 16.522 17.984 54.975 1.00 24.24
C
ATOM 2650 CG2 ILE A 336 15.560 20.302 54.715 1.00 25.02
C
ATOM 2651 CD1 ILE A 336 18.015 18.326 55.099 1.00 27.51
C
ATOM 2652 H ILE A 336 15.670 17.106 52.248 1.00 0.00
H
ATOM 2653 HA ILE A 336 13.911 17.817 54.394 1.00 0.00
H
ATOM 2654 HB ILE A 336 16.361 19.095 53.150 1.00 0.00
H
ATOM 2655 HG13 ILE A 336 16.443 16.956 54.626 1.00 0.00
H
ATOM 2656 HG12 ILE A 336 16.062 17.989 55.964 1.00 0.00
H
ATOM 2657 HG21 ILE A 336 16.510 20.695 55.075 1.00 0.00
H
ATOM 2658 HG22 ILE A 336 15.161 21.050 54.032 1.00 0.00
H
ATOM 2659 HG23 ILE A 336 14.887 20.234 55.570 1.00 0.00
H
ATOM 2660 HD11 ILE A 336 18.587 17.452 55.408 1.00 0.00
H
ATOM 2661 HD12 ILE A 336 18.431 18.667 54.151 1.00 0.00
H
ATOM 2662 HD13 ILE A 336 18.184 19.110 55.837 1.00 0.00

H

ATOM	2663	N	CYS	A	337	13.666	19.562	51.585	1.00	22.82

N

ATOM	2664	CA	CYS	A	337	12.821	20.466	50.798	1.00	23.92

C

ATOM	2665	C	CYS	A	337	11.348	20.007	50.751	1.00	24.00

C

ATOM	2666	O	CYS	A	337	10.461	20.841	50.934	1.00	25.45

O

ATOM	2667	CB	CYS	A	337	13.411	20.716	49.391	1.00	22.95

C

ATOM	2668	SG	CYS	A	337	12.406	21.846	48.385	1.00	25.85

S

ATOM	2669	H	CYS	A	337	14.428	19.124	51.086	1.00	0.00

H

ATOM	2670	HA	CYS	A	337	12.832	21.425	51.320	1.00	0.00

H

ATOM	2671	HB3	CYS	A	337	13.518	19.780	48.846	1.00	0.00

H

ATOM	2672	HB2	CYS	A	337	14.410	21.145	49.474	1.00	0.00

H

ATOM	2673	HG	CYS	A	337	13.217	21.852	47.323	1.00	0.00

H

ATOM	2674	N	ILE	A	338	11.120	18.694	50.563	1.00	23.65

N

ATOM	2675	CA	ILE	A	338	9.789	18.085	50.611	1.00	23.56

C

ATOM	2676	C	ILE	A	338	9.193	18.133	52.028	1.00	26.02

C

ATOM	2677	O	ILE	A	338	8.080	18.630	52.194	1.00	25.70

O

ATOM	2678	CB	ILE	A	338	9.778	16.606	50.106	1.00	23.28

C

ATOM	2679	CG1	ILE	A	338	10.195	16.523	48.623	1.00	22.98

C

ATOM	2680	CG2	ILE	A	338	8.421	15.882	50.301	1.00	24.00

C
ATOM 2681 CD1 ILE A 338 10.747 15.159 48.185 1.00 22.98
C
ATOM 2682 H ILE A 338 11.898 18.066 50.415 1.00 0.00
H
ATOM 2683 HA ILE A 338 9.139 18.668 49.962 1.00 0.00
H
ATOM 2684 HB ILE A 338 10.529 16.056 50.677 1.00 0.00
H
ATOM 2685 HG13 ILE A 338 10.982 17.248 48.435 1.00 0.00
H
ATOM 2686 HG12 ILE A 338 9.360 16.811 47.984 1.00 0.00
H
ATOM 2687 HG21 ILE A 338 8.437 14.876 49.888 1.00 0.00
H
ATOM 2688 HG22 ILE A 338 8.145 15.770 51.348 1.00 0.00
H
ATOM 2689 HG23 ILE A 338 7.615 16.428 49.809 1.00 0.00
H
ATOM 2690 HD11 ILE A 338 10.541 14.976 47.131 1.00 0.00
H
ATOM 2691 HD12 ILE A 338 11.827 15.116 48.324 1.00 0.00
H
ATOM 2692 HD13 ILE A 338 10.315 14.339 48.752 1.00 0.00
H
ATOM 2693 N VAL A 339 9.948 17.601 53.002 1.00 26.29
N
ATOM 2694 CA VAL A 339 9.461 17.325 54.348 1.00 29.21
C
ATOM 2695 C VAL A 339 10.020 18.385 55.316 1.00 29.45
C
ATOM 2696 O VAL A 339 11.125 18.235 55.838 1.00 30.57
O
ATOM 2697 CB VAL A 339 9.876 15.898 54.809 1.00 30.37
C
ATOM 2698 CG1 VAL A 339 9.204 15.510 56.134 1.00 31.20

C
ATOM 2699 CG2 VAL A 339 9.536 14.828 53.759 1.00 31.68
C
ATOM 2700 H VAL A 339 10.870 17.247 52.787 1.00 0.00
H
ATOM 2701 HA VAL A 339 8.371 17.374 54.372 1.00 0.00
H
ATOM 2702 HB VAL A 339 10.957 15.854 54.956 1.00 0.00
H
ATOM 2703 HG11 VAL A 339 9.415 14.476 56.386 1.00 0.00
H
ATOM 2704 HG12 VAL A 339 9.543 16.131 56.961 1.00 0.00
H
ATOM 2705 HG13 VAL A 339 8.120 15.589 56.063 1.00 0.00
H
ATOM 2706 HG21 VAL A 339 9.784 13.829 54.119 1.00 0.00
H
ATOM 2707 HG22 VAL A 339 8.473 14.850 53.518 1.00 0.00
H
ATOM 2708 HG23 VAL A 339 10.092 14.975 52.835 1.00 0.00
H
ATOM 2709 N SER A 340 9.234 19.457 55.499 1.00 30.04
N
ATOM 2710 CA SER A 340 9.519 20.583 56.389 1.00 30.94
C
ATOM 2711 C SER A 340 8.199 20.990 57.079 1.00 31.69
C
ATOM 2712 O SER A 340 7.173 21.041 56.399 1.00 31.64
O
ATOM 2713 CB SER A 340 10.075 21.755 55.555 1.00 32.39
C
ATOM 2714 OG SER A 340 11.463 21.620 55.343 1.00 35.78
O
ATOM 2715 H SER A 340 8.347 19.503 55.018 1.00 0.00
H
ATOM 2716 HA SER A 340 10.259 20.276 57.127 1.00 0.00

H
ATOM 2717 HB3 SER A 340 9.957 22.687 56.104 1.00 0.00
H
ATOM 2718 HB2 SER A 340 9.552 21.869 54.605 1.00 0.00
H
ATOM 2719 HG SER A 340 11.620 20.872 54.756 1.00 0.00
H
ATOM 2720 N PRO A 341 8.211 21.222 58.413 1.00 32.38
N
ATOM 2721 CA PRO A 341 6.976 21.430 59.196 1.00 33.82
C
ATOM 2722 C PRO A 341 6.274 22.788 58.996 1.00 35.40
C
ATOM 2723 O PRO A 341 5.128 22.922 59.424 1.00 36.23
O
ATOM 2724 CB PRO A 341 7.447 21.231 60.643 1.00 33.68
C
ATOM 2725 CG PRO A 341 8.888 21.713 60.637 1.00 33.78
C
ATOM 2726 CD PRO A 341 9.390 21.238 59.280 1.00 32.78
C
ATOM 2727 HA PRO A 341 6.244 20.656 58.956 1.00 0.00
H
ATOM 2728 HB3 PRO A 341 7.425 20.167 60.873 1.00 0.00
H
ATOM 2729 HB2 PRO A 341 6.837 21.742 61.389 1.00 0.00
H
ATOM 2730 HG3 PRO A 341 9.482 21.340 61.471 1.00 0.00
H
ATOM 2731 HG2 PRO A 341 8.908 22.803 60.674 1.00 0.00
H
ATOM 2732 HD2 PRO A 341 10.169 21.896 58.898 1.00 0.00
H
ATOM 2733 HD3 PRO A 341 9.787 20.225 59.363 1.00 0.00
H
ATOM 2734 N ASP A 342 6.947 23.753 58.342 1.00 36.36

N
ATOM 2735 CA ASP A 342 6.412 25.080 58.004 1.00 37.82
C
ATOM 2736 C ASP A 342 5.386 25.054 56.848 1.00 38.30
C
ATOM 2737 O ASP A 342 4.650 26.029 56.694 1.00 39.93
O
ATOM 2738 CB ASP A 342 7.520 26.132 57.718 1.00 39.62
C
ATOM 2739 CG ASP A 342 8.598 25.749 56.693 1.00 41.16
C
ATOM 2740 OD1 ASP A 342 9.522 26.574 56.521 1.00 43.27
O
ATOM 2741 OD2 ASP A 342 8.477 24.686 56.046 1.00 42.71
O1-
ATOM 2742 H ASP A 342 7.880 23.566 58.003 1.00 0.00
H
ATOM 2743 HA ASP A 342 5.868 25.434 58.882 1.00 0.00
H
ATOM 2744 HB3 ASP A 342 8.028 26.338 58.660 1.00 0.00
H
ATOM 2745 HB2 ASP A 342 7.060 27.065 57.391 1.00 0.00
H
ATOM 2746 N ARG A 343 5.358 23.958 56.067 1.00 37.31
N
ATOM 2747 CA ARG A 343 4.438 23.705 54.955 1.00 37.10
C
ATOM 2748 C ARG A 343 2.975 23.660 55.464 1.00 37.98
C
ATOM 2749 O ARG A 343 2.660 22.771 56.257 1.00 38.15
O
ATOM 2750 CB ARG A 343 4.829 22.361 54.301 1.00 35.71
C
ATOM 2751 CG ARG A 343 6.230 22.323 53.655 1.00 32.83
C
ATOM 2752 CD ARG A 343 6.396 23.263 52.458 1.00 31.70

C
ATOM 2753 NE ARG A 343 7.613 22.942 51.702 1.00 29.77
N
ATOM 2754 CZ ARG A 343 8.070 23.621 50.640 1.00 28.95
C
ATOM 2755 NH1 ARG A 343 7.377 24.637 50.109 1.00 27.66
N
ATOM 2756 NH2 ARG A 343 9.250 23.281 50.115 1.00 28.12
N1+
ATOM 2757 H ARG A 343 6.008 23.211 56.272 1.00 0.00
H
ATOM 2758 HA ARG A 343 4.602 24.497 54.227 1.00 0.00
H
ATOM 2759 HB3 ARG A 343 4.090 22.083 53.548 1.00 0.00
H
ATOM 2760 HB2 ARG A 343 4.783 21.583 55.063 1.00 0.00
H
ATOM 2761 HG3 ARG A 343 6.515 21.311 53.373 1.00 0.00
H
ATOM 2762 HG2 ARG A 343 6.949 22.631 54.413 1.00 0.00
H
ATOM 2763 HD3 ARG A 343 6.589 24.264 52.845 1.00 0.00
H
ATOM 2764 HD2 ARG A 343 5.500 23.342 51.848 1.00 0.00
H
ATOM 2765 HE ARG A 343 8.137 22.138 52.022 1.00 0.00
H
ATOM 2766 HH12 ARG A 343 7.736 25.140 49.299 1.00 0.00
H
ATOM 2767 HH11 ARG A 343 6.472 24.884 50.480 1.00 0.00
H
ATOM 2768 HH22 ARG A 343 9.672 23.854 49.386 1.00 0.00
H
ATOM 2769 HH21 ARG A 343 9.762 22.477 50.463 1.00 0.00
H
ATOM 2770 N PRO A 344 2.130 24.641 55.065 1.00 39.05

N
ATOM 2771 CA PRO A 344 0.798 24.831 55.671 1.00 39.38
C
ATOM 2772 C PRO A 344 -0.241 23.781 55.234 1.00 38.87
C
ATOM 2773 O PRO A 344 -0.186 23.297 54.104 1.00 39.29
O
ATOM 2774 CB PRO A 344 0.406 26.244 55.209 1.00 39.90
C
ATOM 2775 CG PRO A 344 1.093 26.413 53.865 1.00 40.56
C
ATOM 2776 CD PRO A 344 2.411 25.675 54.064 1.00 39.66
C
ATOM 2777 HA PRO A 344 0.879 24.814 56.760 1.00 0.00
H
ATOM 2778 HB3 PRO A 344 0.802 26.975 55.916 1.00 0.00
H
ATOM 2779 HB2 PRO A 344 -0.671 26.405 55.147 1.00 0.00
H
ATOM 2780 HG3 PRO A 344 1.222 27.454 53.568 1.00 0.00
H
ATOM 2781 HG2 PRO A 344 0.508 25.912 53.092 1.00 0.00
H
ATOM 2782 HD2 PRO A 344 2.771 25.260 53.122 1.00 0.00
H
ATOM 2783 HD3 PRO A 344 3.166 26.358 54.455 1.00 0.00
H
ATOM 2784 N GLY A 345 -1.182 23.485 56.145 1.00 38.50
N
ATOM 2785 CA GLY A 345 -2.325 22.601 55.902 1.00 37.96
C
ATOM 2786 C GLY A 345 -2.091 21.169 56.410 1.00 37.94
C
ATOM 2787 O GLY A 345 -2.999 20.346 56.298 1.00 36.75
O
ATOM 2788 H GLY A 345 -1.145 23.930 57.051 1.00 0.00

H
ATOM 2789 HA3 GLY A 345 -2.582 22.567 54.842 1.00 0.00
H
ATOM 2790 HA2 GLY A 345 -3.192 23.013 56.419 1.00 0.00
H
ATOM 2791 N VAL A 346 -0.903 20.864 56.960 1.00 37.90
N
ATOM 2792 CA VAL A 346 -0.553 19.561 57.529 1.00 38.39
C
ATOM 2793 C VAL A 346 -1.201 19.356 58.916 1.00 39.30
C
ATOM 2794 O VAL A 346 -1.316 20.309 59.689 1.00 39.35
O
ATOM 2795 CB VAL A 346 0.988 19.407 57.658 1.00 38.81
C
ATOM 2796 CG1 VAL A 346 1.664 19.442 56.275 1.00 37.92
C
ATOM 2797 CG2 VAL A 346 1.669 20.396 58.629 1.00 38.02
C
ATOM 2798 H VAL A 346 -0.189 21.576 57.021 1.00 0.00
H
ATOM 2799 HA VAL A 346 -0.921 18.783 56.856 1.00 0.00
H
ATOM 2800 HB VAL A 346 1.177 18.409 58.057 1.00 0.00
H
ATOM 2801 HG11 VAL A 346 2.744 19.336 56.354 1.00 0.00
H
ATOM 2802 HG12 VAL A 346 1.299 18.638 55.636 1.00 0.00
H
ATOM 2803 HG13 VAL A 346 1.478 20.386 55.765 1.00 0.00
H
ATOM 2804 HG21 VAL A 346 2.754 20.300 58.598 1.00 0.00
H
ATOM 2805 HG22 VAL A 346 1.429 21.430 58.382 1.00 0.00
H
ATOM 2806 HG23 VAL A 346 1.367 20.219 59.660 1.00 0.00

H
ATOM 2807 N GLN A 347 -1.617 18.110 59.188 1.00 39.79
N
ATOM 2808 CA GLN A 347 -2.302 17.704 60.416 1.00 40.19
C
ATOM 2809 C GLN A 347 -1.334 17.388 61.568 1.00 38.86
C
ATOM 2810 O GLN A 347 -1.678 17.668 62.716 1.00 39.58
O
ATOM 2811 CB GLN A 347 -3.199 16.478 60.122 1.00 43.00
C
ATOM 2812 CG GLN A 347 -4.333 16.720 59.101 1.00 47.50
C
ATOM 2813 CD GLN A 347 -5.363 17.772 59.529 1.00 50.27
C
ATOM 2814 OE1 GLN A 347 -5.551 18.036 60.715 1.00 52.52
O
ATOM 2815 NE2 GLN A 347 -6.064 18.361 58.559 1.00 51.48
N
ATOM 2816 H GLN A 347 -1.485 17.377 58.498 1.00 0.00
H
ATOM 2817 HA GLN A 347 -2.930 18.530 60.752 1.00 0.00
H
ATOM 2818 HB3 GLN A 347 -3.638 16.115 61.054 1.00 0.00
H
ATOM 2819 HB2 GLN A 347 -2.578 15.658 59.759 1.00 0.00
H
ATOM 2820 HG3 GLN A 347 -4.864 15.784 58.925 1.00 0.00
H
ATOM 2821 HG2 GLN A 347 -3.906 17.008 58.139 1.00 0.00
H
ATOM 2822 HE22 GLN A 347 -6.760 19.055 58.786 1.00 0.00
H
ATOM 2823 HE21 GLN A 347 -5.923 18.099 57.587 1.00 0.00
H
ATOM 2824 N ASP A 348 -0.160 16.818 61.248 1.00 36.34

N

ATOM	2825	CA	ASP	A	348	0.850	16.400	62.217	1.00	35.34	C
ATOM	2826	C	ASP	A	348	2.206	16.964	61.765	1.00	33.83	C
ATOM	2827	O	ASP	A	348	2.998	16.260	61.138	1.00	33.85	O
ATOM	2828	CB	ASP	A	348	0.837	14.860	62.408	1.00	34.84	C
ATOM	2829	CG	ASP	A	348	1.731	14.320	63.536	1.00	35.23	C
ATOM	2830	OD1	ASP	A	348	1.672	13.089	63.742	1.00	34.74	O
ATOM	2831	OD2	ASP	A	348	2.458	15.117	64.170	1.00	34.97	O1-
ATOM	2832	H	ASP	A	348	0.035	16.583	60.281	1.00	0.00	H
ATOM	2833	HA	ASP	A	348	0.635	16.849	63.189	1.00	0.00	H
ATOM	2834	HB3	ASP	A	348	1.129	14.376	61.475	1.00	0.00	H
ATOM	2835	HB2	ASP	A	348	-0.187	14.556	62.630	1.00	0.00	H
ATOM	2836	N	ALA	A	349	2.447	18.234	62.128	1.00	32.41	N
ATOM	2837	CA	ALA	A	349	3.715	18.933	61.921	1.00	32.51	C
ATOM	2838	C	ALA	A	349	4.874	18.404	62.790	1.00	32.68	C
ATOM	2839	O	ALA	A	349	6.023	18.627	62.419	1.00	32.47	O
ATOM	2840	CB	ALA	A	349	3.506	20.435	62.169	1.00	32.86	C
ATOM	2841	H	ALA	A	349	1.734	18.746	62.627	1.00	0.00	H
ATOM	2842	HA	ALA	A	349	4.001	18.806	60.876	1.00	0.00	

H
ATOM 2843 HB1 ALA A 349 4.421 20.998 61.978 1.00 0.00
H
ATOM 2844 HB2 ALA A 349 2.737 20.841 61.513 1.00 0.00
H
ATOM 2845 HB3 ALA A 349 3.202 20.632 63.198 1.00 0.00
H
ATOM 2846 N ALA A 350 4.572 17.718 63.908 1.00 31.95
N
ATOM 2847 CA ALA A 350 5.567 17.165 64.833 1.00 31.15
C
ATOM 2848 C ALA A 350 6.305 15.937 64.273 1.00 30.76
C
ATOM 2849 O ALA A 350 7.509 15.813 64.500 1.00 30.22
O
ATOM 2850 CB ALA A 350 4.891 16.836 66.172 1.00 32.37
C
ATOM 2851 H ALA A 350 3.605 17.538 64.137 1.00 0.00
H
ATOM 2852 HA ALA A 350 6.313 17.939 65.023 1.00 0.00
H
ATOM 2853 HB1 ALA A 350 5.614 16.457 66.895 1.00 0.00
H
ATOM 2854 HB2 ALA A 350 4.431 17.724 66.606 1.00 0.00
H
ATOM 2855 HB3 ALA A 350 4.112 16.082 66.055 1.00 0.00
H
ATOM 2856 N LEU A 351 5.590 15.076 63.527 1.00 30.64
N
ATOM 2857 CA LEU A 351 6.164 13.926 62.817 1.00 30.92
C
ATOM 2858 C LEU A 351 7.001 14.386 61.608 1.00 30.72
C
ATOM 2859 O LEU A 351 8.072 13.830 61.371 1.00 30.35
O
ATOM 2860 CB LEU A 351 5.019 12.960 62.427 1.00 32.92

C
ATOM 2861 CG LEU A 351 5.405 11.474 62.203 1.00 34.73
C
ATOM 2862 CD1 LEU A 351 4.141 10.589 62.141 1.00 35.25
C
ATOM 2863 CD2 LEU A 351 6.324 11.218 60.990 1.00 36.86
C
ATOM 2864 H LEU A 351 4.594 15.222 63.417 1.00 0.00
H
ATOM 2865 HA LEU A 351 6.826 13.408 63.512 1.00 0.00
H
ATOM 2866 HB3 LEU A 351 4.461 13.350 61.574 1.00 0.00
H
ATOM 2867 HB2 LEU A 351 4.314 12.966 63.257 1.00 0.00
H
ATOM 2868 HG LEU A 351 5.955 11.159 63.091 1.00 0.00
H
ATOM 2869 HD11 LEU A 351 4.237 9.727 62.801 1.00 0.00
H
ATOM 2870 HD12 LEU A 351 3.242 11.127 62.445 1.00 0.00
H
ATOM 2871 HD13 LEU A 351 3.945 10.213 61.138 1.00 0.00
H
ATOM 2872 HD21 LEU A 351 6.012 10.361 60.396 1.00 0.00
H
ATOM 2873 HD22 LEU A 351 6.350 12.065 60.311 1.00 0.00
H
ATOM 2874 HD23 LEU A 351 7.347 11.024 61.312 1.00 0.00
H
ATOM 2875 N ILE A 352 6.518 15.421 60.899 1.00 30.54
N
ATOM 2876 CA ILE A 352 7.197 16.067 59.771 1.00 29.35
C
ATOM 2877 C ILE A 352 8.511 16.756 60.199 1.00 29.73
C
ATOM 2878 O ILE A 352 9.507 16.646 59.484 1.00 30.37

O
ATOM 2879 CB ILE A 352 6.256 17.092 59.073 1.00 29.44
C
ATOM 2880 CG1 ILE A 352 5.026 16.372 58.479 1.00 29.58
C
ATOM 2881 CG2 ILE A 352 6.926 17.949 57.981 1.00 28.41
C
ATOM 2882 CD1 ILE A 352 3.899 17.303 58.028 1.00 29.04
C
ATOM 2883 H ILE A 352 5.630 15.822 61.165 1.00 0.00
H
ATOM 2884 HA ILE A 352 7.453 15.293 59.048 1.00 0.00
H
ATOM 2885 HB ILE A 352 5.897 17.778 59.840 1.00 0.00
H
ATOM 2886 HG13 ILE A 352 4.603 15.681 59.204 1.00 0.00
H
ATOM 2887 HG12 ILE A 352 5.344 15.760 57.637 1.00 0.00
H
ATOM 2888 HG21 ILE A 352 6.223 18.667 57.559 1.00 0.00
H
ATOM 2889 HG22 ILE A 352 7.766 18.521 58.366 1.00 0.00
H
ATOM 2890 HG23 ILE A 352 7.292 17.337 57.161 1.00 0.00
H
ATOM 2891 HD11 ILE A 352 2.928 16.838 58.197 1.00 0.00
H
ATOM 2892 HD12 ILE A 352 3.911 18.247 58.571 1.00 0.00
H
ATOM 2893 HD13 ILE A 352 3.982 17.525 56.966 1.00 0.00
H
ATOM 2894 N GLU A 353 8.499 17.396 61.380 1.00 30.02
N
ATOM 2895 CA GLU A 353 9.667 17.973 62.043 1.00 30.56
C
ATOM 2896 C GLU A 353 10.709 16.907 62.428 1.00 29.99

C
ATOM 2897 O GLU A 353 11.892 17.127 62.190 1.00 29.97
O
ATOM 2898 CB GLU A 353 9.193 18.807 63.251 1.00 32.59
C
ATOM 2899 CG GLU A 353 10.307 19.494 64.065 1.00 35.81
C
ATOM 2900 CD GLU A 353 9.727 20.363 65.181 1.00 38.69
C
ATOM 2901 OE1 GLU A 353 9.978 21.587 65.143 1.00 40.67
O
ATOM 2902 OE2 GLU A 353 9.030 19.791 66.049 1.00 40.80
O1-
ATOM 2903 H GLU A 353 7.630 17.464 61.894 1.00 0.00
H
ATOM 2904 HA GLU A 353 10.142 18.655 61.334 1.00 0.00
H
ATOM 2905 HB3 GLU A 353 8.610 18.170 63.916 1.00 0.00
H
ATOM 2906 HB2 GLU A 353 8.501 19.573 62.902 1.00 0.00
H
ATOM 2907 HG3 GLU A 353 10.927 20.107 63.410 1.00 0.00
H
ATOM 2908 HG2 GLU A 353 10.969 18.757 64.523 1.00 0.00
H
ATOM 2909 N ALA A 354 10.253 15.758 62.959 1.00 29.90
N
ATOM 2910 CA ALA A 354 11.104 14.625 63.335 1.00 29.96
C
ATOM 2911 C ALA A 354 11.828 13.962 62.147 1.00 29.90
C
ATOM 2912 O ALA A 354 12.968 13.530 62.320 1.00 31.40
O
ATOM 2913 CB ALA A 354 10.272 13.597 64.115 1.00 30.18
C
ATOM 2914 H ALA A 354 9.263 15.653 63.137 1.00 0.00

H
ATOM 2915 HA ALA A 354 11.872 15.011 64.008 1.00 0.00
H
ATOM 2916 HB1 ALA A 354 10.895 12.775 64.470 1.00 0.00
H
ATOM 2917 HB2 ALA A 354 9.807 14.053 64.990 1.00 0.00
H
ATOM 2918 HB3 ALA A 354 9.478 13.169 63.504 1.00 0.00
H
ATOM 2919 N ILE A 355 11.183 13.926 60.964 1.00 29.82
N
ATOM 2920 CA ILE A 355 11.807 13.485 59.712 1.00 28.57
C
ATOM 2921 C ILE A 355 12.833 14.521 59.207 1.00 28.03
C
ATOM 2922 O ILE A 355 13.927 14.117 58.816 1.00 27.72
O
ATOM 2923 CB ILE A 355 10.775 13.208 58.574 1.00 29.38
C
ATOM 2924 CG1 ILE A 355 9.752 12.107 58.935 1.00 30.07
C
ATOM 2925 CG2 ILE A 355 11.430 12.860 57.212 1.00 30.16
C
ATOM 2926 CD1 ILE A 355 8.427 12.235 58.169 1.00 30.83
C
ATOM 2927 H ILE A 355 10.239 14.284 60.899 1.00 0.00
H
ATOM 2928 HA ILE A 355 12.345 12.557 59.912 1.00 0.00
H
ATOM 2929 HB ILE A 355 10.207 14.129 58.449 1.00 0.00
H
ATOM 2930 HG13 ILE A 355 9.526 12.120 60.000 1.00 0.00
H
ATOM 2931 HG12 ILE A 355 10.176 11.124 58.736 1.00 0.00
H
ATOM 2932 HG21 ILE A 355 10.700 12.534 56.474 1.00 0.00

H
ATOM 2933 HG22 ILE A 355 11.940 13.716 56.773 1.00 0.00
H
ATOM 2934 HG23 ILE A 355 12.157 12.054 57.319 1.00 0.00
H
ATOM 2935 HD11 ILE A 355 7.726 11.460 58.473 1.00 0.00
H
ATOM 2936 HD12 ILE A 355 7.949 13.195 58.356 1.00 0.00
H
ATOM 2937 HD13 ILE A 355 8.567 12.137 57.093 1.00 0.00
H
ATOM 2938 N GLN A 356 12.483 15.823 59.256 1.00 27.45
N
ATOM 2939 CA GLN A 356 13.348 16.939 58.855 1.00 28.48
C
ATOM 2940 C GLN A 356 14.659 16.979 59.663 1.00 29.24
C
ATOM 2941 O GLN A 356 15.722 17.065 59.051 1.00 27.50
O
ATOM 2942 CB GLN A 356 12.586 18.278 58.971 1.00 28.93
C
ATOM 2943 CG GLN A 356 13.340 19.480 58.350 1.00 29.68
C
ATOM 2944 CD GLN A 356 12.896 20.837 58.900 1.00 31.36
C
ATOM 2945 OE1 GLN A 356 12.616 20.982 60.087 1.00 31.51
O
ATOM 2946 NE2 GLN A 356 12.867 21.859 58.044 1.00 30.04
N
ATOM 2947 H GLN A 356 11.563 16.077 59.591 1.00 0.00
H
ATOM 2948 HA GLN A 356 13.603 16.785 57.804 1.00 0.00
H
ATOM 2949 HB3 GLN A 356 12.371 18.464 60.023 1.00 0.00
H
ATOM 2950 HB2 GLN A 356 11.611 18.194 58.490 1.00 0.00

H
ATOM 2951 HG3 GLN A 356 13.230 19.456 57.265 1.00 0.00
H
ATOM 2952 HG2 GLN A 356 14.410 19.423 58.546 1.00 0.00
H
ATOM 2953 HE22 GLN A 356 12.608 22.779 58.369 1.00 0.00
H
ATOM 2954 HE21 GLN A 356 13.069 21.719 57.064 1.00 0.00
H
ATOM 2955 N ASP A 357 14.555 16.880 61.002 1.00 29.52
N
ATOM 2956 CA ASP A 357 15.679 16.826 61.946 1.00 30.44
C
ATOM 2957 C ASP A 357 16.609 15.627 61.716 1.00 29.70
C
ATOM 2958 O ASP A 357 17.823 15.801 61.796 1.00 30.37
O
ATOM 2959 CB ASP A 357 15.254 16.870 63.435 1.00 32.83
C
ATOM 2960 CG ASP A 357 14.392 18.069 63.845 1.00 34.76
C
ATOM 2961 OD1 ASP A 357 14.418 19.090 63.122 1.00 36.63
O
ATOM 2962 OD2 ASP A 357 13.769 17.961 64.922 1.00 36.89
O1-
ATOM 2963 H ASP A 357 13.633 16.856 61.421 1.00 0.00
H
ATOM 2964 HA ASP A 357 16.273 17.722 61.760 1.00 0.00
H
ATOM 2965 HB3 ASP A 357 16.140 16.864 64.072 1.00 0.00
H
ATOM 2966 HB2 ASP A 357 14.682 15.966 63.652 1.00 0.00
H
ATOM 2967 N ARG A 358 16.039 14.449 61.405 1.00 30.08
N
ATOM 2968 CA ARG A 358 16.790 13.231 61.094 1.00 30.11

C

ATOM 2969 C ARG A 358 17.665 13.375 59.832 1.00 29.92
C

ATOM 2970 O ARG A 358 18.836 13.000 59.875 1.00 29.08
O

ATOM 2971 CB ARG A 358 15.820 12.030 61.037 1.00 31.17
C

ATOM 2972 CG ARG A 358 16.481 10.690 60.659 1.00 33.90
C

ATOM 2973 CD ARG A 358 15.621 9.449 60.939 1.00 36.03
C

ATOM 2974 NE ARG A 358 14.307 9.492 60.282 1.00 38.00
N

ATOM 2975 CZ ARG A 358 13.147 9.845 60.858 1.00 39.39
C

ATOM 2976 NH1 ARG A 358 13.081 10.211 62.146 1.00 39.22
N

ATOM 2977 NH2 ARG A 358 12.029 9.821 60.127 1.00 39.21
N1+

ATOM 2978 H ARG A 358 15.031 14.382 61.357 1.00 0.00
H

ATOM 2979 HA ARG A 358 17.467 13.059 61.934 1.00 0.00
H

ATOM 2980 HB3 ARG A 358 15.016 12.234 60.329 1.00 0.00
H

ATOM 2981 HB2 ARG A 358 15.345 11.931 62.013 1.00 0.00
H

ATOM 2982 HG3 ARG A 358 17.340 10.605 61.326 1.00 0.00
H

ATOM 2983 HG2 ARG A 358 16.885 10.678 59.647 1.00 0.00
H

ATOM 2984 HD3 ARG A 358 15.613 9.128 61.981 1.00 0.00
H

ATOM 2985 HD2 ARG A 358 16.101 8.635 60.398 1.00 0.00
H

ATOM 2986 HE ARG A 358 14.301 9.270 59.297 1.00 0.00

H
ATOM 2987 HH12 ARG A 358 12.202 10.485 62.561 1.00 0.00
H
ATOM 2988 HH11 ARG A 358 13.920 10.236 62.707 1.00 0.00
H
ATOM 2989 HH22 ARG A 358 11.140 10.066 60.540 1.00 0.00
H
ATOM 2990 HH21 ARG A 358 12.064 9.562 59.148 1.00 0.00
H
ATOM 2991 N LEU A 359 17.097 13.959 58.763 1.00 28.41
N
ATOM 2992 CA LEU A 359 17.795 14.261 57.510 1.00 27.76
C
ATOM 2993 C LEU A 359 18.828 15.395 57.665 1.00 27.89
C
ATOM 2994 O LEU A 359 19.878 15.334 57.029 1.00 27.13
O
ATOM 2995 CB LEU A 359 16.766 14.646 56.425 1.00 27.85
C
ATOM 2996 CG LEU A 359 15.730 13.558 56.066 1.00 27.95
C
ATOM 2997 CD1 LEU A 359 14.555 14.191 55.302 1.00 28.15
C
ATOM 2998 CD2 LEU A 359 16.344 12.372 55.298 1.00 28.81
C
ATOM 2999 H LEU A 359 16.127 14.243 58.810 1.00 0.00
H
ATOM 3000 HA LEU A 359 18.329 13.365 57.189 1.00 0.00
H
ATOM 3001 HB3 LEU A 359 17.286 14.947 55.515 1.00 0.00
H
ATOM 3002 HB2 LEU A 359 16.230 15.533 56.767 1.00 0.00
H
ATOM 3003 HG LEU A 359 15.316 13.145 56.984 1.00 0.00
H
ATOM 3004 HD11 LEU A 359 13.963 13.450 54.767 1.00 0.00

H
ATOM 3005 HD12 LEU A 359 13.885 14.710 55.987 1.00 0.00
H
ATOM 3006 HD13 LEU A 359 14.908 14.926 54.580 1.00 0.00
H
ATOM 3007 HD21 LEU A 359 16.227 11.444 55.859 1.00 0.00
H
ATOM 3008 HD22 LEU A 359 15.872 12.223 54.327 1.00 0.00
H
ATOM 3009 HD23 LEU A 359 17.410 12.507 55.119 1.00 0.00
H
ATOM 3010 N SER A 360 18.514 16.400 58.502 1.00 28.62
N
ATOM 3011 CA SER A 360 19.344 17.579 58.753 1.00 30.04
C
ATOM 3012 C SER A 360 20.606 17.266 59.574 1.00 30.70
C
ATOM 3013 O SER A 360 21.666 17.793 59.245 1.00 31.03
O
ATOM 3014 CB SER A 360 18.507 18.672 59.442 1.00 32.12
C
ATOM 3015 OG SER A 360 17.611 19.266 58.527 1.00 36.13
O
ATOM 3016 H SER A 360 17.617 16.386 58.971 1.00 0.00
H
ATOM 3017 HA SER A 360 19.684 17.967 57.790 1.00 0.00
H
ATOM 3018 HB3 SER A 360 19.148 19.461 59.828 1.00 0.00
H
ATOM 3019 HB2 SER A 360 17.957 18.273 60.293 1.00 0.00
H
ATOM 3020 HG SER A 360 16.878 18.659 58.391 1.00 0.00
H
ATOM 3021 N ASN A 361 20.486 16.400 60.597 1.00 31.34
N
ATOM 3022 CA ASN A 361 21.614 15.909 61.398 1.00 31.58

C
ATOM 3023 C ASN A 361 22.500 14.930 60.612 1.00 30.77
C
ATOM 3024 O ASN A 361 23.713 14.963 60.811 1.00 31.06
O
ATOM 3025 CB ASN A 361 21.093 15.275 62.707 1.00 34.28
C
ATOM 3026 CG ASN A 361 20.713 16.318 63.764 1.00 37.26
C
ATOM 3027 OD1 ASN A 361 21.555 17.108 64.186 1.00 39.61
O
ATOM 3028 ND2 ASN A 361 19.458 16.314 64.215 1.00 39.64
N
ATOM 3029 H ASN A 361 19.574 16.027 60.831 1.00 0.00
H
ATOM 3030 HA ASN A 361 22.198 16.799 61.644 1.00 0.00
H
ATOM 3031 HB3 ASN A 361 21.882 14.672 63.161 1.00 0.00
H
ATOM 3032 HB2 ASN A 361 20.269 14.587 62.510 1.00 0.00
H
ATOM 3033 HD22 ASN A 361 19.172 16.976 64.921 1.00 0.00
H
ATOM 3034 HD21 ASN A 361 18.777 15.678 63.824 1.00 0.00
H
ATOM 3035 N THR A 362 21.909 14.127 59.709 1.00 29.26
N
ATOM 3036 CA THR A 362 22.643 13.262 58.777 1.00 28.14
C
ATOM 3037 C THR A 362 23.472 14.077 57.760 1.00 27.56
C
ATOM 3038 O THR A 362 24.611 13.703 57.483 1.00 27.15
O
ATOM 3039 CB THR A 362 21.683 12.313 58.006 1.00 28.47
C
ATOM 3040 OG1 THR A 362 21.037 11.450 58.917 1.00 28.00

O
ATOM 3041 CG2 THR A 362 22.330 11.423 56.932 1.00 27.60
C
ATOM 3042 H THR A 362 20.904 14.148 59.604 1.00 0.00
H
ATOM 3043 HA THR A 362 23.338 12.653 59.357 1.00 0.00
H
ATOM 3044 HB THR A 362 20.896 12.902 57.536 1.00 0.00
H
ATOM 3045 HG1 THR A 362 20.429 11.969 59.454 1.00 0.00
H
ATOM 3046 HG21 THR A 362 21.621 10.686 56.557 1.00 0.00
H
ATOM 3047 HG22 THR A 362 22.671 12.001 56.075 1.00 0.00
H
ATOM 3048 HG23 THR A 362 23.191 10.885 57.324 1.00 0.00
H
ATOM 3049 N LEU A 363 22.905 15.197 57.275 1.00 26.97
N
ATOM 3050 CA LEU A 363 23.542 16.150 56.366 1.00 27.40
C
ATOM 3051 C LEU A 363 24.670 16.952 57.036 1.00 28.23
C
ATOM 3052 O LEU A 363 25.738 17.088 56.442 1.00 27.96
O
ATOM 3053 CB LEU A 363 22.446 17.064 55.766 1.00 26.59
C
ATOM 3054 CG LEU A 363 22.897 18.180 54.793 1.00 26.54
C
ATOM 3055 CD1 LEU A 363 23.619 17.635 53.547 1.00 26.43
C
ATOM 3056 CD2 LEU A 363 21.717 19.098 54.416 1.00 26.28
C
ATOM 3057 H LEU A 363 21.957 15.421 57.546 1.00 0.00
H
ATOM 3058 HA LEU A 363 23.997 15.565 55.567 1.00 0.00

H
ATOM 3059 HB3 LEU A 363 21.905 17.530 56.589 1.00 0.00
H
ATOM 3060 HB2 LEU A 363 21.714 16.437 55.257 1.00 0.00
H
ATOM 3061 HG LEU A 363 23.609 18.808 55.327 1.00 0.00
H
ATOM 3062 HD11 LEU A 363 24.335 18.358 53.162 1.00 0.00
H
ATOM 3063 HD12 LEU A 363 24.184 16.727 53.742 1.00 0.00
H
ATOM 3064 HD13 LEU A 363 22.915 17.411 52.747 1.00 0.00
H
ATOM 3065 HD21 LEU A 363 21.994 20.148 54.524 1.00 0.00
H
ATOM 3066 HD22 LEU A 363 21.387 18.951 53.387 1.00 0.00
H
ATOM 3067 HD23 LEU A 363 20.849 18.930 55.053 1.00 0.00
H
ATOM 3068 N GLN A 364 24.416 17.446 58.260 1.00 29.48
N
ATOM 3069 CA GLN A 364 25.364 18.208 59.074 1.00 31.77
C
ATOM 3070 C GLN A 364 26.592 17.369 59.472 1.00 30.91
C
ATOM 3071 O GLN A 364 27.710 17.881 59.417 1.00 30.91
O
ATOM 3072 CB GLN A 364 24.620 18.756 60.310 1.00 33.97
C
ATOM 3073 CG GLN A 364 25.434 19.741 61.177 1.00 39.97
C
ATOM 3074 CD GLN A 364 24.737 20.157 62.479 1.00 41.73
C
ATOM 3075 OE1 GLN A 364 25.408 20.581 63.416 1.00 45.56
O
ATOM 3076 NE2 GLN A 364 23.408 20.046 62.561 1.00 43.48

N
ATOM 3077 H GLN A 364 23.498 17.310 58.663 1.00 0.00
H
ATOM 3078 HA GLN A 364 25.707 19.054 58.476 1.00 0.00
H
ATOM 3079 HB3 GLN A 364 24.282 17.918 60.922 1.00 0.00
H
ATOM 3080 HB2 GLN A 364 23.719 19.266 59.972 1.00 0.00
H
ATOM 3081 HG3 GLN A 364 25.658 20.640 60.602 1.00 0.00
H
ATOM 3082 HG2 GLN A 364 26.395 19.307 61.450 1.00 0.00
H
ATOM 3083 HE22 GLN A 364 22.932 20.304 63.413 1.00 0.00
H
ATOM 3084 HE21 GLN A 364 22.870 19.704 61.778 1.00 0.00
H
ATOM 3085 N THR A 365 26.365 16.094 59.833 1.00 30.38
N
ATOM 3086 CA THR A 365 27.422 15.132 60.147 1.00 30.54
C
ATOM 3087 C THR A 365 28.230 14.719 58.900 1.00 29.67
C
ATOM 3088 O THR A 365 29.444 14.586 59.023 1.00 30.84
O
ATOM 3089 CB THR A 365 26.876 13.856 60.843 1.00 30.66
C
ATOM 3090 OG1 THR A 365 26.181 14.228 62.017 1.00 32.31
O
ATOM 3091 CG2 THR A 365 27.945 12.833 61.271 1.00 31.16
C
ATOM 3092 H THR A 365 25.417 15.740 59.853 1.00 0.00
H
ATOM 3093 HA THR A 365 28.118 15.614 60.837 1.00 0.00
H
ATOM 3094 HB THR A 365 26.153 13.367 60.189 1.00 0.00

H
ATOM 3095 HG1 THR A 365 25.326 14.592 61.761 1.00 0.00
H
ATOM 3096 HG21 THR A 365 27.500 11.999 61.812 1.00 0.00
H
ATOM 3097 HG22 THR A 365 28.486 12.411 60.426 1.00 0.00
H
ATOM 3098 HG23 THR A 365 28.675 13.293 61.936 1.00 0.00
H
ATOM 3099 N TYR A 366 27.575 14.586 57.728 1.00 29.11
N
ATOM 3100 CA TYR A 366 28.234 14.302 56.446 1.00 28.71
C
ATOM 3101 C TYR A 366 29.173 15.438 55.998 1.00 28.75
C
ATOM 3102 O TYR A 366 30.274 15.149 55.532 1.00 29.49
O
ATOM 3103 CB TYR A 366 27.192 13.953 55.355 1.00 27.69
C
ATOM 3104 CG TYR A 366 27.782 13.511 54.020 1.00 27.26
C
ATOM 3105 CD1 TYR A 366 28.285 14.461 53.107 1.00 27.40
C
ATOM 3106 CD2 TYR A 366 27.866 12.140 53.700 1.00 27.61
C
ATOM 3107 CE1 TYR A 366 28.925 14.045 51.925 1.00 28.23
C
ATOM 3108 CE2 TYR A 366 28.474 11.721 52.501 1.00 28.00
C
ATOM 3109 CZ TYR A 366 29.025 12.675 51.624 1.00 28.28
C
ATOM 3110 OH TYR A 366 29.662 12.279 50.486 1.00 28.34
O
ATOM 3111 H TYR A 366 26.572 14.709 57.695 1.00 0.00
H
ATOM 3112 HA TYR A 366 28.853 13.417 56.600 1.00 0.00

H
ATOM 3113 HB3 TYR A 366 26.531 14.802 55.176 1.00 0.00
H
ATOM 3114 HB2 TYR A 366 26.548 13.151 55.717 1.00 0.00
H
ATOM 3115 HD1 TYR A 366 28.219 15.515 53.329 1.00 0.00
H
ATOM 3116 HD2 TYR A 366 27.480 11.405 54.386 1.00 0.00
H
ATOM 3117 HE1 TYR A 366 29.337 14.784 51.254 1.00 0.00
H
ATOM 3118 HE2 TYR A 366 28.539 10.667 52.270 1.00 0.00
H
ATOM 3119 HH TYR A 366 29.447 11.380 50.205 1.00 0.00
H
ATOM 3120 N ILE A 367 28.730 16.697 56.156 1.00 30.76
N
ATOM 3121 CA ILE A 367 29.488 17.908 55.821 1.00 32.74
C
ATOM 3122 C ILE A 367 30.790 18.070 56.638 1.00 35.15
C
ATOM 3123 O ILE A 367 31.762 18.612 56.113 1.00 35.56
O
ATOM 3124 CB ILE A 367 28.575 19.171 55.934 1.00 32.16
C
ATOM 3125 CG1 ILE A 367 27.620 19.227 54.719 1.00 32.04
C
ATOM 3126 CG2 ILE A 367 29.290 20.533 56.102 1.00 32.16
C
ATOM 3127 CD1 ILE A 367 26.403 20.136 54.916 1.00 32.66
C
ATOM 3128 H ILE A 367 27.798 16.849 56.519 1.00 0.00
H
ATOM 3129 HA ILE A 367 29.791 17.807 54.777 1.00 0.00
H
ATOM 3130 HB ILE A 367 27.957 19.038 56.824 1.00 0.00

H
ATOM 3131 HG13 ILE A 367 27.257 18.229 54.472 1.00 0.00
H
ATOM 3132 HG12 ILE A 367 28.173 19.561 53.840 1.00 0.00
H
ATOM 3133 HG21 ILE A 367 28.576 21.355 56.139 1.00 0.00
H
ATOM 3134 HG22 ILE A 367 29.860 20.587 57.030 1.00 0.00
H
ATOM 3135 HG23 ILE A 367 29.972 20.725 55.273 1.00 0.00
H
ATOM 3136 HD11 ILE A 367 25.621 19.896 54.200 1.00 0.00
H
ATOM 3137 HD12 ILE A 367 25.977 20.030 55.914 1.00 0.00
H
ATOM 3138 HD13 ILE A 367 26.669 21.179 54.758 1.00 0.00
H
ATOM 3139 N ARG A 368 30.801 17.556 57.879 1.00 38.07
N
ATOM 3140 CA ARG A 368 31.978 17.521 58.748 1.00 41.56
C
ATOM 3141 C ARG A 368 32.827 16.248 58.546 1.00 43.08
C
ATOM 3142 O ARG A 368 34.029 16.302 58.795 1.00 43.32
O
ATOM 3143 CB ARG A 368 31.525 17.654 60.217 1.00 43.01
C
ATOM 3144 CG ARG A 368 30.842 19.003 60.528 1.00 46.92
C
ATOM 3145 CD ARG A 368 30.241 19.094 61.940 1.00 50.17
C
ATOM 3146 NE ARG A 368 29.051 18.242 62.084 1.00 53.52
N
ATOM 3147 CZ ARG A 368 28.343 18.060 63.212 1.00 54.12
C
ATOM 3148 NH1 ARG A 368 28.678 18.681 64.351 1.00 55.63

N
ATOM 3149 NH2 ARG A 368 27.279 17.246 63.198 1.00 55.17
N1+
ATOM 3150 H ARG A 368 29.962 17.125 58.242 1.00 0.00
H
ATOM 3151 HA ARG A 368 32.618 18.376 58.521 1.00 0.00
H
ATOM 3152 HB3 ARG A 368 32.393 17.553 60.870 1.00 0.00
H
ATOM 3153 HB2 ARG A 368 30.856 16.829 60.467 1.00 0.00
H
ATOM 3154 HG3 ARG A 368 30.015 19.119 59.828 1.00 0.00
H
ATOM 3155 HG2 ARG A 368 31.502 19.849 60.336 1.00 0.00
H
ATOM 3156 HD3 ARG A 368 30.060 20.128 62.235 1.00 0.00
H
ATOM 3157 HD2 ARG A 368 30.965 18.691 62.648 1.00 0.00
H
ATOM 3158 HE ARG A 368 28.713 17.819 61.229 1.00 0.00
H
ATOM 3159 HH12 ARG A 368 28.129 18.554 65.189 1.00 0.00
H
ATOM 3160 HH11 ARG A 368 29.475 19.301 64.372 1.00 0.00
H
ATOM 3161 HH22 ARG A 368 26.730 17.107 64.034 1.00 0.00
H
ATOM 3162 HH21 ARG A 368 27.019 16.750 62.357 1.00 0.00
H
ATOM 3163 N CYS A 369 32.206 15.136 58.117 1.00 44.44
N
ATOM 3164 CA CYS A 369 32.832 13.816 58.016 1.00 46.85
C
ATOM 3165 C CYS A 369 33.509 13.583 56.652 1.00 46.74
C
ATOM 3166 O CYS A 369 34.693 13.247 56.630 1.00 46.26

O

ATOM 3167 CB CYS A 369 31.821 12.695 58.354 1.00 48.76
C
ATOM 3168 SG CYS A 369 32.576 11.043 58.420 1.00 56.08
S
ATOM 3169 H CYS A 369 31.212 15.166 57.936 1.00 0.00
H
ATOM 3170 HA CYS A 369 33.615 13.759 58.776 1.00 0.00
H
ATOM 3171 HB3 CYS A 369 31.001 12.681 57.635 1.00 0.00
H
ATOM 3172 HB2 CYS A 369 31.374 12.879 59.327 1.00 0.00
H
ATOM 3173 HG CYS A 369 33.331 11.282 59.495 1.00 0.00
H
ATOM 3174 N ARG A 370 32.739 13.715 55.559 1.00 47.07
N
ATOM 3175 CA ARG A 370 33.094 13.221 54.224 1.00 47.21
C
ATOM 3176 C ARG A 370 33.379 14.324 53.194 1.00 47.24
C
ATOM 3177 O ARG A 370 34.073 14.026 52.221 1.00 46.93
O
ATOM 3178 CB ARG A 370 31.962 12.307 53.702 1.00 47.76
C
ATOM 3179 CG ARG A 370 31.658 11.090 54.592 1.00 49.65
C
ATOM 3180 CD ARG A 370 32.739 9.997 54.542 1.00 51.30
C
ATOM 3181 NE ARG A 370 32.555 9.010 55.616 1.00 52.96
N
ATOM 3182 CZ ARG A 370 31.651 8.017 55.667 1.00 53.67
C
ATOM 3183 NH1 ARG A 370 30.814 7.763 54.651 1.00 54.23
N
ATOM 3184 NH2 ARG A 370 31.592 7.261 56.769 1.00 54.08

N1+
ATOM 3185 H ARG A 370 31.789 14.054 55.659 1.00 0.00
H
ATOM 3186 HA ARG A 370 34.009 12.630 54.285 1.00 0.00
H
ATOM 3187 HB3 ARG A 370 32.193 11.959 52.694 1.00 0.00
H
ATOM 3188 HB2 ARG A 370 31.053 12.903 53.615 1.00 0.00
H
ATOM 3189 HG3 ARG A 370 30.753 10.650 54.173 1.00 0.00
H
ATOM 3190 HG2 ARG A 370 31.401 11.364 55.613 1.00 0.00
H
ATOM 3191 HD3 ARG A 370 33.744 10.416 54.552 1.00 0.00
H
ATOM 3192 HD2 ARG A 370 32.653 9.438 53.610 1.00 0.00
H
ATOM 3193 HE ARG A 370 33.126 9.168 56.434 1.00 0.00
H
ATOM 3194 HH12 ARG A 370 30.150 7.004 54.714 1.00 0.00
H
ATOM 3195 HH11 ARG A 370 30.854 8.320 53.810 1.00 0.00
H
ATOM 3196 HH22 ARG A 370 30.915 6.507 56.839 1.00 0.00
H
ATOM 3197 HH21 ARG A 370 32.219 7.426 57.542 1.00 0.00
H
ATOM 3198 N HIS A 371 32.866 15.553 53.388 1.00 46.83
N
ATOM 3199 CA HIS A 371 33.127 16.668 52.470 1.00 47.01
C
ATOM 3200 C HIS A 371 34.574 17.181 52.677 1.00 47.99
C
ATOM 3201 O HIS A 371 34.911 17.528 53.810 1.00 46.99
O
ATOM 3202 CB HIS A 371 32.085 17.780 52.696 1.00 45.11

C
ATOM 3203 CG HIS A 371 32.026 18.775 51.558 1.00 44.21
C
ATOM 3204 ND1 HIS A 371 30.969 18.803 50.664 1.00 43.28
N1+
ATOM 3205 CD2 HIS A 371 32.853 19.833 51.226 1.00 43.81
C
ATOM 3206 CE1 HIS A 371 31.203 19.785 49.800 1.00 43.79
C
ATOM 3207 NE2 HIS A 371 32.326 20.437 50.095 1.00 43.86
N
ATOM 3208 H HIS A 371 32.300 15.749 54.203 1.00 0.00
H
ATOM 3209 HA HIS A 371 32.959 16.279 51.467 1.00 0.00
H
ATOM 3210 HB3 HIS A 371 32.282 18.320 53.621 1.00 0.00
H
ATOM 3211 HB2 HIS A 371 31.095 17.336 52.804 1.00 0.00
H
ATOM 3212 HD1 HIS A 371 30.149 18.200 50.672 1.00 0.00
H
ATOM 3213 HD2 HIS A 371 33.764 20.197 51.678 1.00 0.00
H
ATOM 3214 HE1 HIS A 371 30.544 20.041 48.984 1.00 0.00
H
ATOM 3215 HE2 HIS A 371 32.707 21.230 49.599 1.00 0.00
H
ATOM 3216 N PRO A 372 35.417 17.171 51.615 1.00 49.67
N
ATOM 3217 CA PRO A 372 36.862 17.441 51.749 1.00 51.25
C
ATOM 3218 C PRO A 372 37.195 18.919 52.059 1.00 52.68
C
ATOM 3219 O PRO A 372 36.358 19.791 51.814 1.00 52.39
O
ATOM 3220 CB PRO A 372 37.421 17.012 50.379 1.00 51.31

C
ATOM 3221 CG PRO A 372 36.281 17.255 49.406 1.00 51.05
C
ATOM 3222 CD PRO A 372 35.060 16.870 50.227 1.00 50.21
C
ATOM 3223 HA PRO A 372 37.265 16.799 52.534 1.00 0.00
H
ATOM 3224 HB3 PRO A 372 37.660 15.948 50.405 1.00 0.00
H
ATOM 3225 HB2 PRO A 372 38.329 17.541 50.083 1.00 0.00
H
ATOM 3226 HG3 PRO A 372 36.372 16.694 48.476 1.00 0.00
H
ATOM 3227 HG2 PRO A 372 36.235 18.317 49.157 1.00 0.00
H
ATOM 3228 HD2 PRO A 372 34.178 17.414 49.889 1.00 0.00
H
ATOM 3229 HD3 PRO A 372 34.871 15.800 50.137 1.00 0.00
H
ATOM 3230 N PRO A 373 38.427 19.183 52.556 1.00 54.03
N
ATOM 3231 CA PRO A 373 38.881 20.558 52.833 1.00 55.28
C
ATOM 3232 C PRO A 373 39.178 21.363 51.542 1.00 56.14
C
ATOM 3233 O PRO A 373 39.490 20.753 50.517 1.00 56.44
O
ATOM 3234 CB PRO A 373 40.156 20.336 53.666 1.00 55.37
C
ATOM 3235 CG PRO A 373 40.708 19.004 53.185 1.00 55.24
C
ATOM 3236 CD PRO A 373 39.446 18.193 52.917 1.00 54.59
C
ATOM 3237 HA PRO A 373 38.129 21.053 53.445 1.00 0.00
H
ATOM 3238 HB3 PRO A 373 39.887 20.260 54.721 1.00 0.00

H
ATOM 3239 HB2 PRO A 373 40.886 21.142 53.578 1.00 0.00
H
ATOM 3240 HG3 PRO A 373 41.385 18.529 53.895 1.00 0.00
H
ATOM 3241 HG2 PRO A 373 41.251 19.152 52.251 1.00 0.00
H
ATOM 3242 HD2 PRO A 373 39.620 17.447 52.142 1.00 0.00
H
ATOM 3243 HD3 PRO A 373 39.126 17.679 53.824 1.00 0.00
H
ATOM 3244 N PRO A 374 39.135 22.716 51.616 1.00 56.81
N
ATOM 3245 CA PRO A 374 38.712 23.534 52.770 1.00 57.49
C
ATOM 3246 C PRO A 374 37.215 23.575 53.018 1.00 57.83
C
ATOM 3247 O PRO A 374 36.709 24.231 53.927 1.00 58.58
O
ATOM 3248 CB PRO A 374 39.282 24.924 52.418 1.00 57.24
C
ATOM 3249 CG PRO A 374 39.218 24.986 50.900 1.00 57.37
C
ATOM 3250 CD PRO A 374 39.549 23.556 50.488 1.00 57.05
C
ATOM 3251 HA PRO A 374 39.202 23.187 53.682 1.00 0.00
H
ATOM 3252 HXT PRO A 374 36.609 22.983 52.331 1.00 0.00
H
ATOM 3253 HB3 PRO A 374 40.325 24.978 52.736 1.00 0.00
H
ATOM 3254 HB2 PRO A 374 38.766 25.762 52.891 1.00 0.00
H
ATOM 3255 HG3 PRO A 374 39.888 25.728 50.465 1.00 0.00
H
ATOM 3256 HG2 PRO A 374 38.201 25.230 50.590 1.00 0.00

H
ATOM 3257 HD2 PRO A 374 39.053 23.282 49.555 1.00 0.00
H
ATOM 3258 HD3 PRO A 374 40.625 23.442 50.347 1.00 0.00
H
ATOM 3259 N LEU A 378 30.743 25.505 57.256 1.00 49.88
N
ATOM 3260 CA LEU A 378 29.702 26.546 57.115 1.00 45.66
C
ATOM 3261 C LEU A 378 28.671 26.149 56.018 1.00 41.60
C
ATOM 3262 O LEU A 378 27.602 26.760 55.978 1.00 38.14
O
ATOM 3263 CB LEU A 378 30.353 27.936 56.869 1.00 53.84
C
ATOM 3264 CG LEU A 378 29.420 29.174 56.930 1.00 57.56
C
ATOM 3265 CD1 LEU A 378 28.710 29.309 58.294 1.00 59.64
C
ATOM 3266 CD2 LEU A 378 30.183 30.459 56.546 1.00 59.59
C
ATOM 3267 H1 LEU A 378 31.371 25.746 58.010 1.00 0.00
H
ATOM 3268 H2 LEU A 378 30.326 24.629 57.540 1.00 0.00
H
ATOM 3269 HA LEU A 378 29.159 26.582 58.060 1.00 0.00
H
ATOM 3270 HB3 LEU A 378 30.879 27.916 55.913 1.00 0.00
H
ATOM 3271 HB2 LEU A 378 31.134 28.088 57.616 1.00 0.00
H
ATOM 3272 HG LEU A 378 28.645 29.056 56.175 1.00 0.00
H
ATOM 3273 HD11 LEU A 378 28.671 30.340 58.647 1.00 0.00
H
ATOM 3274 HD12 LEU A 378 27.680 28.959 58.228 1.00 0.00

H
ATOM 3275 HD13 LEU A 378 29.203 28.724 59.072 1.00 0.00
H
ATOM 3276 HD21 LEU A 378 29.649 31.010 55.771 1.00 0.00
H
ATOM 3277 HD22 LEU A 378 30.317 31.136 57.390 1.00 0.00
H
ATOM 3278 HD23 LEU A 378 31.179 30.245 56.157 1.00 0.00
H
ATOM 3279 N LEU A 379 28.973 25.147 55.164 1.00 35.28
N
ATOM 3280 CA LEU A 379 28.152 24.669 54.043 1.00 33.21
C
ATOM 3281 C LEU A 379 26.717 24.254 54.423 1.00 32.24
C
ATOM 3282 O LEU A 379 25.818 24.435 53.606 1.00 31.09
O
ATOM 3283 CB LEU A 379 28.908 23.527 53.317 1.00 33.21
C
ATOM 3284 CG LEU A 379 28.239 22.968 52.035 1.00 31.83
C
ATOM 3285 CD1 LEU A 379 28.062 24.048 50.944 1.00 33.04
C
ATOM 3286 CD2 LEU A 379 28.985 21.723 51.513 1.00 32.12
C
ATOM 3287 H LEU A 379 29.871 24.696 55.272 1.00 0.00
H
ATOM 3288 HA LEU A 379 28.064 25.513 53.359 1.00 0.00
H
ATOM 3289 HB3 LEU A 379 29.046 22.707 54.023 1.00 0.00
H
ATOM 3290 HB2 LEU A 379 29.912 23.869 53.062 1.00 0.00
H
ATOM 3291 HG LEU A 379 27.244 22.610 52.298 1.00 0.00
H
ATOM 3292 HD11 LEU A 379 28.382 23.709 49.959 1.00 0.00

H
ATOM 3293 HD12 LEU A 379 27.015 24.337 50.852 1.00 0.00
H
ATOM 3294 HD13 LEU A 379 28.630 24.951 51.164 1.00 0.00
H
ATOM 3295 HD21 LEU A 379 28.306 20.874 51.419 1.00 0.00
H
ATOM 3296 HD22 LEU A 379 29.434 21.883 50.533 1.00 0.00
H
ATOM 3297 HD23 LEU A 379 29.789 21.413 52.181 1.00 0.00
H
ATOM 3298 N TYR A 380 26.520 23.747 55.653 1.00 31.72
N
ATOM 3299 CA TYR A 380 25.215 23.374 56.205 1.00 32.34
C
ATOM 3300 C TYR A 380 24.222 24.553 56.258 1.00 32.63
C
ATOM 3301 O TYR A 380 23.079 24.388 55.836 1.00 31.56
O
ATOM 3302 CB TYR A 380 25.428 22.705 57.580 1.00 33.03
C
ATOM 3303 CG TYR A 380 24.164 22.352 58.346 1.00 34.66
C
ATOM 3304 CD1 TYR A 380 23.274 21.378 57.844 1.00 35.98
C
ATOM 3305 CD2 TYR A 380 23.867 23.010 59.558 1.00 35.42
C
ATOM 3306 CE1 TYR A 380 22.091 21.073 58.545 1.00 36.98
C
ATOM 3307 CE2 TYR A 380 22.690 22.700 60.262 1.00 36.43
C
ATOM 3308 CZ TYR A 380 21.802 21.732 59.757 1.00 37.93
C
ATOM 3309 OH TYR A 380 20.664 21.433 60.445 1.00 38.77
O
ATOM 3310 H TYR A 380 27.311 23.633 56.269 1.00 0.00

H
ATOM 3311 HA TYR A 380 24.788 22.627 55.533 1.00 0.00
H
ATOM 3312 HB3 TYR A 380 26.033 23.362 58.207 1.00 0.00
H
ATOM 3313 HB2 TYR A 380 26.015 21.794 57.463 1.00 0.00
H
ATOM 3314 HD1 TYR A 380 23.492 20.868 56.916 1.00 0.00
H
ATOM 3315 HD2 TYR A 380 24.539 23.759 59.951 1.00 0.00
H
ATOM 3316 HE1 TYR A 380 21.410 20.330 58.155 1.00 0.00
H
ATOM 3317 HE2 TYR A 380 22.473 23.208 61.191 1.00 0.00
H
ATOM 3318 HH TYR A 380 20.544 21.968 61.233 1.00 0.00
H
ATOM 3319 N ALA A 381 24.692 25.726 56.716 1.00 32.03
N
ATOM 3320 CA ALA A 381 23.919 26.969 56.745 1.00 32.04
C
ATOM 3321 C ALA A 381 23.659 27.556 55.346 1.00 31.59
C
ATOM 3322 O ALA A 381 22.594 28.134 55.137 1.00 32.20
O
ATOM 3323 CB ALA A 381 24.643 27.992 57.632 1.00 32.84
C
ATOM 3324 H ALA A 381 25.656 25.795 57.014 1.00 0.00
H
ATOM 3325 HA ALA A 381 22.951 26.752 57.202 1.00 0.00
H
ATOM 3326 HB1 ALA A 381 24.062 28.911 57.727 1.00 0.00
H
ATOM 3327 HB2 ALA A 381 24.800 27.600 58.637 1.00 0.00
H
ATOM 3328 HB3 ALA A 381 25.617 28.258 57.221 1.00 0.00

H
ATOM 3329 N LYS A 382 24.616 27.376 54.416 1.00 30.09
N
ATOM 3330 CA LYS A 382 24.517 27.827 53.025 1.00 30.96
C
ATOM 3331 C LYS A 382 23.482 27.031 52.210 1.00 30.08
C
ATOM 3332 O LYS A 382 22.801 27.630 51.380 1.00 30.60
O
ATOM 3333 CB LYS A 382 25.898 27.765 52.342 1.00 31.98
C
ATOM 3334 CG LYS A 382 26.961 28.671 52.984 1.00 34.76
C
ATOM 3335 CD LYS A 382 28.310 28.597 52.253 1.00 38.05
C
ATOM 3336 CE LYS A 382 29.399 29.427 52.945 1.00 39.31
C
ATOM 3337 NZ LYS A 382 30.671 29.372 52.206 1.00 41.47
N1+
ATOM 3338 H LYS A 382 25.463 26.886 54.669 1.00 0.00
H
ATOM 3339 HA LYS A 382 24.193 28.870 53.037 1.00 0.00
H
ATOM 3340 HB3 LYS A 382 25.787 28.066 51.300 1.00 0.00
H
ATOM 3341 HB2 LYS A 382 26.258 26.736 52.320 1.00 0.00
H
ATOM 3342 HG3 LYS A 382 27.102 28.395 54.028 1.00 0.00
H
ATOM 3343 HG2 LYS A 382 26.605 29.701 52.989 1.00 0.00
H
ATOM 3344 HD3 LYS A 382 28.181 28.946 51.227 1.00 0.00
H
ATOM 3345 HD2 LYS A 382 28.631 27.557 52.184 1.00 0.00
H
ATOM 3346 HE3 LYS A 382 29.569 29.053 53.954 1.00 0.00

H
ATOM 3347 HE2 LYS A 382 29.087 30.468 53.033 1.00 0.00
H
ATOM 3348 HZ1 LYS A 382 30.988 28.415 52.148 1.00 0.00
H
ATOM 3349 HZ2 LYS A 382 30.538 29.739 51.275 1.00 0.00
H
ATOM 3350 HZ3 LYS A 382 31.365 29.926 52.688 1.00 0.00
H
ATOM 3351 N MET A 383 23.369 25.717 52.475 1.00 29.65
N
ATOM 3352 CA MET A 383 22.383 24.817 51.871 1.00 29.41
C
ATOM 3353 C MET A 383 20.957 25.073 52.376 1.00 29.82
C
ATOM 3354 O MET A 383 20.032 25.072 51.568 1.00 29.12
O
ATOM 3355 CB MET A 383 22.779 23.355 52.132 1.00 28.17
C
ATOM 3356 CG MET A 383 23.978 22.890 51.297 1.00 28.12
C
ATOM 3357 SD MET A 383 24.557 21.225 51.711 1.00 28.48
S
ATOM 3358 CE MET A 383 23.135 20.264 51.139 1.00 28.89
C
ATOM 3359 H MET A 383 23.990 25.298 53.155 1.00 0.00
H
ATOM 3360 HA MET A 383 22.380 24.981 50.792 1.00 0.00
H
ATOM 3361 HB3 MET A 383 21.929 22.712 51.900 1.00 0.00
H
ATOM 3362 HB2 MET A 383 22.987 23.203 53.193 1.00 0.00
H
ATOM 3363 HG3 MET A 383 24.814 23.575 51.415 1.00 0.00
H
ATOM 3364 HG2 MET A 383 23.719 22.913 50.239 1.00 0.00

H
ATOM 3365 HE1 MET A 383 23.351 19.203 51.218 1.00 0.00
H
ATOM 3366 HE2 MET A 383 22.255 20.474 51.744 1.00 0.00
H
ATOM 3367 HE3 MET A 383 22.905 20.486 50.099 1.00 0.00
H
ATOM 3368 N ILE A 384 20.803 25.316 53.689 1.00 30.76
N
ATOM 3369 CA ILE A 384 19.526 25.676 54.311 1.00 32.84
C
ATOM 3370 C ILE A 384 19.014 27.069 53.867 1.00 32.96
C
ATOM 3371 O ILE A 384 17.802 27.274 53.834 1.00 32.83
O
ATOM 3372 CB ILE A 384 19.617 25.563 55.865 1.00 34.53
C
ATOM 3373 CG1 ILE A 384 19.786 24.089 56.320 1.00 36.66
C
ATOM 3374 CG2 ILE A 384 18.487 26.249 56.668 1.00 36.24
C
ATOM 3375 CD1 ILE A 384 18.608 23.149 56.011 1.00 37.66
C
ATOM 3376 H ILE A 384 21.606 25.283 54.302 1.00 0.00
H
ATOM 3377 HA ILE A 384 18.782 24.958 53.961 1.00 0.00
H
ATOM 3378 HB ILE A 384 20.538 26.069 56.162 1.00 0.00
H
ATOM 3379 HG13 ILE A 384 20.000 24.058 57.389 1.00 0.00
H
ATOM 3380 HG12 ILE A 384 20.667 23.662 55.844 1.00 0.00
H
ATOM 3381 HG21 ILE A 384 18.582 26.037 57.733 1.00 0.00
H
ATOM 3382 HG22 ILE A 384 18.516 27.333 56.563 1.00 0.00

H
ATOM 3383 HG23 ILE A 384 17.501 25.911 56.350 1.00 0.00
H
ATOM 3384 HD11 ILE A 384 18.600 22.304 56.701 1.00 0.00
H
ATOM 3385 HD12 ILE A 384 17.643 23.645 56.094 1.00 0.00
H
ATOM 3386 HD13 ILE A 384 18.689 22.742 55.003 1.00 0.00
H
ATOM 3387 N GLN A 385 19.931 27.966 53.458 1.00 32.82
N
ATOM 3388 CA GLN A 385 19.616 29.261 52.850 1.00 33.03
C
ATOM 3389 C GLN A 385 19.086 29.142 51.402 1.00 32.26
C
ATOM 3390 O GLN A 385 18.339 30.024 50.981 1.00 30.38
O
ATOM 3391 CB GLN A 385 20.861 30.175 52.956 1.00 35.19
C
ATOM 3392 CG GLN A 385 20.690 31.641 52.492 1.00 39.54
C
ATOM 3393 CD GLN A 385 19.583 32.412 53.223 1.00 41.95
C
ATOM 3394 OE1 GLN A 385 18.740 33.041 52.590 1.00 44.07
O
ATOM 3395 NE2 GLN A 385 19.581 32.377 54.557 1.00 42.90
N
ATOM 3396 H GLN A 385 20.912 27.733 53.523 1.00 0.00
H
ATOM 3397 HA GLN A 385 18.815 29.705 53.444 1.00 0.00
H
ATOM 3398 HB3 GLN A 385 21.681 29.734 52.393 1.00 0.00
H
ATOM 3399 HB2 GLN A 385 21.200 30.184 53.993 1.00 0.00
H
ATOM 3400 HG3 GLN A 385 20.492 31.674 51.420 1.00 0.00

H
ATOM 3401 HG2 GLN A 385 21.628 32.177 52.638 1.00 0.00
H
ATOM 3402 HE22 GLN A 385 18.868 32.874 55.071 1.00 0.00
H
ATOM 3403 HE21 GLN A 385 20.286 31.858 55.060 1.00 0.00
H
ATOM 3404 N LYS A 386 19.417 28.049 50.683 1.00 30.96
N
ATOM 3405 CA LYS A 386 18.863 27.752 49.352 1.00 30.88
C
ATOM 3406 C LYS A 386 17.367 27.397 49.377 1.00 29.98
C
ATOM 3407 O LYS A 386 16.711 27.553 48.349 1.00 29.72
O
ATOM 3408 CB LYS A 386 19.654 26.625 48.648 1.00 32.29
C
ATOM 3409 CG LYS A 386 21.152 26.894 48.423 1.00 35.09
C
ATOM 3410 CD LYS A 386 21.479 28.276 47.834 1.00 36.92
C
ATOM 3411 CE LYS A 386 22.955 28.438 47.462 1.00 39.17
C
ATOM 3412 NZ LYS A 386 23.218 29.791 46.946 1.00 40.63
N1+
ATOM 3413 H LYS A 386 20.026 27.349 51.083 1.00 0.00
H
ATOM 3414 HA LYS A 386 18.942 28.656 48.749 1.00 0.00
H
ATOM 3415 HB3 LYS A 386 19.202 26.435 47.675 1.00 0.00
H
ATOM 3416 HB2 LYS A 386 19.540 25.686 49.189 1.00 0.00
H
ATOM 3417 HG3 LYS A 386 21.535 26.120 47.759 1.00 0.00
H
ATOM 3418 HG2 LYS A 386 21.683 26.759 49.360 1.00 0.00

H

ATOM	3419	HD3	LYS	A	386	21.218	29.048	48.559	1.00	0.00
H										
ATOM	3420	HD2	LYS	A	386	20.864	28.460	46.954	1.00	0.00
H										
ATOM	3421	HE3	LYS	A	386	23.234	27.716	46.695	1.00	0.00
H										
ATOM	3422	HE2	LYS	A	386	23.590	28.258	48.330	1.00	0.00
H										
ATOM	3423	HZ1	LYS	A	386	22.993	30.471	47.659	1.00	0.00
H										
ATOM	3424	HZ2	LYS	A	386	24.192	29.871	46.692	1.00	0.00
H										
ATOM	3425	HZ3	LYS	A	386	22.639	29.957	46.135	1.00	0.00
H										
ATOM	3426	N	LEU	A	387	16.849	26.964	50.541	1.00	29.25
N										
ATOM	3427	CA	LEU	A	387	15.424	26.725	50.769	1.00	29.50
C										
ATOM	3428	C	LEU	A	387	14.613	28.035	50.794	1.00	29.30
C										
ATOM	3429	O	LEU	A	387	13.477	28.027	50.327	1.00	27.98
O										
ATOM	3430	CB	LEU	A	387	15.212	25.923	52.071	1.00	30.49
C										
ATOM	3431	CG	LEU	A	387	15.903	24.540	52.101	1.00	31.01
C										
ATOM	3432	CD1	LEU	A	387	15.806	23.914	53.502	1.00	33.01
C										
ATOM	3433	CD2	LEU	A	387	15.372	23.578	51.017	1.00	31.27
C										
ATOM	3434	H	LEU	A	387	17.453	26.857	51.344	1.00	0.00
H										
ATOM	3435	HA	LEU	A	387	15.052	26.122	49.941	1.00	0.00
H										
ATOM	3436	HB3	LEU	A	387	14.142	25.779	52.235	1.00	0.00

H
ATOM 3437 HB2 LEU A 387 15.554 26.524 52.913 1.00 0.00
H
ATOM 3438 HG LEU A 387 16.967 24.693 51.915 1.00 0.00
H
ATOM 3439 HD11 LEU A 387 16.650 23.250 53.687 1.00 0.00
H
ATOM 3440 HD12 LEU A 387 15.810 24.670 54.288 1.00 0.00
H
ATOM 3441 HD13 LEU A 387 14.891 23.333 53.618 1.00 0.00
H
ATOM 3442 HD21 LEU A 387 15.204 22.574 51.409 1.00 0.00
H
ATOM 3443 HD22 LEU A 387 14.427 23.918 50.592 1.00 0.00
H
ATOM 3444 HD23 LEU A 387 16.085 23.487 50.197 1.00 0.00
H
ATOM 3445 N ALA A 388 15.210 29.138 51.283 1.00 28.72
N
ATOM 3446 CA ALA A 388 14.614 30.479 51.263 1.00 29.54
C
ATOM 3447 C ALA A 388 14.549 31.084 49.848 1.00 29.67
C
ATOM 3448 O ALA A 388 13.566 31.755 49.530 1.00 29.63
O
ATOM 3449 CB ALA A 388 15.390 31.400 52.217 1.00 30.01
C
ATOM 3450 H ALA A 388 16.156 29.078 51.634 1.00 0.00
H
ATOM 3451 HA ALA A 388 13.592 30.395 51.638 1.00 0.00
H
ATOM 3452 HB1 ALA A 388 14.917 32.380 52.286 1.00 0.00
H
ATOM 3453 HB2 ALA A 388 15.428 30.983 53.224 1.00 0.00
H
ATOM 3454 HB3 ALA A 388 16.415 31.556 51.884 1.00 0.00

H
ATOM 3455 N ASP A 389 15.565 30.789 49.016 1.00 29.62
N
ATOM 3456 CA ASP A 389 15.610 31.121 47.586 1.00 30.08
C
ATOM 3457 C ASP A 389 14.530 30.366 46.788 1.00 29.78
C
ATOM 3458 O ASP A 389 13.896 30.963 45.922 1.00 29.18
O
ATOM 3459 CB ASP A 389 16.995 30.881 46.932 1.00 31.12
C
ATOM 3460 CG ASP A 389 18.209 31.458 47.671 1.00 34.91
C
ATOM 3461 OD1 ASP A 389 19.323 30.958 47.392 1.00 36.63
O
ATOM 3462 OD2 ASP A 389 18.030 32.430 48.436 1.00 35.52
O1-
ATOM 3463 H ASP A 389 16.354 30.267 49.371 1.00 0.00
H
ATOM 3464 HA ASP A 389 15.387 32.186 47.498 1.00 0.00
H
ATOM 3465 HB3 ASP A 389 16.993 31.288 45.920 1.00 0.00
H
ATOM 3466 HB2 ASP A 389 17.149 29.805 46.853 1.00 0.00
H
ATOM 3467 N LEU A 390 14.321 29.083 47.127 1.00 27.64
N
ATOM 3468 CA LEU A 390 13.293 28.193 46.579 1.00 28.50
C
ATOM 3469 C LEU A 390 11.862 28.688 46.850 1.00 27.80
C
ATOM 3470 O LEU A 390 11.044 28.640 45.934 1.00 26.98
O
ATOM 3471 CB LEU A 390 13.512 26.778 47.164 1.00 28.87
C
ATOM 3472 CG LEU A 390 14.127 25.729 46.216 1.00 31.08

C
ATOM 3473 CD1 LEU A 390 15.298 26.249 45.366 1.00 31.00
C
ATOM 3474 CD2 LEU A 390 14.523 24.474 47.021 1.00 31.75
C
ATOM 3475 H LEU A 390 14.912 28.673 47.837 1.00 0.00
H
ATOM 3476 HA LEU A 390 13.416 28.167 45.494 1.00 0.00
H
ATOM 3477 HB3 LEU A 390 12.576 26.368 47.542 1.00 0.00
H
ATOM 3478 HB2 LEU A 390 14.144 26.856 48.044 1.00 0.00
H
ATOM 3479 HG LEU A 390 13.345 25.440 45.515 1.00 0.00
H
ATOM 3480 HD11 LEU A 390 16.056 25.482 45.219 1.00 0.00
H
ATOM 3481 HD12 LEU A 390 14.957 26.544 44.373 1.00 0.00
H
ATOM 3482 HD13 LEU A 390 15.785 27.113 45.817 1.00 0.00
H
ATOM 3483 HD21 LEU A 390 14.148 23.570 46.548 1.00 0.00
H
ATOM 3484 HD22 LEU A 390 15.603 24.369 47.122 1.00 0.00
H
ATOM 3485 HD23 LEU A 390 14.116 24.490 48.033 1.00 0.00
H
ATOM 3486 N ARG A 391 11.595 29.182 48.072 1.00 27.87
N
ATOM 3487 CA ARG A 391 10.314 29.785 48.462 1.00 28.52
C
ATOM 3488 C ARG A 391 10.013 31.090 47.705 1.00 28.78
C
ATOM 3489 O ARG A 391 8.857 31.313 47.351 1.00 28.82
O
ATOM 3490 CB ARG A 391 10.294 30.037 49.984 1.00 30.35

C
ATOM 3491 CG ARG A 391 10.293 28.757 50.834 1.00 32.51
C
ATOM 3492 CD ARG A 391 8.917 28.091 50.954 1.00 34.99
C
ATOM 3493 NE ARG A 391 9.027 26.776 51.597 1.00 37.12
N
ATOM 3494 CZ ARG A 391 9.176 26.531 52.910 1.00 37.91
C
ATOM 3495 NH1 ARG A 391 9.209 27.516 53.817 1.00 38.94
N
ATOM 3496 NH2 ARG A 391 9.285 25.265 53.328 1.00 37.42
N1+
ATOM 3497 H ARG A 391 12.317 29.167 48.780 1.00 0.00
H
ATOM 3498 HA ARG A 391 9.528 29.071 48.212 1.00 0.00
H
ATOM 3499 HB3 ARG A 391 9.427 30.639 50.262 1.00 0.00
H
ATOM 3500 HB2 ARG A 391 11.167 30.636 50.249 1.00 0.00
H
ATOM 3501 HG3 ARG A 391 10.571 29.077 51.839 1.00 0.00
H
ATOM 3502 HG2 ARG A 391 11.049 28.036 50.533 1.00 0.00
H
ATOM 3503 HD3 ARG A 391 8.418 28.033 49.987 1.00 0.00
H
ATOM 3504 HD2 ARG A 391 8.267 28.686 51.596 1.00 0.00
H
ATOM 3505 HE ARG A 391 9.074 25.995 50.957 1.00 0.00
H
ATOM 3506 HH12 ARG A 391 9.336 27.292 54.802 1.00 0.00
H
ATOM 3507 HH11 ARG A 391 9.124 28.480 53.531 1.00 0.00
H
ATOM 3508 HH22 ARG A 391 9.306 25.063 54.325 1.00 0.00

H
ATOM 3509 HH21 ARG A 391 9.277 24.500 52.669 1.00 0.00
H
ATOM 3510 N SER A 392 11.052 31.902 47.441 1.00 28.76
N
ATOM 3511 CA SER A 392 10.955 33.143 46.667 1.00 29.36
C
ATOM 3512 C SER A 392 10.686 32.886 45.171 1.00 28.02
C
ATOM 3513 O SER A 392 9.916 33.630 44.562 1.00 28.38
O
ATOM 3514 CB SER A 392 12.241 33.970 46.860 1.00 31.21
C
ATOM 3515 OG SER A 392 12.310 34.474 48.178 1.00 37.19
O
ATOM 3516 H SER A 392 11.976 31.647 47.763 1.00 0.00
H
ATOM 3517 HA SER A 392 10.113 33.722 47.050 1.00 0.00
H
ATOM 3518 HB3 SER A 392 12.255 34.821 46.178 1.00 0.00
H
ATOM 3519 HB2 SER A 392 13.133 33.381 46.651 1.00 0.00
H
ATOM 3520 HG SER A 392 12.549 33.754 48.768 1.00 0.00
H
ATOM 3521 N LEU A 393 11.284 31.817 44.620 1.00 26.23
N
ATOM 3522 CA LEU A 393 11.037 31.346 43.256 1.00 26.10
C
ATOM 3523 C LEU A 393 9.627 30.747 43.103 1.00 25.57
C
ATOM 3524 O LEU A 393 8.957 31.046 42.115 1.00 26.87
O
ATOM 3525 CB LEU A 393 12.123 30.328 42.846 1.00 26.02
C
ATOM 3526 CG LEU A 393 13.515 30.943 42.576 1.00 26.66

C
ATOM 3527 CD1 LEU A 393 14.614 29.856 42.530 1.00 28.52
C
ATOM 3528 CD2 LEU A 393 13.510 31.838 41.318 1.00 29.45
C
ATOM 3529 H LEU A 393 11.957 31.283 45.160 1.00 0.00
H
ATOM 3530 HA LEU A 393 11.087 32.206 42.585 1.00 0.00
H
ATOM 3531 HB3 LEU A 393 11.805 29.809 41.943 1.00 0.00
H
ATOM 3532 HB2 LEU A 393 12.194 29.558 43.615 1.00 0.00
H
ATOM 3533 HG LEU A 393 13.762 31.586 43.421 1.00 0.00
H
ATOM 3534 HD11 LEU A 393 15.376 30.045 43.288 1.00 0.00
H
ATOM 3535 HD12 LEU A 393 14.219 28.859 42.726 1.00 0.00
H
ATOM 3536 HD13 LEU A 393 15.126 29.804 41.570 1.00 0.00
H
ATOM 3537 HD21 LEU A 393 14.334 31.625 40.640 1.00 0.00
H
ATOM 3538 HD22 LEU A 393 12.593 31.734 40.736 1.00 0.00
H
ATOM 3539 HD23 LEU A 393 13.595 32.887 41.599 1.00 0.00
H
ATOM 3540 N ASN A 394 9.176 29.962 44.095 1.00 24.50
N
ATOM 3541 CA ASN A 394 7.821 29.392 44.154 1.00 26.07
C
ATOM 3542 C ASN A 394 6.734 30.487 44.153 1.00 27.28
C
ATOM 3543 O ASN A 394 5.778 30.391 43.381 1.00 26.29
O
ATOM 3544 CB ASN A 394 7.680 28.463 45.384 1.00 26.75

C
ATOM 3545 CG ASN A 394 6.473 27.512 45.350 1.00 28.26
C
ATOM 3546 OD1 ASN A 394 5.589 27.603 44.506 1.00 28.24
O
ATOM 3547 ND2 ASN A 394 6.434 26.563 46.276 1.00 28.79
N
ATOM 3548 H ASN A 394 9.817 29.694 44.837 1.00 0.00
H
ATOM 3549 HA ASN A 394 7.652 28.798 43.254 1.00 0.00
H
ATOM 3550 HB3 ASN A 394 7.658 29.041 46.308 1.00 0.00
H
ATOM 3551 HB2 ASN A 394 8.563 27.827 45.438 1.00 0.00
H
ATOM 3552 HD22 ASN A 394 5.689 25.880 46.313 1.00 0.00
H
ATOM 3553 HD21 ASN A 394 7.194 26.461 46.943 1.00 0.00
H
ATOM 3554 N GLU A 395 6.939 31.549 44.949 1.00 28.62
N
ATOM 3555 CA GLU A 395 6.068 32.727 44.991 1.00 30.55
C
ATOM 3556 C GLU A 395 5.913 33.399 43.617 1.00 29.66
C
ATOM 3557 O GLU A 395 4.781 33.625 43.191 1.00 29.50
O
ATOM 3558 CB GLU A 395 6.590 33.730 46.047 1.00 33.44
C
ATOM 3559 CG GLU A 395 6.173 33.409 47.495 1.00 40.01
C
ATOM 3560 CD GLU A 395 4.689 33.679 47.747 1.00 44.38
C
ATOM 3561 OE1 GLU A 395 3.932 32.689 47.843 1.00 46.89
O
ATOM 3562 OE2 GLU A 395 4.335 34.877 47.822 1.00 46.99

O1-
ATOM 3563 H GLU A 395 7.760 31.584 45.539 1.00 0.00
H
ATOM 3564 HA GLU A 395 5.073 32.386 45.280 1.00 0.00
H
ATOM 3565 HB3 GLU A 395 6.271 34.745 45.802 1.00 0.00
H
ATOM 3566 HB2 GLU A 395 7.678 33.762 46.008 1.00 0.00
H
ATOM 3567 HG3 GLU A 395 6.750 34.024 48.186 1.00 0.00
H
ATOM 3568 HG2 GLU A 395 6.404 32.372 47.739 1.00 0.00
H
ATOM 3569 N GLU A 396 7.030 33.624 42.912 1.00 29.08
N
ATOM 3570 CA GLU A 396 7.019 34.155 41.549 1.00 28.78
C
ATOM 3571 C GLU A 396 6.330 33.195 40.555 1.00 28.20
C
ATOM 3572 O GLU A 396 5.442 33.625 39.816 1.00 27.96
O
ATOM 3573 CB GLU A 396 8.460 34.530 41.130 1.00 30.45
C
ATOM 3574 CG GLU A 396 8.586 35.245 39.766 1.00 30.29
C
ATOM 3575 CD GLU A 396 7.909 36.614 39.677 1.00 33.32
C
ATOM 3576 OE1 GLU A 396 7.687 37.234 40.739 1.00 32.46
O
ATOM 3577 OE2 GLU A 396 7.624 37.024 38.533 1.00 32.27
O1-
ATOM 3578 H GLU A 396 7.932 33.388 43.309 1.00 0.00
H
ATOM 3579 HA GLU A 396 6.420 35.067 41.575 1.00 0.00
H
ATOM 3580 HB3 GLU A 396 9.071 33.627 41.099 1.00 0.00

H

ATOM	3581	HB2	GLU	A	396	8.911	35.156	41.902	1.00	0.00
H										
ATOM	3582	HG3	GLU	A	396	8.158	34.621	38.985	1.00	0.00
H										
ATOM	3583	HG2	GLU	A	396	9.639	35.383	39.521	1.00	0.00
H										
ATOM	3584	N	HIS	A	397	6.705	31.905	40.604	1.00	26.94
N										
ATOM	3585	CA	HIS	A	397	6.164	30.833	39.759	1.00	27.05
C										
ATOM	3586	C	HIS	A	397	4.638	30.732	39.849	1.00	27.50
C										
ATOM	3587	O	HIS	A	397	3.973	30.765	38.817	1.00	27.05
O										
ATOM	3588	CB	HIS	A	397	6.831	29.478	40.092	1.00	26.64
C										
ATOM	3589	CG	HIS	A	397	6.313	28.296	39.301	1.00	26.04
C										
ATOM	3590	ND1	HIS	A	397	5.449	27.347	39.831	1.00	27.22
N										
ATOM	3591	CD2	HIS	A	397	6.559	27.887	38.008	1.00	26.36
C										
ATOM	3592	CE1	HIS	A	397	5.214	26.443	38.876	1.00	26.59
C										
ATOM	3593	NE2	HIS	A	397	5.855	26.710	37.746	1.00	26.30
N										
ATOM	3594	H	HIS	A	397	7.444	31.639	41.251	1.00	0.00
H										
ATOM	3595	HA	HIS	A	397	6.398	31.080	38.723	1.00	0.00
H										
ATOM	3596	HB3	HIS	A	397	6.711	29.255	41.151	1.00	0.00
H										
ATOM	3597	HB2	HIS	A	397	7.905	29.535	39.924	1.00	0.00
H										
ATOM	3598	HD1	HIS	A	397	5.072	27.317	40.768	1.00	0.00

H
ATOM 3599 HD2 HIS A 397 7.183 28.341 37.251 1.00 0.00
H
ATOM 3600 HE1 HIS A 397 4.575 25.579 39.004 1.00 0.00
H
ATOM 3601 N SER A 398 4.116 30.679 41.080 1.00 28.04
N
ATOM 3602 CA SER A 398 2.691 30.611 41.386 1.00 29.62
C
ATOM 3603 C SER A 398 1.906 31.806 40.799 1.00 29.87
C
ATOM 3604 O SER A 398 0.856 31.614 40.180 1.00 28.32
O
ATOM 3605 CB SER A 398 2.549 30.478 42.921 1.00 31.44
C
ATOM 3606 OG SER A 398 1.212 30.296 43.348 1.00 38.72
O
ATOM 3607 H SER A 398 4.747 30.676 41.881 1.00 0.00
H
ATOM 3608 HA SER A 398 2.300 29.702 40.925 1.00 0.00
H
ATOM 3609 HB3 SER A 398 2.958 31.360 43.417 1.00 0.00
H
ATOM 3610 HB2 SER A 398 3.132 29.626 43.276 1.00 0.00
H
ATOM 3611 HG SER A 398 1.220 30.146 44.301 1.00 0.00
H
ATOM 3612 N LYS A 399 2.463 33.018 40.935 1.00 30.06
N
ATOM 3613 CA LYS A 399 1.823 34.257 40.486 1.00 31.61
C
ATOM 3614 C LYS A 399 1.834 34.396 38.955 1.00 30.91
C
ATOM 3615 O LYS A 399 0.818 34.804 38.390 1.00 30.21
O
ATOM 3616 CB LYS A 399 2.495 35.454 41.186 1.00 33.79

C

ATOM	3617	CG	LYS	A	399	2.214	35.476	42.701	1.00	38.63	C
ATOM	3618	CD	LYS	A	399	3.153	36.401	43.488	1.00	41.48	C
ATOM	3619	CE	LYS	A	399	3.124	36.087	44.991	1.00	43.57	C
ATOM	3620	NZ	LYS	A	399	4.113	36.883	45.735	1.00	44.90	N1+
ATOM	3621	H	LYS	A	399	3.385	33.118	41.337	1.00	0.00	H
ATOM	3622	HA	LYS	A	399	0.777	34.229	40.801	1.00	0.00	H
ATOM	3623	HB3	LYS	A	399	2.148	36.393	40.754	1.00	0.00	H
ATOM	3624	HB2	LYS	A	399	3.571	35.414	41.003	1.00	0.00	H
ATOM	3625	HG3	LYS	A	399	2.290	34.467	43.106	1.00	0.00	H
ATOM	3626	HG2	LYS	A	399	1.180	35.778	42.873	1.00	0.00	H
ATOM	3627	HD3	LYS	A	399	2.860	37.439	43.323	1.00	0.00	H
ATOM	3628	HD2	LYS	A	399	4.173	36.307	43.111	1.00	0.00	H
ATOM	3629	HE3	LYS	A	399	3.358	35.035	45.155	1.00	0.00	H
ATOM	3630	HE2	LYS	A	399	2.132	36.262	45.407	1.00	0.00	H
ATOM	3631	HZ1	LYS	A	399	5.028	36.748	45.331	1.00	0.00	H
ATOM	3632	HZ2	LYS	A	399	3.867	37.861	45.709	1.00	0.00	H
ATOM	3633	HZ3	LYS	A	399	4.128	36.552	46.694	1.00	0.00	H
ATOM	3634	N	GLN	A	400	2.951	34.002	38.317	1.00	29.20	

N

ATOM 3635 CA GLN A 400 3.066 33.854 36.864 1.00 28.78

C

ATOM 3636 C GLN A 400 2.117 32.774 36.318 1.00 27.95

C

ATOM 3637 O GLN A 400 1.347 33.062 35.403 1.00 27.64

O

ATOM 3638 CB GLN A 400 4.521 33.555 36.443 1.00 28.49

C

ATOM 3639 CG GLN A 400 5.479 34.766 36.456 1.00 30.64

C

ATOM 3640 CD GLN A 400 6.855 34.380 35.905 1.00 31.34

C

ATOM 3641 OE1 GLN A 400 7.330 33.276 36.148 1.00 33.90

O

ATOM 3642 NE2 GLN A 400 7.524 35.248 35.154 1.00 31.02

N

ATOM 3643 H GLN A 400 3.758 33.710 38.864 1.00 0.00

H

ATOM 3644 HA GLN A 400 2.767 34.801 36.409 1.00 0.00

H

ATOM 3645 HB3 GLN A 400 4.515 33.152 35.427 1.00 0.00

H

ATOM 3646 HB2 GLN A 400 4.924 32.760 37.073 1.00 0.00

H

ATOM 3647 HG3 GLN A 400 5.604 35.143 37.472 1.00 0.00

H

ATOM 3648 HG2 GLN A 400 5.063 35.580 35.860 1.00 0.00

H

ATOM 3649 HE22 GLN A 400 8.435 34.998 34.795 1.00 0.00

H

ATOM 3650 HE21 GLN A 400 7.183 36.177 34.941 1.00 0.00

H

ATOM 3651 N TYR A 401 2.148 31.577 36.925 1.00 26.56

N

ATOM 3652 CA TYR A 401 1.321 30.421 36.566 1.00 27.31

C
ATOM 3653 C TYR A 401 -0.185 30.742 36.607 1.00 27.98
C
ATOM 3654 O TYR A 401 -0.914 30.339 35.702 1.00 27.25
O
ATOM 3655 CB TYR A 401 1.690 29.195 37.436 1.00 27.75
C
ATOM 3656 CG TYR A 401 0.811 27.968 37.240 1.00 27.44
C
ATOM 3657 CD1 TYR A 401 1.125 27.012 36.251 1.00 28.38
C
ATOM 3658 CD2 TYR A 401 -0.355 27.806 38.019 1.00 29.26
C
ATOM 3659 CE1 TYR A 401 0.274 25.909 36.038 1.00 28.43
C
ATOM 3660 CE2 TYR A 401 -1.217 26.722 37.784 1.00 29.62
C
ATOM 3661 CZ TYR A 401 -0.901 25.766 36.803 1.00 29.95
C
ATOM 3662 OH TYR A 401 -1.734 24.704 36.616 1.00 30.37
O
ATOM 3663 H TYR A 401 2.821 31.415 37.674 1.00 0.00
H
ATOM 3664 HA TYR A 401 1.579 30.164 35.539 1.00 0.00
H
ATOM 3665 HB3 TYR A 401 1.640 29.468 38.491 1.00 0.00
H
ATOM 3666 HB2 TYR A 401 2.728 28.911 37.251 1.00 0.00
H
ATOM 3667 HD1 TYR A 401 2.014 27.127 35.646 1.00 0.00
H
ATOM 3668 HD2 TYR A 401 -0.610 28.530 38.780 1.00 0.00
H
ATOM 3669 HE1 TYR A 401 0.521 25.192 35.271 1.00 0.00
H
ATOM 3670 HE2 TYR A 401 -2.116 26.622 38.372 1.00 0.00

H
ATOM 3671 HH TYR A 401 -1.339 23.861 36.882 1.00 0.00
H
ATOM 3672 N ARG A 402 -0.626 31.503 37.620 1.00 29.27
N
ATOM 3673 CA ARG A 402 -2.003 31.979 37.732 1.00 32.16
C
ATOM 3674 C ARG A 402 -2.409 32.866 36.539 1.00 32.00
C
ATOM 3675 O ARG A 402 -3.414 32.575 35.895 1.00 31.31
O
ATOM 3676 CB ARG A 402 -2.202 32.680 39.090 1.00 36.10
C
ATOM 3677 CG ARG A 402 -3.640 33.172 39.344 1.00 41.28
C
ATOM 3678 CD ARG A 402 -3.828 33.709 40.769 1.00 45.66
C
ATOM 3679 NE ARG A 402 -5.121 34.383 40.940 1.00 49.74
N
ATOM 3680 CZ ARG A 402 -5.400 35.649 40.591 1.00 51.64
C
ATOM 3681 NH1 ARG A 402 -4.472 36.433 40.030 1.00 52.67
N
ATOM 3682 NH2 ARG A 402 -6.623 36.135 40.813 1.00 52.69
N1+
ATOM 3683 H ARG A 402 0.017 31.747 38.368 1.00 0.00
H
ATOM 3684 HA ARG A 402 -2.648 31.097 37.712 1.00 0.00
H
ATOM 3685 HB3 ARG A 402 -1.519 33.527 39.165 1.00 0.00
H
ATOM 3686 HB2 ARG A 402 -1.918 31.990 39.886 1.00 0.00
H
ATOM 3687 HG3 ARG A 402 -4.276 32.292 39.240 1.00 0.00
H
ATOM 3688 HG2 ARG A 402 -3.997 33.887 38.600 1.00 0.00

H
ATOM 3689 HD3 ARG A 402 -2.975 34.272 41.150 1.00 0.00
H
ATOM 3690 HD2 ARG A 402 -3.924 32.839 41.419 1.00 0.00
H
ATOM 3691 HE ARG A 402 -5.861 33.810 41.322 1.00 0.00
H
ATOM 3692 HH12 ARG A 402 -4.680 37.387 39.772 1.00 0.00
H
ATOM 3693 HH11 ARG A 402 -3.544 36.080 39.858 1.00 0.00
H
ATOM 3694 HH22 ARG A 402 -6.858 37.086 40.567 1.00 0.00
H
ATOM 3695 HH21 ARG A 402 -7.338 35.567 41.243 1.00 0.00
H
ATOM 3696 N CYS A 403 -1.594 33.886 36.227 1.00 32.10
N
ATOM 3697 CA CYS A 403 -1.770 34.740 35.048 1.00 33.04
C
ATOM 3698 C CYS A 403 -1.770 33.932 33.732 1.00 32.14
C
ATOM 3699 O CYS A 403 -2.609 34.181 32.866 1.00 30.55
O
ATOM 3700 CB CYS A 403 -0.717 35.872 35.042 1.00 35.60
C
ATOM 3701 SG CYS A 403 -0.913 37.026 33.649 1.00 44.46
S
ATOM 3702 H CYS A 403 -0.732 34.014 36.740 1.00 0.00
H
ATOM 3703 HA CYS A 403 -2.753 35.207 35.139 1.00 0.00
H
ATOM 3704 HB3 CYS A 403 0.291 35.457 35.008 1.00 0.00
H
ATOM 3705 HB2 CYS A 403 -0.785 36.447 35.966 1.00 0.00
H
ATOM 3706 HG CYS A 403 -0.593 36.150 32.692 1.00 0.00

H
ATOM 3707 N LEU A 404 -0.835 32.976 33.622 1.00 30.59
N
ATOM 3708 CA LEU A 404 -0.670 32.061 32.495 1.00 30.86
C
ATOM 3709 C LEU A 404 -1.911 31.181 32.280 1.00 29.62
C
ATOM 3710 O LEU A 404 -2.451 31.155 31.179 1.00 30.21
O
ATOM 3711 CB LEU A 404 0.620 31.226 32.705 1.00 32.55
C
ATOM 3712 CG LEU A 404 0.920 30.151 31.637 1.00 34.87
C
ATOM 3713 CD1 LEU A 404 1.119 30.780 30.251 1.00 36.26
C
ATOM 3714 CD2 LEU A 404 2.112 29.265 32.041 1.00 35.52
C
ATOM 3715 H LEU A 404 -0.149 32.879 34.368 1.00 0.00
H
ATOM 3716 HA LEU A 404 -0.545 32.670 31.599 1.00 0.00
H
ATOM 3717 HB3 LEU A 404 0.546 30.720 33.662 1.00 0.00
H
ATOM 3718 HB2 LEU A 404 1.478 31.895 32.790 1.00 0.00
H
ATOM 3719 HG LEU A 404 0.071 29.471 31.582 1.00 0.00
H
ATOM 3720 HD11 LEU A 404 1.754 30.164 29.614 1.00 0.00
H
ATOM 3721 HD12 LEU A 404 0.165 30.901 29.740 1.00 0.00
H
ATOM 3722 HD13 LEU A 404 1.578 31.764 30.328 1.00 0.00
H
ATOM 3723 HD21 LEU A 404 2.968 29.382 31.375 1.00 0.00
H
ATOM 3724 HD22 LEU A 404 2.461 29.488 33.048 1.00 0.00

H

ATOM 3725 HD23 LEU A 404 1.836 28.210 32.029 1.00 0.00

H

ATOM 3726 N SER A 405 -2.351 30.479 33.329 1.00 29.30

N

ATOM 3727 CA SER A 405 -3.460 29.524 33.285 1.00 29.83

C

ATOM 3728 C SER A 405 -4.825 30.206 33.010 1.00 28.16

C

ATOM 3729 O SER A 405 -5.712 29.594 32.417 1.00 28.54

O

ATOM 3730 CB SER A 405 -3.408 28.692 34.590 1.00 31.58

C

ATOM 3731 OG SER A 405 -4.266 27.566 34.587 1.00 34.11

O

ATOM 3732 H SER A 405 -1.845 30.539 34.212 1.00 0.00

H

ATOM 3733 HA SER A 405 -3.272 28.848 32.449 1.00 0.00

H

ATOM 3734 HB3 SER A 405 -3.649 29.321 35.449 1.00 0.00

H

ATOM 3735 HB2 SER A 405 -2.395 28.319 34.752 1.00 0.00

H

ATOM 3736 HG SER A 405 -3.951 26.948 35.256 1.00 0.00

H

ATOM 3737 N PHE A 406 -4.949 31.489 33.381 1.00 25.99

N

ATOM 3738 CA PHE A 406 -6.119 32.341 33.145 1.00 26.56

C

ATOM 3739 C PHE A 406 -6.252 32.838 31.689 1.00 26.23

C

ATOM 3740 O PHE A 406 -7.337 33.291 31.322 1.00 25.59

O

ATOM 3741 CB PHE A 406 -6.112 33.523 34.144 1.00 26.14

C

ATOM 3742 CG PHE A 406 -6.549 33.253 35.583 1.00 28.26

C
ATOM 3743 CD1 PHE A 406 -6.814 34.354 36.426 1.00 29.88
C
ATOM 3744 CD2 PHE A 406 -6.554 31.956 36.150 1.00 29.84
C
ATOM 3745 CE1 PHE A 406 -7.117 34.158 37.766 1.00 30.77
C
ATOM 3746 CE2 PHE A 406 -6.859 31.780 37.493 1.00 31.26
C
ATOM 3747 CZ PHE A 406 -7.143 32.876 38.297 1.00 30.45
C
ATOM 3748 H PHE A 406 -4.165 31.935 33.837 1.00 0.00
H
ATOM 3749 HA PHE A 406 -7.020 31.753 33.332 1.00 0.00
H
ATOM 3750 HB3 PHE A 406 -6.746 34.329 33.772 1.00 0.00
H
ATOM 3751 HB2 PHE A 406 -5.107 33.947 34.184 1.00 0.00
H
ATOM 3752 HD1 PHE A 406 -6.776 35.359 36.030 1.00 0.00
H
ATOM 3753 HD2 PHE A 406 -6.319 31.084 35.560 1.00 0.00
H
ATOM 3754 HE1 PHE A 406 -7.326 35.007 38.401 1.00 0.00
H
ATOM 3755 HE2 PHE A 406 -6.866 30.786 37.915 1.00 0.00
H
ATOM 3756 HZ PHE A 406 -7.376 32.730 39.342 1.00 0.00
H
ATOM 3757 N GLN A 407 -5.178 32.732 30.883 1.00 25.64
N
ATOM 3758 CA GLN A 407 -5.169 33.037 29.449 1.00 25.17
C
ATOM 3759 C GLN A 407 -6.008 31.979 28.688 1.00 25.33
C
ATOM 3760 O GLN A 407 -5.783 30.790 28.922 1.00 24.86

O

ATOM 3761 CB GLN A 407 -3.695 33.007 28.969 1.00 25.99

C

ATOM 3762 CG GLN A 407 -3.445 33.456 27.513 1.00 26.23

C

ATOM 3763 CD GLN A 407 -3.750 34.932 27.242 1.00 26.81

C

ATOM 3764 OE1 GLN A 407 -3.811 35.752 28.155 1.00 28.94

O

ATOM 3765 NE2 GLN A 407 -3.913 35.284 25.965 1.00 21.63

N

ATOM 3766 H GLN A 407 -4.320 32.346 31.255 1.00 0.00

H

ATOM 3767 HA GLN A 407 -5.552 34.049 29.340 1.00 0.00

H

ATOM 3768 HB3 GLN A 407 -3.314 31.992 29.052 1.00 0.00

H

ATOM 3769 HB2 GLN A 407 -3.077 33.607 29.640 1.00 0.00

H

ATOM 3770 HG3 GLN A 407 -4.020 32.836 26.823 1.00 0.00

H

ATOM 3771 HG2 GLN A 407 -2.397 33.286 27.265 1.00 0.00

H

ATOM 3772 HE22 GLN A 407 -4.106 36.247 25.728 1.00 0.00

H

ATOM 3773 HE21 GLN A 407 -3.836 34.596 25.230 1.00 0.00

H

ATOM 3774 N PRO A 408 -6.945 32.402 27.803 1.00 25.83

N

ATOM 3775 CA PRO A 408 -7.709 31.492 26.920 1.00 27.42

C

ATOM 3776 C PRO A 408 -6.873 30.446 26.156 1.00 29.12

C

ATOM 3777 O PRO A 408 -5.838 30.788 25.583 1.00 29.12

O

ATOM 3778 CB PRO A 408 -8.428 32.446 25.953 1.00 25.96

C
ATOM 3779 CG PRO A 408 -8.639 33.708 26.768 1.00 25.60
C
ATOM 3780 CD PRO A 408 -7.367 33.791 27.600 1.00 26.30
C
ATOM 3781 HA PRO A 408 -8.448 30.991 27.549 1.00 0.00
H
ATOM 3782 HB3 PRO A 408 -9.366 32.035 25.578 1.00 0.00
H
ATOM 3783 HB2 PRO A 408 -7.799 32.675 25.091 1.00 0.00
H
ATOM 3784 HG3 PRO A 408 -9.496 33.571 27.428 1.00 0.00
H
ATOM 3785 HG2 PRO A 408 -8.819 34.595 26.161 1.00 0.00
H
ATOM 3786 HD2 PRO A 408 -6.590 34.322 27.050 1.00 0.00
H
ATOM 3787 HD3 PRO A 408 -7.563 34.322 28.532 1.00 0.00
H
ATOM 3788 N GLU A 409 -7.353 29.190 26.194 1.00 31.66
N
ATOM 3789 CA GLU A 409 -6.799 27.998 25.532 1.00 34.64
C
ATOM 3790 C GLU A 409 -5.476 27.471 26.135 1.00 34.56
C
ATOM 3791 O GLU A 409 -4.907 26.527 25.582 1.00 34.24
O
ATOM 3792 CB GLU A 409 -6.699 28.196 23.993 1.00 37.88
C
ATOM 3793 CG GLU A 409 -7.963 28.744 23.291 1.00 43.65
C
ATOM 3794 CD GLU A 409 -9.205 27.879 23.504 1.00 46.69
C
ATOM 3795 OE1 GLU A 409 -9.885 28.098 24.530 1.00 49.95
O
ATOM 3796 OE2 GLU A 409 -9.448 27.010 22.639 1.00 49.48

O1-
ATOM 3797 H GLU A 409 -8.226 29.030 26.675 1.00 0.00
H
ATOM 3798 HA GLU A 409 -7.525 27.207 25.721 1.00 0.00
H
ATOM 3799 HB3 GLU A 409 -6.441 27.246 23.525 1.00 0.00
H
ATOM 3800 HB2 GLU A 409 -5.864 28.858 23.761 1.00 0.00
H
ATOM 3801 HG3 GLU A 409 -7.775 28.823 22.219 1.00 0.00
H
ATOM 3802 HG2 GLU A 409 -8.181 29.758 23.626 1.00 0.00
H
ATOM 3803 N CYS A 410 -5.027 28.059 27.253 1.00 33.81
N
ATOM 3804 CA CYS A 410 -3.796 27.690 27.949 1.00 35.08
C
ATOM 3805 C CYS A 410 -3.923 26.342 28.688 1.00 34.78
C
ATOM 3806 O CYS A 410 -2.956 25.585 28.738 1.00 34.24
O
ATOM 3807 CB CYS A 410 -3.338 28.824 28.885 1.00 36.13
C
ATOM 3808 SG CYS A 410 -1.683 28.536 29.555 1.00 41.53
S
ATOM 3809 H CYS A 410 -5.515 28.874 27.606 1.00 0.00
H
ATOM 3810 HA CYS A 410 -3.021 27.563 27.190 1.00 0.00
H
ATOM 3811 HB3 CYS A 410 -4.035 28.956 29.715 1.00 0.00
H
ATOM 3812 HB2 CYS A 410 -3.308 29.766 28.339 1.00 0.00
H
ATOM 3813 HG CYS A 410 -1.680 29.655 30.296 1.00 0.00
H
ATOM 3814 N SER A 411 -5.124 26.015 29.185 1.00 34.62

N
ATOM 3815 CA SER A 411 -5.435 24.774 29.903 1.00 35.60
C
ATOM 3816 C SER A 411 -5.102 23.485 29.120 1.00 35.51
C
ATOM 3817 O SER A 411 -4.722 22.493 29.741 1.00 35.62
O
ATOM 3818 CB SER A 411 -6.907 24.823 30.361 1.00 36.58
C
ATOM 3819 OG SER A 411 -7.805 24.765 29.268 1.00 38.87
O
ATOM 3820 H SER A 411 -5.910 26.623 29.003 1.00 0.00
H
ATOM 3821 HA SER A 411 -4.813 24.769 30.801 1.00 0.00
H
ATOM 3822 HB3 SER A 411 -7.101 25.729 30.935 1.00 0.00
H
ATOM 3823 HB2 SER A 411 -7.122 23.983 31.022 1.00 0.00
H
ATOM 3824 HG SER A 411 -8.704 24.775 29.607 1.00 0.00
H
ATOM 3825 N MET A 412 -5.204 23.543 27.780 1.00 35.70
N
ATOM 3826 CA MET A 412 -4.820 22.470 26.862 1.00 36.25
C
ATOM 3827 C MET A 412 -3.297 22.350 26.677 1.00 34.53
C
ATOM 3828 O MET A 412 -2.821 21.250 26.408 1.00 34.48
O
ATOM 3829 CB MET A 412 -5.503 22.689 25.498 1.00 40.58
C
ATOM 3830 CG MET A 412 -7.038 22.718 25.570 1.00 45.64
C
ATOM 3831 SD MET A 412 -7.861 22.788 23.957 1.00 52.62
S
ATOM 3832 CE MET A 412 -7.462 24.485 23.464 1.00 50.53

C
ATOM 3833 H MET A 412 -5.533 24.397 27.353 1.00 0.00
H
ATOM 3834 HA MET A 412 -5.175 21.522 27.273 1.00 0.00
H
ATOM 3835 HB3 MET A 412 -5.200 21.901 24.807 1.00 0.00
H
ATOM 3836 HB2 MET A 412 -5.144 23.623 25.065 1.00 0.00
H
ATOM 3837 HG3 MET A 412 -7.380 23.566 26.164 1.00 0.00
H
ATOM 3838 HG2 MET A 412 -7.393 21.821 26.080 1.00 0.00
H
ATOM 3839 HE1 MET A 412 -7.936 24.717 22.511 1.00 0.00
H
ATOM 3840 HE2 MET A 412 -7.826 25.190 24.212 1.00 0.00
H
ATOM 3841 HE3 MET A 412 -6.387 24.619 23.352 1.00 0.00
H
ATOM 3842 N LYS A 413 -2.562 23.460 26.826 1.00 31.53
N
ATOM 3843 CA LYS A 413 -1.100 23.488 26.755 1.00 30.85
C
ATOM 3844 C LYS A 413 -0.448 23.024 28.069 1.00 30.52
C
ATOM 3845 O LYS A 413 0.689 22.566 28.036 1.00 30.68
O
ATOM 3846 CB LYS A 413 -0.622 24.901 26.366 1.00 31.05
C
ATOM 3847 CG LYS A 413 -1.046 25.328 24.949 1.00 32.36
C
ATOM 3848 CD LYS A 413 -0.635 26.775 24.640 1.00 32.77
C
ATOM 3849 CE LYS A 413 -0.761 27.160 23.157 1.00 34.50
C
ATOM 3850 NZ LYS A 413 0.329 26.580 22.354 1.00 36.17

N1+
ATOM 3851 H LYS A 413 -3.001 24.306 27.168 1.00 0.00
H
ATOM 3852 HA LYS A 413 -0.770 22.794 25.979 1.00 0.00
H
ATOM 3853 HB3 LYS A 413 0.467 24.931 26.422 1.00 0.00
H
ATOM 3854 HB2 LYS A 413 -0.976 25.628 27.096 1.00 0.00
H
ATOM 3855 HG3 LYS A 413 -2.128 25.236 24.836 1.00 0.00
H
ATOM 3856 HG2 LYS A 413 -0.604 24.648 24.221 1.00 0.00
H
ATOM 3857 HD3 LYS A 413 0.388 26.940 24.979 1.00 0.00
H
ATOM 3858 HD2 LYS A 413 -1.258 27.449 25.229 1.00 0.00
H
ATOM 3859 HE3 LYS A 413 -0.713 28.243 23.044 1.00 0.00
H
ATOM 3860 HE2 LYS A 413 -1.724 26.847 22.754 1.00 0.00
H
ATOM 3861 HZ1 LYS A 413 0.285 25.573 22.393 1.00 0.00
H
ATOM 3862 HZ2 LYS A 413 0.242 26.889 21.397 1.00 0.00
H
ATOM 3863 HZ3 LYS A 413 1.217 26.894 22.724 1.00 0.00
H
ATOM 3864 N LEU A 414 -1.184 23.115 29.184 1.00 30.09
N
ATOM 3865 CA LEU A 414 -0.840 22.516 30.473 1.00 28.96
C
ATOM 3866 C LEU A 414 -1.154 20.996 30.465 1.00 28.33
C
ATOM 3867 O LEU A 414 -1.345 20.416 29.395 1.00 29.10
O
ATOM 3868 CB LEU A 414 -1.566 23.306 31.593 1.00 28.64

C
ATOM 3869 CG LEU A 414 -1.149 24.799 31.682 1.00 30.41
C
ATOM 3870 CD1 LEU A 414 -2.083 25.601 32.604 1.00 31.19
C
ATOM 3871 CD2 LEU A 414 0.333 24.999 32.056 1.00 30.79
C
ATOM 3872 H LEU A 414 -2.102 23.531 29.113 1.00 0.00
H
ATOM 3873 HA LEU A 414 0.234 22.612 30.621 1.00 0.00
H
ATOM 3874 HB3 LEU A 414 -1.391 22.843 32.564 1.00 0.00
H
ATOM 3875 HB2 LEU A 414 -2.641 23.234 31.423 1.00 0.00
H
ATOM 3876 HG LEU A 414 -1.282 25.238 30.692 1.00 0.00
H
ATOM 3877 HD11 LEU A 414 -2.197 26.622 32.240 1.00 0.00
H
ATOM 3878 HD12 LEU A 414 -3.081 25.170 32.641 1.00 0.00
H
ATOM 3879 HD13 LEU A 414 -1.710 25.656 33.628 1.00 0.00
H
ATOM 3880 HD21 LEU A 414 0.466 25.714 32.868 1.00 0.00
H
ATOM 3881 HD22 LEU A 414 0.818 24.072 32.355 1.00 0.00
H
ATOM 3882 HD23 LEU A 414 0.888 25.389 31.202 1.00 0.00
H
ATOM 3883 N THR A 415 -1.183 20.348 31.637 1.00 27.70
N
ATOM 3884 CA THR A 415 -1.609 18.950 31.778 1.00 26.69
C
ATOM 3885 C THR A 415 -2.673 18.857 32.890 1.00 26.91
C
ATOM 3886 O THR A 415 -2.705 19.753 33.744 1.00 26.39

O
ATOM 3887 CB THR A 415 -0.427 17.988 32.124 1.00 26.61
C
ATOM 3888 OG1 THR A 415 -0.137 17.869 33.514 1.00 25.35
O
ATOM 3889 CG2 THR A 415 0.859 18.268 31.339 1.00 26.48
C
ATOM 3890 H THR A 415 -1.083 20.844 32.508 1.00 0.00
H
ATOM 3891 HA THR A 415 -2.066 18.616 30.847 1.00 0.00
H
ATOM 3892 HB THR A 415 -0.743 16.991 31.814 1.00 0.00
H
ATOM 3893 HG1 THR A 415 0.628 17.264 33.622 1.00 0.00
H
ATOM 3894 HG21 THR A 415 1.563 17.440 31.413 1.00 0.00
H
ATOM 3895 HG22 THR A 415 0.652 18.413 30.279 1.00 0.00
H
ATOM 3896 HG23 THR A 415 1.357 19.162 31.709 1.00 0.00
H
ATOM 3897 N PRO A 416 -3.469 17.759 32.934 1.00 27.60
N
ATOM 3898 CA PRO A 416 -4.408 17.542 34.056 1.00 27.16
C
ATOM 3899 C PRO A 416 -3.767 17.453 35.455 1.00 27.48
C
ATOM 3900 O PRO A 416 -4.377 17.923 36.412 1.00 26.90
O
ATOM 3901 CB PRO A 416 -5.139 16.236 33.696 1.00 29.00
C
ATOM 3902 CG PRO A 416 -5.006 16.124 32.189 1.00 28.36
C
ATOM 3903 CD PRO A 416 -3.627 16.710 31.923 1.00 28.22
C
ATOM 3904 HA PRO A 416 -5.124 18.366 34.042 1.00 0.00

H
ATOM 3905 HB3 PRO A 416 -6.180 16.235 34.019 1.00 0.00
H
ATOM 3906 HB2 PRO A 416 -4.653 15.376 34.160 1.00 0.00
H
ATOM 3907 HG3 PRO A 416 -5.764 16.749 31.713 1.00 0.00
H
ATOM 3908 HG2 PRO A 416 -5.126 15.107 31.816 1.00 0.00
H
ATOM 3909 HD2 PRO A 416 -2.859 15.950 32.073 1.00 0.00
H
ATOM 3910 HD3 PRO A 416 -3.570 17.064 30.894 1.00 0.00
H
ATOM 3911 N LEU A 417 -2.536 16.922 35.530 1.00 25.90
N
ATOM 3912 CA LEU A 417 -1.751 16.829 36.759 1.00 25.39
C
ATOM 3913 C LEU A 417 -1.189 18.201 37.169 1.00 24.67
C
ATOM 3914 O LEU A 417 -1.290 18.571 38.335 1.00 24.55
O
ATOM 3915 CB LEU A 417 -0.643 15.771 36.575 1.00 25.12
C
ATOM 3916 CG LEU A 417 0.180 15.431 37.839 1.00 25.26
C
ATOM 3917 CD1 LEU A 417 -0.701 14.863 38.971 1.00 24.40
C
ATOM 3918 CD2 LEU A 417 1.342 14.478 37.487 1.00 25.23
C
ATOM 3919 H LEU A 417 -2.059 16.652 34.682 1.00 0.00
H
ATOM 3920 HA LEU A 417 -2.420 16.493 37.553 1.00 0.00
H
ATOM 3921 HB3 LEU A 417 0.036 16.121 35.799 1.00 0.00
H
ATOM 3922 HB2 LEU A 417 -1.086 14.851 36.191 1.00 0.00

H
ATOM 3923 HG LEU A 417 0.636 16.349 38.211 1.00 0.00
H
ATOM 3924 HD11 LEU A 417 -0.197 14.091 39.551 1.00 0.00
H
ATOM 3925 HD12 LEU A 417 -0.984 15.646 39.673 1.00 0.00
H
ATOM 3926 HD13 LEU A 417 -1.626 14.427 38.590 1.00 0.00
H
ATOM 3927 HD21 LEU A 417 1.272 13.515 37.993 1.00 0.00
H
ATOM 3928 HD22 LEU A 417 1.391 14.268 36.419 1.00 0.00
H
ATOM 3929 HD23 LEU A 417 2.297 14.919 37.765 1.00 0.00
H
ATOM 3930 N VAL A 418 -0.665 18.976 36.209 1.00 24.66
N
ATOM 3931 CA VAL A 418 -0.203 20.349 36.436 1.00 25.57
C
ATOM 3932 C VAL A 418 -1.343 21.288 36.890 1.00 26.33
C
ATOM 3933 O VAL A 418 -1.122 22.156 37.738 1.00 27.09
O
ATOM 3934 CB VAL A 418 0.517 20.894 35.167 1.00 25.14
C
ATOM 3935 CG1 VAL A 418 0.598 22.424 35.001 1.00 26.89
C
ATOM 3936 CG2 VAL A 418 1.930 20.297 35.092 1.00 25.97
C
ATOM 3937 H VAL A 418 -0.606 18.627 35.255 1.00 0.00
H
ATOM 3938 HA VAL A 418 0.517 20.317 37.257 1.00 0.00
H
ATOM 3939 HB VAL A 418 -0.026 20.524 34.297 1.00 0.00
H
ATOM 3940 HG11 VAL A 418 1.227 22.687 34.150 1.00 0.00

H
ATOM 3941 HG12 VAL A 418 -0.380 22.870 34.818 1.00 0.00
H
ATOM 3942 HG13 VAL A 418 1.031 22.903 35.878 1.00 0.00
H
ATOM 3943 HG21 VAL A 418 2.405 20.501 34.131 1.00 0.00
H
ATOM 3944 HG22 VAL A 418 2.569 20.704 35.876 1.00 0.00
H
ATOM 3945 HG23 VAL A 418 1.905 19.217 35.229 1.00 0.00
H
ATOM 3946 N LEU A 419 -2.551 21.055 36.373 1.00 26.33
N
ATOM 3947 CA LEU A 419 -3.786 21.727 36.776 1.00 27.78
C
ATOM 3948 C LEU A 419 -4.354 21.222 38.121 1.00 29.03
C
ATOM 3949 O LEU A 419 -5.278 21.850 38.633 1.00 30.30
O
ATOM 3950 CB LEU A 419 -4.811 21.585 35.631 1.00 28.60
C
ATOM 3951 CG LEU A 419 -4.461 22.437 34.387 1.00 30.69
C
ATOM 3952 CD1 LEU A 419 -5.229 21.959 33.137 1.00 30.94
C
ATOM 3953 CD2 LEU A 419 -4.650 23.945 34.661 1.00 31.11
C
ATOM 3954 H LEU A 419 -2.646 20.362 35.636 1.00 0.00
H
ATOM 3955 HA LEU A 419 -3.576 22.786 36.922 1.00 0.00
H
ATOM 3956 HB3 LEU A 419 -5.810 21.859 35.977 1.00 0.00
H
ATOM 3957 HB2 LEU A 419 -4.881 20.530 35.362 1.00 0.00
H
ATOM 3958 HG LEU A 419 -3.404 22.288 34.162 1.00 0.00

H
ATOM 3959 HD11 LEU A 419 -5.874 22.726 32.713 1.00 0.00
H
ATOM 3960 HD12 LEU A 419 -4.534 21.657 32.352 1.00 0.00
H
ATOM 3961 HD13 LEU A 419 -5.861 21.096 33.346 1.00 0.00
H
ATOM 3962 HD21 LEU A 419 -5.171 24.466 33.858 1.00 0.00
H
ATOM 3963 HD22 LEU A 419 -5.211 24.131 35.578 1.00 0.00
H
ATOM 3964 HD23 LEU A 419 -3.684 24.434 34.781 1.00 0.00
H
ATOM 3965 N GLU A 420 -3.805 20.135 38.679 1.00 29.76
N
ATOM 3966 CA GLU A 420 -4.226 19.534 39.946 1.00 31.06
C
ATOM 3967 C GLU A 420 -3.201 19.820 41.064 1.00 30.59
C
ATOM 3968 O GLU A 420 -3.596 20.026 42.211 1.00 30.30
O
ATOM 3969 CB GLU A 420 -4.517 18.035 39.670 1.00 32.62
C
ATOM 3970 CG GLU A 420 -5.065 17.166 40.825 1.00 36.69
C
ATOM 3971 CD GLU A 420 -4.033 16.576 41.787 1.00 38.47
C
ATOM 3972 OE1 GLU A 420 -2.821 16.653 41.496 1.00 40.46
O
ATOM 3973 OE2 GLU A 420 -4.482 16.051 42.827 1.00 41.56
O1-
ATOM 3974 H GLU A 420 -3.065 19.650 38.191 1.00 0.00
H
ATOM 3975 HA GLU A 420 -5.165 19.985 40.275 1.00 0.00
H
ATOM 3976 HB3 GLU A 420 -3.630 17.562 39.250 1.00 0.00

H
ATOM 3977 HB2 GLU A 420 -5.258 17.990 38.872 1.00 0.00
H
ATOM 3978 HG3 GLU A 420 -5.599 16.318 40.396 1.00 0.00
H
ATOM 3979 HG2 GLU A 420 -5.804 17.730 41.395 1.00 0.00
H
ATOM 3980 N VAL A 421 -1.908 19.852 40.712 1.00 31.25
N
ATOM 3981 CA VAL A 421 -0.808 20.176 41.616 1.00 32.00
C
ATOM 3982 C VAL A 421 -0.633 21.696 41.768 1.00 32.63
C
ATOM 3983 O VAL A 421 -0.726 22.198 42.886 1.00 32.82
O
ATOM 3984 CB VAL A 421 0.526 19.523 41.144 1.00 31.60
C
ATOM 3985 CG1 VAL A 421 1.807 20.021 41.849 1.00 31.31
C
ATOM 3986 CG2 VAL A 421 0.432 17.996 41.281 1.00 31.20
C
ATOM 3987 H VAL A 421 -1.653 19.526 39.784 1.00 0.00
H
ATOM 3988 HA VAL A 421 -1.045 19.781 42.606 1.00 0.00
H
ATOM 3989 HB VAL A 421 0.656 19.737 40.082 1.00 0.00
H
ATOM 3990 HG11 VAL A 421 2.676 19.428 41.563 1.00 0.00
H
ATOM 3991 HG12 VAL A 421 2.045 21.054 41.593 1.00 0.00
H
ATOM 3992 HG13 VAL A 421 1.711 19.965 42.933 1.00 0.00
H
ATOM 3993 HG21 VAL A 421 1.359 17.520 40.981 1.00 0.00
H
ATOM 3994 HG22 VAL A 421 0.226 17.695 42.308 1.00 0.00

H
ATOM 3995 HG23 VAL A 421 -0.362 17.591 40.656 1.00 0.00
H
ATOM 3996 N PHE A 422 -0.372 22.395 40.653 1.00 33.47
N
ATOM 3997 CA PHE A 422 -0.124 23.839 40.666 1.00 34.95
C
ATOM 3998 C PHE A 422 -1.435 24.630 40.545 1.00 35.99
C
ATOM 3999 O PHE A 422 -1.526 25.737 41.071 1.00 36.37
O
ATOM 4000 CB PHE A 422 0.869 24.228 39.550 1.00 34.60
C
ATOM 4001 CG PHE A 422 2.229 23.556 39.644 1.00 34.76
C
ATOM 4002 CD1 PHE A 422 3.073 23.821 40.742 1.00 34.88
C
ATOM 4003 CD2 PHE A 422 2.640 22.616 38.675 1.00 35.16
C
ATOM 4004 CE1 PHE A 422 4.294 23.171 40.849 1.00 34.92
C
ATOM 4005 CE2 PHE A 422 3.878 21.997 38.788 1.00 34.70
C
ATOM 4006 CZ PHE A 422 4.702 22.271 39.873 1.00 34.73
C
ATOM 4007 H PHE A 422 -0.342 21.939 39.753 1.00 0.00
H
ATOM 4008 HA PHE A 422 0.326 24.122 41.621 1.00 0.00
H
ATOM 4009 HB3 PHE A 422 1.030 25.307 39.577 1.00 0.00
H
ATOM 4010 HB2 PHE A 422 0.441 24.026 38.570 1.00 0.00
H
ATOM 4011 HD1 PHE A 422 2.771 24.519 41.510 1.00 0.00
H
ATOM 4012 HD2 PHE A 422 2.004 22.382 37.835 1.00 0.00

H

ATOM 4013 HE1 PHE A 422 4.924 23.371 41.702 1.00 0.00

H

ATOM 4014 HE2 PHE A 422 4.204 21.298 38.032 1.00 0.00

H

ATOM 4015 HZ PHE A 422 5.661 21.779 39.958 1.00 0.00

H

ATOM 4016 N GLY A 423 -2.435 24.046 39.867 1.00 37.39

N

ATOM 4017 CA GLY A 423 -3.792 24.594 39.738 1.00 38.89

C

ATOM 4018 C GLY A 423 -4.717 24.121 40.876 1.00 39.70

C

ATOM 4019 O GLY A 423 -4.361 23.320 41.743 1.00 40.52

O

ATOM 4020 H GLY A 423 -2.246 23.148 39.442 1.00 0.00

H

ATOM 4021 HA3 GLY A 423 -4.215 24.302 38.778 1.00 0.00

H

ATOM 4022 HA2 GLY A 423 -3.785 25.686 39.738 1.00 0.00

H

ATOM 4023 HXT GLY A 423 -5.720 24.546 40.849 1.00 0.00

H

TER 4024 GLY A 423

HETATM 4025 C1 UNK Z 999 16.164 20.620 30.977 1.00 0.00

C

HETATM 4026 N1 UNK Z 999 15.110 20.442 31.981 1.00 0.00

N

HETATM 4027 C2 UNK Z 999 15.309 19.601 33.011 1.00 0.00

C

HETATM 4028 N2 UNK Z 999 14.433 20.007 33.928 1.00 0.00

N

HETATM 4029 C3 UNK Z 999 13.925 19.473 35.243 1.00 0.00

C

HETATM 4030 C4 UNK Z 999 14.534 18.089 35.656 1.00 0.00

C

HETATM 4031 C5 UNK Z 999 14.035 16.925 34.753 1.00 0.00
C
HETATM 4032 O1 UNK Z 999 14.631 15.695 35.176 1.00 0.00
O
HETATM 4033 C6 UNK Z 999 14.567 14.585 34.407 1.00 0.00
C
HETATM 4034 C7 UNK Z 999 15.407 13.434 34.953 1.00 0.00
C
HETATM 4035 BR1 UNK Z 999 15.245 11.825 33.777 1.00 0.00
Br
HETATM 4036 O2 UNK Z 999 13.905 14.465 33.377 1.00 0.00
O
HETATM 4037 C8 UNK Z 999 12.490 16.882 34.654 1.00 0.00
C
HETATM 4038 C9 UNK Z 999 11.942 18.265 34.238 1.00 0.00
C
HETATM 4039 C10 UNK Z 999 12.346 19.411 35.202 1.00 0.00
C
HETATM 4040 C11 UNK Z 999 11.755 19.086 36.603 1.00 0.00
C
HETATM 4041 C12 UNK Z 999 11.817 20.824 34.672 1.00 0.00
C
HETATM 4042 C13 UNK Z 999 10.513 21.377 35.330 1.00 0.00
C
HETATM 4043 C14 UNK Z 999 10.153 22.822 34.939 1.00 0.00
C
HETATM 4044 C15 UNK Z 999 11.334 23.817 35.126 1.00 0.00
C
HETATM 4045 C16 UNK Z 999 11.795 23.972 36.612 1.00 0.00
C
HETATM 4046 C17 UNK Z 999 12.453 23.298 34.178 1.00 0.00
C
HETATM 4047 C18 UNK Z 999 13.411 24.486 34.016 1.00 0.00
C
HETATM 4048 C19 UNK Z 999 12.490 25.722 34.107 1.00 0.00
C

HETATM 4049 C20 UNK Z 999 11.071 25.220 34.503 1.00 0.00
C
HETATM 4050 C21 UNK Z 999 10.234 26.272 35.323 1.00 0.00
C
HETATM 4051 C22 UNK Z 999 9.835 27.489 34.438 1.00 0.00
C
HETATM 4052 C23 UNK Z 999 8.652 27.165 33.503 1.00 0.00
C
HETATM 4053 C24 UNK Z 999 8.331 28.179 32.399 1.00 0.00
C
HETATM 4054 C25 UNK Z 999 7.460 27.580 31.270 1.00 0.00
C
HETATM 4055 C26 UNK Z 999 6.216 26.839 31.800 1.00 0.00
C
HETATM 4056 C27 UNK Z 999 8.278 26.660 30.348 1.00 0.00
C
HETATM 4057 C28 UNK Z 999 8.967 25.715 36.027 1.00 0.00
C
HETATM 4058 C29 UNK Z 999 13.002 21.874 34.539 1.00 0.00
C
HETATM 4059 N3 UNK Z 999 14.002 21.302 33.576 1.00 0.00
N
HETATM 4060 C30 UNK Z 999 14.239 21.435 32.261 1.00 0.00
C
HETATM 4061 O3 UNK Z 999 13.769 22.267 31.484 1.00 0.00
O
HETATM 4062 C31 UNK Z 999 13.846 21.829 35.834 1.00 0.00
C
HETATM 4063 C32 UNK Z 999 14.307 20.614 36.188 1.00 0.00
C
HETATM 4064 O4 UNK Z 999 16.045 18.620 33.070 1.00 0.00
O
HETATM 4065 H1 UNK Z 999 11.492 20.687 33.639 1.00 0.00
H
HETATM 4066 H2 UNK Z 999 10.546 25.042 33.564 1.00 0.00
H

HETATM 4067 H3 UNK Z 999 12.004 23.169 33.188 1.00 0.00
H
HETATM 4068 H4 UNK Z 999 14.396 17.095 33.739 1.00 0.00
H
HETATM 4069 H5 UNK Z 999 10.878 26.653 36.119 1.00 0.00
H
HETATM 4070 H6 UNK Z 999 17.152 20.371 31.368 1.00 0.00
H
HETATM 4071 H7 UNK Z 999 16.203 21.636 30.578 1.00 0.00
H
HETATM 4072 H8 UNK Z 999 15.968 19.946 30.144 1.00 0.00
H
HETATM 4073 H9 UNK Z 999 15.622 18.154 35.609 1.00 0.00
H
HETATM 4074 H10 UNK Z 999 14.275 17.879 36.693 1.00 0.00
H
HETATM 4075 H11 UNK Z 999 16.457 13.723 34.977 1.00 0.00
H
HETATM 4076 H12 UNK Z 999 15.081 13.166 35.958 1.00 0.00
H
HETATM 4077 H13 UNK Z 999 12.164 16.184 33.884 1.00 0.00
H
HETATM 4078 H14 UNK Z 999 12.059 16.557 35.596 1.00 0.00
H
HETATM 4079 H15 UNK Z 999 12.302 18.500 33.240 1.00 0.00
H
HETATM 4080 H16 UNK Z 999 10.853 18.212 34.177 1.00 0.00
H
HETATM 4081 H17 UNK Z 999 10.668 19.007 36.576 1.00 0.00
H
HETATM 4082 H18 UNK Z 999 12.024 19.819 37.355 1.00 0.00
H
HETATM 4083 H19 UNK Z 999 12.134 18.166 37.019 1.00 0.00
H
HETATM 4084 H20 UNK Z 999 9.684 20.699 35.130 1.00 0.00
H

HETATM 4085 H21 UNK Z 999 10.617 21.382 36.409 1.00 0.00
H
HETATM 4086 H22 UNK Z 999 9.821 22.823 33.898 1.00 0.00
H
HETATM 4087 H23 UNK Z 999 9.281 23.125 35.511 1.00 0.00
H
HETATM 4088 H24 UNK Z 999 12.707 24.563 36.692 1.00 0.00
H
HETATM 4089 H25 UNK Z 999 11.988 23.020 37.086 1.00 0.00
H
HETATM 4090 H26 UNK Z 999 11.078 24.458 37.262 1.00 0.00
H
HETATM 4091 H27 UNK Z 999 14.174 24.498 34.794 1.00 0.00
H
HETATM 4092 H28 UNK Z 999 13.935 24.455 33.059 1.00 0.00
H
HETATM 4093 H29 UNK Z 999 12.884 26.439 34.827 1.00 0.00
H
HETATM 4094 H30 UNK Z 999 12.452 26.233 33.144 1.00 0.00
H
HETATM 4095 H31 UNK Z 999 9.569 28.337 35.072 1.00 0.00
H
HETATM 4096 H32 UNK Z 999 10.691 27.821 33.850 1.00 0.00
H
HETATM 4097 H33 UNK Z 999 8.813 26.191 33.048 1.00 0.00
H
HETATM 4098 H34 UNK Z 999 7.748 27.063 34.106 1.00 0.00
H
HETATM 4099 H35 UNK Z 999 7.816 29.019 32.855 1.00 0.00
H
HETATM 4100 H36 UNK Z 999 9.246 28.586 31.972 1.00 0.00
H
HETATM 4101 H37 UNK Z 999 7.102 28.412 30.660 1.00 0.00
H
HETATM 4102 H38 UNK Z 999 6.477 25.995 32.439 1.00 0.00
H

HETATM 4103 H39 UNK Z 999 5.646 26.424 30.977 1.00 0.00
H
HETATM 4104 H40 UNK Z 999 5.558 27.494 32.367 1.00 0.00
H
HETATM 4105 H41 UNK Z 999 8.747 25.837 30.885 1.00 0.00
H
HETATM 4106 H42 UNK Z 999 9.067 27.215 29.842 1.00 0.00
H
HETATM 4107 H43 UNK Z 999 7.645 26.219 29.580 1.00 0.00
H
HETATM 4108 H44 UNK Z 999 8.261 25.274 35.324 1.00 0.00
H
HETATM 4109 H45 UNK Z 999 8.446 26.504 36.572 1.00 0.00
H
HETATM 4110 H46 UNK Z 999 9.168 24.939 36.751 1.00 0.00
H
HETATM 4111 H47 UNK Z 999 14.050 22.697 36.437 1.00 0.00
H
HETATM 4112 H48 UNK Z 999 14.846 20.443 37.102 1.00 0.00
H
HETATM 4113 X-7 UNK 0 16.793 26.712 33.893 1.00 0.00
X
CONECT 3246 3252
CONECT 4018 4023
CONECT 3252 3246
CONECT 4023 4018
CONECT 4025 4026 4070 4071 4072
CONECT 4026 4025 4060 4027
CONECT 4027 4026 4028 4064
CONECT 4027 4064
CONECT 4028 4027 4059 4029
CONECT 4029 4028 4039 4063 4030
CONECT 4030 4029 4031 4073 4074
CONECT 4031 4030 4068 4032 4037
CONECT 4032 4031 4033
CONECT 4033 4032 4034 4036

CONECT 4033 4036
CONECT 4034 4033 4035 4075 4076
CONECT 4035 4034
CONECT 4036 4033
CONECT 4036 4033
CONECT 4037 4031 4038 4077 4078
CONECT 4038 4037 4039 4079 4080
CONECT 4039 4029 4038 4040 4041
CONECT 4040 4039 4081 4082 4083
CONECT 4041 4039 4058 4065 4042
CONECT 4065 4041
CONECT 4042 4041 4043 4084 4085
CONECT 4043 4042 4044 4086 4087
CONECT 4044 4043 4049 4045 4046
CONECT 4045 4044 4088 4089 4090
CONECT 4046 4044 4047 4067 4058
CONECT 4047 4046 4048 4091 4092
CONECT 4048 4047 4049 4093 4094
CONECT 4049 4044 4048 4066 4050
CONECT 4066 4049
CONECT 4050 4049 4069 4051 4057
CONECT 4051 4050 4052 4095 4096
CONECT 4052 4051 4053 4097 4098
CONECT 4053 4052 4054 4099 4100
CONECT 4054 4053 4055 4056 4101
CONECT 4055 4054 4102 4103 4104
CONECT 4056 4054 4105 4106 4107
CONECT 4057 4050 4108 4109 4110
CONECT 4067 4046
CONECT 4058 4041 4046 4059 4062
CONECT 4059 4028 4058 4060
CONECT 4060 4026 4059 4061
CONECT 4060 4061
CONECT 4061 4060
CONECT 4061 4060
CONECT 4062 4058 4063 4111

CONECT 4062 4063
CONECT 4063 4029 4062 4112
CONECT 4063 4062
CONECT 4064 4027
CONECT 4064 4027
CONECT 4068 4031
CONECT 4069 4050
CONECT 4070 4025
CONECT 4071 4025
CONECT 4072 4025
CONECT 4073 4030
CONECT 4074 4030
CONECT 4075 4034
CONECT 4076 4034
CONECT 4077 4037
CONECT 4078 4037
CONECT 4079 4038
CONECT 4080 4038
CONECT 4081 4040
CONECT 4082 4040
CONECT 4083 4040
CONECT 4084 4042
CONECT 4085 4042
CONECT 4086 4043
CONECT 4087 4043
CONECT 4088 4045
CONECT 4089 4045
CONECT 4090 4045
CONECT 4091 4047
CONECT 4092 4047
CONECT 4093 4048
CONECT 4094 4048
CONECT 4095 4051
CONECT 4096 4051
CONECT 4097 4052
CONECT 4098 4052

CONECT 4099 4053
CONECT 4100 4053
CONECT 4101 4054
CONECT 4102 4055
CONECT 4103 4055
CONECT 4104 4055
CONECT 4105 4056
CONECT 4106 4056
CONECT 4107 4056
CONECT 4108 4057
CONECT 4109 4057
CONECT 4110 4057
CONECT 4111 4062
CONECT 4112 4063
ENDMDL
END
